# Cancer outcomes of pregnancy after diagnosis of breast cancer in premenopausal women: an updated systematic review and meta-analysis

**DOI:** 10.3389/fonc.2025.1644566

**Published:** 2025-10-14

**Authors:** Heting Mei, Qingya Song, Wenping Lu, Xiyue Wang, Jiaxin Liu, Weijia Zhang, Lei Chang, Zhili Zhuo

**Affiliations:** ^1^ Department of Oncology, China Academy of Chinese Medical Sciences Guang’anmen Hospital, Beijing, China; ^2^ Graduate School, Beijing University of Chinese Medicine, Beijing, China

**Keywords:** breast cancer, pregnancy, cancer outcomes, meta-analysis, review

## Abstract

**Introduction:**

Breast cancer is a type of hormone-driven cancer, and pregnancy may increase the risk of recurrence in patients due to hormone surge. Our study was designed to explore the cancer outcomes of premenopausal women who had primary breast cancer and who became pregnant at any time after diagnosis.

**Methods:**

Searches were conducted in ten databases until July 2024, with no language restrictions. We analyzed aggregate data across all study populations and performed subgroup analyses by stage, estrogen receptor status, BRCA mutation status, HER2 status, previous treatment, lymph node status, interval between pregnancy and diagnosis, pregnancy type, reproductive status, and breastfeeding status.

**Results:**

Fifty-two studies were included. The pregnancy group had longer overall survival (n = 18) [RR = 1.09 (1.06, 1.12), P < 0.001]. Nine studies reported the same results [HR = 0.60 (0.48, 0.73), P < 0.001]. The disease-free survival (n = 7) of the pregnancy group was not significantly longer [HR = 0.90 (0.79, 1.02), P = 0.111]. The pregnancy group had longer breast cancer-specific survival (n = 3) [HR = 0.55 (0.40, 0.76), P < 0.001]. The pregnancy group had a lower recurrence rate (n = 17) [RR = 0.61 (0.55, 0.68), P < 0.001]. The pregnancy group had a higher loco-regional recurrence rate, although the difference was not statistically significant (n = 4) [RR = 1.04 (0.74, 1.47), P = 0.814]. The pregnancy group had a lower distant recurrence rate (n = 5) [RR = 0.50 (0.37, 0.68), P < 0.001]. The pregnancy group had a higher contralateral breast cancer rate, although the difference was not statistically significant (n = 3) [RR = 1.06 (0.76, 1.48), P = 0.742].

**Discussion:**

Our findings indicate that pregnancy after breast cancer does not lead to adverse cancer outcomes. Stage, estrogen receptor status, therapy choice (hormone, chemotherapy, or endocrine therapy combined with chemotherapy), and reproductive status are not associated with overall survival. BRCA2 mutation may negatively affect disease-free survival in pregnant patients with breast cancer.

**Systematic review registration:**

https://www.crd.york.ac.uk/prospero/, identifier CRD42024499971.

## Graphical Abstract

Impact of Pregnancy on Survival and Recurrence Outcomes in Breast Cancer Patients. Study flowchart illustrating data sources and analysis results for breast cancer survival. Begins with screening 35,018 records across ten databases, resulting in 52 included studies. Experimental group consists of 9,288 pregnant women; control group has 123,429 non-pregnant women. Graphs show pregnant women have higher survival probabilities and lower recurrence rates in several areas. Statistically significant increases were noted in overall and breast cancer-specific survival, along with significant reductions in certain recurrence rates. No statistically significant association was observed for disease-free survival, loco-regional recurrence, or contralateral breast cancer rates.

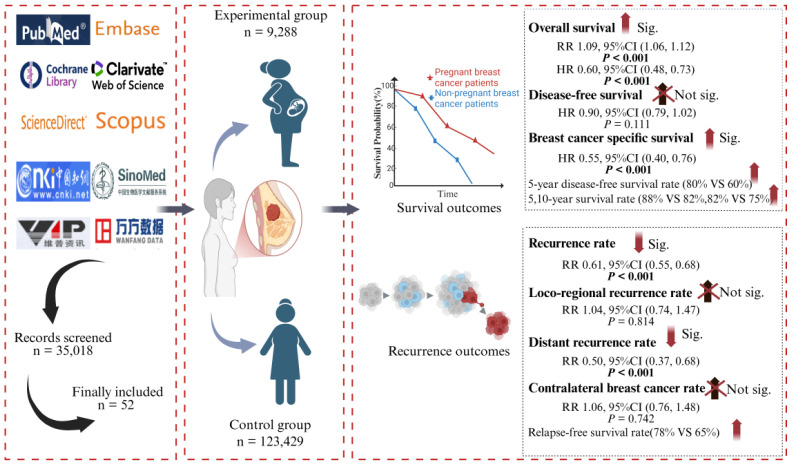

## 1 Introduction

Global statistical data for 2022 revealed that breast cancer (BC) was the most prevalent cancer among women, with 2,308,897 new cases reported worldwide (1). Owing to the improved survival and rejuvenation of patients with BC and the delay of reproductive age in modern people, the convergence of BC diagnosis age and reproductive age distribution is enhanced (2).

However, BC is a hormone-responsive cancer type, and with the increase in female hormones during pregnancy, there is a widespread apprehension that pregnancy may heighten the patient’s risk of recurrence (3). BC patients and their physicians still worry about the safety of babies and mothers after the diagnosis and treatment of BC (4).

There is no consensus on this topic. We aimed to explore the cancer outcomes of premenopausal women who had primary BC and who became pregnant at any time after diagnosis. Compared with previous related meta-analyses, this article included the latest evidence and more databases, such as the POSITIVE clinical trial (5), the study of young BC *BRCA* carriers (6), and Chinese databases, making it more comprehensive.

## 2 Materials and methods

### 2.1 Study subjects and outcome indicators

The experimental group included premenopausal women who had primary BC and who became pregnant at some point after diagnosis. The control group included nonpregnant women with BC.

The outcome indicators included overall survival (OS), disease-free survival(DFS), BC-specific survival, recurrence rate, loco-regional recurrence, distant recurrence, contralateral BC, 5-year relapse-free survival (RFS), 5-year DFS, and 5,10-year survival. OS is defined as the time from the start of a treatment or randomization to the time of death from any cause. DFS is defined as the time from the start of treatment or randomization until the recurrence of disease or death from any cause. BC-specific survival is defined as the time from the diagnosis of BC until death from BC or until the last follow-up visit if the patient is still alive and has not died from BC. RFS is defined as the time interval between complete response after antitumor treatment and the cutoff for recurrence or follow-up.

### 2.2 Search strategies

We conducted this systematic review and meta-analysis in accordance with the PRISMA 2020 guidelines (7) and MOOSE statement (8). The following databases were searched: PubMed, EMBASE, Cochrane Library, Science Direct, Web of Science, Scopus, CNKI, VIP, Wan Fang, and SinoMed, with no language restrictions until July 2024.

A combination of subject words and free words was used for retrieval. The search terms included “Breast Neoplasms” AND “Pregnancy OR Fertilization OR Parturition OR Fertility OR Obstetrics”. More details are provided in **Appendix A**. Registration: CRD 42024499971 (PROSPERO). The full protocol is available on the PROSPERO website.

### 2.3 Inclusion and exclusion criteria

The inclusion criteria were as follows: (1) premenopausal women; (2) studies reporting on pregnancy after primary BC diagnosis; (3) studies with available information on cancer outcomes; (4) prospective and retrospective cohort studies, prospective clinical trials, case–control studies and case series; and (5) outcomes that could be extracted or measured.

The exclusion criteria were as follows: (1) combination with other malignant tumors; (2) pregnancy-related BC (primary BC diagnosed during pregnancy or within one year after pregnancy (lactation); (3) case reports or case series including fewer than 10 patients; and (4) ongoing studies for which results were not presented or published at the time of the literature search.

### 2.4 Literature screening and data extraction

Two reviewers (Mei and Song) independently evaluated the titles and abstracts. A third author (Lu) resolved any disagreements. Full papers were reviewed, and data extraction was performed independently by five reviewers (Mei, Song, Wang, Liu, and Zhang), who were pilot tested by the other two reviewers (Chang and Zhuo). The extracted data included the first author, country, type of literature, year of publication, recruitment time, follow-up time, interval between diagnosis and pregnancy, tumor characteristics, previous treatment, number of patients in each group, cancer outcomes, and cancer outcomes in the subgroups.

### 2.5 Quality evaluation

Two authors (Mei and Song) independently conducted the quality evaluation, and the third author (Lu) resolved the differences. The quality assessment of cohort studies and case–control studies was conducted using the Newcastle–Ottawa Scale (NOS) (9), that of nonrandomized clinical trials was conducted using MINORS (10), and that of case series was conducted using the JBI critical appraisal tool (11).

### 2.6 Statistical analysis

The meta-analysis was carried out using Stata 17.0 software. For dichotomous variables, we selected either the relative risk (RR) or the odds ratio (OR) and their 95% confidence interval (CI) according to the type of study. If only the effect size hazard ratio (HR) was included in the original literature, the HR was directly combined. A table-based form was used to describe the results, and forest plots were used to graphically represent the results of the syntheses. The chi-square test was employed to assess heterogeneity among the studies. *P* < 0.05 and *I^2^* > 50% suggested statistical heterogeneity, in which case a random effects model was used. Otherwise, a fixed-effects model was used. Sensitivity analysis was performed using the one-by-one elimination method. Publication bias analysis was conducted utilizing both the funnel plot and Egger’s test. The significance level was α =0.05.

Subgroup analysis was used to investigate possible sources of heterogeneity, including BC stage, estrogen receptor(ER) status, *BRCA* mutation status, HER2 status, previous treatment, lymph node status, interval between pregnancy and diagnosis, pregnancy type, reproductive status, and breastfeeding status. Owing to the limitation of the available extracted data for subgroups, the majority of the outcome indicators were derived from only two articles. The sensitivity analysis chart composed solely of two articles had limited significance; consequently, this chart was excluded during the subgroup analysis.

## 3 Results

### 3.1 Literature retrieval results

**Figure 1:**
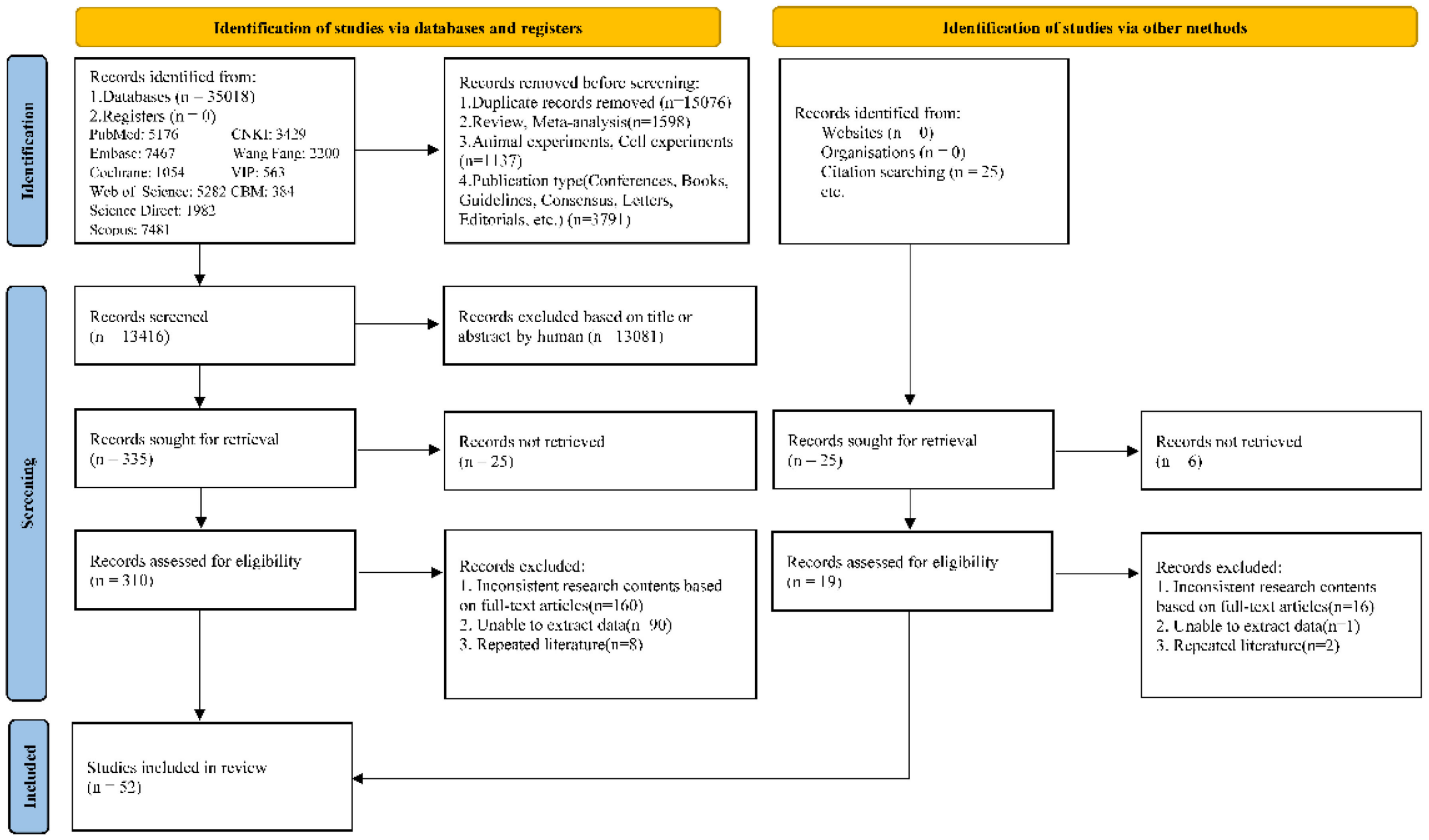
PRISMA 2020 flow diagram of study selection process.

Among the 35,018 identified records, 52 studies were included. The PRISMA flow diagram is shown in **Figure 1**. The pregnancy group included 9,288 patients, and nonpregnancy group included 123,429 patients. Basic information is presented in **Table 1** and **Table 2**.

**Table 1:** Basic information of included articles.

No.	Year	First-Author	Country	Study design	Recruitment time	Follow-up time	Interval between diagnosis and pregnancy
1	2009	Pentti M. Rissanen	Finland	Retrospective case series	1936-1959	At least 7 years from the date of admission for mammary carcinoma.	Between treatment and pregnancy:<1 year:24 pregnancies;<3 years:28 pregnancies;<5 years:13 pregnancies;<10 years:7 pregnancies;>10 years:1pregnancy.
2	2023	Tomohiro Ochi	Japan	Retrospective cohort study	2005-2014	Median,82(± 38) months.	50(± 27) months.
3	2016	DU Yanze	China	Retrospective cohort study	1995-2004	19-120months (Median,84months).	/
4	1994	Risto Sankila	Finland	Retrospective cohort study	1967-1990	/	≥ 10 months.
5	1995	By Eva von Schoultz	Sweden	Prospective cohort study	1971-1988	1–19 years (Median,7 years).	Mean,3.0 years.
6	1996	Nicholaos A.Malamos	Greece	Case-control study	1978-1995	12-168months (Median,38 months).	7–100 months (Median,31 months).
7	1998	Niels Kroman	Denmark	Prospective cohort study	1977-1994	35067 patient-years.	Birth:11–147 months (Median,32 months); Miscarriage:6-50 Months (Median,23 months); Induced abortion:3–89months (Median,22 months).
8	1999	Priscilla Velentgas	America	Prospective cohort study	1983-1992	22-179months (Median,97 months).	Within 2 years.
9	2001	By Shari Gelber	America	Retrospective cohort study	/	Median,7.4 years.	Mean (± SD),33.0 ± 21.1 months (median, 27.8 months).
10	2003	Beth A. Mueller	America	Prospective cohort study	1980-1993/1994	With births:16–203 months (Median,105 months); without births:12–214 months (Median,99 months).	10 months or more.
11	2004	L. Johnetta Blakely	America	Prospective cohort study	1974 -1998	Median,13 years.	/
12	2017	LI Fei	China	Prospective cohort study	2005-2010	Routine, more than 5 years.	Between treatment and pregnancy:3 months:1 case;12 months: 2 cases;24–36 months:12 cases; 36–36 months: 5 cases.
13	2017	M. Al Khaduri	Oman	Retrospective cohort study	2007-2015	/	/
14	2017	Lauren Nye	America	Retrospective cohort study	2000-2010	Pregnancy:57–185 months (mean, 110 months); no pregnancy:23-168 months (mean,78 months).	Within 5 years.
15	2017	N. Sinha	America	Prospective cohort study	/	5.2 ± 2.6 years since diagnosis.	/
16	2008	Niels Kroman	Denmark	Prospective cohort study	1977-2005	95616 patient-years.	Full-term pregnancy:10–228 months (Median,39 months); Spontaneous abortion: 5–204 months (Median,26 months); Induced abortion: 3-200months (Median,23 months).
17	2009	J. Y. Wu	China	Retrospective cohort study	1989-2007	6–237 months (Median,62 months).	Between pregnancy and operation:17-84months (Median,54.5months), the earliest was 17 months after operation.
18	2010	H. M. Verkooijen	Singapore and Swedish	Prospective cohort study	1986-2006	With births:1.7–43.9 years (Median,14.3 years), a total of 8408 person-years.	At least 12 months.
19	2019	MIHONG CHOI	Korea	Retrospective cohort study	2007- 2015	/	9 months or more.
20	2019	LUO Sheng	China	Retrospective cohort study	2013-2017	Average,38.54 ± 3.67months.	/
21	2019	E. Rosenberg	Sweden	Retrospective cohort study	1982-2014	IVF group:2.6-24.0 years (mean,10.3 (SD4.2) years); Control:2.6-24.0 years (mean,10.7 (SD4.4) years).	IVF group: Mean,4.9 years; Control group: Mean,3.8 years.
22	2019	Matteo Lambertini	Belgium	Prospective cohort study	NeoALTTO trials:2008-2010; ALTTO trials: 2007-2011	6.13-6.55 years (Median,6.23 years).	/
23	2012	Octavi Córdoba	Spain	Retrospective cohort study	1995-2005	A mean of 6 years.	10–84 months (Median,44.5 months).
24	2013	Robin J. Bell	Australia	Prospective cohort study	2004-2006	At least 5 years from diagnosis.	Between 2 and 6 years.
25	2013	Adriana Valentini	Canada	Case-control study	1985-2010	Mean,10.2 years from diagnosis.	Mean time was 2.4 years.
26	2021	Yang Li	China	Retrospective cohort study	2006-2014	29–156 months (Median,101months)	/
27	2012	Hatem A. Azim Jr	Belgium	Retrospective cohort study	1977-2007	Pregnant:3.1-6.9 years (Median,4.7 years from conception); nonpregnant:2.5-7.2 years (Median,4.7 years from conception).	Median,2.4 years.
28	2022	Richard A. Anderson	England	Prospective cohort study	1981-2017	5.2-21.1 years (Median,12.2 yrs).	2.6-6.3 years (Median,4.1 years).
29	2022	Soo Youn Bae	Korea	Retrospective cohort study	2004-2014	69.2–132.5months (Median,97.9 months).	2.0-5.0 years (Median,3.3 years).
30	2022	Minsun Kang	Korea	Retrospective cohort study	2002-2017	Median,10 years from BC diagnosis until death.	Within 3 years after the BC diagnosis: Median,1 year; ≥3 years after the BC diagnosis: Median,5 years.
31	2022	J. Alejandro Rauh-Hain	America	Retrospective cohort study	2000-2012	Median,111 months in the matched pregnancy analysis.	Pregnancy group:21.8–49.2 months (Median,32.6 months); control group:21.8–49.9months (Median, 32.9 months).
32	2018	Matteo Lambertini	Belgium	Case-control study	1977-2007	Median,9.6 years from BC diagnosis (7.2 years after pregnancy, interquartile range 4.8 to 8.7 years).	/
33	2016	Anne-Sophie Hamy	France	Retrospective case series	2005-2011	11–104 months (Median,59 months).	Between last chemotherapy and prepgnancy:3.5–54.7 months(Median,35.6 months). (HR-negative:41.0 months, HR-positive:37.0 months).
34	1990	REGINA SUTTON	America	Retrospective case series	/	/	Between last chemotherapy to pregnancy:0 to 87 months.
35	1981	JAMES C. HARVEY	America	Retrospective case series	1940-1970	/	Less than 2 years after mastectomy.
36	1989	R. M. Clark	Canada	Retrospective case series	1931-1985	/	/
37	2008	T. Kojouharova	France	Prospective case series	1993-2007	Mean,105 (S.D. 43) months.	Mean,40 months (SD = 31).
38	2020	SHU-CHUN CHUANG	China	Retrospective cohort study	2002-2014	Comparison group mean:3.81 years; BCPP group mean:4.28 years.	/
39	2020	Matteo Lambertini	Italy	Retrospective cohort study	2000-2012	6.2-11.2 years (Median,8.3 years from BC diagnosis).	3.1-6.7years (Median,4.5 years).HR-positive:4.3-7.7years (Median,6.3 years); HR-negative:2.7-5.6 years (Median,4.0 yeas).
40	2020	Moo Hyun Lee	Korea	Retrospective cohort study	2002-2010	/	Median,1,153 days.
41	2015	Oranite Goldrat	Belgium	Retrospective cohort study	2000-2009	Spontaneous:9 years from BC diagnosis,5 years from conception; ART:8.5 years from BC diagnosis; 4 years from conception.	Between conception and last follow-up: spontaneous group: Mean, 63 months; ART groups: Mean,50 months.
42	1937	STUART W. HARRINGTON	America	Retrospective case series	1910-1933	Three years or more.	Between operation and livebirths: the shortest interval: eleven and one-half months; the longest interval: twelve and one-half years;22 of the 39 patients having livebirths: between one and three years.
43	1996	Anne E. LETHABY	New Zealand	Retrospective case series	1976-1985	Median,10.2 years.	5 months-7 years 5 months (Median,2 years 3 months).
44	2022	Angelena Crown	America	Retrospective cohort study	2009-2015	4–127 months (Median,70 months).	/
45	2021	Mary Kathryn Abel	America	Prospective cohort study	2010-2019	/	An average of 3.7 ± 2.1 years.
46	2023	Ann H. Partridge	America	Prospective clinical trial	2014-2019	Median,41 months (3.4 years),1638 patient-years.	/
47	2023	Hideyuki Iwahata	Japan	Retrospective case series	2013-2022	/	/
48	2024	Hatem A. Azim Jr	Belgium	Prospective clinical trial	2014-2019	Median,41 months.	/
49	2024	Matteo Lambertini	Italy	Retrospective cohort study	2000-2020	6.4-13.4 years (Median,9.1 years).	ART group: Median 4.2 years. Spontaneous group:Median3.3 years.
50	2024	Matteo Lambertini	Italy	Retrospective cohort study	2000-2020	4.5-12.6 years (Median,7.8 years).	2.2-5.3years (Median,3.5 years). HR–positive:2.8-6.3years (Median,4.3years); HR–negative:2.0-4.8 years (Median,3.2years).
51	2024	Young-jin Lee	Korea	Retrospective cohort study	2010-2020	/	/
52	2024	Ji Hye Kim	Korea	Retrospective cohort study	2009-2020	Mean, 58.9 (± 33.5) months after surgery. Mean, 43.2 (± 24.1) months post-pregnancy.	The average time was 34.6 (± 17.6) months from treatment-termination.

**Table 2:** Cancer outcomes of included articles.

No.	First-Author	Pregnant/nonpregnant BC patients	Cancer outcomes	Cancer outcomes in subgroups
1	Pentti M. Rissanen[50]	53/0	Pregnancy: ①5-year survival rate:41/53,77.4%; ②10-year survival rate:32/46,69.5%; ③5-year death rate:12/53; ④10-year death rate:14/46	1、;Pregnancy(53):(1)Stage I: interval between radiotherapy and pregnancy①5-year survival rate:<1 year:8/10;<3 years: 15/15;<5 years:2/2;<10 years:1/1;②10-year survival rate: <1 year:7/8;<3 years: 11/11;<5 years:2/2;<10 years:1/1;(2)Stage II and Stage III: interval between radiotherapy and pregnancy①5-year survival rate: <1 year:6/10;<3 years:5/10;<5 years:1/4;<10 years:1/1;②10-year survival rate:<1 year:5/9;<3 years: 3/8;<5 years:0/1;<10 years:1/1.
2	Tomohiro Ochi	childbirth/non-childbirth:104/2250	Childbirth VS Non-childbirth cohort: ①Relapse-free survival: HR = 0.469(0.221–0.992), p=0.047, relapse:11/10VS 284/2250, local relapse:2/104 VS 92/2250, distant relapse:7/104 VS 179/2250, contralateral BC:2/104 VS13/2250; ②Overall survival: HR = 0.208(0.029–1.494), p=0.119, death: 1/104 VS 92/2250	1、;Childbirth cohort(104):received ART VS did not receive ART①relapse:1/35 VS 6/69,localrelapse:0/35 VS 2/69,distant relapse:1/35 VS 2/69,contralateral BC:0/35 VS 2/69;②death:1/35 VS 0/69;2、;Childbirth cohort and ER positive(79):interruption of adjuvant hormonal therapy VS scheduled hormonal therapy: ①relapse:3/26 VS 1/53, local relapse:1/26 VS 1/53, distant relapse:2/26 VS 0/53
3	DU Yanze	68/173	Pregnancy VS no pregnancy: ①5year disease-free survival rate:60.29% (41/68) VS 34.68% (60/173), P = 0.004; ②5year overall survival rate:72.06% (49/68) VS 52.60% (91/173), P = 0.002; ③Recurrence rate:11.76% (8/68) VS17.91% (31/173), P<0.001; ④Mortality rate:27.94% (19/68) VS47.40% (82/173), P = 0.003	/
4	Risto Sankila	91/471	1、;No delivered VS delivered:①Mortality rate145/471 VS 8/91,RR=4.8(2.2-10.3);2、;Delivered VS no delivered:①5-year survival rate:92% (85-99) VS 69% (64-73); ②10-year survival rate:92% (85-99) VS 60% (54-66); ③15-year survival rate:79% (60-97) VS 55% (47-62)	1、;Mortality rate: No delivered VS delivered(471/91):(1)Tumor stage ①localized disease: RR = 6.0 (1.9-19.1);②nonlocalized disease: RR = 6.5(1.6-26.8);③unknown stage: RR = 1.2(0.3-6.0);(2)Interval from diagnosis to delivery①10-24months:RR=11.3(1.6-82.8);②25-60months:RR=2.6(1.1-6.0);③61-154months:RR=NA;2、;Mortality rate: children before diagnosis:①NO: RR = 4.9;②YES VSNO: RR = 3.9(2.5-6.0);③0:RR=4.9;④1 VS 0:RR=3.6(2.1-6.1);⑤2 VS 0:RR=3.9(2.3-6.5);⑥3–7 VS 0:RR=4.2(2.3-7.7);3、;5-year cumulative survival rates: delivered VS No delivered(91/471):(1)Tumor stage:①localized disease:96% (90-100)VS 77%(72-83)② nonlocalized disease:79%(52-100) VS 49%(39-59)③unknown stage:75%(32-100)VS63%(41-84);(2)①10–24 months: localized disease(91%VS66%); nonlocalized disease (100%VS38%);②25-60months:localized disease(95%VS78%); nonlocalized disease (67%VS48%)③61-154months:localized disease(100%VS88%);nonlocalized disease (100%VS69%);4、;10-year cumulative survival rates: delivered VS No delivered (91/471): (1) Tumor stage: ①localized disease:96%(90-100) VS67%(61-74)② nonlocalized disease:79%(52-100)VS42%(29-54)③unknown stage:75%(32-100)VS54%(30-78)(2) ①10-24months:localized disease(91%VS57%);nonlocalized disease (100%VS38%)②25–60 months: localized disease(95%VS71%);nonlocalized disease (67%VS38%);③61-154months:localized disease(100%VS71%); nonlocalized disease(-%VS-%);5、;15-year cumulative survival rates: delivered VS No delivered (91/471):(1) Tumor stage: ①localized disease:84% (63-100) VS60% (51-71) ②nonlocalized disease:79% (52-100) VS42% (29-54) ③ unknown stage:38% (0-95) VS54% (30-78)
5	By Eva von Schoultz	50/2069	Pregnancy VS no pregnancy: ①Distant metastasis:4/50 VS 489/2069, HR = 0.48(0.18-1.29), P = 0.14	/
6	NicholaosA. Malamos	21/222	Pregnancy VS no pregnancy: ①Recurrence rate:3/21 VS 87/222, P<0.05; ②Survival: the median one:87months VS 48months(P = 0.00057)	1、;Recurrence rate: pregnancy VS no pregnancy (21/222): ①StageI:0/9 VS 10/39, P<0.1; ②StageII:2/10 VS 44/116, P=NS; ③StageIII:1/2 VS 33/63, P=NS; ④StageIV:0/0 VS 0/4, P=NS
7	Niels Kroman	173/5552	Risk of death: ①full-term pregnancy VS without full-term pregnancy: RR = 0.55(0.28-1.06), P = 0.08; ② Induced abortion VS without induced abortion: RR = 1.0(0.67-1.50); ③ miscarriage VS without miscarriage: RR = 0.36(0.09-1.45)	1、;Risk of death: Reproductive status: ①full-term pregnancy VS without full-term pregnancy: RR = 0.55(0.28-1.06), P = 0.08; ②induced abortion VS without induced abortion: RR = 1.0(0.67-1.50); ③miscarriage VS without miscarriage: RR = 0.36(0.09-1.45); 2、;Risk of death:①Having a low-risk tumor (n=2110): full-term pregnancy VS without full-term pregnancy: RR = 0.61(0.19-1.91)
8	Priscilla Velentgas	53/265	Pregnancy VS no pregnancy: ①Risk of Mortality:5/53 VS 34/265, RR = 0.8(0.3-2.3)	1、;Risk of Mortality:①with any live births VS without subsequent pregnancies:5/36 VS 26/163,RR=1.1(0.4-3.7);②spontaneous and induced abortions VS no pregnancy:0/17 VS 4/85,RR=0(0.0-7.8);2、;Risk of Mortality: pregnancy VS no pregnancy (53/265): ①Local disease at diagnosis:1/36 VS 14/180, RR = 0.2(0.02-2.4); ②regional disease at diagnosis:4/17 VS20/85, RR = 1.4(0.4-5.2)
9	By Shari Gelber	94/188	Pregnancy VS no pregnancy: ①5-year survival rate:92% (± 3%) VS 85% (± 3%); ②10-year survival rate:86% (± 4%) VS 74% (± 4%); ③Overall survival:11/94 VS 35/188, RR = 0.44(0.21-0.96), P = 0.04	1、;10-year survival rate: pregnancy VS no pregnancy (94/188): ①women who had at least one full-term pregnancy (73):85% (± 5%) VS 79% (± 4%); ② women who had less than a full-term pregnancy (21):91% (± 9%) VS 51% (± 10%)
10	Beth A. Mueller	live birth/no live birth:328/2002	With births occurring VS without births occurring: ①Risk of Mortality:62/328 VS 532/2002, RR = 0.54(0.41-0.71)	Risk of Mortality: with births occurring VS without births occurring:1、;①local disease at diagnosis:28/208 VS 231/1278,RR=0.59(0.40– 0.89);②regional disease at diagnosis:31/106 VS 288/697,RR=0.54(0.37– 0.78);2、;①lymph node positive:25/77VS 220/550,RR=0.65(0.42– 0.99); ②lymph node negative:16/136 VS 152/901,RR=0.56(0.33– 0.95);3、;①tumor size less than 2 cm:11/76 VS 86/481,RR=0.70(0.36– 1.4);②tumor size 2 cm or larger:28/127 VS 267/846,RR=0.52(0.35– 0.77);4、; ①had received chemotherapy:21/85 VS 203/652,RR=0.54(0.34– 0.86);②had not received chemotherapy:26/154 VS 197/829,RR=0.56(0.37– 0.86);5、;① had radiotherapy:14/93 VS 143/569,RR=0.44(0.25– 0.77);② had not radiotherapy:33/152 VS 266/947,RR=0.59(0.41– 0.86);6、;① had received other treatment modalities such as hormonal or immunotherapy:6/24 VS 83/220,RR=0.36(0.15– 0.88);② had not received other treatment modalities such as hormonal or immunotherapy:42/227 VS 332/1335,RR=0.57(0.41– 0.79);7、;elapsed time intervals in years since diagnosis:① 2 to less than 3 years: RR = 0.49(0.27– 0.86); ②3 to less than 4 years: RR = 0.30(0.12– 0.71); ③4 to less than 5 years: RR = 0.19(0.05– 0.81)
11	L.JohnettaBlakely	47/323	Pregnancy VS no pregnancy: ①Recurrence rate:10/47(23%) VS 174/323(54%); ②Relapse-free survival: 2-year RFS (%):94 ± 4VS69 ± 3;5-year RFS (%):82 ± 6VS49 ± 3, HR = 0.70 (0.25-1.95), P = 0.49.	/
12	LI Fei	20/20	Pregnancy VS no pregnancy: ①Recurrence rate:2/20 VS 4/20, P = 0.027; ②Distant metastasis rate:1/20 VS 4/20, P = 0.017; ③Mortality rate:1/20 VS 2/20, P = 0.624; ④5-year disease -free survival rate:16/20 VS 10/20, P = 0.011; ⑤5-year overall survival rate:19/20 VS 18/20, P = 0.357	/
13	M. Al Khaduri	14/91	/	1、;Recurrence rate: pregnancy VS no pregnancy (14/91): ①pregnancy was within two years of BC diagnosis: RR = 0.42(0.05-2.97); ②beyond two years: RR = 0.21(0.01- 3.78)
14	Lauren Nye	ER-positive:32/29	①Recurrence rate: no pregnancy VS pregnancy:4/29 VS 8/32, P = 0.34; ②5-year disease-free survival rate: pregnancy VS no pregnancy:84% (72-97%) VS 92% (81-100%), P = 0.69.	/
15	N. Sinha	HR-positive and interrupted treatment:16/29.	Pregnancy VS pregnancy: ①Recurrence rate:0/16 VS 0/29	/
16	Niels Kroman	371/9865	Risk of death: ①full-term pregnancy VS without full-term pregnancy: RR = 0.73 (0.54-0.99), P = 0.04; ②induced abortion VS without induced abortion: RR = 0.84 (0.64-1.11); ③ spontaneous abortion VS without spontaneous abortion: RR = 0.35 (0.15-0.85)	1、;Risk of death: Reproductive status:①full-term pregnancy VS without full-term pregnancy: RR = 0.73 (0.54–0.99),P=0.04;②induced abortion VS without induced abortion: RR = 0.84 (0.64–1.11);③spontaneous abortion VS without spontaneous abortion: RR = 0.35 (0.15–0.85);2、;Risk of death: having a low-risk tumor (n= 2 901) full-term pregnancy VS without full-term pregnancy: RR = 0.56(0.29-1.11)
17	J. Y. Wu	18/414	Pregnancy VS no pregnancy: ①Disease-free survival:88.9% (2/18) VS 71.7% (117/414), P = 0.044; HR = 0.174(0.039-0.771), P = 0.021; ②Overall survival:100% (0/18) VS 88.2% (49/414), P = 0.076;	/
18	H. M. Verkooijen	childbirth/non-childbirth:492/8529	Subsequent childbirth/Without subsequent childbirth: ①5-year cumulative overall mortality rate:3.0 (1.8 -5.0) VS 23.4(22.3-24.4); ②10-year cumulative overall mortality rate: 9.3 (7.1-12.4) VS 34.5(33.4- 35.6); ③ 15-year cumulative overall mortality rate:16.8 (13.3-20.9) VS 40.7(39.5-41.9); ④mortality rate:68/492 VS 2661/8529	1、;Standardized mortality ratios(SMRs):BC women with subsequent childbirth VS young women in the general population:①SMR=13.6(10.6 to 17.3);②Interval between BC diagnosis and subsequent childbirth: 12–24 months: SMR = 22.6 (14.3, 33.9);③24.1–48 months: SMR = 14.7 (10.1, 20.7);④more than 48 months: SMR = 6.9 (3.6, 12.1);⑤Time since BC diagnosis(years) 0-2:SMR=1.9 (0.1, 11.0);⑥2-5:SMR=15.6 (8.5, 26.2);⑦ 5-7:SMR=17·6 (9.1, 30.8);⑧7-10:SMR=15.0 (8.6, 24.3);⑨ 10-12:SMR=14.8 (7.4, 26.6);⑩ 12-15:SMR=12.7 (7.0, 21.3);2、;SMRs: BC women without subsequent childbirth VS young women in the general population:①SMR=33.7(32.4 to35.0);②0-2:SMR=64.0 (59.1, 69.1);③2-5:SMR=69.2 (65.1, 73.6); ④ 5-7:SMR=34.2 (30.7, 38.0);⑤ 7-10:SMR=20.8 (18.6, 23.2);⑥10-12:SMR=13.4 (11.3, 15.7); ⑦ 12-15:SMR=8.8 (7.5, 10.4);3、;Mortality ratios: with subsequent childbirth VS without subsequent childbirth: Time since BC diagnosis(years):①0-2:1/492 VS 646/8529;②2-5:14/492 VS 1045/8529;③5-7:12/492 VS 346/8529;④ 7-10:16/492 VS 326/8529; ⑤ 10-12:11/492 VS 146/8529; ⑥ 12-15:14/492 VS 152/8529;
19	MIHONGCHOI	57/3630	Pregnancy VS no pregnancy: ①5-year survival rates:98.11% (87.35–99.73) VS 95.71% (94.96–96.36); p = 0.6881; ② Overall survival: HR = 0.86 (0.26-2.83), p = 0.8004	/
20	LUO Sheng	11/19	Pregnancy VS no pregnancy: ①Recurrence:2/11 VS 7/19,18.18% VS 36.84%, P <0.05; ②Disease-free survival:10/11 VS 13/19,90.91% VS 68.42%, P <0.05; ③Overall survival:11/11 VS16/19,100.00% VS 84.21%, P <0.05	/
21	E. Rosenberg	IVF conception/spontaneous conception:37/148	/	1、;Risk of relapse:①IVF conception VS spontaneous conception:0/37 VS 36/148, P = 0.0002② IVF after completed BC treatment VS spontaneous conception:0/29 VS 36/148; ③fertility preservation at the time of diagnosis VS spontaneous conception:0/8 VS 36/148;
22	Matteo Lambertini	HER2-positive:85/1307	Pregnancy VS no pregnancy: ①Disease-free survival:9/85 VS 254/1307, HR = 1.12(0.52-2.42); ②Overall survival:2/85 VS 130/1307	/
23	Octavi Córdoba	18/97	Pregnancy VS no pregnancy: ①5-year survival rate:100% VS 80%; ②5-year disease-free survival rate:94% VS 64%(P = 0.009).	/
24	Robin J. Bell	9/37	Pregnancy VS no pregnancy: ①Recurrence:2/9 VS 6/37; ②Overall survival:0/9 VS 1/37	/
25	Adriana Valentini	BRCA1 or BRCA2 mutation:53/269	Pregnancy VS no pregnancy: ①15-year survival rates:93.6% VS 88.6%; ②BC-specific survival:1/53 VS 12/269; ③BC-specific mortality at 15 years: HR = 0.73(0.21-2.68), p = 0.64	/
26	Yang Li	68/264	Pregnancy VS no pregnancy: ①Recurrence:3/68 VS 32/264; ②Death:0/68 VS 7/264; ③Disease-free survival: median DFS time:102.5months(33-156months) VS 94months(17-155months), P = 0.058; ④Overall survival: OS time:106 months(50-156months) VS 100months(29-155months), P = 0.152.	1、;Disease-free survival: pregnancy VS no pregnancy:(1)①HR-positive: P = 0.657;②HR-negative: P = 0.026;(2)①BC pregnancy interval≤ 5 years(30):P=0.042,HR=0.08(0.022 -1.237),P=0.080;2、;Overall survival: pregnancy VS no pregnancy:①HR-positive: P = 0.250;②HR-negative: P = 0.389;3、;Disease-free survival: pregnancy group and received endocrine therapy (ET) (43): ET interval ≤ 30 months VS ET interval>30 months:2/11vs1/32, P = 0.01
27	Hatem A. Azim Jr	333/874	Pregnancy VS no pregnancy: ①Disease-free survival: HR = 0.84(0.66-1.06), p = 0.14; ②Overall survival: HR = 0.72(0.54-0.97), p = 0.03.	1、;Disease-free survival: pregnancy VS no pregnancy:(1)①ER–positive: HR = 0.91(0.67–1.24),P = 0.55;② ER–negative: HR = 0.75(0.51–1.08),P = 0.12;(2)①Completed: HR = 0.79(0.57-1.08), P = 0.14;② Abortion: HR = 0.87(0.58-1.31), P = 0.5;(3)Time to Pregnancy After BC diagnosis:①<2 years: HR = 0.56(0.34-0.92),P =0.02;②≥2years:HR=1.13(0.64-1.98),P =0.68;(4)①<2 years, ER–positive: HR = 0.72;②<2 years, ER–negative: HR = 0.58;(5)①no pregnancy:≥2years VS <2years:HR= 2.2(1.7 -2.8), P <0.001;②pregnancy:≥2years VS <2years:HR=1.1(0.78-1.7),P =0.45;2、;Overall survival: pregnancy VS no pregnancy: ①ER–positive: HR = 0.89(0.61–1.29), P = 0.52; ② ER–negative: HR = 0.54(0.33–0.87), P = 0.01;
28	Richard A. Anderson	live birth/no live birth:290/1682	live birth VS no live birth: ①20-year overall survival rate:73.9% (67.7%-80.7%) VS 64.6% (61.8%-67.5%); ②Overall survival: HR = 0.65 (0.50-0.85), P = 0.002.	1、;Overall survival: live birth VS no live birth:①interval between diagnosis and live birth<5years:20-year overall survival rate:71.9%(64.6%-80.0%)VS 60.6%(57.3%-64.1%),HR=0.66(0.49-0.89),p = 0.006;② interval between diagnosis and live birth≥5years:15-year overall survival rate:80.5%(70.6%-91.8%)VS 75.8%(71.4%-80.5%),HR=0.63 (0.36-1.13), p= 0.121;③ER positive:10-year overall survival rate:84.1%(75.5%-93.7%)VS 78.9%(75.3%-82.8%),HR=0.66 (0.37-1.18), p= 0.160;④ ER negative:10-year overall survival rate:79.8%(68.9%-92.3%)VS 77.4%(73.1%-82.1%),HR=0.72 (0.38-1.35), p = 0.301;⑤ known exposure to chemotherapy:20-year overall survival rate:55.3%(43.5%-70.3%)VS 53.4%(47.9%-59.5%),HR=0.86 (0.64-1.20), p = 0.33;⑥had not had a pregnancy before BC:20-year overall survival rate:73.6%(64.4%-84.1%)VS 62.7%(58.7%-67.0%),HR=0.56(0.38-0.82),P=0.003;⑦had a pregnancy before BC:20-year overall survival rate:74.4%(66.4%-83.4%)VS 66.2%(62.4%-70.2%),HR=0.76(0.53-1.09),p=0.134;⑧women with one subsequent live birth:20-year overall survival rate:72.5%(65.4%-80.3%)VS 61.6%(58.1%-65.2%),HR=0.73(0.54-0.98),p=0.033;⑨women with more than one subsequent live birth:15-year overall survival rate:81.7%(70.7%-94.3%)VS 73.8%(68.9%-79.1%),HR=0.84(0.46-1.50),p=0.57;⑩ tumor stage I:10-year overall survival rate:82.4%(73.0%-93.1%)VS 83.8%(80.4%-87.3%),HR=0.74 (0.40-1.35), p=0.328;11、;tumor stage IIorIII:10-year overall survival rate:81.5%(71.0%-93.6%)VS75.1%(70.2%-80.2%),HR=0.71 (0.37-1.37), p = 0.303;12、;ER positive, live birth with 5 years from diagnosis: HR = 0.54 (0.26-1.1), p = 0.091;13、;ER positive, live birth more than 5 years from diagnosis: HR = 0.79 (0.31-2.0), p= 0.629
29	Soo YounBae	1826/43939	Pregnancy VS no pregnancy ①Risk of mortality: HR = 0.43(0.35-0.53), p < 0.001	1、;Risk of mortality: Time to Pregnancy After BC diagnosis: pregnancy:①≥49months VS ≤12 months: HR = 0.15(0.06-0.36),p<0.001;②25–48 months VS ≤12 months: HR = 0.64(0.31-1.32),P=0.225;③13–24 months VS ≤ 12 months: HR = 1.06(0.50-2.25),P=0.873; 2、;Risk of mortality:① no treatment: live birth VS no pregnancy: HR = 0.43(0.15-1.27), p =0.127;Failure to birth VS no pregnancy: HR = 1.10 (0.38-3.22), p =0.866;②endocrine therapy-only: live birth VS no pregnancy: HR = 0.24 (0.05-1.22),P=0.085; Failure to birth VS no pregnancy: HR = 0.76 (0.22- 2.67), p =0.674;③ chemotherapy-only, live birth VS no pregnancy: HR = 0.23(0.15-0.36), p < 0.001; Failure to birth VS no pregnancy: HR = 0.45 (0.28-0.72), p < 0.001;④ endocrine therapy and chemotherapy, live birth VS no pregnancy: HR = 0.31(0.18 -0.56), p < 0.001;Failure to birth VS no pregnancy: HR = 0.94 (0.64-1.39), p= 0.762;⑤ chemotherapy and trastuzumab, live birth VS no pregnancy: HR = 0.23 (0.05-1.13),P=0.071; Failure to birth VS no pregnancy: HR = 0.80 (0.31-2.03), p= 0.630;⑥endocrine therapy, chemotherapy, and trastuzumab: live birth VS no pregnancy: HR = 0.24 (0.02- 3.90),P=0.316; Failure to birth VS no pregnancy: HR = 0.24 (0.01-3.82), p= 0.308;⑦The endocrine therapy group: live birth VS no pregnancy: HR = 0.25 (0.14-0.43),p < 0.001; Failure to birth VS no pregnancy: HR = 0.82 (0.56-1.20), p= 0.299;⑧ The non-endocrine therapy group: live birth VS no pregnancy: HR = 0.22 (0.14-0.33),p < 0.001; Failure to birth VS no pregnancy: HR = 0.49 (0.33-0.73), p < 0.001;3、;Risk of mortality:live birth VS no pregnancy: HR = 0.27 (0.20-0.38),p<0.001.
30	Minsun Kang	272/272	Pregnancy VS no pregnancy: ①10-year overall survival rate:97.4% VS 91.9%; ②Overall survival: HR = 0.29(0.12–0.68), P = 0.005; ③Disease-free survival:8 years VS 8years,25/272 VS 20/272, HR = 1.10(0.61–1.99), P = 0.760.	1、;Overall survival: pregnancy:①Pregnant <3 yr VS ≥3 yr from diagnosis: HR = 2.32 (0.43–12.59) P = 0.329;2、;Overall survival: Pregnant vs. nonpregnant:①Completed: HR = 0.22 (0.08–0.57)P= 0.002;② Miscarriage: HR = 1.98 (0.18–21.83) P = 0.578;3、;Overall survival:①Type of pregnancy among starting before 3 yr from diagnosis: Completed (<3 yr vs. ≥3 yr):HR=1.53 (0.24–9.90) P = 0.657;4、;Overall survival: Pregnant vs. nonpregnant:①Hormone therapy (HR)-Yes: HR = 0.17 (0.04–0.80) P = 0.025;② Hormone therapy (HR)-NO: HR = 0.37 (0.13–1.03) P = 0.057;③Target therapy (HER2)-Yes: HR = 0.23 (0.03–2.05)P= 0.187;④Target therapy (HER2)-NO: HR = 0.32 (0.13–0.80) P = 0.015;⑤Chemotherapy-Yes: HR = 0.42 (0.17–1.03)P= 0.057; ⑥ Chemotherapy-NO: ND;⑦Combination of clinical treatment options:(1)HR+, HER2+, Chemo+:ND;(2)HR+, HER2-, Chemo+:HR=0.35 (0.07–1.82) P = 0.212; (3)HR+, HER2-, Chemo-:ND;(4) HR-, HER2+, Chemo+:HR=1.62 (0.08–32.48) P = 0.753;(5) HR-, HER2-, Chemo+:HR=0.64 (0.18–2.25)P= 0.482;(6) HR-, HER2-, Chemo-:ND;5、;Disease-free survival:①Pregnant <3 yr: Pregnant vs. nonpregnant: HR = 0.99(0.50–1.94),P = 0.974;②Pregnant≥3 yr from diagnosis: Pregnant vs. nonpregnant: HR = 1.48(0.42–5.24), P = 0.546;③Pregnant <3 yr VS ≥3 yr from diagnosis: HR = 2.46 (0.95–6.38) P = 0.064;6、;Disease-free survival: Pregnant vs. nonpregnant:①Completed: HR = 0.94 (0.51–1.74)P= 0.852;② Miscarriage: ND;7、;Disease-free survival: Type of pregnancy among starting before 3 yr from diagnosis:①Completed (<3 yr vs. ≥3 yr):HR=2.80 (0.99–7.89) P = 0.052;②Miscarriage (<3 yr vs. ≥3 yr):HR=2.69 (0.07–104.16) P = 0.596;8、;Disease-free survival: Pregnant vs. nonpregnant:①Hormone therapy (HR)-Yes: HR = 0.88 (0.33–2.35) P = 0.793;② Hormone therapy (HR)-NO: HR = 1.24 (0.59–2.63) P = 0.572;③Target therapy (HER2)-Yes: HR = 0.22 (0.02–2.34)P= 0.211;④Target therapy (HER2)-NO: HR = 1.18 (0.62–2.25) P = 0.604;⑤Chemotherapy-Yes: HR = 0.82 (0.36–1.84)P= 0.623;⑥ Chemotherapy-NO: HR = 1.46 (0.60–3.58)P=0.407;⑦Combination of clinical treatment options:(1)HR+, HER2+, Chemo+:ND;(2)HR+, HER2-, Chemo+:HR=0.43 (0.12–1.72) P = 0.230;(3) HR+, HER2-, Chemo-:HR=-5.38 (0.58–50.16)P=0.140;(4)HR-, HER2+, Chemo+:HR=0.23 (0.01–3.63) P = 0.295;(5) HR-, HER2-, Chemo+:HR=1.87 (0.47–7.47)P= 0.379;(6) HR-, HER2-, Chemo-:HR-1.10 (0.40–3.04) P = 0.851
31	J. Alejandro Rauh-Hain	520/520	Pregnancy VS no pregnancy: ①BC disease-specific survival:23/520 VS 48/520, HR = 0.43(0.24–0.77); ②5-year disease-specific survival rate:95.6% VS 90.6%; ③Overall survival: HR = 0.47(0.27–0.79)	1、;BC disease-specific survival: Pregnant vs. nonpregnant: ①HR positive: HR = 0.43(0.2–0.91); ② HR negative: triple negative BC: HR = 0.3(0.08–1.11); ③achieved a pregnancy within 1–2 years: HR = 0.83(0.36–1.93); ④ beyond 2 years from diagnosis: HR = 0.32(0.16–0.66); ⑤stage I:HR=0.31(0.06–1.55);
32	Matteo Lambertini	333/874	Pregnancy VS no pregnancy: ①Disease-free survival: HR = 0.85(0.68-1.06), p = 0.15; ②Overall survival: HR = 0.72(0.55-0.94), p = 0.02.	1、;Disease-free survival: Pregnant vs. nonpregnant:(1)①Completed: HR = 0.85(0.63-1.14), P = 0.27;② Abortion: HR = 0.8(0.56- 1.13),P=0.20;(2)①ER positive: HR = 0.94(0.70-1.26),P=0.68;② ER negative: HR = 0.75(0.53-1.06),P=0.1;(3)①<2 y from diagnosis: HR = 0.65(0.47 - 0.90),P =0.008);②≥2 y from diagnosis: HR = 1.12(0.82-1.54), P = 0.47;(4)①patients who breastfed their newborns: HR = 0.70(0.26 - 1.94), P = 0.50;②patients who did not breastfed their newborns: HR = 1.44(0.77-2.69), P = 0.25;2、;Overall survival: Pregnant vs. nonpregnant:(1)①ER positive: HR = 0.84(0.60- 1.18), P = 0.32; ②ER negative: HR = 0.57(0.36-0.90), P = 0.01;3、;Disease-free survival: Pregnant and ER positive: Prior chemotherapy VS No chemotherapy:48/141 VS 14/53,HR=1.28 (0.71- 2.33),P=0.41;4、;Disease-free survival: Pregnant and ER positive: Duration of endocrine therapy:> 5 years of endocrine therapy VS ≤ 5 years of endocrine therapy:7/33 VS 5/24, HR = 0.98 (0.31-3.10), P = 0.98
33	Anne-Sophie Hamy	17/117	Pregnancy VS no pregnancy ①Recurrence:2/17 VS 46/117	/
34	REGINA SUTTON	25/202	Pregnancy: ①Recurrence:7/25; ②Mortality:3/25	1、;Recurrence: pregnancy: ①ER positive:2/4; ②ER negative:5/21
35	JAMES C. HARVEY	22/19	/	1、;10-year survival rate without evidence of disease: pregnancy ①axillary nodes negative for tumor:69%;② axillary nodes positive for tumor:71%;2、;Mortality: pregnancy: ①axillary nodes negative for tumor:4/14; ② axillary nodes positive for tumor:?/8
36	R. M. Clark	136/0	Pregnancy: ①5-year survival rate:78%; ②10-year survival rate:64%; ③5-year Relapse-free rates:60%; ④10-year Relapse-free rates:54%	1、;5-year survival rate: Pregnant:①multiple pregnancies:97%;② single pregnancies:73%; ③Interval from initial treatment to subsequent pregnancy:<6months:59%; ④ 6-23months:75%;⑥ 24-59months:92%;⑦continuing to a live birth:87%;⑧ therapeutic abortion:58%;2、;10year-survival rate: Pregnant:①negative nodes:76%;②positive nodes:63%;③continuing to a live birth:72%;④ therapeutic abortion:43%;3、;Survival rate: Pregnant:①<6 vs 12-23months:P = 0.032;②<6 vs 24- 59months:P<0.0005;③ <6 vs ≥60months:P<0.0005;④ 6–11 vs ≥60months:P=0.023;⑤12–23 vs ≥60months:P=0.03;4、;Survival rate: Pregnant: ①Live Birth vs, Spontaneous Abortion: P = 0 055; ②Live Birth vs Therapeutic Abortion: P <0.0005
37	T. Kojouharova	20/0	Pregnancy: ①Overall survival:90%; ②Recurrence:5/20Local recurrence:1/20; New primary cancer:1/20; Distant metastasis:3/20; ③Mortality:2/20	1、;Recurrence: pregnancy: Interval from diagnosis to subsequent pregnancy:①<2years:1/6;② ≥2years:4/14;2、;Mortality: pregnancy:①<2years:1/6; ② ≥2years:1/14
38	SHU- CHUN CHUANG	249/914	Pregnancy VS pregnancy①Mortality:12/249 VS 69/914; HR = 0.44(0.23 - 0.84); P = 0.06	1、;Mortality: pregnancy VS no pregnancy:(1)①ER-positive: HR = 0.23(0.07-0.77),P=0.03,3/87 VS 32/311;②ER-negative: HR = 0.38 (0.12-1.24),P=0.26,4/46 VS 20/143;(2)①<1 year from diagnosis: HR = 0.84 (0.25- 2.81),4/28 VS 69/914;②≥1 to <2 years: HR = 0.70 (0.24-2.02),4/40 VS69/914;③≥2 to <3 years: HR = 0.40 (0.10-1.63),2/63 VS69/914;④≥3 years: HR = 0.19(0.05-0.78),2/118 VS69/914;(3)By hormone therapy:①YES: HR = 0.46 (0.15-1.38),3/111 VS 29/535;②NO: HR = 0.40 (0.17- 0.91),9/138 VS 40/379;(4)By chemotherapy:①YES: HR = 0.40 (0.18-0.90),7/148 VS 53/670;②NO: HR = 0.48 (0.15-1.56),5/101 VS 16/244;(5)By stage at diagnosis:① I:HR=0.39 (0.11-1.41),4/126 VS 16/438;②II and III: HR = 0.37 (0.16-0.82),8/123 VS 53/476; (6)By positive nodes ①0:HR=0.62 (0.25-1.52),6/173 VS 32/657;②1–3:HR=0.68 (0.17-2.66),3/34 VS 14/146;③≥4:HR=0.31 (0.07-1.47),2/20 VS 20/65;(7)①Completed pregnancy: HR = 0.40 (0.20-0.82),9/219 VS 69/914;②Spontaneous or induced abortion: HR = 0.73 (0.17-3.03),3/30 VS 69/914;
39	Matteo Lambertini	Germline BRCA mutations:195/1057	Pregnancy VS pregnancy: ①Recurrence rate: Loco-regional recurrence: 16/62 VS 75/425; Distant recurrence:11/62 VS 139/425; Second primary malignancy, except BC:5/62 VS 40/425; Second primary BC:30/62 VS 162/425②Disease-free survival:62/195 VS 425/1057, HR = 0.87(0.61 -1.23); P = 0.41; ③Overall survival:14/195 VS 158/1057HR=0.88(0.50 -1.56); P = 0.66	1、;Disease-free survival: pregnancy VS no pregnancy:(1) ①BRCA1:HR=0.64 (0.41-0.99) ②BRCA2: HR = 1.94 (1.10 - 3.45); (2) ①HR-positive: HR = 1.46 (0.84- 2.55); ② HR-negative: HR=0.67 (0.42 -1.04)
40	Moo Hyun Lee	992/30769	Pregnancy VS pregnancy: ①Recurrence rate:138/992 VS 7559/30769, HR = 0.450(0.380-0.533), P< 0.001; ②Mortality:44/992 VS 2256/30769, HR = 0.486(0.360-0.655), P< 0.001; ③5-year Relapse-free rates:90.27% VS 79.60%; ④5-year survival rate:96.32% VS 92.37%	1、;Recurrence rate:①Successfully delivered VS no pregnancy:60/622 VS 7559/30769,HR=0.301(0.233-0.387),P< 0.001;②Failed to deliver VS no pregnancy:78/370 VS 7559/30769,HR=0.730(0.584-0.912), P = 0.006;③Successfully delivered VS Failed to deliver:60/622 VS 78/370,HR=0.410(0.293-0.514),P<0.001;2、;Mortality: ①Successfully delivered VS no pregnancy:20/622 VS 2,256/30769,HR=0.345(0.222-0.535),P< 0.001;②Failed to deliver VS no pregnancy:24/370 VS 2,256/30769,HR=0.741(0.496-1.108), P = 0.144;③Successfully delivered VS Failed to deliver:20/622 VS 24/370,HR=0.461(0.255-0.834),P=0.011;3、;5-year recurrence rate:①Successfully delivered VS no pregnancy:93.58% VS 79.60%;②Failed to deliver VS no pregnancy:84.61% VS 79.60%;③Successfully delivered VS Failed to deliver:93.58% VS84.61%;4、;5-year survival rate:①Successfully delivered VS no pregnancy:97.38% VS 92.37%; ② Failed to deliver VS no pregnancy:94.44% VS 92.37%; ③Successfully delivered VS Failed to deliver:97.38% VS 94.44%
41	Oranite Goldrat	198/0	/	1、;Recurrence rate: spontaneous VS ART groups:28/173 VS 2/25,Local recurrence:8/173 VS 0/25,Distant recurrence:10/173 VS 2/25,Contralateral BC:7/173 VS 0/25,2nd primary cancer (non-breast):3/173 VS 0/25;2、;Mortality: spontaneous VS ART groups:11/173 VS 1/25
42	STUART W. HARRINGTON	55/0	Pregnancy ①3-year survival rate:52/55,94.5%; ②5-year survival rate:42/53,79.2%; ③10-year survival rate:28/37,75.7%; ④15-year survival rate:15/21,71.4%;	1、;3-year survival rate:①with axillary metastases:22/25 88.0%;②without axillary metastases:30/30,100.0%; 2、;5-year survival rate: ①with axillary metastases:13/23, 56.5%;②without axillary metastases:29/30, 96.7%;3、;10-year survival rate:①with axillary metastases:6/12,50.0%;②without axillary metastases:22/25, 88.0%;4、;15-year survival rate:①with axillary metastases:3/5, 60.0%;②without axillary metastases:12/16,75.0%;5、;Survival rate: Intervals after First Birth Subsequent to Operation:(1)I Yr. or More:①Total:41/45②with axillary metastases:17/21③without axillary metastases:24/24(2) 2 or More①Total:34/44②with axillary metastases:12/20③without axillary metastases: 22/24(3) 3 or More①Total:30/41②with axillary metastases:8/17③without axillary metastases: 22/24(4)5 or More①Total:28/37②with axillary metastases:8/14③without axillary metastases:20/23 (5)10 or More①Total:14/22②with axillary metastases:2/4③without axillary metastases: 12/18(6) 15 or More①Total:8/14②with axillary metastases:1/4③without axillary metastases:7/10;6、;Survival rate: delivered at full term: Intervals after First Birth Subsequent to Operation:(1)I Yr. or More:①Total:33/37②with axillary metastases:12/16③without axillary metastases:21/21(2) 2 or More①Total:29/37②with axillary metastases:10/16③without axillary metastases: 19/21(3) 3 or More①Total:26/35②with axillary metastases:7/14③without axillary metastases: 19/21(4)5 or More①Total:24/31②with axillary metastases:7/11③without axillary metastases:17/20 (5)10 or More①Total:12/18②with axillary metastases:2/3③without axillary metastases: 10/15(6) 15 or More①Total:7/12②with axillary metastases:1/3③without axillary metastases:6/9;
43	Anne E.LETHABY	14/334	Pregnancy VS pregnancy: ①Mortality:5/14 VS 153/334	1、;Mortality: pregnancy VS pregnancy: ①node-negative:2/10 VS 68/207; ②node-positive:3/4 VS 85/127
44	Angelena Crown	35/137	Pregnancy VS pregnancy: ①Mortality:0/35 VS 7/137②Recurrence rate:2/35 VS 16/137	/
45	Mary Kathryn Abel	35/11	Pregnancy VS pregnancy: ①Recurrence rate:1/35 VS 0/11	1、;Recurrence rate:pregnancy VS no pregnancy: ①spontaneous:1/22VS 0/12; ② ART groups:0/13 VS 0/11
46	Ann H. Partridge	①ER-positive, interruption of endocrine therapy to attempt pregnancy:516;②ER-positive, interruption of endocrine therapy to successfully pregnancy VS no pregnancy:368/129;③Patients with a pregnancy at or before 18 months VS patients without a pregnancy at or before 18 months:305/137;	1、;Interruption of adjuvant endocrine therapy to attempt pregnancy VS the external control cohort of patients who did not interrupt their adjuvant endocrine therapy:①BC events rate((local, regional, or distant recurrence or a new invasive contralateral BC):total:44/516 VS 168/1499,HR=0.81 (0.57-1.15);②3-year incidence of BC events:8.9% (6.3-11.6) VS 9.2% (7.6-10.8);③Distant recurrence rate:22/516 VS 118/1499,HR=0.70 (0.44-1.12);④3-year incidence of distant recurrence events:4.5% (2.7 -6.4) VS 5.8% (4.5-7.2);⑤Local recurrence rate:14/516 VS 28/1499;⑥Regional recurrence rate:6/516 VS 20/1499;⑦Contralateral BC rate:3/516 VS 8/1499;⑧Distant recurrence-Soft tissue/distant LN rate:0/516 VS 5/1499;⑨Distant recurrence-Bone rate:4/516 VS 38/1499;⑩Distant recurrence-Viscera rate:17/516 VS 69/1499;2、;Patients with a pregnancy at or before 18 months VS patients without a pregnancy at or before 18 months: BC events rate: ①6-month following the 18-month landmark (24 months post enrollment:0.3% (0.0%-2.3%) VS 3.0% (1.1%-7.9%); ②18-month following the 18-month landmark (36 months post enrollment):2.8% (1.3%-5.7%) VS 4.7% (2.2%-10%); ③HR=0.532(0.272-1.043)	1、;3-year incidence of BC events: interruption of adjuvant endocrine therapy to attempt pregnancy:(1)BRCA status①Positive:5/38,14.5% (6.3–31.6); ② Other mutation:4/21,10.5% (2.7–35.9); ③ No documented mutation:35/457,8.4% (6.1–11.5);(2)HER2 status①Positive:6/134,4.9% (2.2–10.6);②Negative:38/382,10.4% (7.6–14.1);(3)No. of positive lymph nodes①0:21/342,6.6%(4.3–10.0);② 1-3:18/151,12.6% (8.1–19.3); ③ 4-9:5/23,18.7% (7.4–42.6);(4)Tumor size①≤2 cm:24/331,7.6% (5.1–11.2);② >2 to ≤5 cm:16/161,10.3% (6.3–16.5);③>5 cm:4/21,21.1% (8.3–47.6);(5)Tumor histologic grade①1:5/89,6.4% (2.7–14.8);② 2:21/252,8.9% (5.8–13.6);③3:18/172,10.4% (6.6–16.2);(6)Breast surgery①Mastectomy:29/233,12.7% (8.9–17.9);② Breast-conserving procedure:15/283,5.7% (3.4–9.5);(7)Previous endocrine therapy ①SERM alone:20/215, 9.9% (6.4–15.1);②SERM and OFS:20/184,11.4% (7.4–17.4);③ AI and OFS:1/82,1.2%(0.2–8.4);④ Other:3/35,8.8% (2.9–24.9);(8)Previous chemotherapy ①Anthracycline-based:8/36,19.4%(9.8–36.5);② Taxane-based:4/66,6.7% (2.5–16.9);③ Both anthracycline- and taxane-based:16/215,8.4% (5.2–13.4);④Neither anthracycline- nor taxane-based:0/3;⑤ None:16/196,8.4% (5.1–13.6)
47	Hideyuki Iwahata	①ER-positive, interruption of endocrine therapy to attempt pregnancy:56/0; ②ER-positive, interruption of endocrine therapy to successfully pregnancy:35/0;	①Recurrence rate: interruption of adjuvant endocrine therapy to attempt pregnancy:3/46	/
48	Hatem A. Azim Jr	①ER-positive, interruption of endocrine therapy to attempt pregnancy:516;②ER-positive, interruption of endocrine therapy to successfully pregnancy VS no pregnancy:368/129;③Patients with a pregnancy at or before 18 months VS patients without a pregnancy at or before 18 months:305/137;	/	1、;3-year incidence of BC events:①ovarian stimulation for cryopreservation at diagnosis 179 VS no ovarian stimulation for cryopreservation at diagnosis 318: 9.7%(6.0-15.4) VS 8.7% (6.0-12.5);2、;BC events rate: ovarian stimulation for IVF after enrollment 80 VS no ovarian stimulation for IVF after enrollment436: ①18-month following the 24-month landmark:2/71 (71 of 80 reached the 24-month landmark) VS 8/326 (326 of 436 reached the landmark)
49	Matteo Lambertini	BRCA carriers:543/0	/	1、;Disease-free survival: ART pregnancy group VS spontaneous pregnancy group:13/107 VS 118/436, p=0.147, HR = 0.72(0.38-1.33)
50	Matteo Lambertini	BRCA carriers:659/4073	Pregnancy VS pregnancy: ①Disease-free survival: HR = 0.99(0.81-1.20), P = 0.90,204/659 VS 1479/4073; ②BC–specific survival: HR = 0.60(0.40-0.88), P = 0.009,35/659 VS 523/4073; ③Overall survival: HR = 0.58(0.40-0.85), P = 0.005,39/659 VS 570/4073	1、;Disease-free survival: pregnancy VS no pregnancy:(1)①BRCA1: HR = 0.80(0.63-1.01);②BRCA2:HR=1.55(1.12-2.16);③BRCA1 and BRCA2:HR=4.49 (0.28-72.17);④BRCA, unknown if BRCA1 or BRCA2:NE;(2)①HR–positive: HR = 1.30(0.95-1.76); ②HR–negative: HR = 0.76(0.60-0.95);③Unknown: HR = 0.28 (0.04-2.21);(3)ERBB2 status:①Positive: HR = 0.61 (0.22-1.71);②Negative: HR = 1.07 (0.87-1.31);③Unknown: HR = 0.42 (0.17-1.02);(4)use of Chemotherapy:①No chemotherapy: HR = 0.77 (0.39-1.52);②(Neo)adjuvant chemotherapy: HR = 1.00 (0.82-1.23);③Unknown: HR = 0.77 (0.39-1.52);(5) use of endocrine therapy:①no use of endocrine therapy: HR = 0.85(0.67-1.08); ②use of endocrine therapy: HR = 1.55(1.08-2.21);③Unknown: HR = 0.13 (0.01-2.95);(6)the interval between diagnosis and pregnancy:①≤ 2 years: HR = 0.52 (0.37-0.73); ②> 2 years: HR = 0.66 (0.54-0.80);(7)Reproductive status:①Abortion/miscarriage: HR = 0.49 (0.32-0.76);②Completed pregnancy: HR = 0.65 (0.54-0.79);(8)Breastfeeding:①Not applicable (pregnancy not completed/ongoing)/unknown: HR = 0.68 (0.51-0.91);② Breastfed: HR = 0.76 (0.55-1.05);③ No breastfeeding: HR = 0.50 (0.37-0.67);2、;BC–specific survival: pregnancy VS no pregnancy:(1)①BRCA1: HR = 0.44 (0.26-0.73);②BRCA2:HR=1.02 (0.57-1.81);③BRCA1 and BRCA2:NE;④BRCA, unknown if BRCA1 or BRCA2:NE;(2)①HR–positive: HR = 0.80 (0.44-1.45); ②HR–negative: HR = 0.44 (0.28-0.71);③Unknown: NE;(3)ERBB2 status:①Positive: HR = 0.52 (0.11-2.36);②Negative: HR = 0.60 (0.40-0.91);③Unknown: HR = 0.59 (0.12-2.97);(4)use of Chemotherapy:①No chemotherapy: HR = 1.08 (0.30-3.91);②(Neo)adjuvant chemotherapy: HR = 0.57 (0.38-0.86);③Unknown: NE;(5)use of endocrine therapy:①no use of endocrine therapy: HR = 0.59 (0.36-0.95);② use of endocrine therapy: HR = 0.63 (0.33-1.20);③Unknown: NE;(6)the interval between diagnosis and pregnancy:①≤ 2 years: HR = 0.34 (0.18-0.63);② > 2 years: HR = 0.50 (0.31-0.80);(7)Reproductive status:①Abortion/miscarriage: HR = 0.45 (0.22-0.93);②Completed pregnancy: HR = 0.44 (0.28-0.68);(8)Breastfeeding:①Not applicable (pregnancy not completed/ongoing)/unknown: HR = 0.54 (0.30-0.94);②Breastfed: HR = 0.45 (0.22-0.93);③No breastfeeding: HR = 0.34 (0.17-0.70);3、;Overall survival: pregnancy VS no pregnancy:(1)①BRCA1: HR = 0.42 (0.26-0.68);②BRCA2:HR=1.00 (0.58-1.72);③BRCA1 and BRCA2:NE;④BRCA, unknown if BRCA1 or BRCA2:NE;(2)①HR–positive: HR = 0.78 (0.44-1.38); ②HR–negative: HR = 0.43 (0.27-0.66);③Unknown: NE;(3)ERBB2 status:①Positive: HR = 0.73 (0.21-2.60);②Negative: HR = 0.56 (0.38-0.83);③Unknown: HR = 0.62 (0.13-3.02);(4)use of Chemotherapy:①No chemotherapy: HR = 0.90 (0.25-3.24);②(Neo)adjuvant chemotherapy: HR = 0.56 (0.38-0.82);③Unknown: NE;(5)use of endocrine therapy:①no use of endocrine therapy: HR = 0.57 (0.36-0.90);② use of endocrine therapy: HR = 0.59 (0.31-1.13);③Unknown: NE;(6)the interval between diagnosis and pregnancy:①≤ 2 years: HR = 0.35 (0.19-0.64);② > 2 years: HR = 0.47 (0.30-0.74);(7)Reproductive status:①Abortion/miscarriage: HR = 0.42 (0.20-0.86);②Completed pregnancy: HR = 0.44 (0.29-0.67);(8)Breastfeeding:①Not applicable (pregnancy not completed/ongoing)/unknown: HR = 0.49 (0.28-0.86);② Breastfed: HR = 0.50 (0.26-0.98);③No breastfeeding: HR = 0.33 (0.17-0.66)
51	Young- jin Lee	①ER-positive, interruption of endocrine therapy to attempt pregnancy:76; ②ER-positive, interruption of endocrine therapy to successfully pregnancy VS no prepregnancy:53/23	1、;Interruption of adjuvant endocrine therapy to attempt pregnancy:①Recurrence rate:13/76;②loco-regional Recurrence rate:8/76;③Distant Recurrence rate:5/76;2、;Interruption of adjuvant endocrine therapy to successfully pregnancy VS no pregnancy:①Recurrence rate:9/53 VS 4/23	/
52	Ji Hye Kim	13/94	Pregnancy VS nulliparous VS previous pregnancy: ①Overall survival: P = 0.608; ②Disease-free survival: P = 0.591	/

### 3.2 Quality evaluation

The results are presented in **Appendix B**. Among the cohort studies, 21 were categorized as high-quality and 17 as medium-quality. Among the case–control studies, two were deemed medium-quality, and one was deemed high-quality.

### 3.3 Cancer outcomes

#### 3.3.1 Overall survival

**Figure 2:**
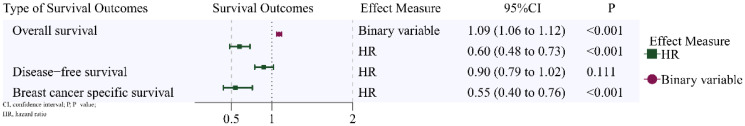
Forest plot of survival outcomes in pregnant BC patients compared with nonpregnant BC patients.

A meta-analysis of OS was performed on the basis of the dichotomous data (number of events) reported by 18 studies (6, 12–28), which included 3,390 pregnant BC patients and 52,166 nonpregnant BC patients. The pregnancy group had longer OS [RR = 1.09, 95% CI (1.06, 1.12), *P* < 0.001] (**Figure 2**). A funnel plot (**Figure C.33**) and Egger’s test (*P* = 0.099) revealed no significant publication bias. The sensitivity analysis is shown in **Figure C.34**.

A separate meta-analysis of OS was conducted using HRs directly extracted from Cox proportional hazards models in 9 studies (29–37), involving 4,037 pregnant BC patients and 67,391 nonpregnant BC patients. The pregnancy group had longer OS [HR = 0.60, 95% CI (0.48, 0.73), *P* < 0.001] (**Figure 2**). A funnel plot (**Figure C.35**) and Egger’s test (*P* = 0.770) revealed no significant publication bias. The sensitivity analysis results are shown in **Figure C.36**.

#### 3.3.2 Disease-free survival

Seven studies (12, 19, 22, 35–38) compared the DFS of 1,666 pregnant BC patients and 10,247 nonpregnant BC patients. The pregnancy group had numerically longer DFS compared to the nonpregnancy group, although this difference did not reach statistical significance [HR = 0.90, 95% CI (0.79, 1.02), *P* = 0.111] (**Figure 2**). A funnel plot (**Figure C.37**) and Egger’s test (*P* = 0.197) revealed no significant publication bias. The results of the sensitivity analysis are shown in **Figure C.38**.

#### 3.3.3 Breast cancer-specific survival

Three studies (34, 38, 39) compared the BC-specific survival of 1,232 pregnant BC patients with that of 4,862 nonpregnant BC patients. The BC-specific survival of the pregnancy group was longer [HR = 0.55, 95% CI (0.40, 0.76), *P* < 0.001] (**Figure 2**). A funnel plot (**Figure C.39**) and Egger’s test (*P* = 0.972) revealed no significant publication bias. The results of the sensitivity analysis are shown in **Figure C.40**.

#### 3.3.4 Recurrence rate

**Figure 3:**
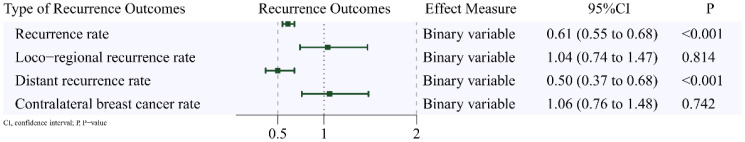
Forest plot of recurrence outcomes in pregnant BC patients compared with nonpregnant BC patients.

Seventeen studies (12, 13, 18, 21, 23, 24, 26, 27, 35, 40–47) compared the recurrence rates of the 2,273 pregnant BC patients with those of 39,019 nonpregnant BC patients. The pregnancy group had a lower recurrence rate [RR = 0.61, 95% CI (0.55, 0.68), *P* < 0.001] (**Figure 3**). A funnel plot (**Figure C.41**) and Egger’s test (*P* = 0.763) revealed no significant publication bias. The results of the sensitivity analysis are shown in **Figure C.42**.

#### 3.3.5 Loco-regional recurrence rate

Four studies (12, 18, 35, 47) reported the loco-regional recurrence rate of 835 pregnant BC patients compared with 4,826 nonpregnant BC patients. The loco-regional recurrence rate was slightly higher in the pregnancy group, but the difference was not statistically significant [RR = 1.04, 95% CI (0.74, 1.47), *P* = 0.814] (**Figure 3**). A funnel plot (**Figure C.43**) and Egger’s test results (*P* = 0.012) revealed significant publication bias. The results of the sensitivity analysis are shown in **Figure C.44**.

#### 3.3.6 Distant recurrence rate

Five studies (12, 18, 35, 40, 47) reported the distant recurrence rate of 885 pregnant BC patients and 6,895 nonpregnant BC patients. The distant recurrence rate of the pregnancy group was lower [RR = 0.50, 95% CI (0.37, 0.68), *P* < 0.001] (**Figure 3**). A funnel plot (**Figure C.45**) and Egger’s test (*P* = 0.520) revealed no significant publication bias. The sensitivity analysis is shown in **Figure C.46**.

#### 3.3.7 Contralateral breast cancer rate

Three studies (12, 35, 47) reported the contralateral BC rate of 815 pregnant BC patients and 4,806 nonpregnant BC patients. The pregnancy group had a higher contralateral BC rate, but the difference was not statistically significant [RR = 1.06, 95% CI (0.76, 1.48), *P* = 0.742] (**Figure 3**). A funnel plot (**Figure C.47**) and Egger’s test (*P* = 0.456) revealed no significant publication bias. However, sensitivity analysis indicated that the results were unstable and were influenced predominantly by one study (35) (**Figure C.48**).

#### 3.3.8 Relapse-free survival rate

Three studies (26, 42, 48) reported the 5-year RFS rate, including 1,175 pregnant BC patients. The result was 78% [95% CI (0.58, 0.97), *P <* 0.001] (**Figure C.1**). A funnel plot (**Figure C.49**) and Egger’s test (*P* = 0.411) revealed no significant publication bias. The results of the sensitivity analysis are shown in **Figure C.50**.

Two studies (26, 42) reported the 5-year RFS rate, including 31,092 nonpregnant BC patients. The result was 65% [95% CI (0.34, 0.95), *P <* 0.001] (**Figure C.2**). A funnel plot (**Figure C.51**) indicated that there might be publication bias. Sensitivity analysis indicated that the pooled estimate was unstable (**Figure C.52**).

#### 3.3.9 5-year disease-free survival rate

Four studies (13, 18, 43, 49) reported the 5-year DFS rate, including 138 pregnant BC patients. The result was 80% [95% CI (0.64, 0.95), *P <* 0.001] (**Figure C.3**). A funnel plot (**Figure C.53**) and Egger’s test (*P* = 0.934) revealed no significant publication bias. The sensitivity analysis results are shown in **Figure C.54**.

Four studies (13, 18, 43, 49) reported the 5-year DFS rate, including 319 nonpregnant BC patients. The result was 60% [95% CI (0.33, 0.88), *P <* 0.001] (**Figure C.4**). A funnel plot (**Figure C.55**) and Egger’s test (*P* = 0.682) revealed no significant publication bias. The sensitivity analysis is shown in **Figure C.56**.

#### 3.3.10 5-year survival rate

Nine studies (13, 14, 16, 18, 26, 31, 48, 50, 51) reported on the 5-year survival rate of 1,564 BC patients in the pregnancy group. The result was 88% [95% CI (0.83,0.93), *P <* 0.001] (**Figure C.5**). A funnel plot (**Figure C.57**) and Egger’s test (*P* = 0.012) revealed significant publication bias. The sensitivity analysis is shown in **Figure C.58**.

Seven cohort studies (13, 14, 16, 18, 26, 31, 49) reported on the 5-year survival rate of 35,348 BC patients in the nonpregnancy group. The result was 82% [95% CI (0.78,0.86), *P <* 0.001] (**Figure C.6**). A funnel plot (**Figure C.59**) and Egger’s test (*P* = 0.198) revealed no significant publication bias. The results of the sensitivity analysis are shown in **Figure C.60**.

#### 3.3.11 10-year survival rate

Six studies (14, 16, 36, 48, 50, 51) reported the 10-year survival rate of 676 pregnant patients with BC. The result was 82% [95% CI (0.71,0.92), *P <* 0.001] (**Figure C.7**). A funnel plot (**Figure C.61**) and Egger’s test (*P* = 0.027) revealed significant publication bias. The sensitivity analysis is shown in **Figure C.62**.

Three studies (14, 16, 36) reported the 10-year survival rate of 931 nonpregnant BC patients. The result was 75% [95% CI (0.54,0.97), *P <* 0.001] (**Figure C.8**). A funnel plot (**Figure C.63**) and Egger’s test (*P* = 0.562) revealed no significant publication bias. The sensitivity analysis is shown in **Figure C.64**.

### 3.4 Subgroup analysis

#### 3.4.1 BC stage

Pregnancy was a protective factor for OS in stage I BC patients (15, 17, 25, 32, 34) [HR = 0.59, 95% CI (0.43, 0.80), *P =* 0.001] (**Figure C.9**). A funnel plot (**Figure C.65**) and Egger’s test (*P* = 0.106) revealed no significant publication bias. The results of the sensitivity analysis are shown in **Figure C.66**.

Stage II and III BC patients in the pregnant group had longer OS (15, 17, 25, 32) [HR = 0.57, 95% CI (0.43, 0.77), *P <* 0.001] (**Figure C.10**). A funnel plot (**Figure C.67**) and Egger’s test (*P* = 0.554) revealed no significant publication bias. Sensitivity analysis indicated that the results were unstable and were predominantly influenced by one study (17) (**Figure C.68**).

#### 3.4.2 ER status

##### 3.4.2.1 Overall survival

ER-positive BC patients in the pregnant group had longer OS (25, 32, 37, 38) [HR = 0.75, 95% CI (0.58, 0.96), *P* = 0.024] (**Figure C.11**). A funnel plot **(Figure C.69**) and Egger’s test (*P* = 0.072) revealed no significant publication bias. Sensitivity analysis indicated that the pooled estimate was unstable and was influenced by the articles published in 2022 (32) and 2020 (25) (**Figure C.70**).

ER-negative BC patients in the pregnant group had longer OS (25, 32, 37, 38) [HR = 0.52, 95% CI (0.40, 0.69), *P <* 0.001] (**Figure C.12**). A funnel plot (**Figure C.71**) and Egger’s test (*P* = 0.968) revealed no significant publication bias. The sensitivity analysis results are shown in **Figure C.72**.

##### 3.4.2.2 Disease free survival

ER-positive BC patients in the pregnancy group had shorter DFS, but the difference was not statistically significant (35, 37, 38) [HR = 1.14, 95% CI (0.93, 1.39), *P* = 0.203] (**Figure C.13**). A funnel plot (**Figure C.73**) and Egger’s test (*P* = 0.591) revealed no significant publication bias. The sensitivity analysis results are shown in **Figure C.74**.

ER-negative BC patients in the pregnant group had longer DFS (35, 37, 38) [HR = 0.74, 95% CI (0.62, 0.89), *P* = 0.001] (**Figure C.14**). A funnel plot (**Figure C.75**) and Egger’s test (*P* = 0.322) revealed no significant publication bias. The sensitivity analysis is shown in **Figure C.76**.

#### 3.4.3 *BRCA* mutation

The pregnancy group had longer DFS in *BRCA* mutation BC patients, although this difference was not statistically significant (35, 38) [HR = 0.96, 95% CI (0.81, 1.14), *P* = 0.640] (**Figure C.15**). A funnel plot is shown in **Figure C.77**. Among *BRCA1* mutation BC patients, those in the pregnancy group had longer DFS (35, 38) [HR = 0.76, 95% CI (0.62, 0.94), *P* = 0.010]. Among *BRCA2* mutation BC patients, those in the pregnancy group had shorter DFS (35, 38) [HR = 1.64, 95% CI (1.23, 2.18), *P* = 0.001].

#### 3.4.4 HER2 status

The pregnancy group had longer DFS in HER2 positive BC patients, although this difference was not statistically significant (22, 38) [HR = 0.90, 95% CI (0.49, 1.66), *P* = 0.737] (**Figure C.16**). A funnel plot (**Figure C.78**) indicated that there was no publication bias.

#### 3.4.5 Hormone therapy

The pregnancy group had better OS among BC patients who had received hormone therapy (17, 25, 33, 36, 38) [HR = 0.36, 95% CI (0.25, 0.50), *P <* 0.001] (**Figure C.17**). A funnel plot (**Figure C.79**) and Egger’s test (*P* = 0.872) revealed no significant publication bias. The sensitivity analysis results are shown in **Figure C.80**.

The pregnancy group had better OS among BC patients who did not receive hormone therapy (17, 25, 33, 36, 38) [HR = 0.41, 95% CI (0.26, 0.64), *P <* 0.001] (**Figure C.18**). A funnel plot (**Figure C.81**) and Egger’s test (*P* = 0.714) revealed no significant publication bias. The sensitivity analysis results are shown in **Figure C.82**.

#### 3.4.6 Chemotherapy

The pregnancy group had longer OS in BC patients who had received chemotherapy (17, 25, 32, 33, 36, 38) [HR = 0.48, 95% CI (0.31, 0.74), *P* = 0.001] (**Figure C.19**). A funnel plot (**Figure C.83**) and Egger’s test (*P* = 0.364) revealed no significant publication bias. The sensitivity analysis results are shown in **Figure C.84**.

The pregnancy group had longer OS among BC patients who did not receive chemotherapy (17, 25, 38) [HR = 0.57, 95% CI (0.39, 0.84), *P* = 0.004] (**Figure C.20**). A funnel plot (**Figure C.85**) and Egger’s test (*P =* 0.721) revealed no significant publication bias. Sensitivity analysis indicated that the pooled estimate was unstable and was influenced by the article (17). (**Figure C.86**).

#### 3.4.7 Endocrine therapy plus chemotherapy

The pregnancy group had longer OS in BC patients who had received endocrine therapy plus chemotherapy (33, 36) [HR = 0.31, 95% CI (0.18, 0.54), *P <* 0.001] (**Figure C.21**). A funnel plot (**Figure C.87**) indicated that there was no publication bias.

#### 3.4.8 Chemotherapy plus trastuzumab

The pregnancy group had longer OS in BC patients who had received chemotherapy plus trastuzumab, although this difference was not statistically significant (33, 36) [HR = 0.35, 95% CI (0.09, 1.39), *P* = 0.135] (**Figure C.22**). A funnel plot (**Figure C.88**) indicated that there might be publication bias.

#### 3.4.9 ER positivity and interruption of adjuvant endocrine therapy

The recurrence rate was not significantly lower in the pregnant group of ER-positive BC patients who had interruption of adjuvant endocrine therapy (46, 47) [RR = 0.77, 95% CI (0.57, 1.05), *P* = 0.098] (**Figure C.23**). A funnel plot (**Figure C.89**) indicated that there was no publication bias.

#### 3.4.10 Lymph node status

The pregnancy group had longer OS in patients with lymph node-positive BC, although this difference was not statistically significant (17, 25, 28) [RR = 1.10, 95% CI (0.99, 1.22), *P* = 0.074] (**Figure C.24**). A funnel plot (**Figure C.90**) and Egger’s test (*P* = 0.695) revealed no significant publication bias. The sensitivity analysis is shown in **Figure C.91**.

The pregnancy group had longer OS in patients with lymph node-negative BC (17, 25, 28) [RR = 1.04, 95% CI (1.00, 1.08), *P* = 0.033] (**Figure C.25**). A funnel plot (**Figure C.92**) and Egger’s test (*P* = 0.267) revealed no significant publication bias. Sensitivity analysis indicated that the results were unstable (**Figure C.93**).

#### 3.4.11 Interval between diagnosis and pregnancy

The pregnancy group had longer DFS in BC patients with an interval between diagnosis and pregnancy ≤2 years (37, 38)[HR = 0.58, 95% CI (0.46, 0.74), *P <* 0.001] (**Figure C.26**). A funnel plot (**Figure C.94**) indicated that there was no publication bias.

The pregnancy group had longer DFS in BC patients, with an interval between diagnosis and pregnancy >2 years, although this difference was not statistically significant (37, 38) [HR = 0.85, 95% CI (0.50, 1.42), *P* = 0.529] (**Figure C.27**). A funnel plot (**Figure C.95**) indicated that there might be publication bias.

#### 3.4.12 Pregnancy type

BC patients who had received assisted reproductive technology(ART) had a lower recurrence rate than those who had achieved spontaneous conception (12, 45, 52, 53) [RR = 0.18, 95% CI (0.06, 0.57), *P* = 0.003] (**Figure C.28**). A funnel plot (**Figure C.96**) and Egger’s test (*P* = 0.457) revealed no significant publication bias. Sensitivity analysis indicated that the pooled estimate was unstable and was influenced by the article (52) (**Figure C.97**).

#### 3.4.13 Reproductive status

BC patients with full-term pregnancies had longer OS than nonpregnant BC patients did (12, 15, 17, 25, 26, 29, 30, 32, 33, 36, 38) [HR = 0.47, 95% CI (0.36, 0.60), *P <* 0.001] (**Figure C.29**). A funnel plot (**Figure C.98**) and Egger’s test (*P* = 0.449) revealed no significant publication bias. The sensitivity analysis is shown in **Figure C.99**.

BC patients with spontaneous or induced abortions had longer OS than nonpregnant BC patients (25, 26, 36, 38) [HR = 0.67, 95% CI (0.48, 0.94), *P* = 0.020] (**Figure C.30**). A funnel plot (**Figure C.100**) and Egger’s test (*P* = 0.848) revealed no significant publication bias. Sensitivity analysis indicated that the pooled estimate was unstable and was influenced by the article (6) (**Figure C.101**).

#### 3.4.14 Breastfeeding

Pregnant BC patients who had breastfed their newborns had longer DFS than nonpregnant BC patients, although this difference was not statistically significant (37, 38) [HR = 0.75, 95% CI (0.55, 1.03), *P* = 0.072] (**Figure C.31**). A funnel plot revealed no significant publication bias (**Figure C.102**).

Pregnant BC patients who had not breastfed their newborns had longer DFS than nonpregnant BC patients, although this difference was not statistically significant (37, 38) [HR = 0.82, 95% CI (0.29, 2.30), *P* = 0.702] (**Figure C.32**). A funnel plot indicated that there might be publication bias (**Figure C.103**).

## 4 Discussion

Recently, a growing number of studies have indicated that pregnant patients with BC do not face an elevated risk of relapse (6, 54). However, owing to the complexity of cancer and its treatment, oncologists remain concerned about the outcomes of pregnancy following a BC diagnosis. This systematic review provides updated evidence on pregnancy after breast cancer.

Our study revealed that pregnant BC patients had better cancer outcomes, such as OS, BC-specific survival, recurrence rate, and distant recurrence rate, than nonpregnant BC patients did. Subgroup analysis revealed that pregnant BC patients had longer OS than nonpregnant BC patients did, and the results were not related to BC stage, ER status, hormone therapy, chemotherapy, or endocrine therapy combined with chemotherapy or reproductive status.

The pregnancy group had significantly longer DFS among ER-negative BC patients. However, ER-positive BC patients of the pregnancy group had shorter DFS, although the difference was not statistically significant. ER-positive pregnant patients might interrupt adjuvant endocrine therapy because of pregnancy, which might result in a shorter DFS. However, the POSITIVE trial (5) indicated that temporarily discontinuing endocrine therapy did not result in an increased short-term risk of BC events. We suspected that the small sample size of the included articles or the disparity in outcome indicators compared with those of the POSITIVE trial might explain the observed results. We look forward to longer follow-up of the POSITIVE trial to support this line of inquiry.

Among *BRCA1* mutation BC patients, pregnant BC patients had significantly longer DFS. However, among patients with *BRCA2* mutation BC patients, pregnant patients with BC had significantly shorter DFS. Pregnancy has potential protective effects on patients with *BRCA1* mutations and may have negative effects on *BRCA2* mutation carriers (35). Late age at first birth, breastfeeding, and delayed menarche protect only BC patients with *BRCA1* mutations (55). The *BRCA2* mutation population deserves further attention, and pregnancy requires careful consideration.

Notably, the results revealed that compared with BC patients who had achieved spontaneous conception, BC patients who had received ART had a lower recurrence rate. One study revealed that ART treatment could reduce the incidence of BC (56), but the literature does not support that ART treatment is associated with better survival results. This may reflect selection bias. BC patients with earlier stages and better physical condition are more likely to consider ART for pregnancy, which could contribute to a lower recurrence rate.

This study has several limitations. First, most of the included studies were retrospective in design. Second, the limited number of studies available for certain subgroups reduced the robustness of the findings. Most importantly, direct comparisons between clinically relevant subgroups were precluded by the lack of available data; consequently, the analyses had to rely on indirect comparisons, which are susceptible to unmeasured confounding. Furthermore, the results may be influenced by a “healthy mother effect”, wherein women who conceive after a cancer diagnosis tend to have earlier-stage disease and a lower intrinsic risk of relapse, potentially biasing the outcomes. To address these limitations, we emphasize the need for future large-scale, prospective studies. Such studies should be specifically designed to enable direct comparisons between carefully defined subgroups, incorporating key clinicopathological variables such as breastfeeding status, nodal status, reproductive history, and the timing of pregnancy relative to diagnosis, among others.

## 5 Conclusions

Our results indicate that pregnancy after a BC diagnosis does not lead to adverse cancer outcomes. *BRCA2* mutation may be a harmful factor for DFS in pregnant BC patients and deserves attention.

## Data Availability

The original contributions presented in the study are included in the article/**Supplementary Material**. Further inquiries can be directed to the corresponding author.
