# Supplementary material for: Cancer outcomes of pregnancy after diagnosis of breast cancer in premenopausal women: an updated systematic review and meta-analysis
Source: Front Oncol. 2025 Oct 14;15:1644566. doi: 10.3389/fonc.2025.1644566 (PMC12558755; doi:10.3389/fonc.2025.1644566)
Supplement: Supplementary file 1 [file DataSheet1.docx]

**Appendix A Search strategy**

1、PubMed -5176

((("Breast Neoplasms"[Mesh]) OR ((Breast Neoplasm [Title/Abstract]) OR (Breast Neoplasms [Title/Abstract]) OR (Neoplasm, Breast[Title/Abstract]) OR (Neoplasms, Breast[Title/Abstract]) OR (Breast Tumors[Title/Abstract]) OR (Breast Tumor[Title/Abstract]) OR (Tumor, Breast[Title/Abstract]) OR (Tumors, Breast[Title/Abstract]) OR (Breast Cancer[Title/Abstract]) OR (Cancer, Breast[Title/Abstract]) OR (Mammary Cancer[Title/Abstract]) OR (Cancer, Mammary[Title/Abstract]) OR (Cancers, Mammary[Title/Abstract]) OR (Mammary Cancers[Title/Abstract]) OR (Malignant Neoplasm of Breast[Title/Abstract]) OR (Breast Malignant Neoplasm[Title/Abstract]) OR (Breast Malignant Neoplasms[Title/Abstract]) OR (Malignant Tumor of Breast[Title/Abstract]) OR (Breast Malignancy[Title/Abstract]) OR (Breast Malign*[Title/Abstract]) OR (Breast Malignant Tumor[Title/Abstract]) OR (Breast Malignant Tumors[Title/Abstract]) OR (Cancer of Breast[Title/Abstract]) OR (Cancer of the Breast[Title/Abstract]) OR (Mammary Carcinoma, Human[Title/Abstract]) OR (Carcinoma, Human Mammary[Title/Abstract]) OR (Carcinomas, Human Mammary[Title/Abstract]) OR (Human Mammary Carcinomas[Title/Abstract]) OR (Mammary Carcinomas, Human[Title/Abstract]) OR (Human Mammary Carcinoma[Title/Abstract]) OR (Mammary Neoplasms, Human[Title/Abstract]) OR (Human Mammary Neoplasm[Title/Abstract]) OR (Human Mammary Neoplasms[Title/Abstract]) OR (Neoplasm, Human Mammary[Title/Abstract]) OR (Neoplasms, Human Mammary[Title/Abstract]) OR (Mammary Neoplasm, Human[Title/Abstract]) OR (Breast Carcinoma[Title/Abstract]) OR (Breast Carcinomas[Title/Abstract]) OR (Carcinoma, Breast[Title/Abstract]) OR (Carcinomas, Breast[Title/Abstract]))) AND (((((("Pregnancy"[Mesh]) OR ("Fertilization"[Mesh])) OR ("Parturition"[Mesh])) OR ("Fertility"[Mesh])) OR ("Obstetrics"[Mesh])) OR ((Pregnancy[Title/Abstract]) OR (Pregnancies[Title/Abstract]) OR (Gestation[Title/Abstract]) OR (Pregnant[Title/Abstract]) OR (Fertilization[Title/Abstract]) OR (Fertilizations[Title/Abstract]) OR (Fertilization, Delayed[Title/Abstract]) OR (Delayed Fertilization[Title/Abstract]) OR (Delayed Fertilizations[Title/Abstract]) OR (Fertilizations, Delayed[Title/Abstract]) OR (Fertilization, Polyspermic[Title/Abstract]) OR (Fertilizations, Polyspermic[Title/Abstract]) OR (Polyspermic Fertilization[Title/Abstract]) OR (Polyspermic Fertilizations[Title/Abstract]) OR (Conception[Title/Abstract]) OR (Conceptions[Title/Abstract]) OR (Conceiving[Title/Abstract]) OR (Parturition[Title/Abstract]) OR (Parturitions[Title/Abstract]) OR (Birth[Title/Abstract]) OR (Births[Title/Abstract]) OR (Childbirth[Title/Abstract]) OR (Childbirths[Title/Abstract]) OR (Fertility[Title/Abstract]) OR (Fecundability[Title/Abstract]) OR (Fecundity[Title/Abstract]) OR (Differential Fertility[Title/Abstract]) OR (Fertility, Differential[Title/Abstract]) OR (Fertility Determinants[Title/Abstract]) OR (Determinant, Fertility[Title/Abstract]) OR (Determinants, Fertility[Title/Abstract]) OR (Fertility Determinant[Title/Abstract]) OR (Subfecundity[Title/Abstract]) OR (Fertility Preferences[Title/Abstract]) OR (Fertility Preference[Title/Abstract]) OR (Preference, Fertility[Title/Abstract]) OR (Preferences, Fertility[Title/Abstract]) OR (Fertility, Below Replacement[Title/Abstract]) OR (Below Replacement Fertility[Title/Abstract]) OR (Marital Fertility[Title/Abstract]) OR (Fertility, Marital[Title/Abstract]) OR (Natural Fertility[Title/Abstract]) OR (Fertility, Natural[Title/Abstract]) OR (World Fertility Survey[Title/Abstract]) OR (Fertility Survey, World[Title/Abstract]) OR (Fertility Surveys, World[Title/Abstract]) OR (Survey, World Fertility[Title/Abstract]) OR (Surveys, World Fertility[Title/Abstract]) OR (World Fertility Surveys[Title/Abstract]) OR (Fertility Incentives[Title/Abstract]) OR (Fertility Incentive[Title/Abstract]) OR (Obstetrics[Title/Abstract]) OR (Obstetrical outcomes[Title/Abstract])))) AND ((((("Cohort Studies"[Mesh]) OR ("Case-Control Studies"[Mesh])) OR ("Clinical Trial" [Publication Type])) OR ("Randomized Controlled Trial" [Publication Type])) OR ((cohort studies[Title/Abstract]) OR (case-control studies[Title/Abstract]) OR (comparative study[Title/Abstract]) OR (cohort*[Title/Abstract]) OR (compared[Title/Abstract]) OR (case control[Title/Abstract]) OR (case series[Title/Abstract]) OR (randomized controlled trial[Title/Abstract]) OR (controlled clinical trial[Title/Abstract]) OR (randomized[Title/Abstract]) OR (placebo[Title/Abstract]) OR (drug therapy[Title/Abstract]) OR (randomly[Title/Abstract]) OR (clinical trial[Title/Abstract]))) Filters: from 1000/1/1 - 2024/07/01

2、Embase-7467

#17 #3 AND #10 AND #16 AND [1900-2024]/py 7467

#16 #11 OR #12 OR #13 OR #14 OR #15 9,772,554

#15 'cohort studies':ab,ti OR 'case-control studies':ab,ti OR 'comparative study':ab,ti OR 'cohort*':ab,ti OR 'compared':ab,ti OR 'case control':ab,ti OR 'case series':ab,ti OR 'randomized controlled trial':ab,ti OR 'controlled clinical trial':ab,ti OR 'randomized':ab,ti OR 'placebo':ab,ti OR 'drug therapy':ab,ti OR 'randomly':ab,ti OR 'clinical trial':ab,ti 8836664

#14 'randomized controlled trial'/exp 831,567

#13 'clinical trial'/exp 1,930,414

#12 'case control study'/exp 236,342

#11 'cohort analysis'/exp 1,180,938

#10 #4 OR #5 OR #6 OR #7 OR #8 OR #9 1,824,358

#9 'pregnancy':ab,ti OR 'pregnancies':ab,ti OR 'gestation':ab,ti OR 'pregnant':ab,ti OR 'fertilization':ab,ti OR 'fertilizations':ab,ti OR 'fertilization, delayed':ab,ti OR 'delayed fertilization':ab,ti OR 'delayed fertilizations':ab,ti OR 'fertilizations, delayed':ab,ti OR 'fertilization, polyspermic':ab,ti OR 'fertilizations, polyspermic':ab,ti OR 'polyspermic fertilization':ab,ti OR 'polyspermic fertilizations':ab,ti OR 'conception':ab,ti OR 'conceptions':ab,ti OR 'conceiving':ab,ti OR 'parturition':ab,ti OR 'parturitions':ab,ti OR 'birth':ab,ti OR 'births':ab,ti OR 'childbirth':ab,ti OR 'childbirths':ab,ti OR 'fertility':ab,ti OR 'fecundability':ab,ti OR 'fecundity':ab,ti OR 'differential fertility':ab,ti OR 'fertility, differential':ab,ti OR 'fertility determinants':ab,ti OR 'determinant, fertility':ab,ti OR 'determinants, fertility':ab,ti OR 'fertility determinant':ab,ti OR 'subfecundity':ab,ti OR 'fertility preferences':ab,ti OR 'fertility preference':ab,ti OR 'preference, fertility':ab,ti OR 'preferences, fertility':ab,ti OR 'fertility, below replacement':ab,ti OR 'below replacement fertility':ab,ti OR 'marital fertility':ab,ti OR 'fertility, marital':ab,ti OR 'natural fertility':ab,ti OR 'fertility, natural':ab,ti OR 'world fertility survey':ab,ti OR 'fertility survey, world':ab,ti OR 'fertility surveys, world':ab,ti OR 'survey, world fertility':ab,ti OR 'surveys, world fertility':ab,ti OR 'world fertility surveys':ab,ti OR 'fertility incentives':ab,ti OR 'fertility incentive':ab,ti OR 'obstetrics':ab,ti OR 'obstetrical outcomes':ab,ti 1477975

#8 'obstetrics'/exp 47,618

#7 'fertility'/exp 99,761

#6 'birth'/exp 33,790

#5 'fertilization'/exp 41,547

#4 'pregnancy'/exp 910,890

#3 #1 OR #2 729271

#2 'breast neoplasm':ab,ti OR 'breast neoplasms':ab,ti OR 'neoplasm, breast':ab,ti OR 'neoplasms, breast':ab,ti OR 'breast tumors':ab,ti OR 'breast tumor':ab,ti OR 'tumor, breast':ab,ti OR 'tumors, breast':ab,ti OR 'breast cancer':ab,ti OR 'cancer, breast':ab,ti OR 'mammary cancer':ab,ti OR 'cancer, mammary':ab,ti OR 'cancers, mammary':ab,ti OR 'mammary cancers':ab,ti OR 'malignant neoplasm of breast':ab,ti OR 'breast malignant neoplasm':ab,ti OR 'breast malignant neoplasms':ab,ti OR 'malignant tumor of breast':ab,ti OR 'breast malignancy':ab,ti OR 'breast malign*':ab,ti OR 'breast malignant tumor':ab,ti OR 'breast malignant tumors':ab,ti OR 'cancer of breast':ab,ti OR 'cancer of the breast':ab,ti OR 'mammary carcinoma, human':ab,ti OR 'carcinoma, human mammary':ab,ti OR 'carcinomas, human mammary':ab,ti OR 'human mammary carcinomas':ab,ti OR 'mammary carcinomas, human':ab,ti OR 'human mammary carcinoma':ab,ti OR 'mammary neoplasms, human':ab,ti OR 'human mammary neoplasm':ab,ti OR 'human mammary neoplasms':ab,ti OR 'neoplasm, human mammary':ab,ti OR 'neoplasms, human mammary':ab,ti OR 'mammary neoplasm, human':ab,ti OR 'breast carcinoma':ab,ti OR 'breast carcinomas':ab,ti OR 'carcinoma, breast':ab,ti OR 'carcinomas, breast':ab,ti 554916

#1 'breast cancer'/exp 614,013

3、Cochrane-1054

ID Search Hits

#1 MeSH descriptor: [Breast Neoplasms] explode all trees 20302

#2 (Breast Neoplasm):ti,ab,kw OR (Breast Neoplasms):ti,ab,kw OR (Neoplasm, Breast):ti,ab,kw OR (Neoplasms, Breast):ti,ab,kw OR (Breast Tumors):ti,ab,kw OR (Breast Tumor):ti,ab,kw OR (Tumor, Breast):ti,ab,kw OR (Tumors, Breast):ti,ab,kw OR (Breast Cancer):ti,ab,kw OR (Cancer, Breast):ti,ab,kw OR (Mammary Cancer):ti,ab,kw OR (Cancer, Mammary):ti,ab,kw OR (Cancers, Mammary):ti,ab,kw OR (Mammary Cancers):ti,ab,kw OR (Malignant Neoplasm of Breast):ti,ab,kw OR (Breast Malignant Neoplasm):ti,ab,kw OR (Breast Malignant Neoplasms):ti,ab,kw OR (Malignant Tumor of Breast):ti,ab,kw OR (Breast Malignancy):ti,ab,kw OR (Breast Malign*):ti,ab,kw OR (Breast Malignant Tumor):ti,ab,kw OR (Breast Malignant Tumors):ti,ab,kw OR (Cancer of Breast):ti,ab,kw OR (Cancer of the Breast):ti,ab,kw OR (Mammary Carcinoma, Human):ti,ab,kw OR (Carcinoma, Human Mammary):ti,ab,kw OR (Carcinomas, Human Mammary):ti,ab,kw OR (Human Mammary Carcinomas):ti,ab,kw OR (Mammary Carcinomas, Human):ti,ab,kw OR (Human Mammary Carcinoma):ti,ab,kw OR (Mammary Neoplasms, Human):ti,ab,kw OR (Human Mammary Neoplasm):ti,ab,kw OR (Human Mammary Neoplasms):ti,ab,kw OR (Neoplasm, Human Mammary):ti,ab,kw OR (Neoplasms, Human Mammary):ti,ab,kw OR (Mammary Neoplasm, Human):ti,ab,kw OR (Breast Carcinoma):ti,ab,kw OR (Breast Carcinomas):ti,ab,kw OR (Carcinoma, Breast):ti,ab,kw OR (Carcinomas, Breast):ti,ab,kw 48768

#3 #1 OR #2 48768

#4 MeSH descriptor: [Pregnancy] explode all trees 34185

#5 MeSH descriptor: [Parturition] explode all trees 907

#6 MeSH descriptor: [Obstetrics] explode all trees 338

#7 (Pregnancy):ti,ab,kw OR (Pregnancies):ti,ab,kw OR (Gestation):ti,ab,kw OR (Pregnant):ti,ab,kw OR (Fertilization):ti,ab,kw OR (Fertilizations):ti,ab,kw OR (Fertilization, Delayed):ti,ab,kw OR (Delayed Fertilization):ti,ab,kw OR (Delayed Fertilizations):ti,ab,kw OR (Fertilizations, Delayed):ti,ab,kw OR (Fertilization, Polyspermic):ti,ab,kw OR (Fertilizations, Polyspermic):ti,ab,kw OR (Polyspermic Fertilization):ti,ab,kw OR (Polyspermic Fertilizations):ti,ab,kw OR (Conception):ti,ab,kw OR (Conceptions):ti,ab,kw OR (Conceiving):ti,ab,kw OR (Parturition):ti,ab,kw OR (Parturitions):ti,ab,kw OR (Birth):ti,ab,kw OR (Births):ti,ab,kw OR (Childbirth):ti,ab,kw OR (Childbirths):ti,ab,kw OR (Fertility):ti,ab,kw OR (Fecundability):ti,ab,kw OR (Fecundity):ti,ab,kw OR (Differential Fertility):ti,ab,kw OR (Fertility, Differential):ti,ab,kw OR (Fertility Determinants):ti,ab,kw OR (Determinant, Fertility):ti,ab,kw OR (Determinants, Fertility):ti,ab,kw OR (Fertility Determinant):ti,ab,kw OR (Subfecundity):ti,ab,kw OR (Fertility Preferences):ti,ab,kw OR (Fertility Preference):ti,ab,kw OR (Preference, Fertility):ti,ab,kw OR (Preferences, Fertility):ti,ab,kw OR (Fertility, Below Replacement):ti,ab,kw OR (Below Replacement Fertility):ti,ab,kw OR (Marital Fertility):ti,ab,kw OR (Fertility, Marital):ti,ab,kw OR (Natural Fertility):ti,ab,kw OR (Fertility, Natural):ti,ab,kw OR (World Fertility Survey):ti,ab,kw OR (Fertility Survey, World):ti,ab,kw OR (Fertility Surveys, World):ti,ab,kw OR (Survey, World Fertility):ti,ab,kw OR (Surveys, World Fertility):ti,ab,kw OR (World Fertility Surveys):ti,ab,kw OR (Fertility Incentives):ti,ab,kw OR (Fertility Incentive):ti,ab,kw OR (Obstetrics):ti,ab,kw OR (Obstetrical outcomes):ti,ab,kw 118560

#8 #4 OR #5 OR #6 OR #7 118736

#9 #3 AND #8 1054

time: Start year-Present, search date:2024-07-01

4、Web of Science -5282

# Database: Web of Science Core Collection

# Searches:

1: TS=(Breast Neoplasm OR Breast Neoplasms OR Neoplasm, Breast OR Neoplasms, Breast OR Breast Tumors OR Breast Tumor OR Tumor, Breast OR Tumors, Breast OR Breast Cancer OR Cancer, Breast OR Mammary Cancer OR Cancer, Mammary OR Cancers, Mammary OR Mammary Cancers OR Malignant Neoplasm of Breast OR Breast Malignant Neoplasm OR Breast Malignant Neoplasms OR Malignant Tumor of Breast OR Breast Malignancy OR Breast Malign* OR Breast Malignant Tumor OR Breast Malignant Tumors OR Cancer of Breast OR Cancer of the Breast OR Mammary Carcinoma, Human OR Carcinoma, Human Mammary OR Carcinomas, Human Mammary OR Human Mammary Carcinomas OR Mammary Carcinomas, Human OR Human Mammary Carcinoma OR Mammary Neoplasms, Human OR Human Mammary Neoplasm OR Human Mammary Neoplasms OR Neoplasm, Human Mammary OR Neoplasms, Human Mammary OR Mammary Neoplasm, Human OR Breast Carcinoma OR Breast Carcinomas OR Carcinoma, Breast OR Carcinomas, Breast) Timespan: 1900-01-01 to 2024-07-01 Results: 569,444

2: TS=(Pregnancy OR Pregnancies OR Gestation OR Pregnant OR Fertilization OR Fertilizations OR Fertilization, Delayed OR Delayed Fertilization OR Delayed Fertilizations OR Fertilizations, Delayed OR Fertilization, Polyspermic OR Fertilizations, Polyspermic OR Polyspermic Fertilization OR Polyspermic Fertilizations OR Conception OR Conceptions OR Conceiving OR Parturition OR Parturitions OR Birth OR Births OR Childbirth OR Childbirths OR Fertility OR Fecundability OR Fecundity OR Differential Fertility OR Fertility, Differential OR Fertility Determinants OR Determinant, Fertility OR Determinants, Fertility OR Fertility Determinant OR Subfecundity OR Fertility Preferences OR Fertility Preference OR Preference, Fertility OR Preferences, Fertility OR Fertility, Below Replacement OR Below Replacement Fertility OR Marital Fertility OR Fertility, Marital OR Natural Fertility OR Fertility, Natural OR World Fertility Survey OR Fertility Survey, World OR Fertility Surveys, World OR Survey, World Fertility OR Surveys, World Fertility OR World Fertility Surveys OR Fertility Incentives OR Fertility Incentive OR Obstetrics OR Obstetrical outcomes) Timespan: 1900-01-01 to 2024-07-01 Results: 921,557

3: TS=(cohort studies OR case-control studies OR comparative study OR cohort* OR compared OR case control OR case series OR randomized controlled trial OR controlled clinical trial OR randomized OR placebo OR drug therapy OR randomly OR clinical trial) Timespan: 1900-01-01 to 2024-07-01 Results: 8,763,368

4: #1 AND #2 AND #3 Timespan: 1900-01-01 to 2024-07-01 Results: 5,282

5、Science Direct-1982

Year: 1900-2024

Title, abstract, keywords: (breast cancer OR breast neoplasm OR breast tumor OR breast carcinoma OR breast malignancy) AND (pregnancy OR gestation OR fertility OR parturition)

6、Scopus-7481

( TITLE-ABS-KEY ( "Breast Neoplasm" ) OR TITLE-ABS-KEY ( "Breast Neoplasms" ) OR TITLE-ABS-KEY ( "Neoplasm, Breast" ) OR TITLE-ABS-KEY ( "Neoplasms, Breast" ) OR TITLE-ABS-KEY ( "Breast Tumors" ) OR TITLE-ABS-KEY ( "Breast Tumor" ) OR TITLE-ABS-KEY ( "Tumor, Breast" ) OR TITLE-ABS-KEY ( "Tumors, Breast" ) OR TITLE-ABS-KEY ( "Breast Cancer" ) OR TITLE-ABS-KEY ( "Cancer, Breast" ) OR TITLE-ABS-KEY ( "Mammary Cancer" ) OR TITLE-ABS-KEY ( "Cancer, Mammary" ) OR TITLE-ABS-KEY ( "Cancers, Mammary" ) OR TITLE-ABS-KEY ( "Mammary Cancers" ) OR TITLE-ABS-KEY ( "Malignant Neoplasm of Breast" ) OR TITLE-ABS-KEY ( "Breast Malignant Neoplasm" ) OR TITLE-ABS-KEY ( "Breast Malignant Neoplasms" ) OR TITLE-ABS-KEY ( "Malignant Tumor of Breast" ) OR TITLE-ABS-KEY ( "Breast Malignancy" ) OR TITLE-ABS-KEY ( "Breast Malign*" ) OR TITLE-ABS-KEY ( "Breast Malignant Tumor" ) OR TITLE-ABS-KEY ( "Breast Malignant Tumors" ) OR TITLE-ABS-KEY ( "Cancer of Breast" ) OR TITLE-ABS-KEY ( "Cancer of the Breast" ) OR TITLE-ABS-KEY ( "Mammary Carcinoma, Human" ) OR TITLE-ABS-KEY ( "Carcinoma, Human Mammary" ) OR TITLE-ABS-KEY ( "Carcinomas, Human Mammary" ) OR TITLE-ABS-KEY ( "Human Mammary Carcinomas" ) OR TITLE-ABS-KEY ( "Mammary Carcinomas, Human" ) OR TITLE-ABS-KEY ( "Human Mammary Carcinoma" ) OR TITLE-ABS-KEY ( "Mammary Neoplasms, Human" ) OR TITLE-ABS-KEY ( "Human Mammary Neoplasm" ) OR TITLE-ABS-KEY ( "Human Mammary Neoplasms" ) OR TITLE-ABS-KEY ( "Neoplasm, Human Mammary" ) OR TITLE-ABS-KEY ( "Neoplasms, Human Mammary" ) OR TITLE-ABS-KEY ( "Mammary Neoplasm, Human" ) OR TITLE-ABS-KEY ( "Breast Carcinoma" ) OR TITLE-ABS-KEY ( "Breast Carcinomas" ) OR TITLE-ABS-KEY ( "Carcinoma, Breast" ) OR TITLE-ABS-KEY ( "Carcinomas, Breast" ) ) AND ( TITLE-ABS-KEY ( "Pregnancy" ) OR TITLE-ABS-KEY ( "Pregnancies" ) OR TITLE-ABS-KEY ( "Gestation" ) OR TITLE-ABS-KEY ( "Pregnant" ) OR TITLE-ABS-KEY ( "Fertilization" ) OR TITLE-ABS-KEY ( "Fertilizations" ) OR TITLE-ABS-KEY ( "Fertilization, Delayed" ) OR TITLE-ABS-KEY ( "Delayed Fertilization" ) OR TITLE-ABS-KEY ( "Delayed Fertilizations" ) OR TITLE-ABS-KEY ( "Fertilizations, Delayed" ) OR TITLE-ABS-KEY ( "Fertilization, Polyspermic" ) OR TITLE-ABS-KEY ( "Fertilizations, Polyspermic" ) OR TITLE-ABS-KEY ( "Polyspermic Fertilization" ) OR TITLE-ABS-KEY ( "Polyspermic Fertilizations" ) OR TITLE-ABS-KEY ( "Conception" ) OR TITLE-ABS-KEY ( "Conceptions" ) OR TITLE-ABS-KEY ( "Conceiving" ) OR TITLE-ABS-KEY ( "Parturition" ) OR TITLE-ABS-KEY ( "Parturitions" ) OR TITLE-ABS-KEY ( "Birth" ) OR TITLE-ABS-KEY ( "Births" ) OR TITLE-ABS-KEY ( "Childbirth" ) OR TITLE-ABS-KEY ( "Childbirths" ) OR TITLE-ABS-KEY ( "Fertility" ) OR TITLE-ABS-KEY ( "Fecundability" ) OR TITLE-ABS-KEY ( "Fecundity" ) OR TITLE-ABS-KEY ( "Differential Fertility" ) OR TITLE-ABS-KEY ( "Fertility, Differential" ) OR TITLE-ABS-KEY ( "Fertility Determinants" ) OR TITLE-ABS-KEY ( "Determinant, Fertility" ) OR TITLE-ABS-KEY ( "Determinants, Fertility" ) OR TITLE-ABS-KEY ( "Fertility Determinant" ) OR TITLE-ABS-KEY ( "Subfecundity" ) OR TITLE-ABS-KEY ( "Fertility Preferences" ) OR TITLE-ABS-KEY ( "Fertility Preference" ) OR TITLE-ABS-KEY ( "Preference, Fertility" ) OR TITLE-ABS-KEY ( "Preferences, Fertility" ) OR TITLE-ABS-KEY ( "Fertility, Below Replacement" ) OR TITLE-ABS-KEY ( "Below Replacement Fertility" ) OR TITLE-ABS-KEY ( "Marital Fertility" ) OR TITLE-ABS-KEY ( "Fertility, Marital" ) OR TITLE-ABS-KEY ( "Natural Fertility" ) OR TITLE-ABS-KEY ( "Fertility, Natural" ) OR TITLE-ABS-KEY ( "World Fertility Survey" ) OR TITLE-ABS-KEY ( "Fertility Survey, World" ) OR TITLE-ABS-KEY ( "Fertility Surveys, World" ) OR TITLE-ABS-KEY ( "Survey, World Fertility" ) OR TITLE-ABS-KEY ( "Surveys, World Fertility" ) OR TITLE-ABS-KEY ( "World Fertility Surveys" ) OR TITLE-ABS-KEY ( "Fertility Incentives" ) OR TITLE-ABS-KEY ( "Fertility Incentive" ) OR TITLE-ABS-KEY ( "Obstetrics" ) OR TITLE-ABS-KEY ( "Obstetrical outcomes" ) ) AND ( TITLE-ABS-KEY ( "cohort studies" ) OR TITLE-ABS-KEY ( "case-control studies" ) OR TITLE-ABS-KEY ( "comparative study" ) OR TITLE-ABS-KEY ( "cohort*" ) OR TITLE-ABS-KEY ( "compared" ) OR TITLE-ABS-KEY ( "case control" ) OR TITLE-ABS-KEY ( "case series" ) OR TITLE-ABS-KEY ( "randomized controlled trial" ) OR TITLE-ABS-KEY ( "controlled clinical trial" ) OR TITLE-ABS-KEY ( "randomized" ) OR TITLE-ABS-KEY ( "placebo" ) OR TITLE-ABS-KEY ( "drug therapy" ) OR TITLE-ABS-KEY ( "randomly" ) OR TITLE-ABS-KEY ( "clinical trial" ) ) AND PUBYEAR > 1952 AND PUBYEAR < 2025

7、CNKI-3429

(TKA % 'Malignant Breast Tumor' OR TKA % 'Malignant Breast Cancer' OR TKA % 'Breast Cancer' OR TKA % 'Carcinoma of the Breast' OR TKA % 'Breast Rock' OR TKA % 'Breast Tumor' OR TKA % 'Mammary Tumor') AND (TKA % 'Pregnancy' OR TKA % 'Gestation' OR TKA % 'Fertilization' OR TKA % 'Conception' OR TKA % 'Implantation' OR TKA % 'Childbirth' OR TKA % 'Birth' OR TKA % 'Reproduction' OR TKA % 'Procreation' OR TKA % 'Pregnancy and Childbirth') AND (AB % 'Cohort Study' OR AB % 'Case-Control Study' OR AB % 'Clinical Trial' OR AB % 'Group Study' OR AB % 'Fixed Cohort Study' OR AB % 'Prospective Study' OR AB % 'Retrospective Study' OR AB % 'Randomized Controlled Trial' OR AB % 'Clinical Research')

time: Start year-2024-07-01

8、Wang Fang-2200

(Subject: (Malignant Breast Tumor OR Malignant Breast Cancer OR Breast Cancer OR Carcinoma of the Breast OR Breast Rock OR Breast Tumor OR Mammary Tumor) AND Subject: (Pregnancy OR Gestation OR Fertilization OR Conception OR Implantation OR Childbirth OR Birth OR Reproduction OR Procreation OR Pregnancy and Childbirth) AND Subject: (Cohort Study OR Case-Control Study OR Clinical Trial OR Group Study OR Fixed Cohort Study OR Prospective Study OR Retrospective Study OR Randomized Controlled Trial OR Clinical Research)) time: Start year-2024-07-01

9、VIP-563

U = (Malignant Breast Tumor + Malignant Breast Cancer + Breast Cancer + Carcinoma of the Breast + Breast Rock + Breast Tumor + Mammary Tumor) AND U = (Pregnancy + Gestation + Fertilization + Conception + Implantation + Childbirth + Birth + Reproduction + Procreation + Pregnancy and Childbirth) AND U = (Cohort Study + Case-Control Study + Clinical Trial + Group Study + Fixed Cohort Study + Prospective Study + Retrospective Study + Randomized Controlled Trial + Clinical Research)

time: Start year-Present, search date:2024-07-01

10、CBM-384

1. "Breast Tumor" [Unweighted: Extended] 116,019
2. ("Malignant Breast Tumor" [Common Fields: Smart] OR "Malignant Breast Cancer" [Common Fields: Smart] OR "Breast Cancer" [Common Fields: Smart] OR "Carcinoma of the Breast" [Common Fields: Smart] OR "Breast Rock" [Common Fields: Smart] OR "Breast Tumor" [Common Fields: Smart] OR "Mammary Tumor" [Common Fields: Smart]) 138,857
3. (#2) OR (#1) 138,857
4. "Pregnancy" [Unweighted: Extended] 123,359
5. "Fertilization" [Unweighted: Extended] 3,472
6. "Childbirth" [Unweighted: Extended] 62,781
7. "Fertility" [Unweighted: Extended] 8,157
8. ("Pregnancy" [Common Fields: Smart] OR "Gestation" [Common Fields: Smart] OR "Fertilization" [Common Fields: Smart] OR "Conception" [Common Fields: Smart] OR "Implantation" [Common Fields: Smart] OR "Childbirth" [Common Fields: Smart] OR "Birth" [Common Fields: Smart] OR "Reproduction" [Common Fields: Smart] OR "Procreation" [Common Fields: Smart] OR "Pregnancy and Childbirth" [Common Fields: Smart]) 939,370
9. (#8) OR (#7) OR (#6) OR (#5) OR (#4) 940,558
10. "Cohort Study" [Unweighted: Extended] 242,490
11. "Case-Control Study" [Unweighted: Extended] 138,955
12. "Clinical Trial" [Unweighted: Extended] 208,020
13. "Prospective Study" [Unweighted: Extended] 11,807
14. "Retrospective Study" [Unweighted: Extended] 121,194
15. "Randomized Controlled Trial" [Unweighted: Extended] 197,991
16. "Clinical Research" [Unweighted: Extended] 208,564
17. ("Cohort Study" [Common Fields: Smart] OR "Case-Control Study" [Common Fields: Smart] OR "Clinical Trial" [Common Fields: Smart] OR "Group Study" [Common Fields: Smart] OR "Fixed Cohort Study" OR "Prospective Study" [Common Fields: Smart] OR "Retrospective Study" [Common Fields: Smart] OR "Randomized Controlled" [Common Fields: Smart] OR "Clinical Research" [Common Fields: Smart]) 800,892
18. (#17) OR (#16) OR (#15) OR (#14) OR (#13) OR (#12) OR (#11) OR (#10) 800,892

19) (((#18) AND (#9) AND (#3)) AND Date Range 1900-2024) 384
