# Supplementary material for: Cancer outcomes of pregnancy after diagnosis of breast cancer in premenopausal women: an updated systematic review and meta-analysis
Source: Front Oncol. 2025 Oct 14;15:1644566. doi: 10.3389/fonc.2025.1644566 (PMC12558755; doi:10.3389/fonc.2025.1644566)
Supplement: Supplementary file 2 [file DataSheet2.docx]

**Appendix B Quality evaluation**

Table B.1 Quality evaluation of cohort studies

| **First-Author** | **Year of publication** | **Selection** | **Comparability** | **Exposure** | **Total score** |
| --- | --- | --- | --- | --- | --- |
| Tomohiro Ochi | 2023 | 3 | 2 | 3 | 8 |
| DU Yanze | 2016 | 3 | 1 | 2 | 6 |
| Risto Sankila | 1994 | 4 | 1 | 1 | 6 |
| By Eva von Schoultz | 1995 | 4 | 1 | 1 | 6 |
| Niels Kroman | 1998 | 4 | 2 | 1 | 7 |
| Priscilla Velentgas | 1999 | 4 | 1 | 2 | 7 |
| By Shari Gelber | 2001 | 2 | 1 | 1 | 4 |
| Beth A. Mueller | 2003 | 4 | 2 | 2 | 8 |
| L. Johnetta Blakely | 2004 | 3 | 1 | 1 | 5 |
| LI Fei | 2017 | 3 | 0 | 2 | 5 |
| M. Al Khaduri | 2017 | 3 | 1 | 1 | 5 |
| Lauren Nye | 2017 | 4 | 1 | 1 | 6 |
| N. Sinha | 2017 | 2 | 0 | 2 | 4 |
| Niels Kroman | 2008 | 4 | 2 | 1 | 7 |
| J. Y. Wu | 2009 | 3 | 1 | 1 | 5 |
| H. M. Verkooijen | 2010 | 4 | 2 | 2 | 8 |
| MIHONG CHOI | 2019 | 4 | 2 | 2 | 8 |
| LUO Sheng | 2019 | 3 | 1 | 2 | 6 |
| E. Rosenberg | 2019 | 4 | 1 | 2 | 7 |
| Matteo Lambertini | 2019 | 2 | 1 | 2 | 5 |
| Octavi Córdoba | 2012 | 4 | 1 | 2 | 7 |
| Robin J. Bell | 2013 | 3 | 1 | 2 | 6 |
| Yang Li | 2021 | 3 | 2 | 3 | 8 |
| Hatem A. Azim Jr | 2012 | 3 | 2 | 3 | 8 |
| Richard A. Anderson | 2022 | 4 | 2 | 2 | 8 |
| Soo Youn Bae | 2022 | 4 | 2 | 2 | 8 |
| Minsun Kang | 2022 | 4 | 2 | 2 | 8 |
| J. Alejandro Rauh-Hain | 2022 | 4 | 2 | 2 | 8 |
| SHU-CHUN CHUANG | 2020 | 4 | 2 | 2 | 8 |
| Matteo Lambertini | 2020 | 3 | 2 | 2 | 7 |
| Moo Hyun Lee | 2020 | 4 | 1 | 3 | 8 |
| Oranite Goldrat | 2015 | 4 | 1 | 2 | 7 |
| Angelena Crown | 2022 | 3 | 1 | 1 | 5 |
| Mary Kathryn Abel | 2021 | 3 | 1 | 2 | 6 |
| Matteo Lambertini | 2024 | 4 | 1 | 2 | 7 |
| Matteo Lambertini | 2024 | 4 | 2 | 3 | 9 |
| Young-jin Lee | 2024 | 2 | 1 | 1 | 4 |
| Ji Hye Kim | 2024 | 3 | 1 | 2 | 6 |

Table B.2 Quality evaluation of case-control studies

| **First-Author** | **Year of publication** | **selection** | **comparability** | **exposure** | **Total score** |
| --- | --- | --- | --- | --- | --- |
| Nicholaos A. Malamos | 1996 | 2 | 1 | 1 | 4 |
| Adriana Valentini | 2013 | 3 | 1 | 2 | 6 |
| Matteo Lambertini | 2018 | 3 | 2 | 2 | 7 |

| Table B.3 Quality evaluation of case series | | | | | | | | | |
| --- | --- | --- | --- | --- | --- | --- | --- | --- | --- |
| **Items** | Pentti M. Rissanen  2009 | Anne-Sophie Hamy  2016 | REGINA SUTTON  1990 | JAMES C. HARVEY  1981 | R. M. Clark  1989 | T. Kojouh-arova  2008 | STUART W.  HARRINGTON  1937 | Anne E. LETHABY  1996 | Hideyuki Iwahata  2023 |
| Were there clear criteria for inclusion in the case series? | Yes | Yes | Yes | Yes | Yes | Yes | No | Yes | Yes |
| Was the condition measured in a standard, reliable way for all participants included in the case series? | Unclear | Yes | Unclear | Unclear | Unclear | Unclear | Unclear | Yes | Unclear |
| Were valid methods used for identification of the condition for all participants included in the case series? | Unclear | Yes | Unclear | Unclear | Unclear | Unclear | Unclear | Yes | Unclear |
| Did the case series have consecutive inclusion of participants? | Unclear | Yes | Unclear | Unclear | Unclear | Unclear | Unclear | Unclear | Unclear |
| Did the case series have complete inclusion of participants? | No | No | No | No | No | No | No | Yes | No |
| Was there clear reporting of the demographics of the participants in the study? | No | No | No | No | No | No | No | No | No |
| Was there clear reporting of clinical information of the participants? | Yes | Yes | Yes | Yes | Yes | Yes | Yes | Yes | No |
| Were the outcomes or follow up results of cases clearly reported? | Yes | Yes | Yes | Yes | Yes | Yes | Yes | Yes | Yes |
| Was there clear reporting of the presenting site(s)/clinic(s) demographic information? | No | No | Yes | No | Yes | Yes | No | Yes | No |
| Was statistical analysis appropriate? | Yes | Yes | Yes | Yes | Yes | Yes | Yes | Yes | Yes |

Table B.4 Quality evaluation of non-randomized clinical trials

| Items | Ann H. Partridge  2023 | Hatem A. Azim Jr2024 |
| --- | --- | --- |
| 1. Clearly stated research aim | 2 | 2 |
| 2. Coherence in patient inclusion | 2 | 2 |
| 3. Anticipated data collection | 2 | 2 |
| 4. Endpoint indicators appropriately reflecting the research aim | 2 | 2 |
| 5. Objectivity in the evaluation of endpoint indicators | 2 | 2 |
| 6. Adequacy of follow-up duration | 1 | 1 |
| 7. Loss to follow-up rate below 5% | 1 | 1 |
| 8. Estimation of sample size | 2 | 2 |
| 9. Appropriateness of control group selection | 2 | 2 |
| 10. Synchronization of control group with the experimental group | 1 | 1 |
| 11. Comparability of baseline characteristics between groups | 2 | 2 |
| 12. Appropriateness of statistical analysis | 2 | 2 |
| Total | 21 | 21 |
