# Supplementary material for: Cancer outcomes of pregnancy after diagnosis of breast cancer in premenopausal women: an updated systematic review and meta-analysis
Source: Front Oncol. 2025 Oct 14;15:1644566. doi: 10.3389/fonc.2025.1644566 (PMC12558755; doi:10.3389/fonc.2025.1644566)
Supplement: Supplementary file 3 [file DataSheet3.doc]

**Appendix C Forest diagrams, funnel diagrams and sensitivity analysis diagrams**


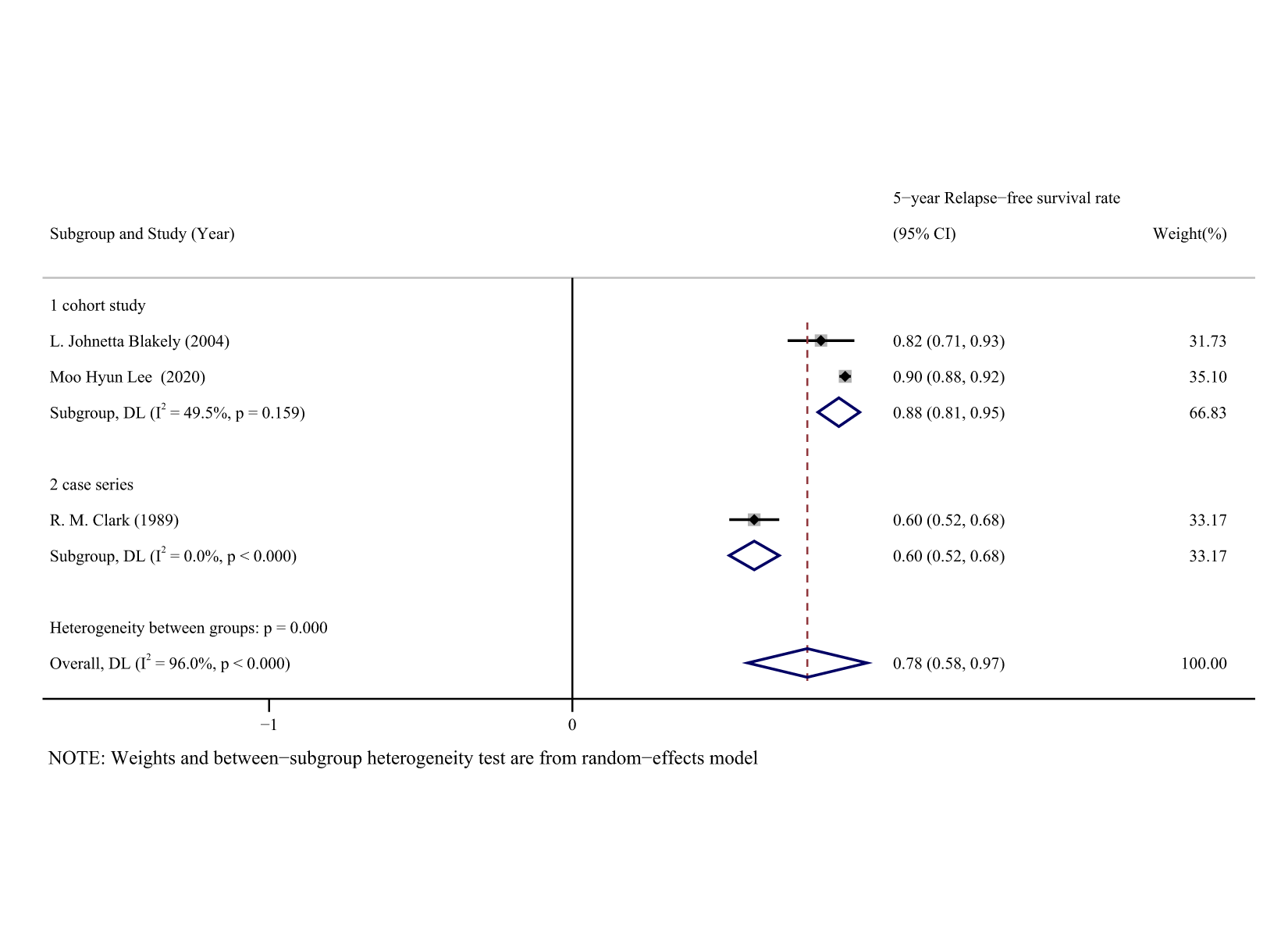


Figure C.1 Forest plot of 5-year relapse-free survival rate in pregnant BC patients


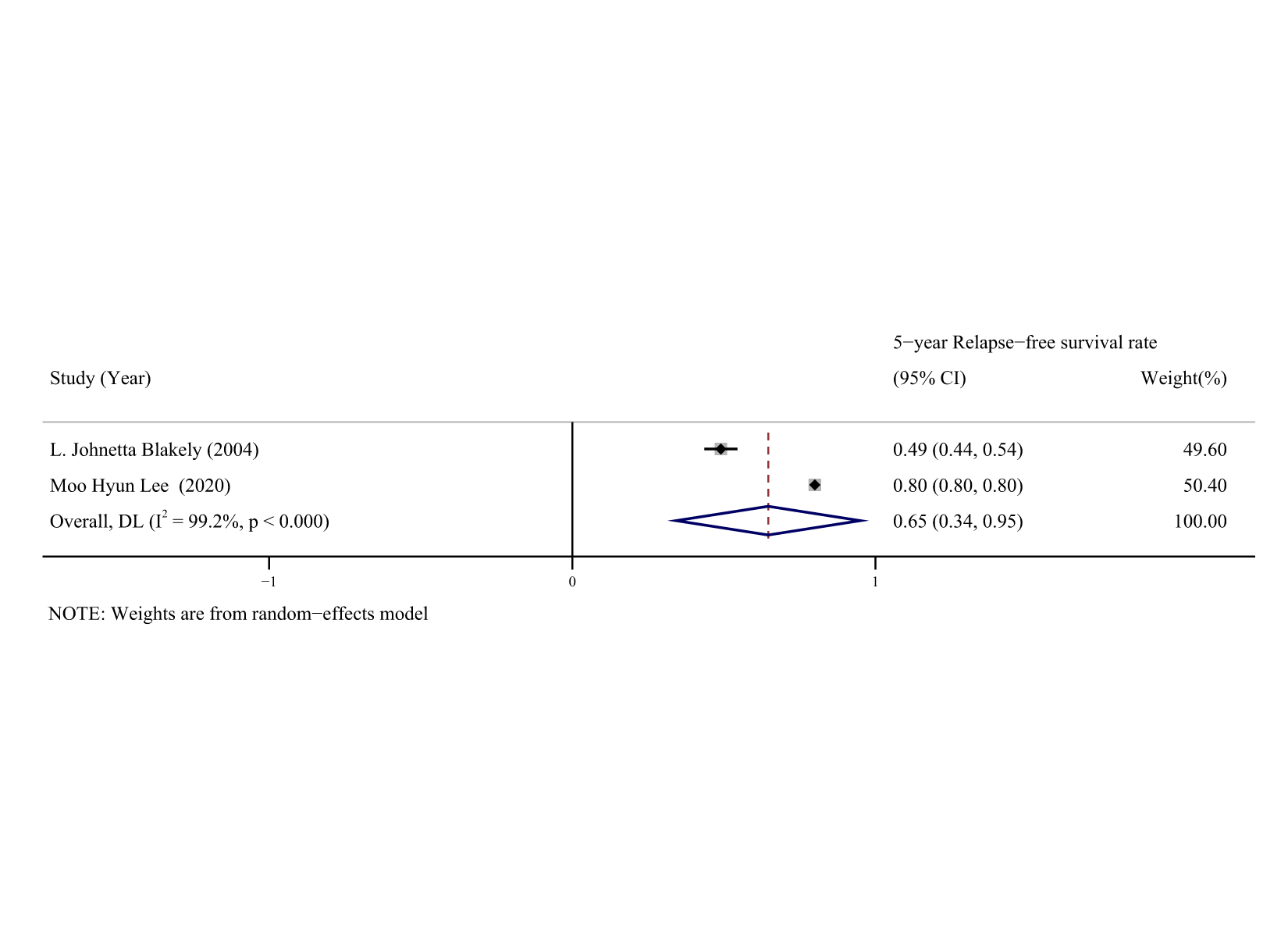


Figure C.2 Forest plot of 5-year relapse-free survival rate in non-pregnant BC patients


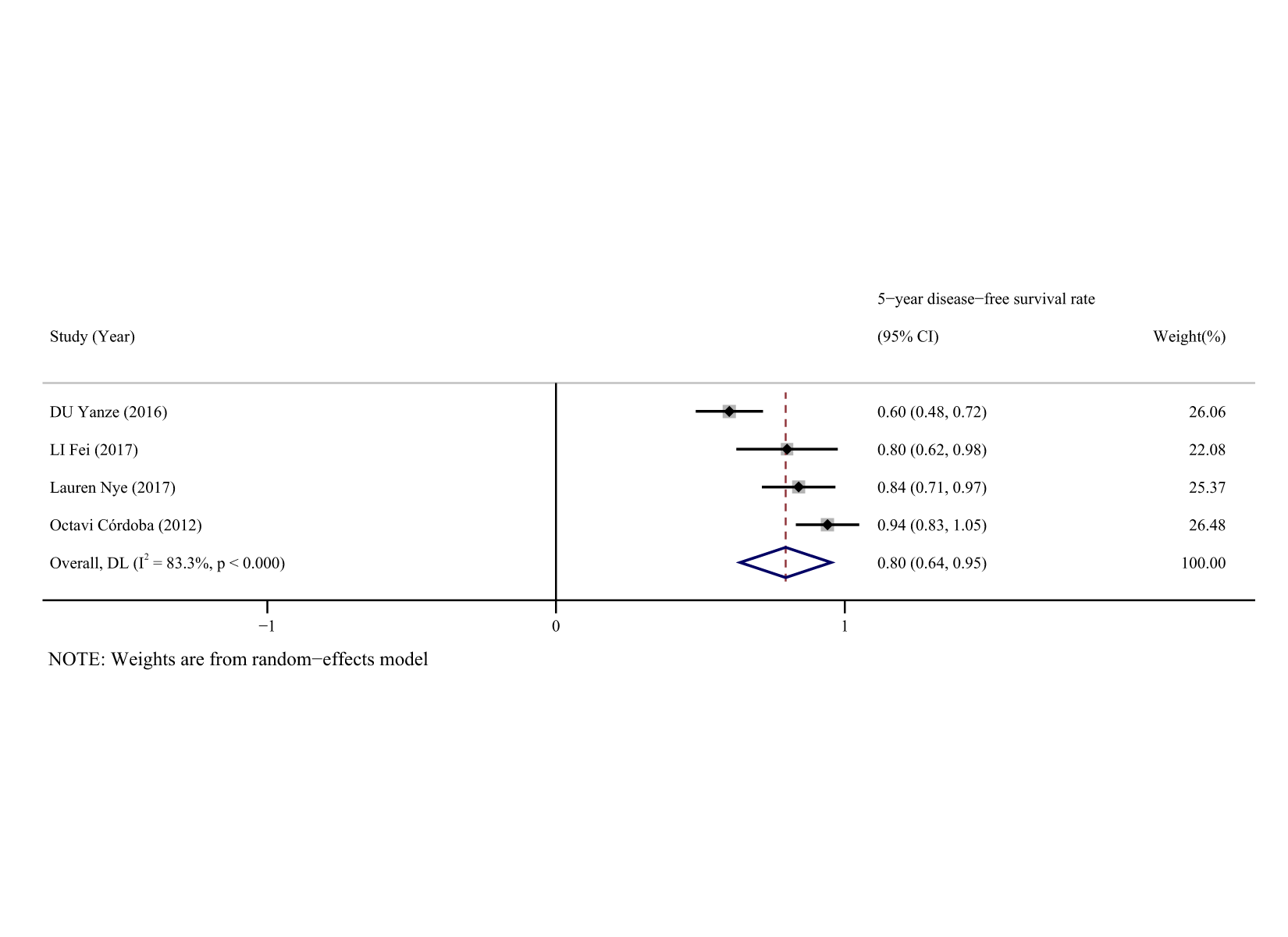


Figure C.3 Forest plot of 5-year disease-free survival rate in pregnant BC patients


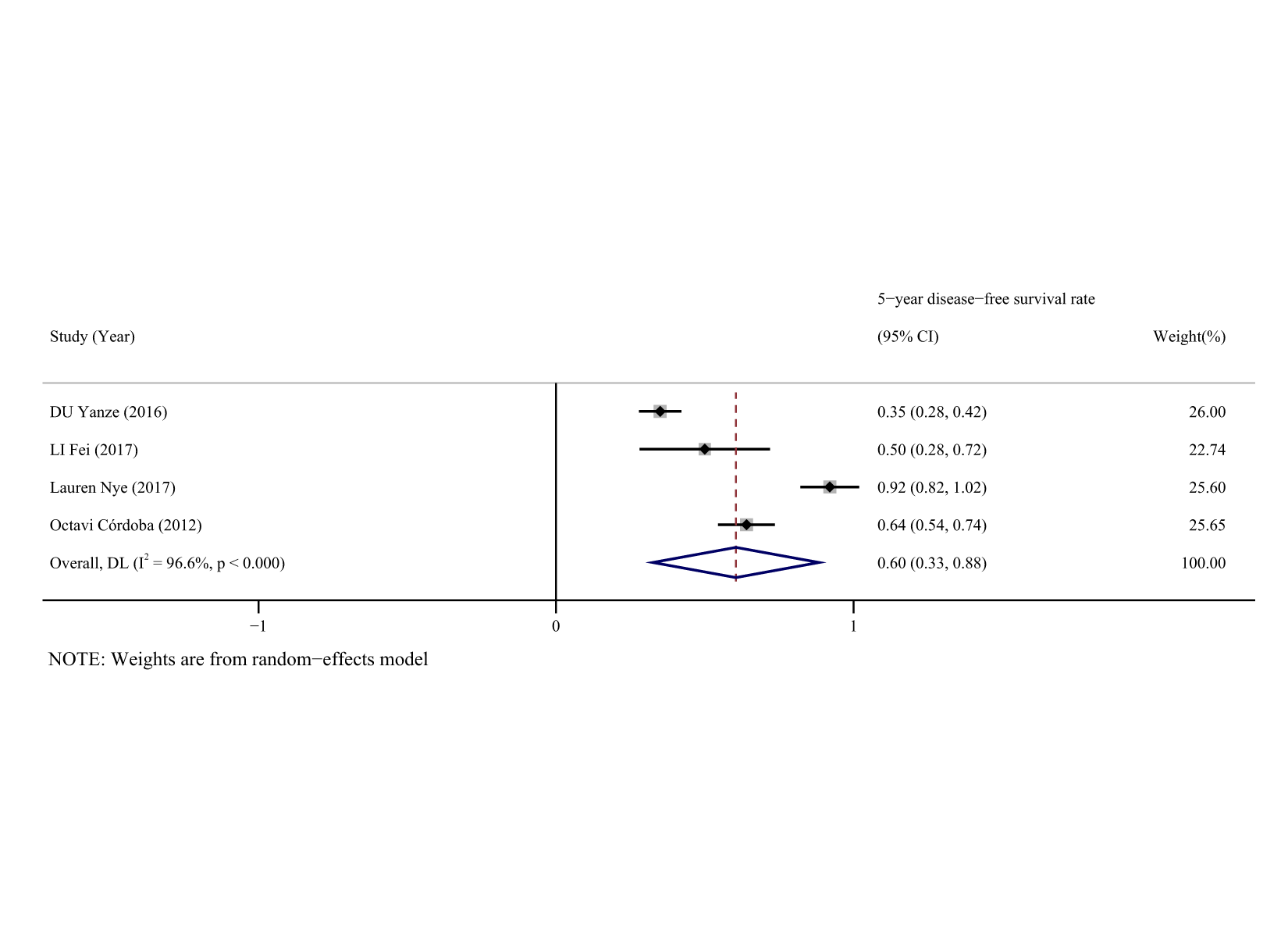


Figure C.4 Forest plot of 5-year disease-free survival rate in non-pregnant BC patients


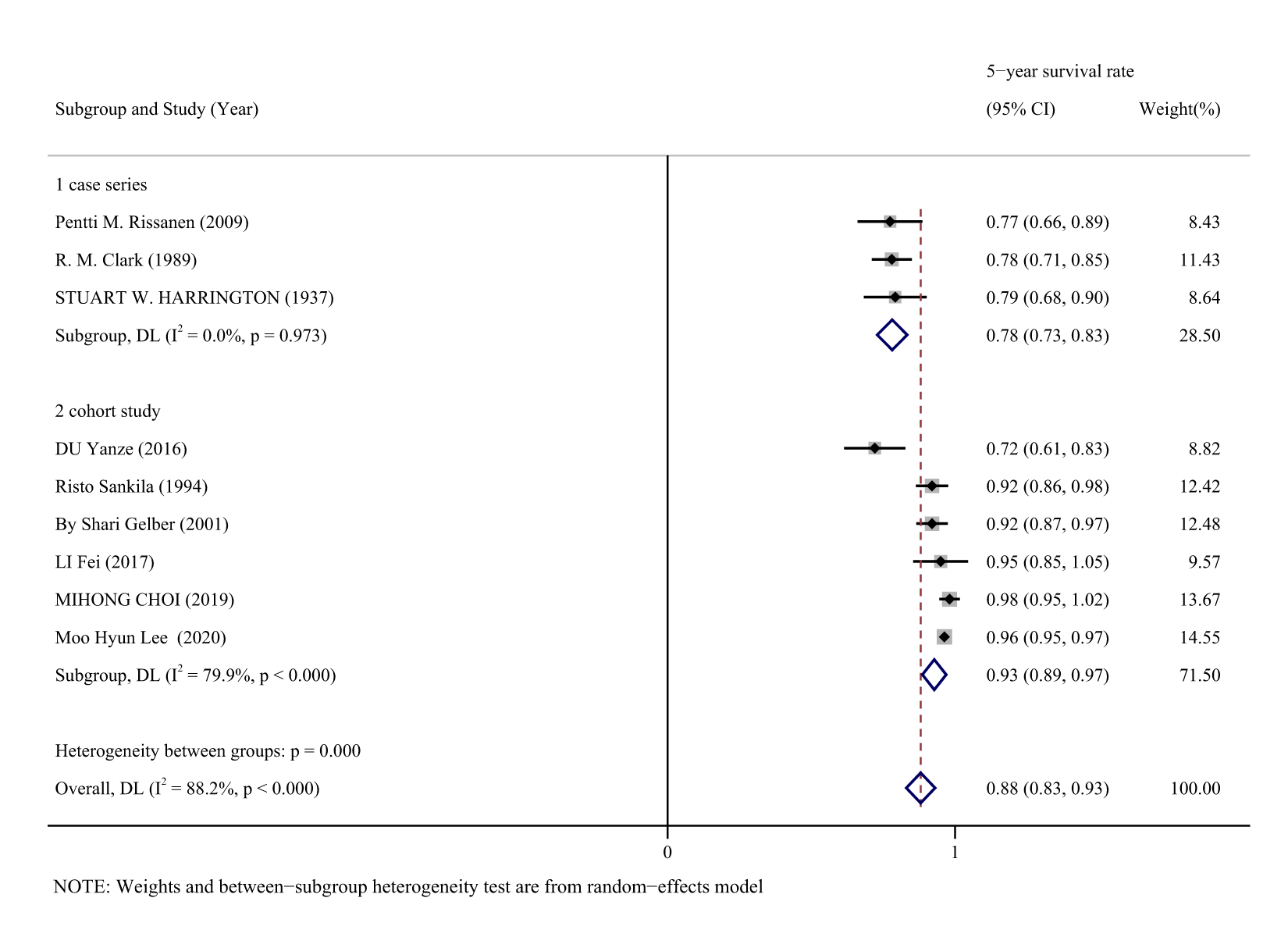


Figure C.5 Forest plot of 5-year survival rate in pregnant BC patients


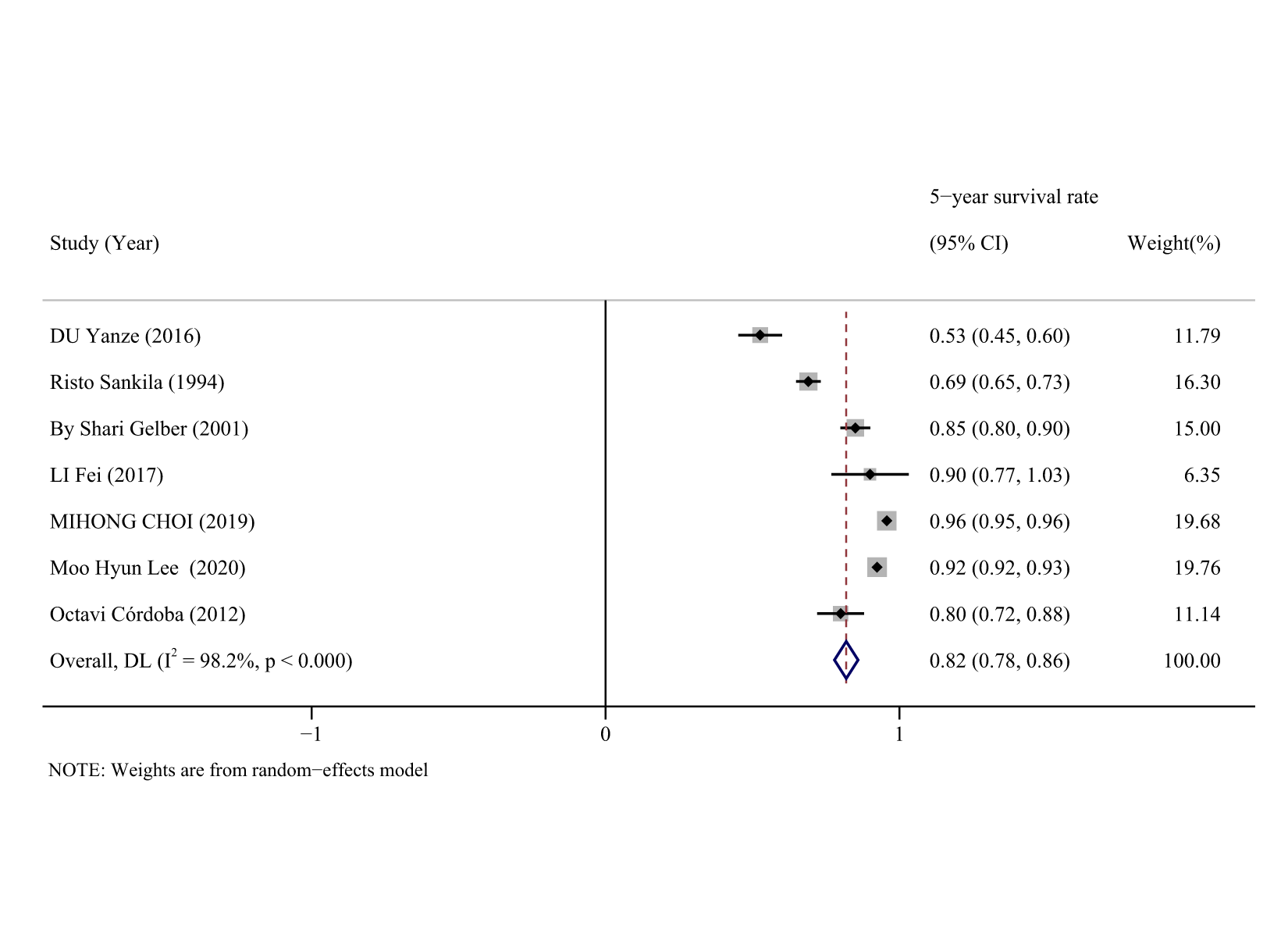


Figure C.6 Forest plot of 5-year survival in the non-pregnant BC patients

**
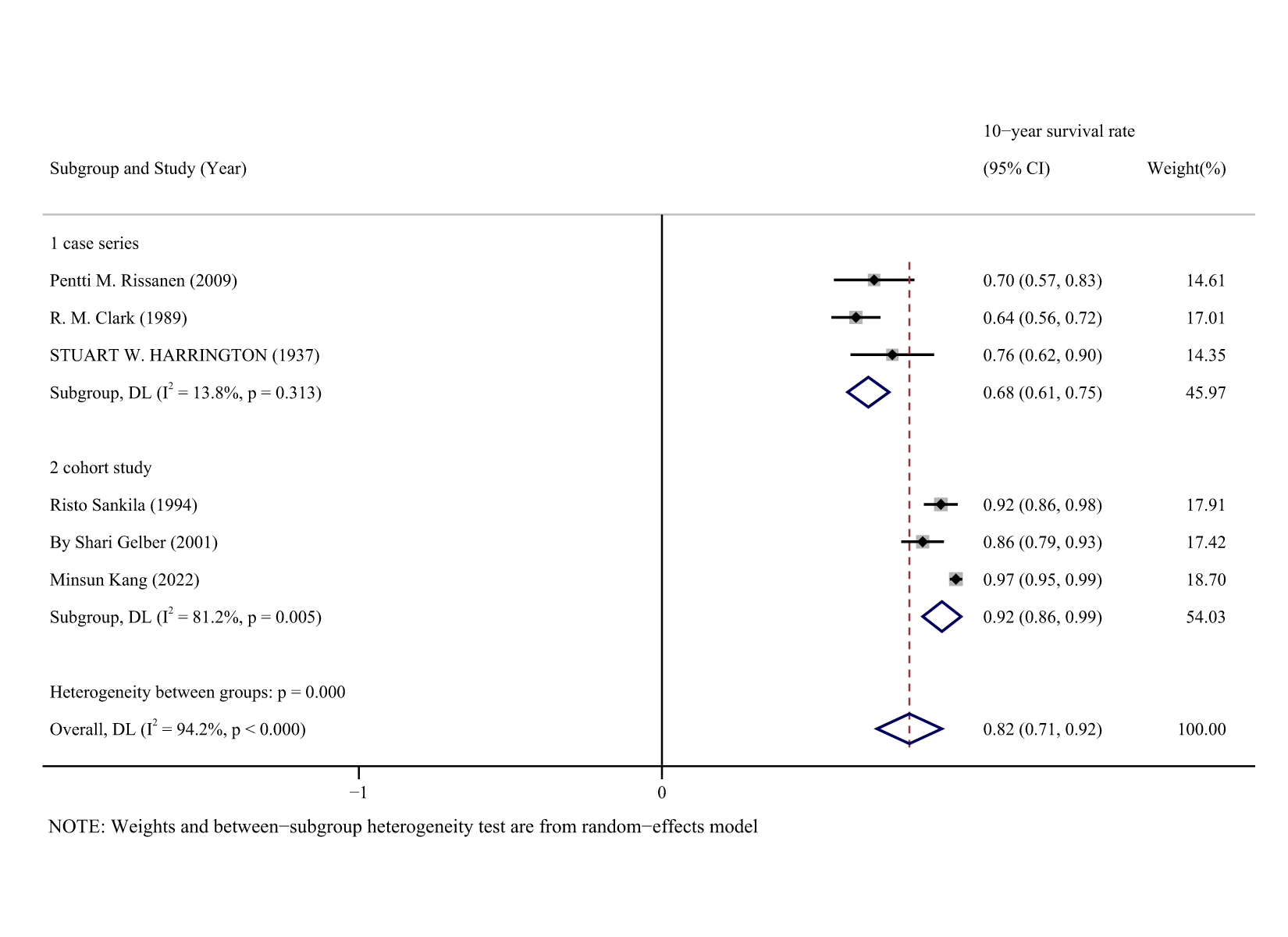
**

Figure C.7 Forest plot of 10-year survival in pregnant BC patients


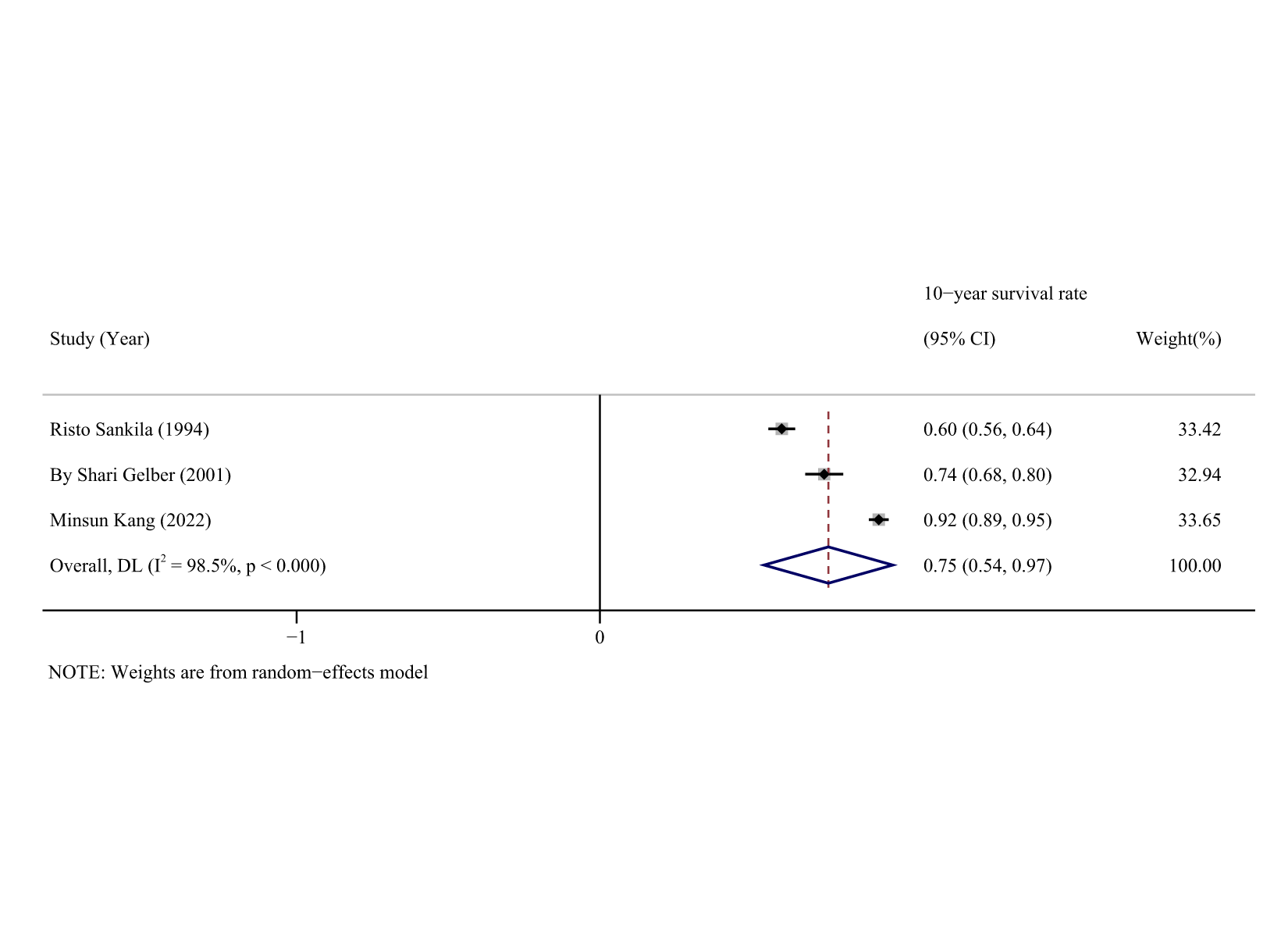


Figure C.8 Forest plot of 10-year survival in non-pregnant BC patients


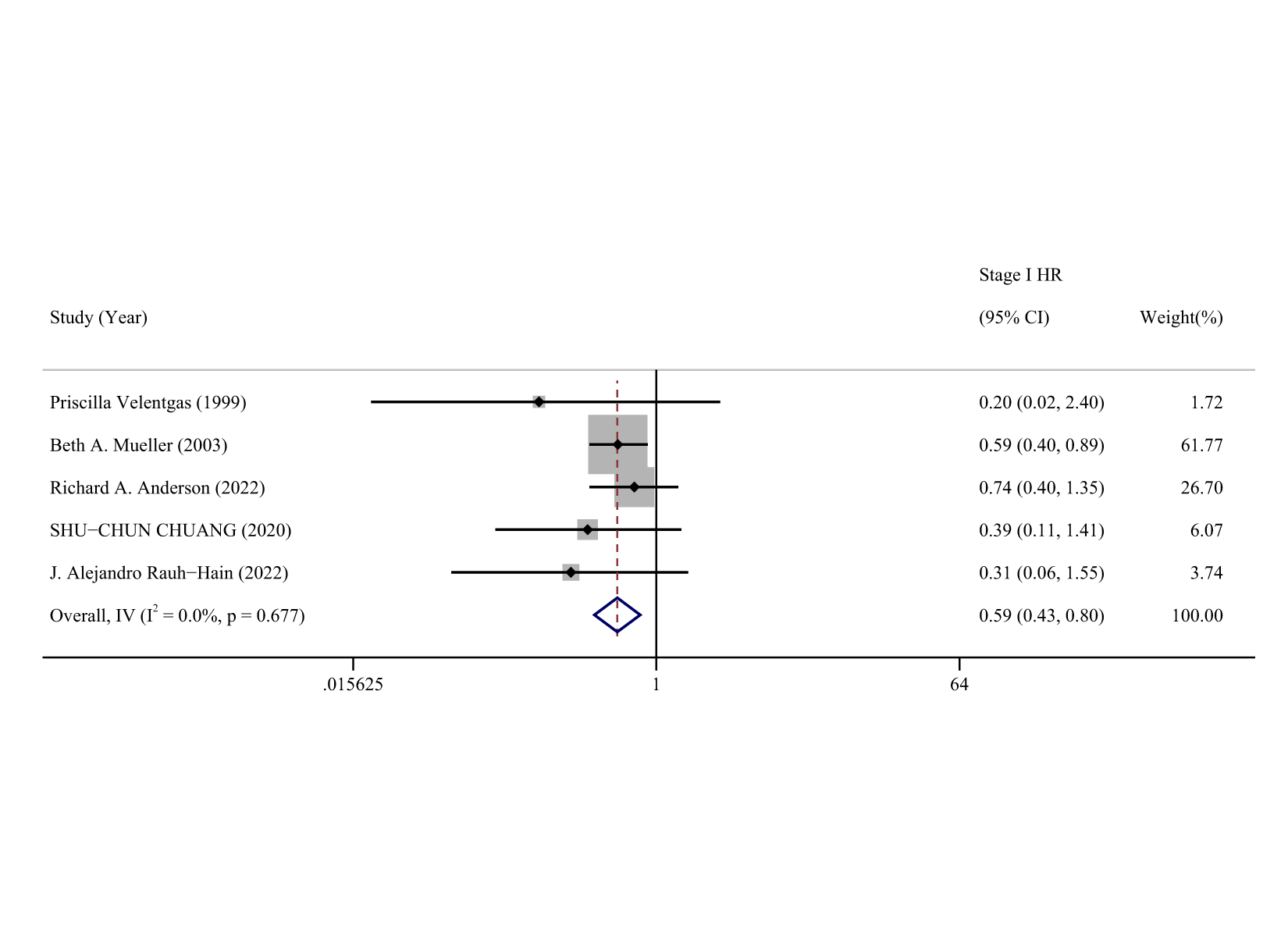
 Figure C.9 Forest plot of overall survival in stage Ⅰ pregnant BC patients compared with non-pregnant BC patients by HR (hazard ratio, HR)


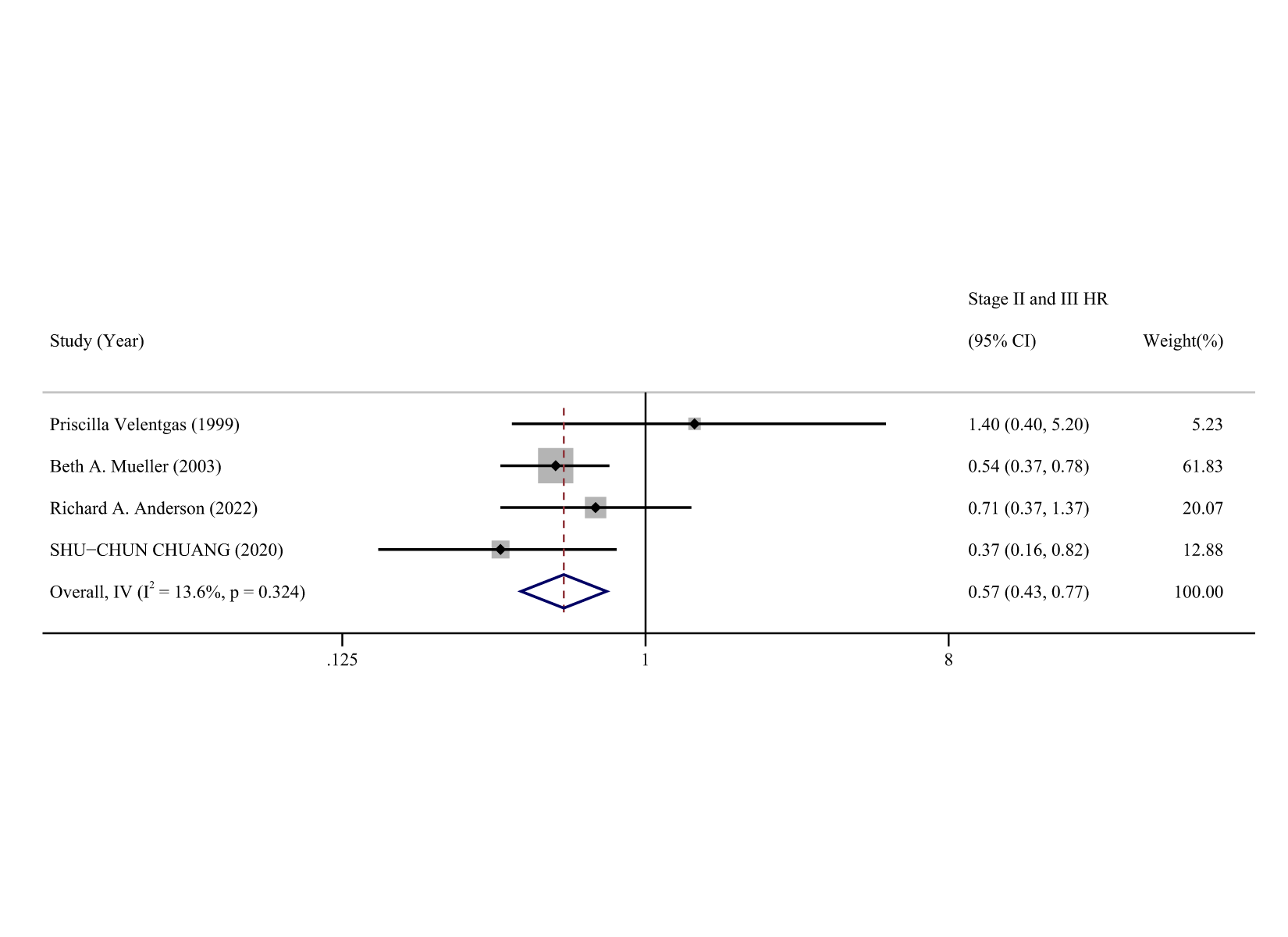


Figure C.10 Forest plot of overall survival in stage II and III pregnant BC patients compared with non-pregnant BC patients by HR (hazard ratio, HR)


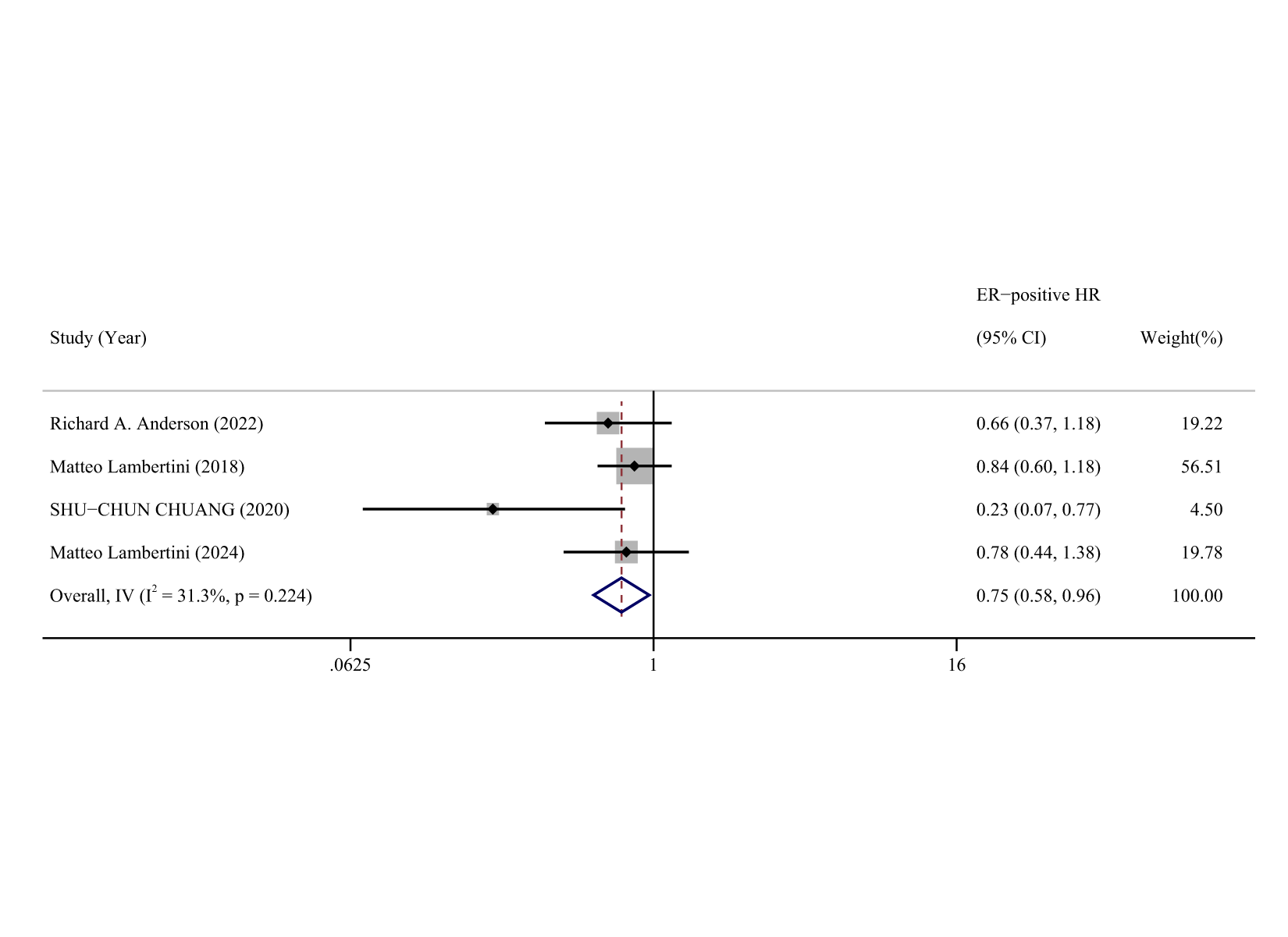


Figure C.11 Forest plot of overall survival in ER-positive pregnant BC patients compared with non-pregnant BC patients by HR (hazard ratio, HR)


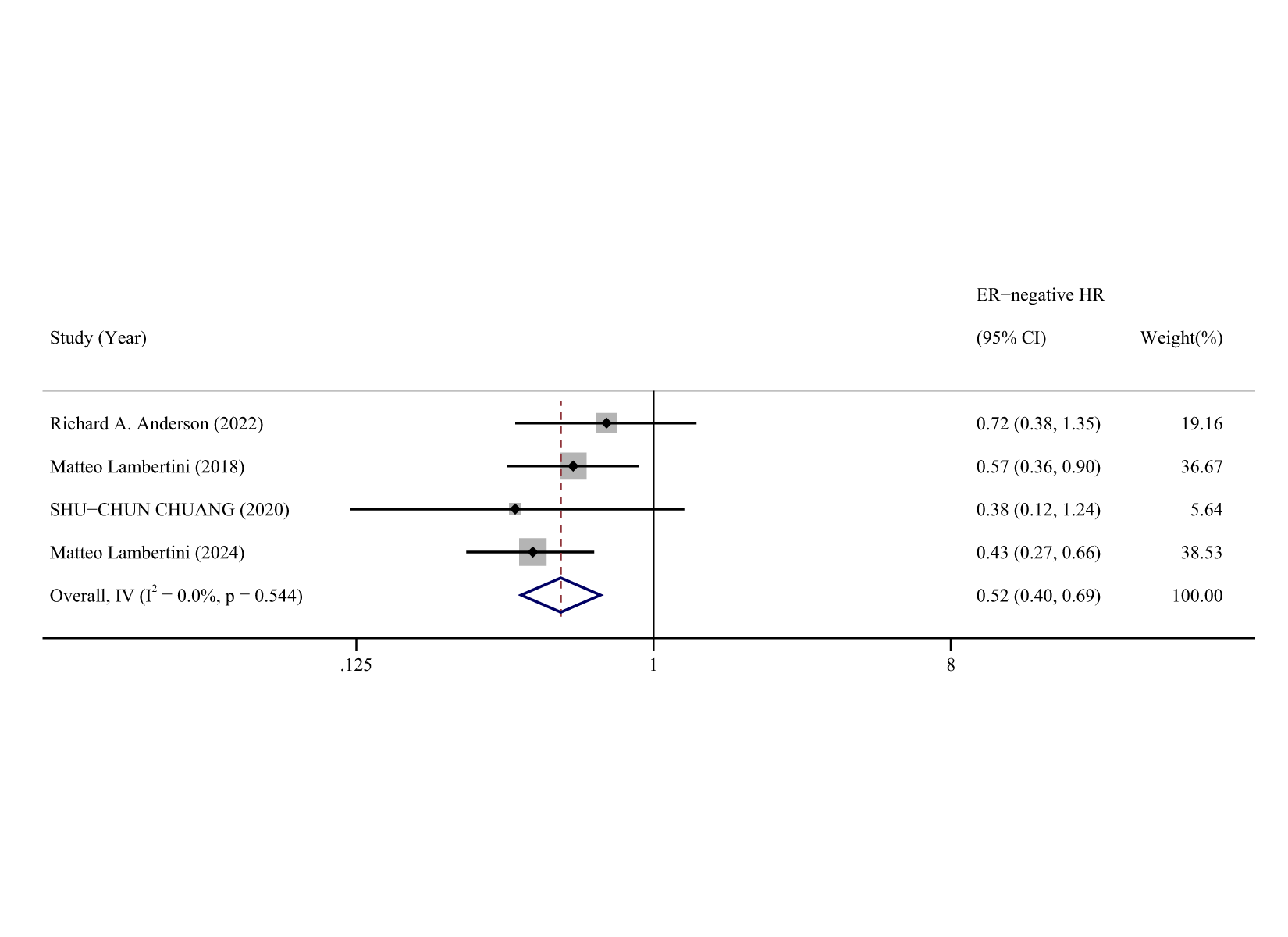


Figure C.12 Forest plot of overall survival in ER-negative pregnant BC patients compared with non-pregnant BC patients by HR (hazard ratio, HR)


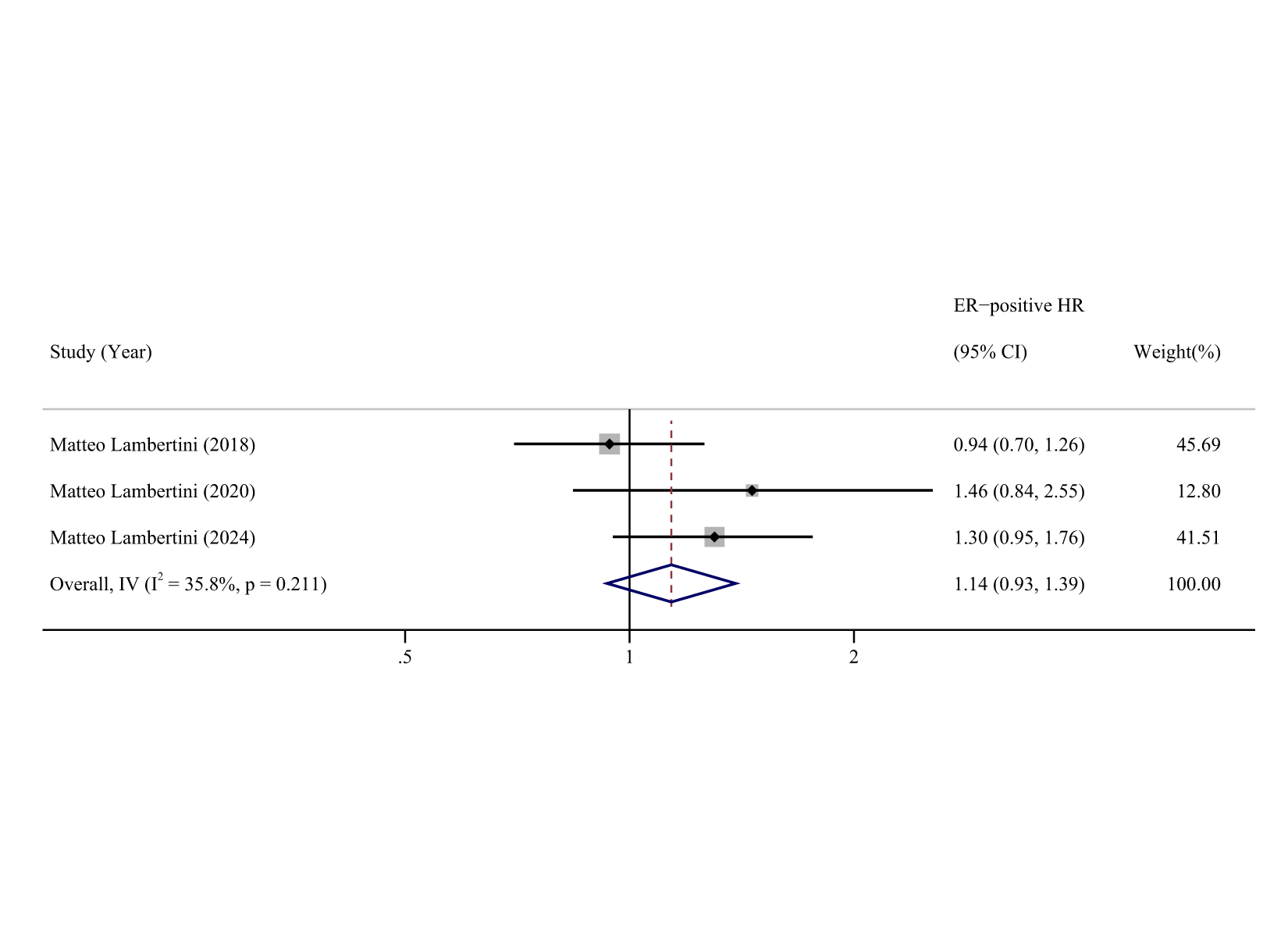


Figure C.13 Forest plot of disease-free survival in ER-positive pregnant BC patients compared with non-pregnant BC patients by HR (hazard ratio, HR)


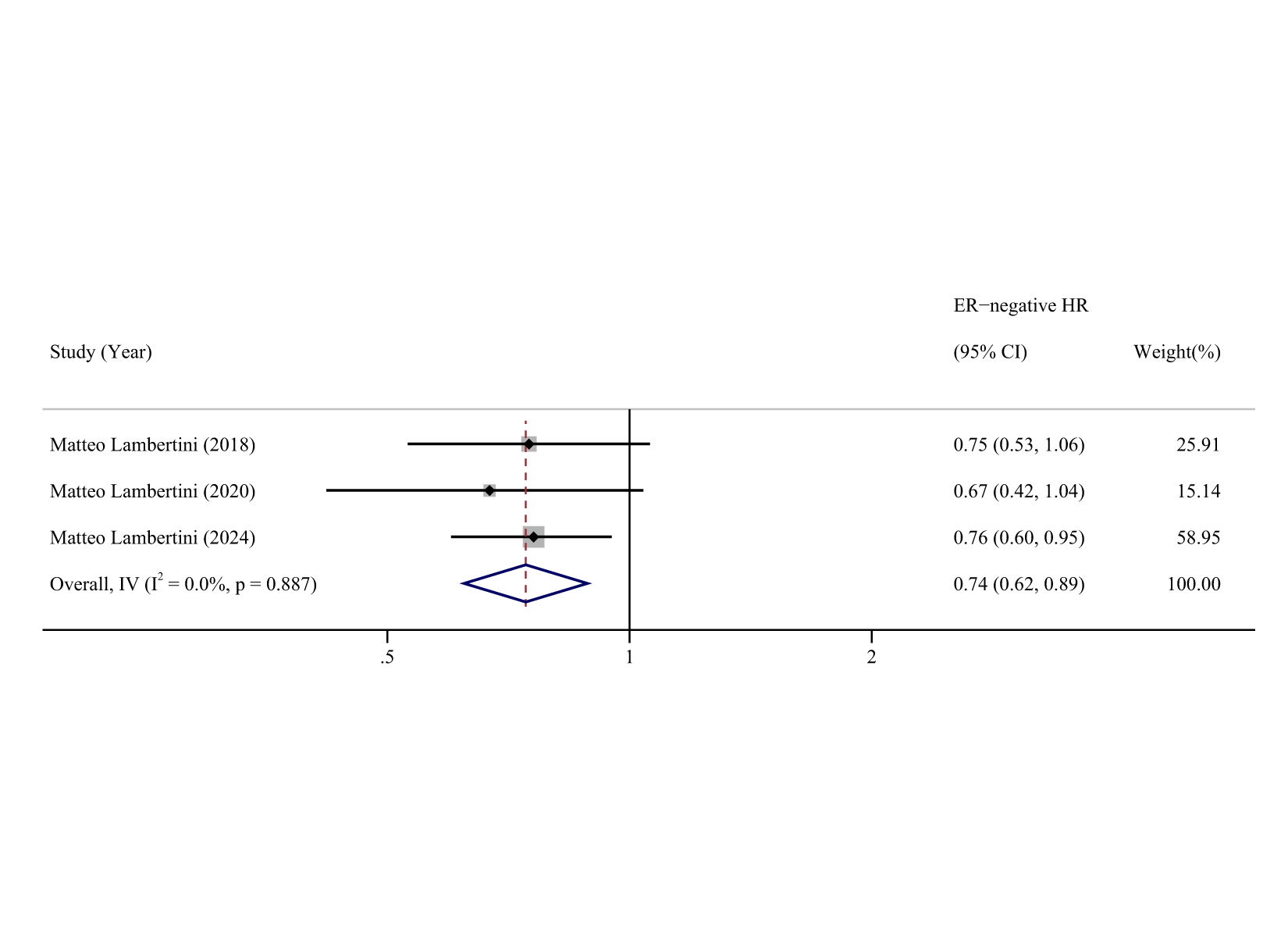


Figure C.14 Forest plot of disease-free survival in ER-negative pregnant BC patients compared with non-pregnant BC patients by HR (hazard ratio, HR)


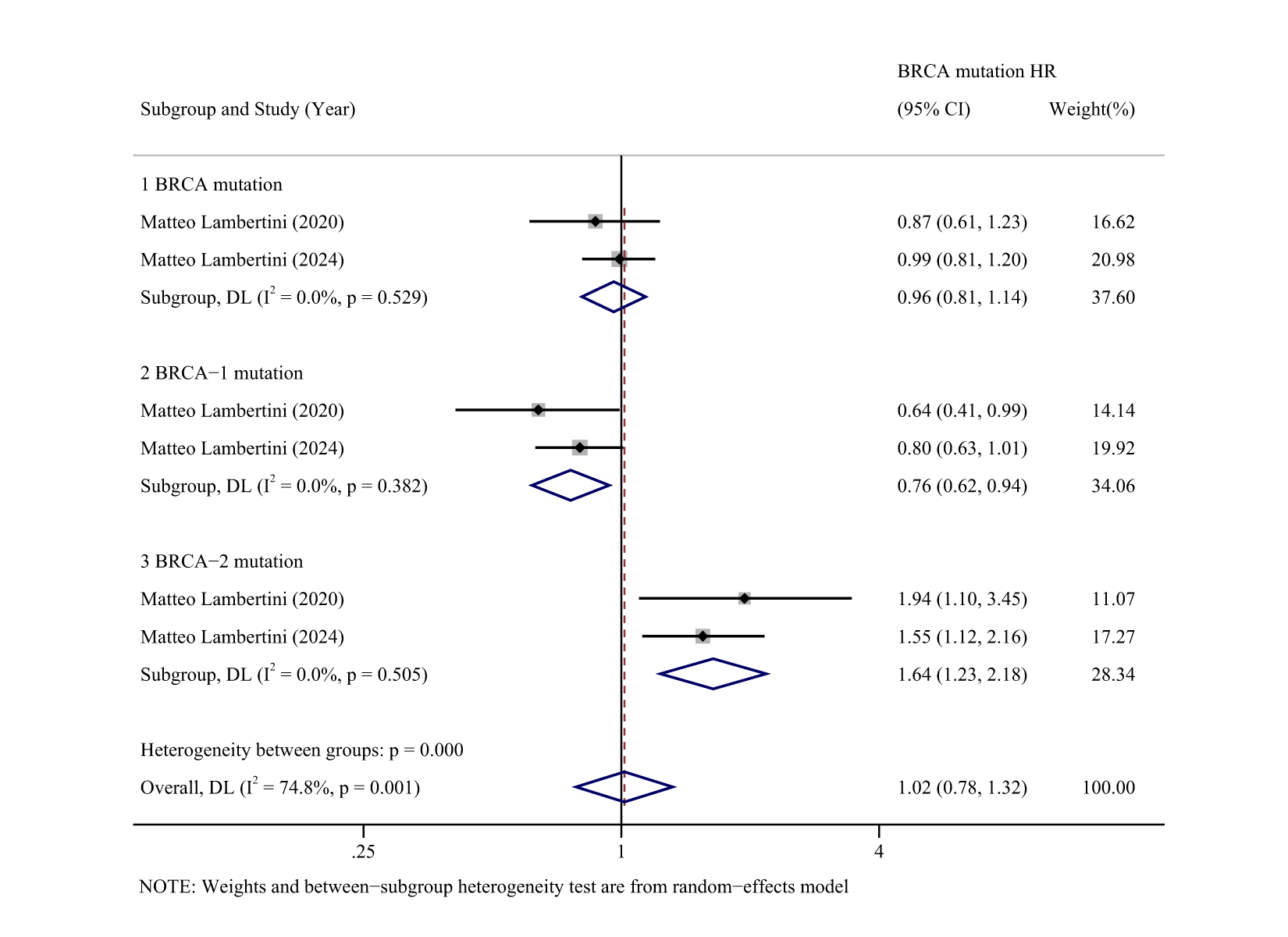


Figure C.15 Forest plot of disease-free survival in BRCA mutation pregnant BC patients compared with non-pregnant BC patients by HR (hazard ratio, HR)


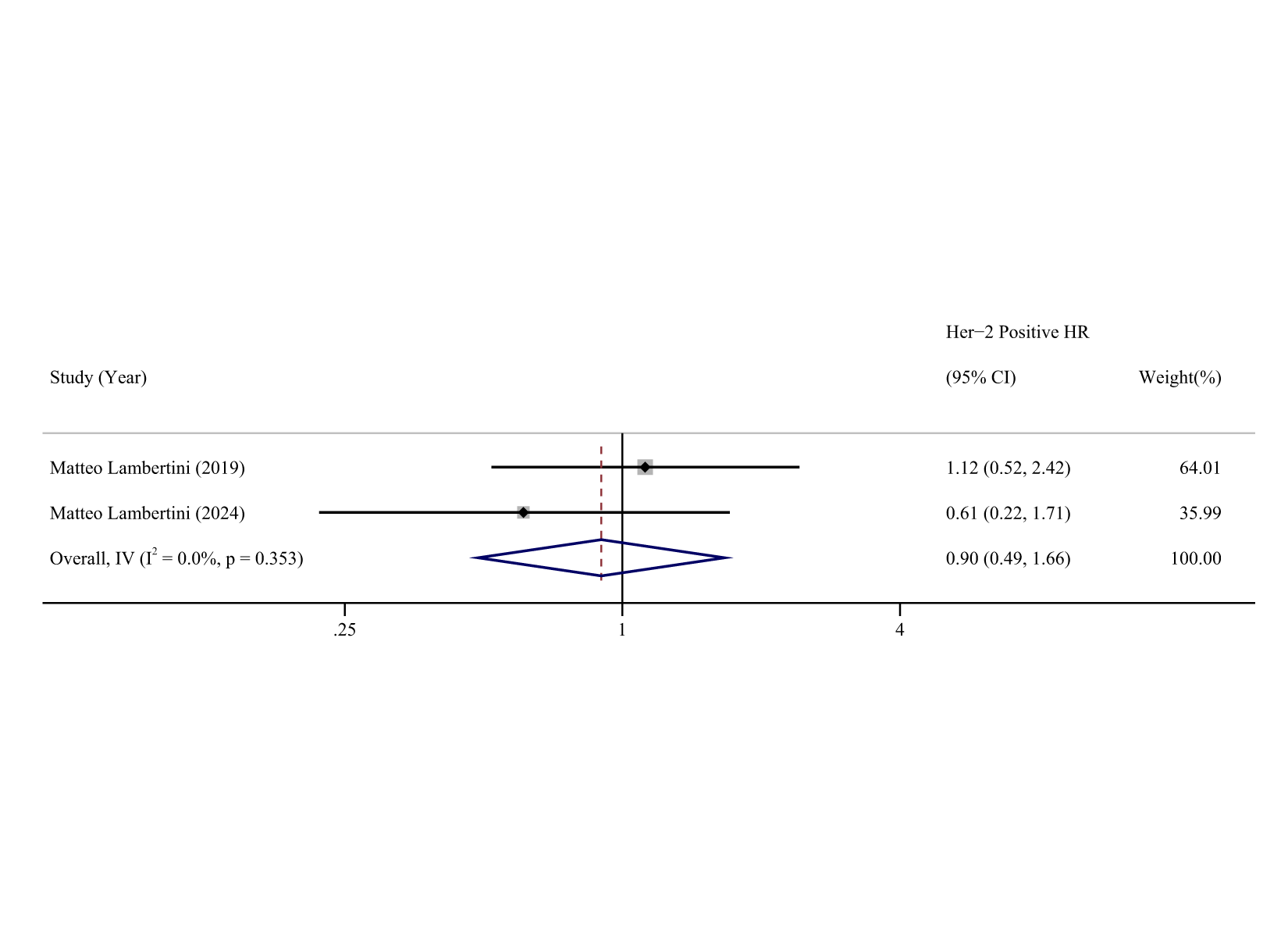


Figure C.16 Forest plot of disease-free survival in HER-2 positive pregnant BC patients compared with non-pregnant BC patients by HR (hazard ratio, HR)
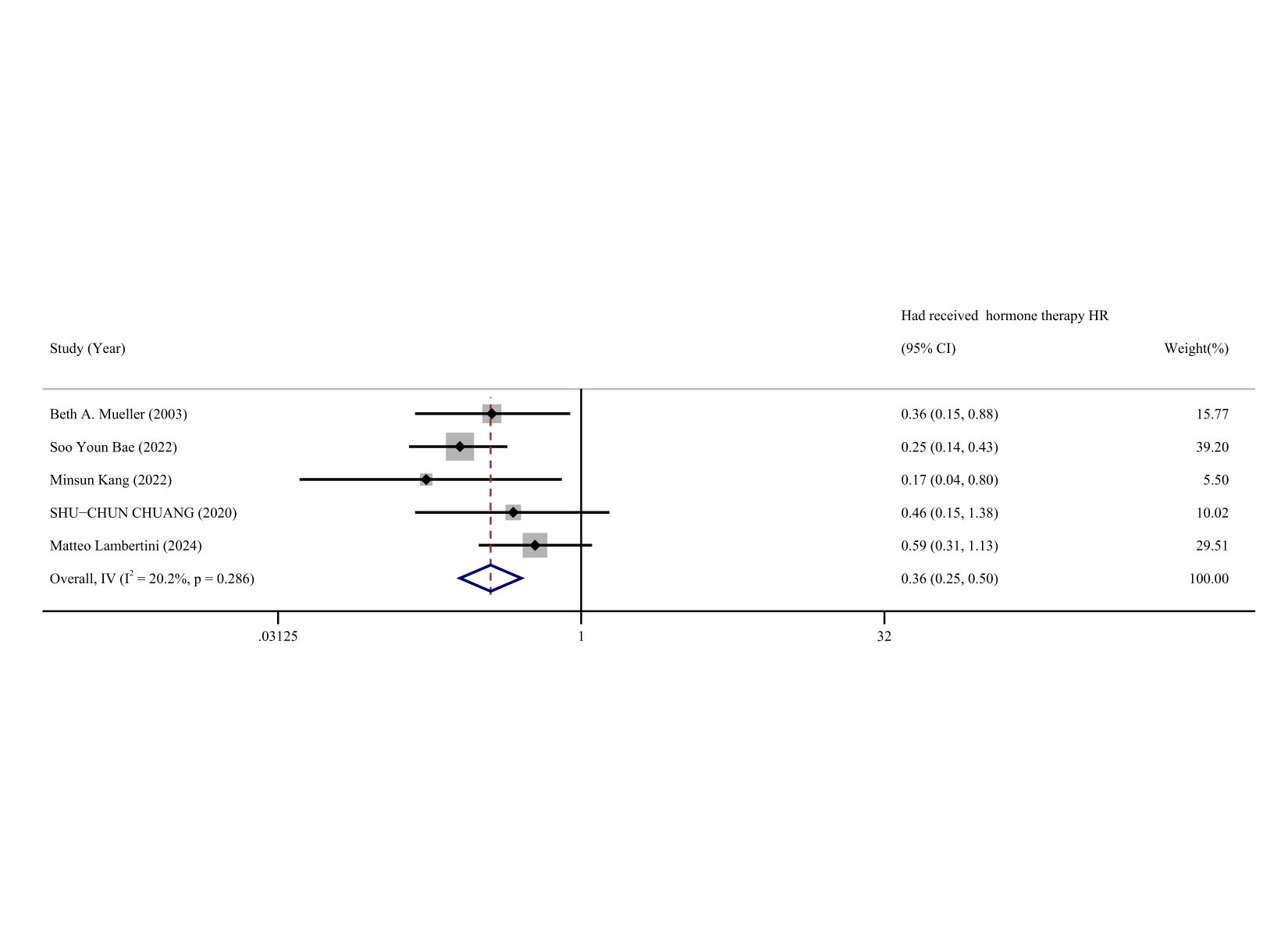


Figure C.17 Forest plot of overall survival in pregnant BC patients who had received hormone therapy compared with non-pregnant BC patients by HR (hazard ratio, HR)


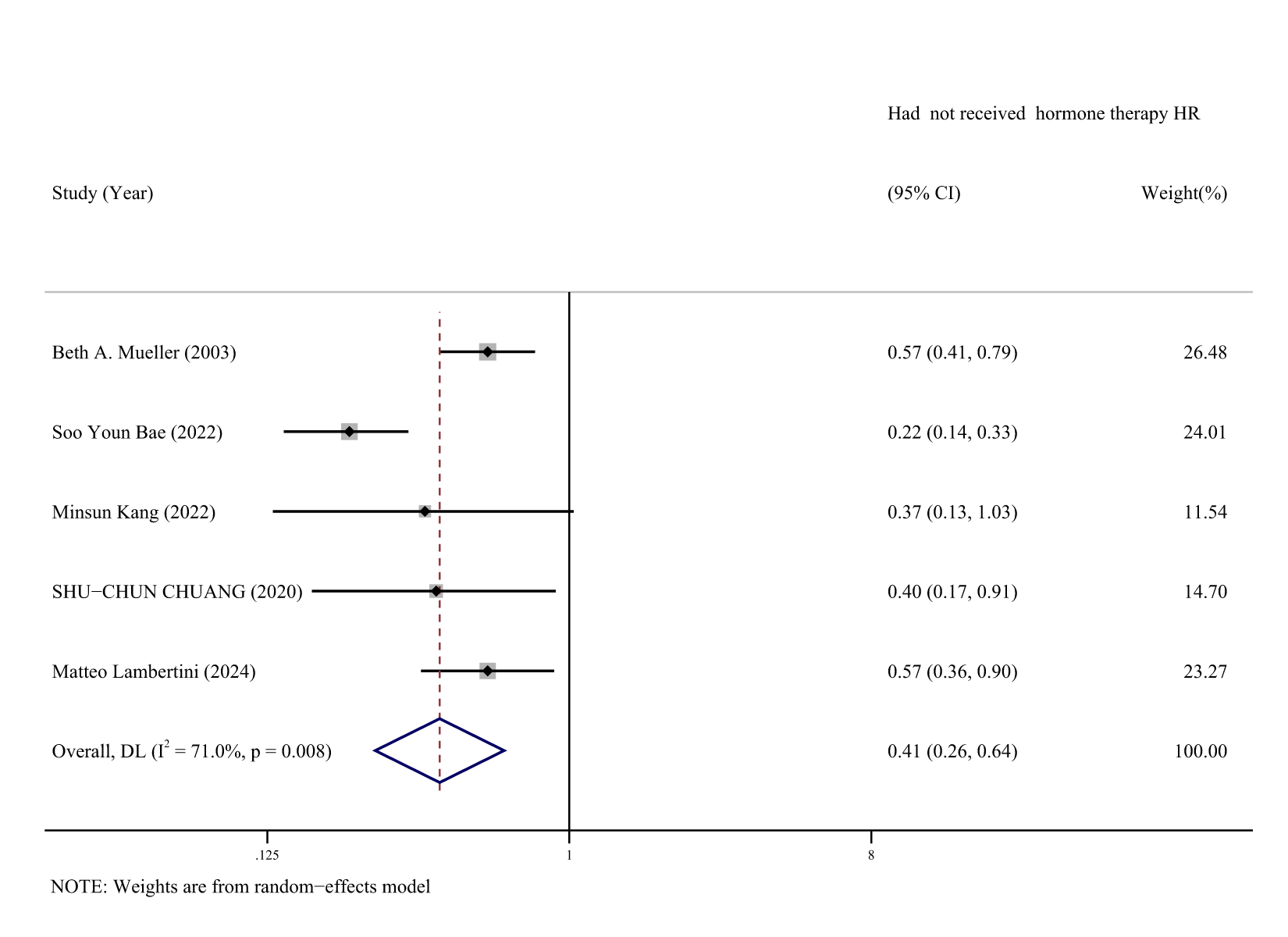


Figure C.18 Forest plot of overall survival in pregnant BC patients who had not received hormone therapy compared with non-pregnant BC patients by HR (hazard ratio, HR)


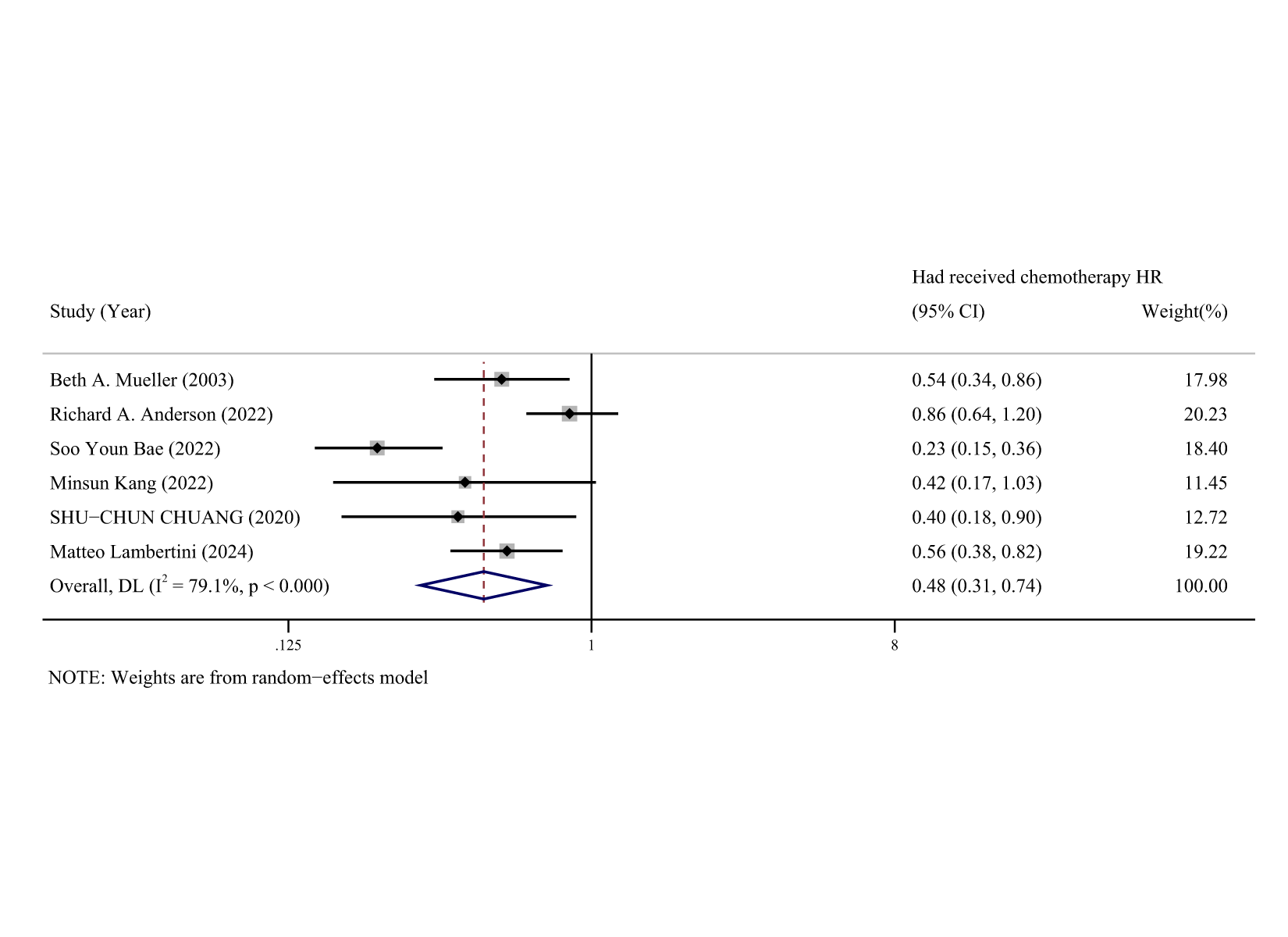


Figure C.19 Forest plot of overall survival in pregnant BC patients who had received chemotherapy compared with non-pregnant BC patients by HR (hazard ratio, HR)


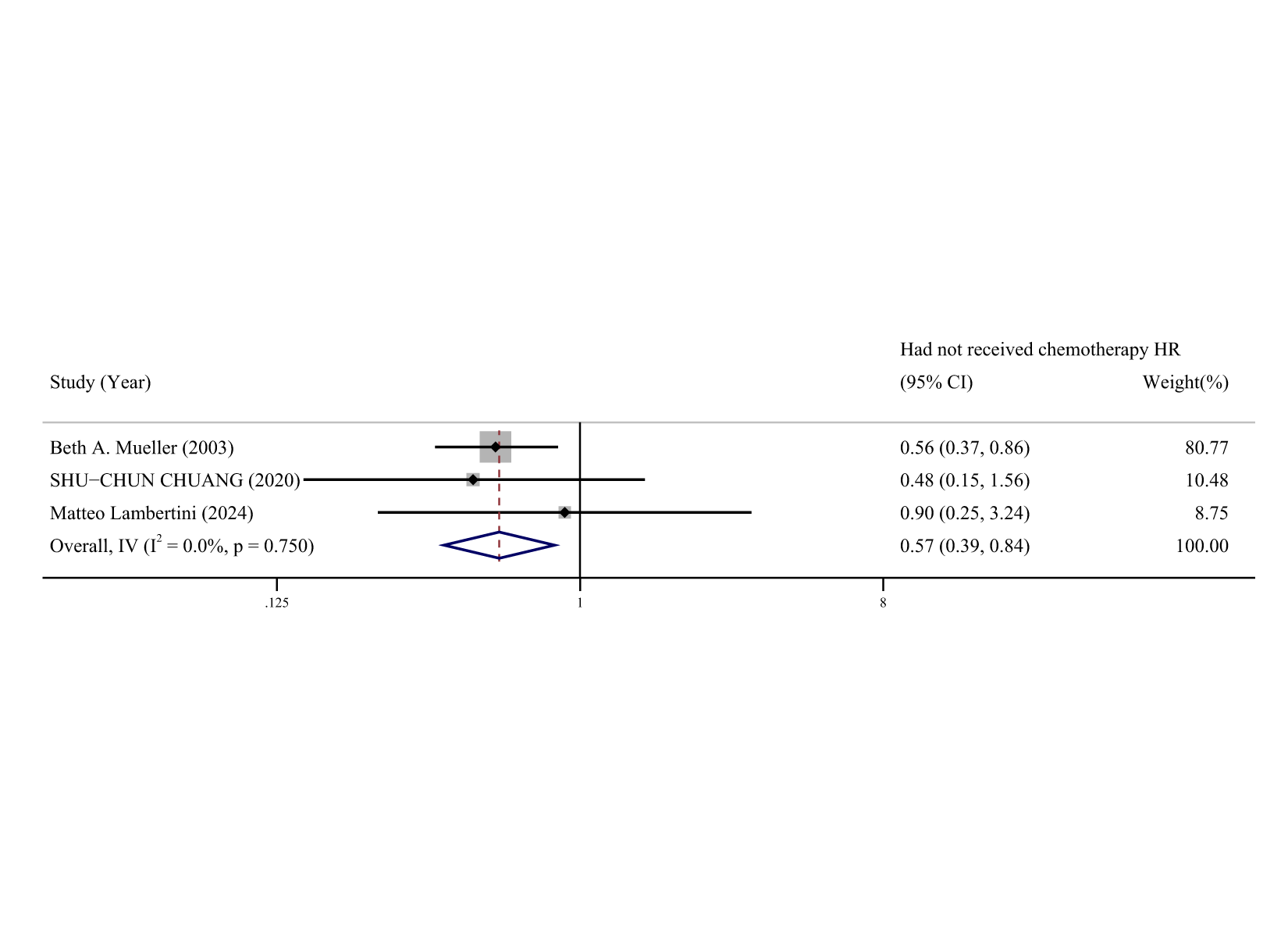


Figure C.20 Forest plot of overall survival in pregnant BC patients who had not received chemotherapy compared with non-pregnant BC patients by HR (hazard ratio, HR)


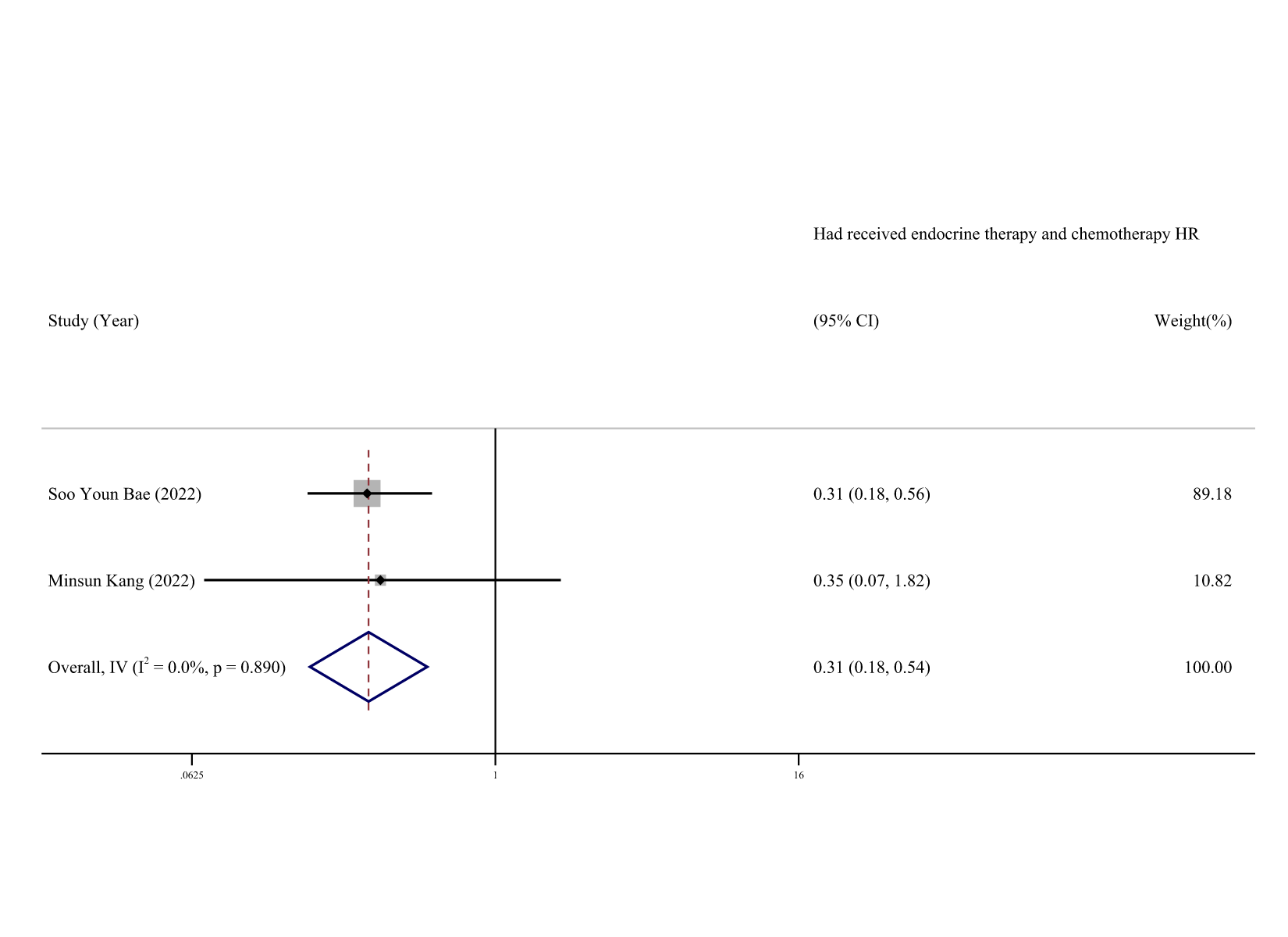


Figure C.21 Forest plot of overall survival in pregnant BC patients who had received endocrine therapy and chemotherapy compared with non-pregnant BC patients by HR (hazard ratio, HR)


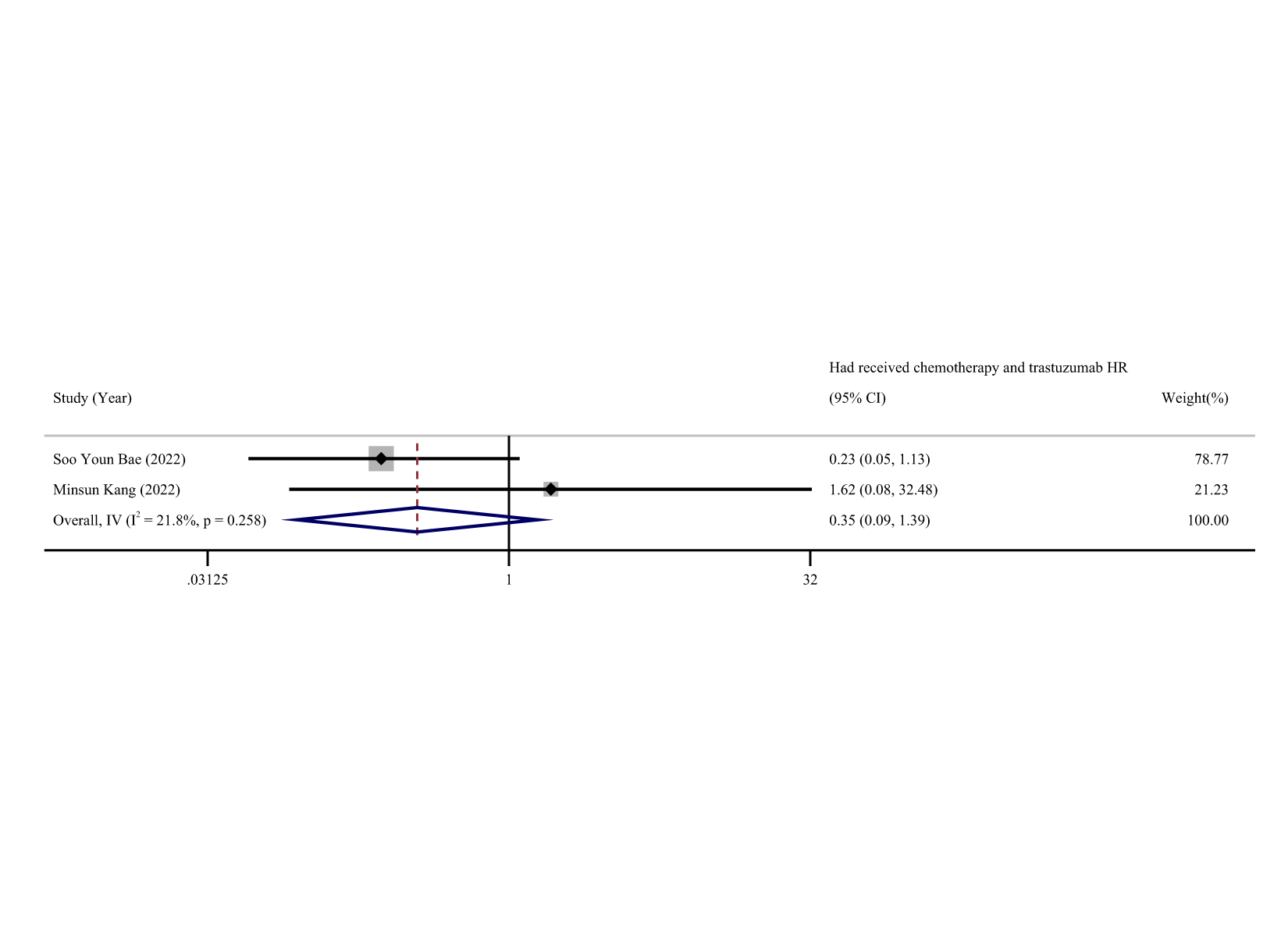


Figure C.22 Forest plot of overall survival in pregnant BC patients who had received chemotherapy and trastuzumab compared with non-pregnant BC patients by HR (hazard ratio, HR)


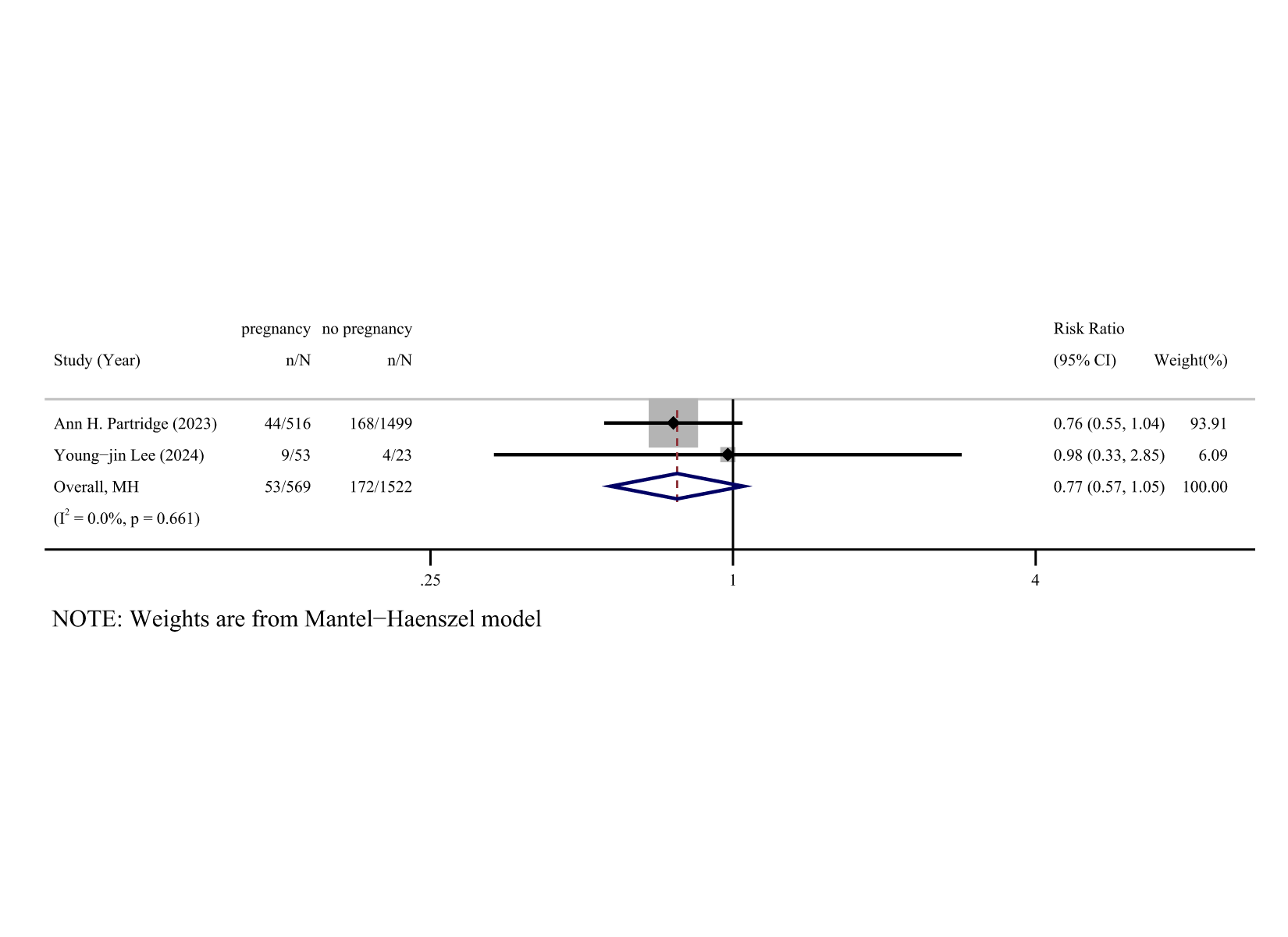


Figure C.23 Forest plot of recurrence rate in ER-positive pregnant BC patients who had had interruption of adjuvant endocrine therapy compared with non-pregnant BC patients by RR (relative risk, RR)


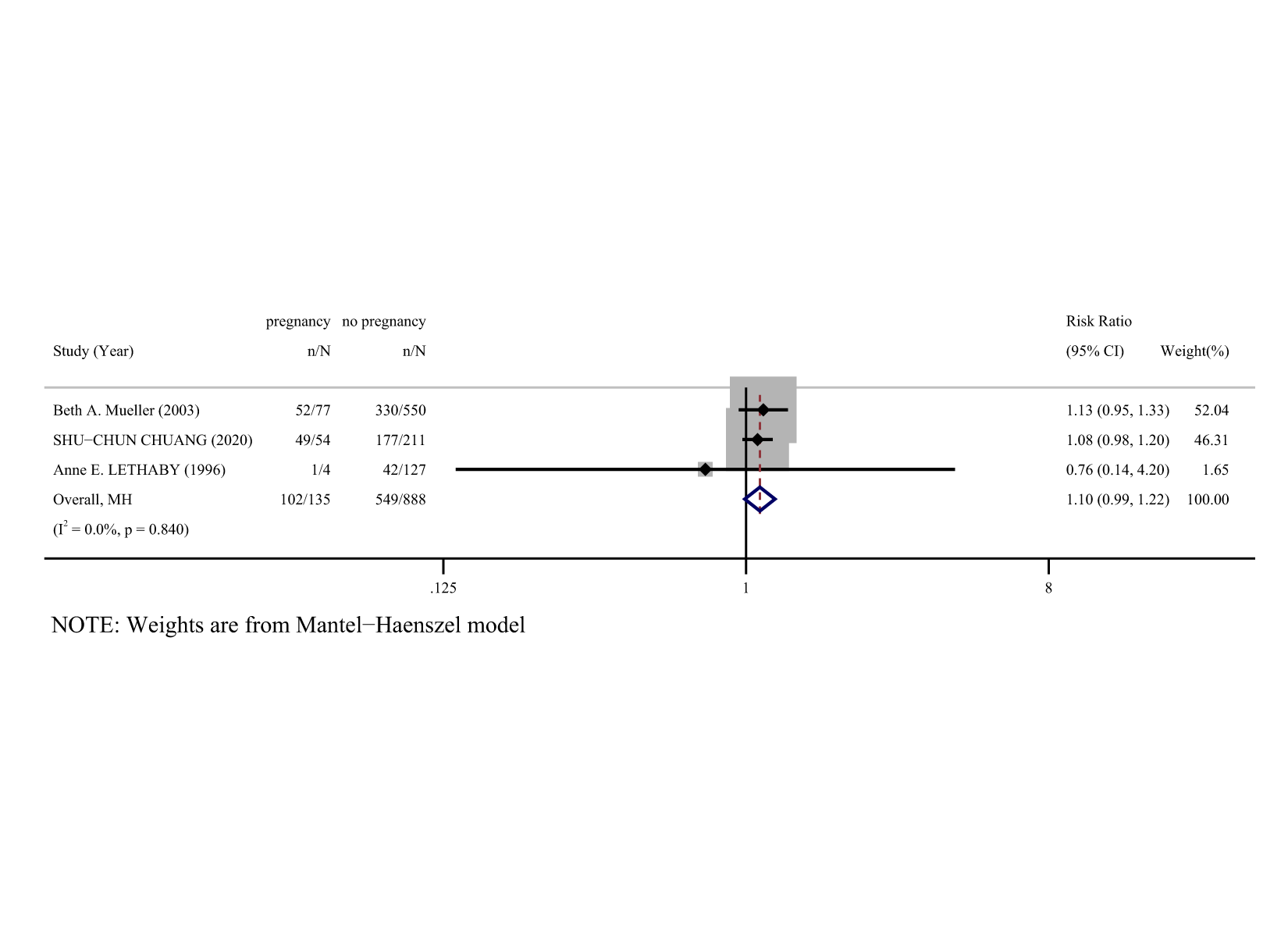


Figure C.24 Forest plot of overall survival in lymph node positive pregnant BC patients compared with non-pregnant BC patients by RR (relative risk, RR)


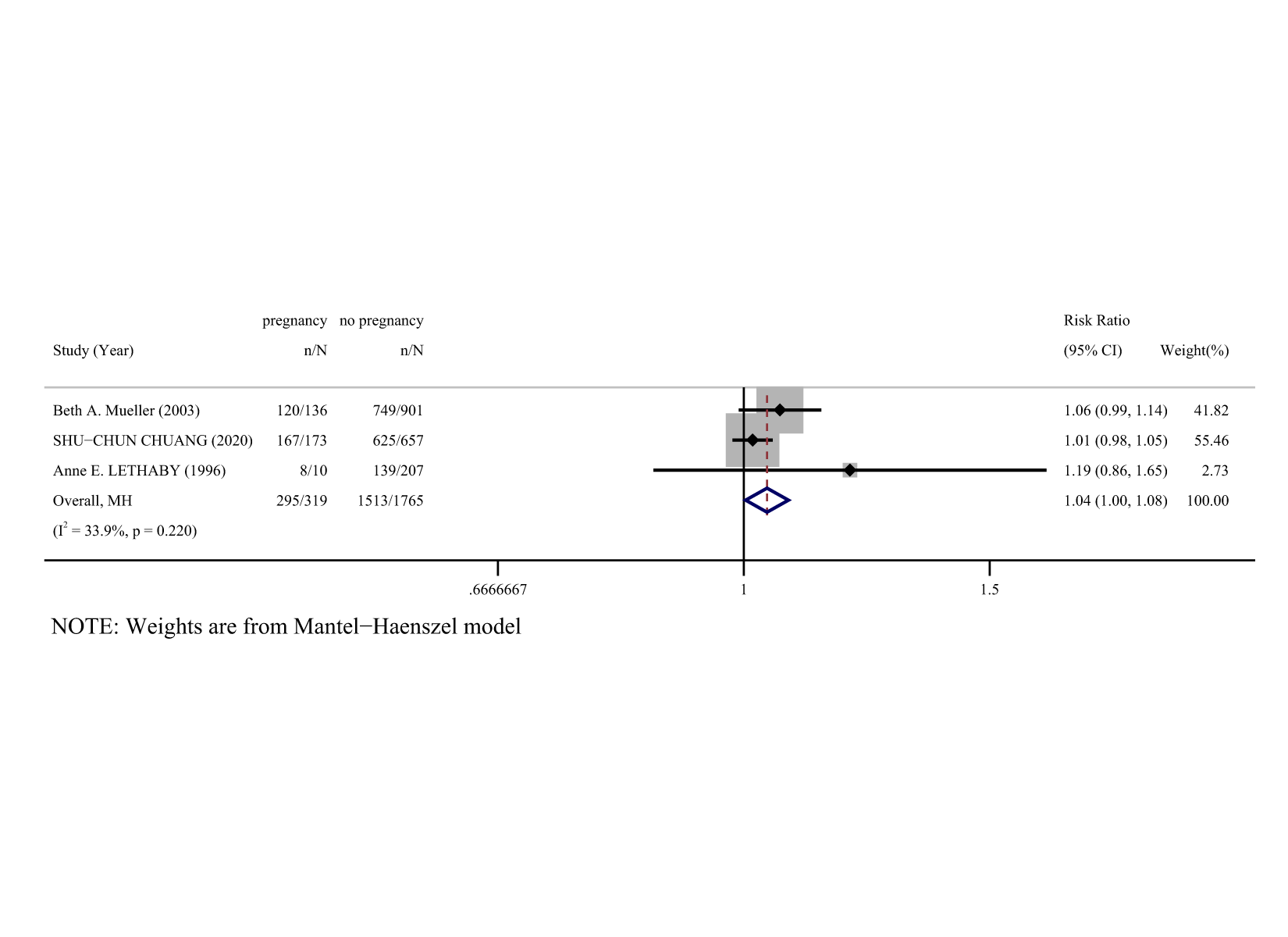


Figure C.25 Forest plot of overall survival in lymph node negative pregnant BC patients compared with non-pregnant BC patients by RR (relative risk, RR)


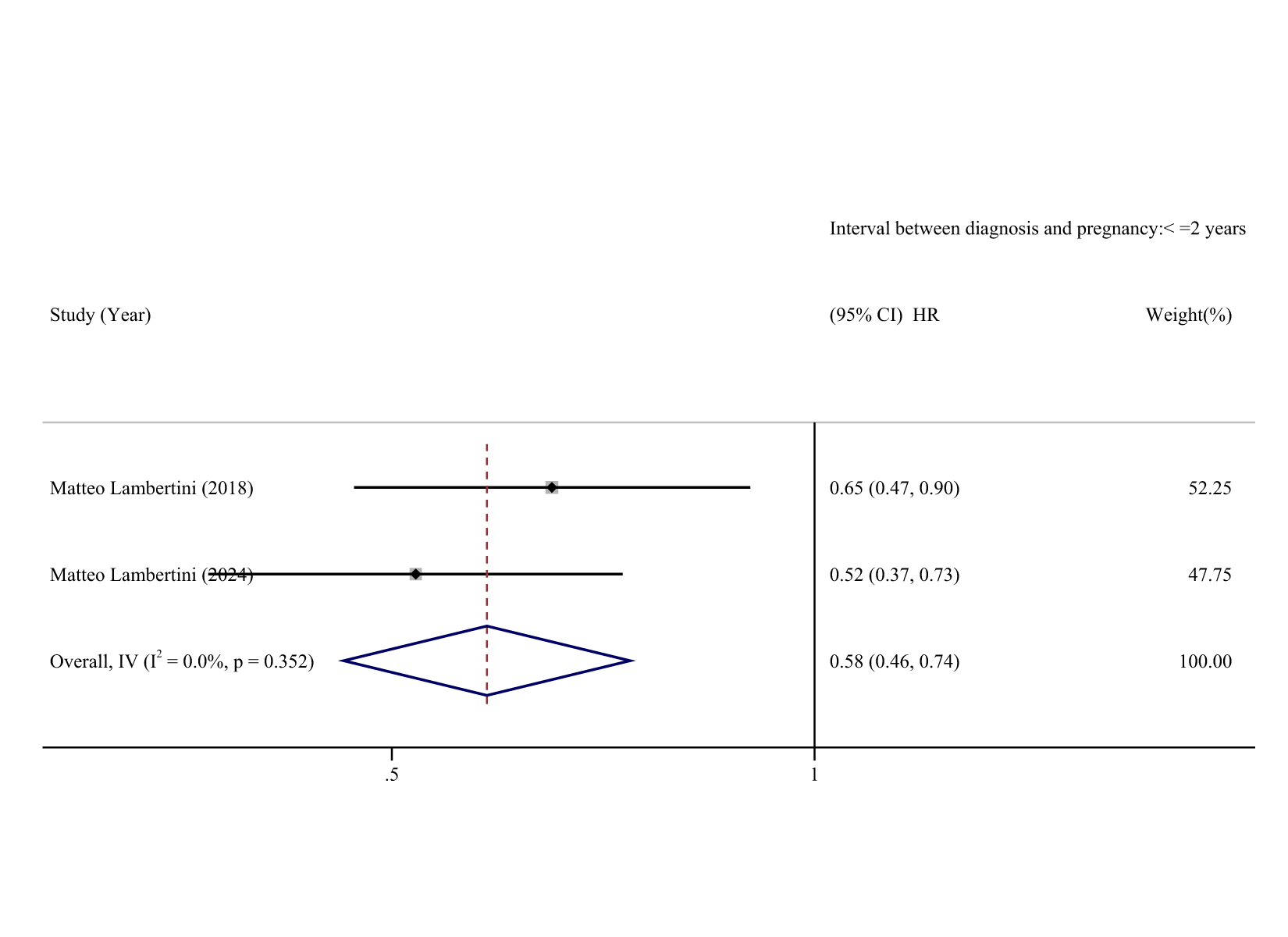


Figure C.26 Forest plot of disease-free survival in pregnant BC patients who had an interval between diagnosis and pregnancy ≤2 years compared with non-pregnant BC patients by HR (hazard ratio, HR)


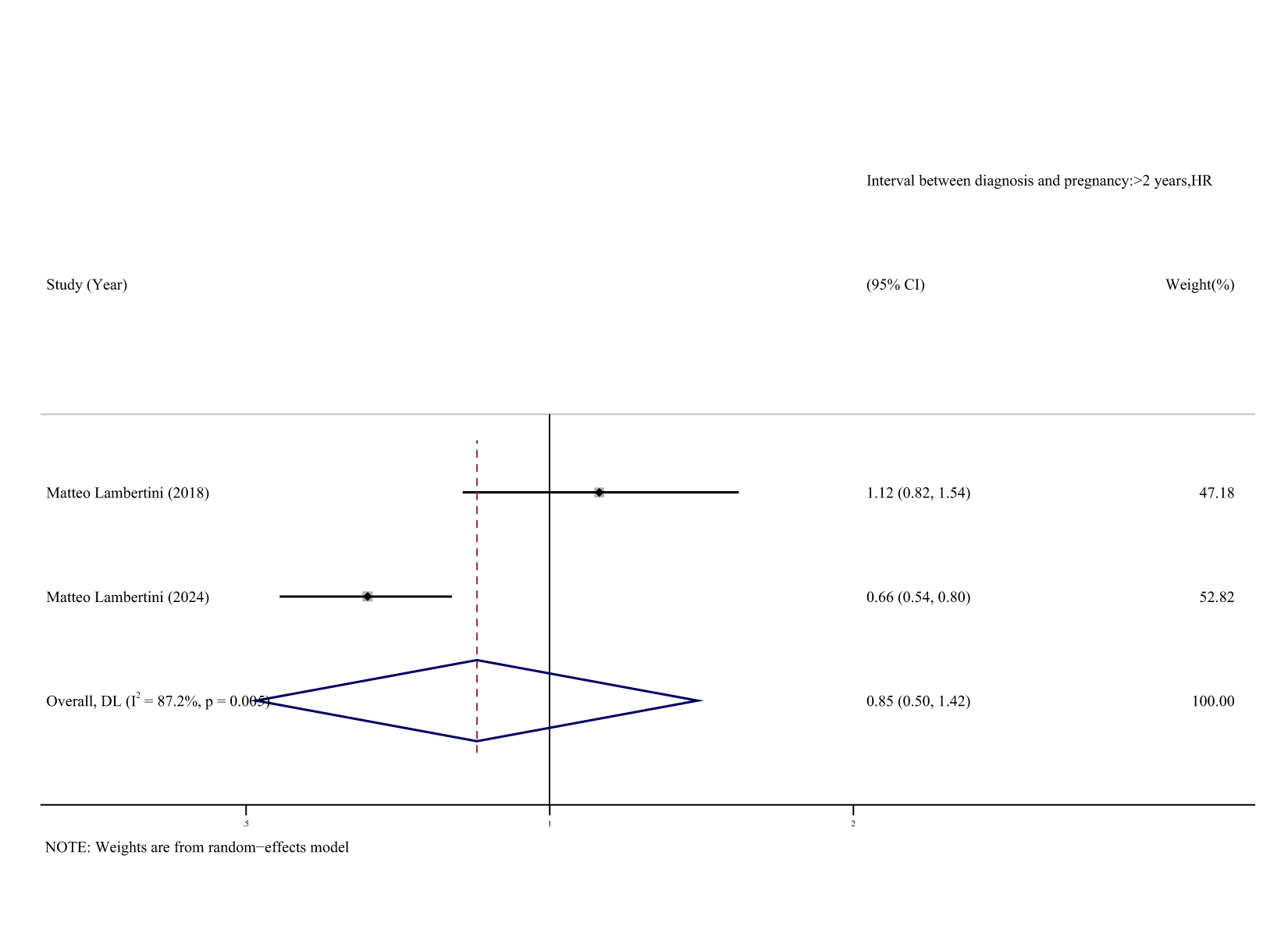


Figure C.27 Forest plot of disease-free survival in pregnant BC patients who had an interval between diagnosis and pregnancy ＞2 years compared with non-pregnant BC patients by HR (hazard ratio, HR)


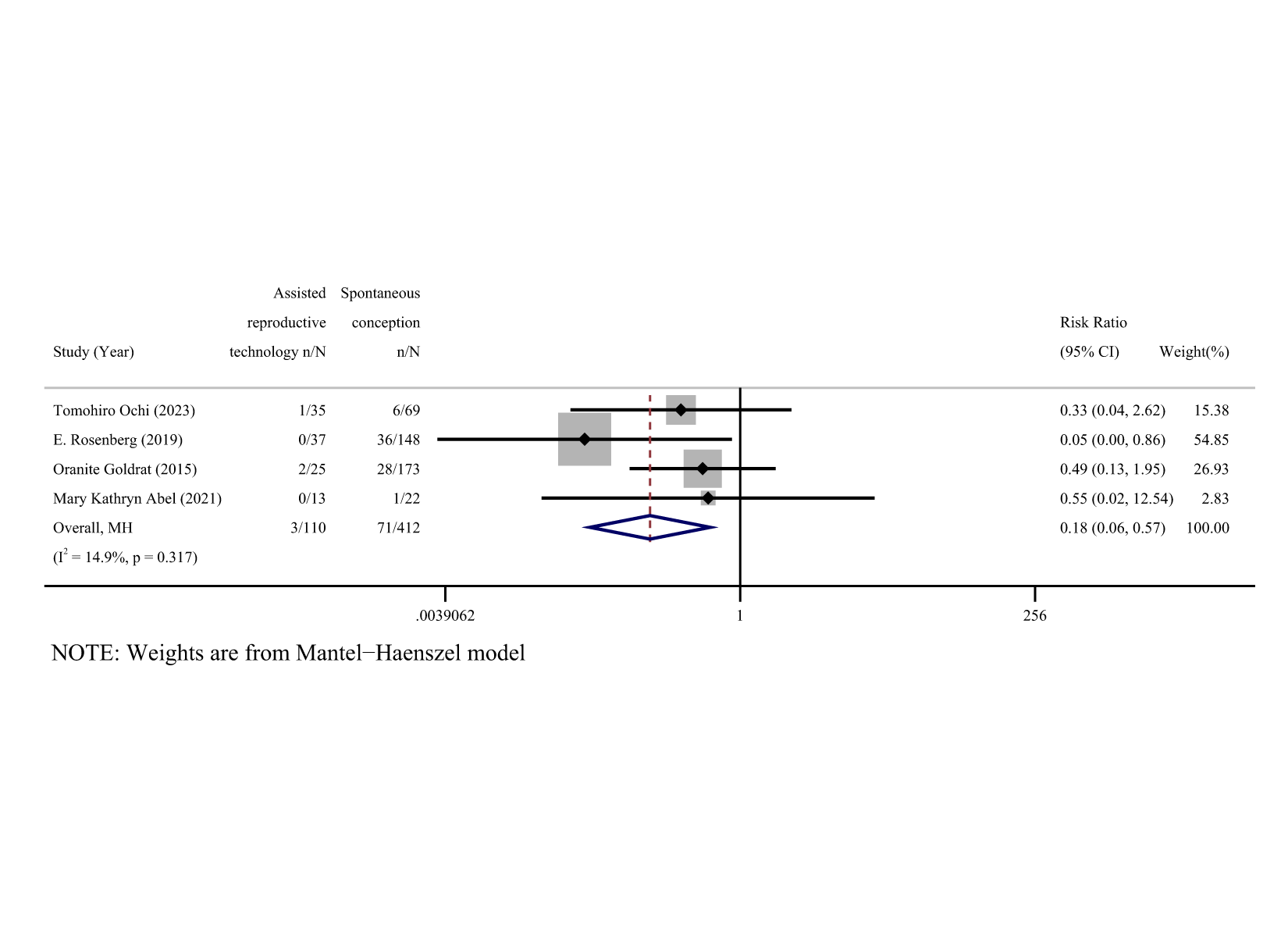


Figure C.28 Forest plot of recurrence rate in pregnant BC patients who had received assisted reproductive technology compared with pregnant BC patients who had received spontaneous conception by RR (relative risk, RR)


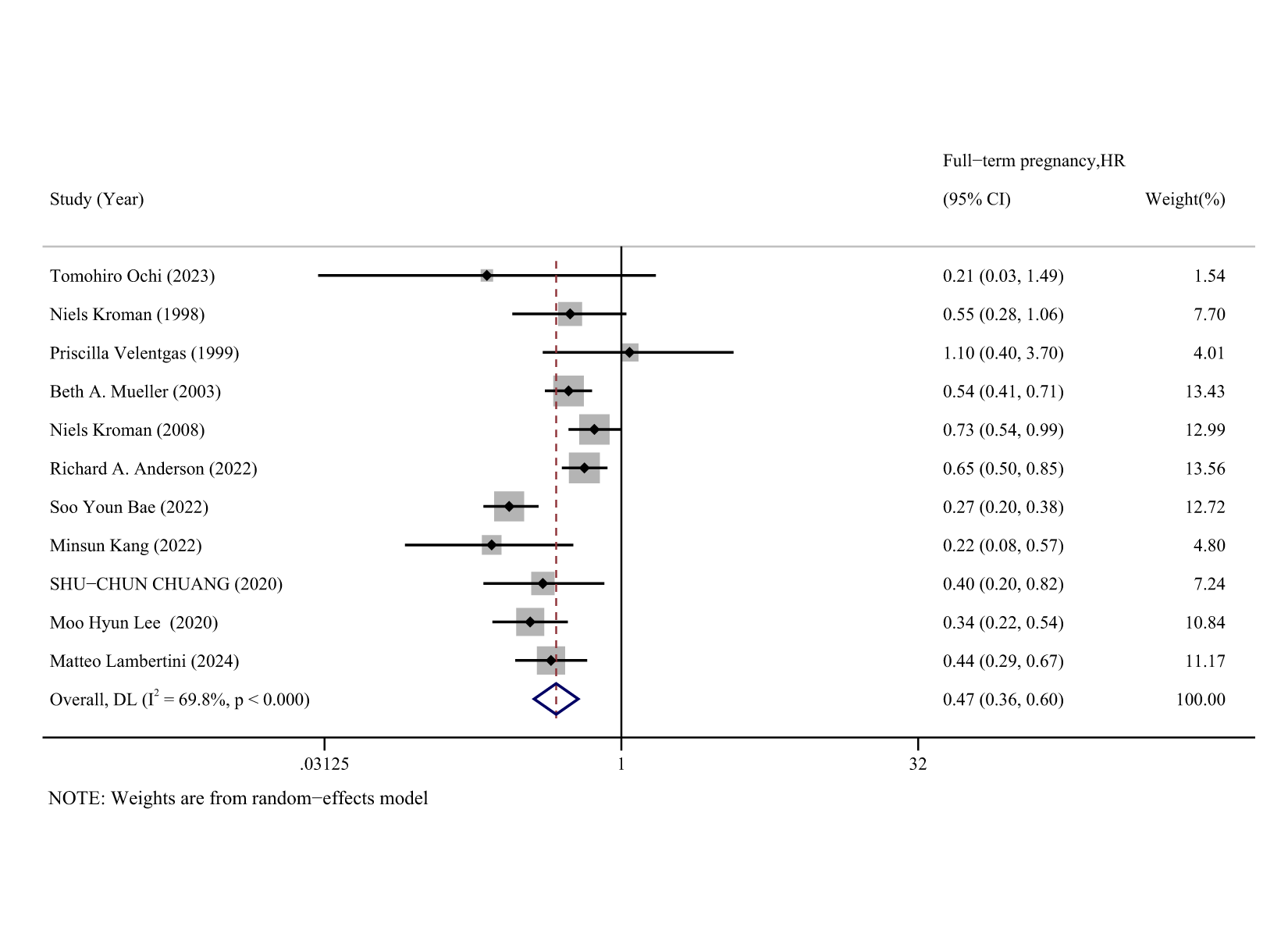


Figure C.29 Forest plot of overall survival in BC patients with full-term pregnancy compared with non-pregnant BC patients by HR (hazard ratio, HR)


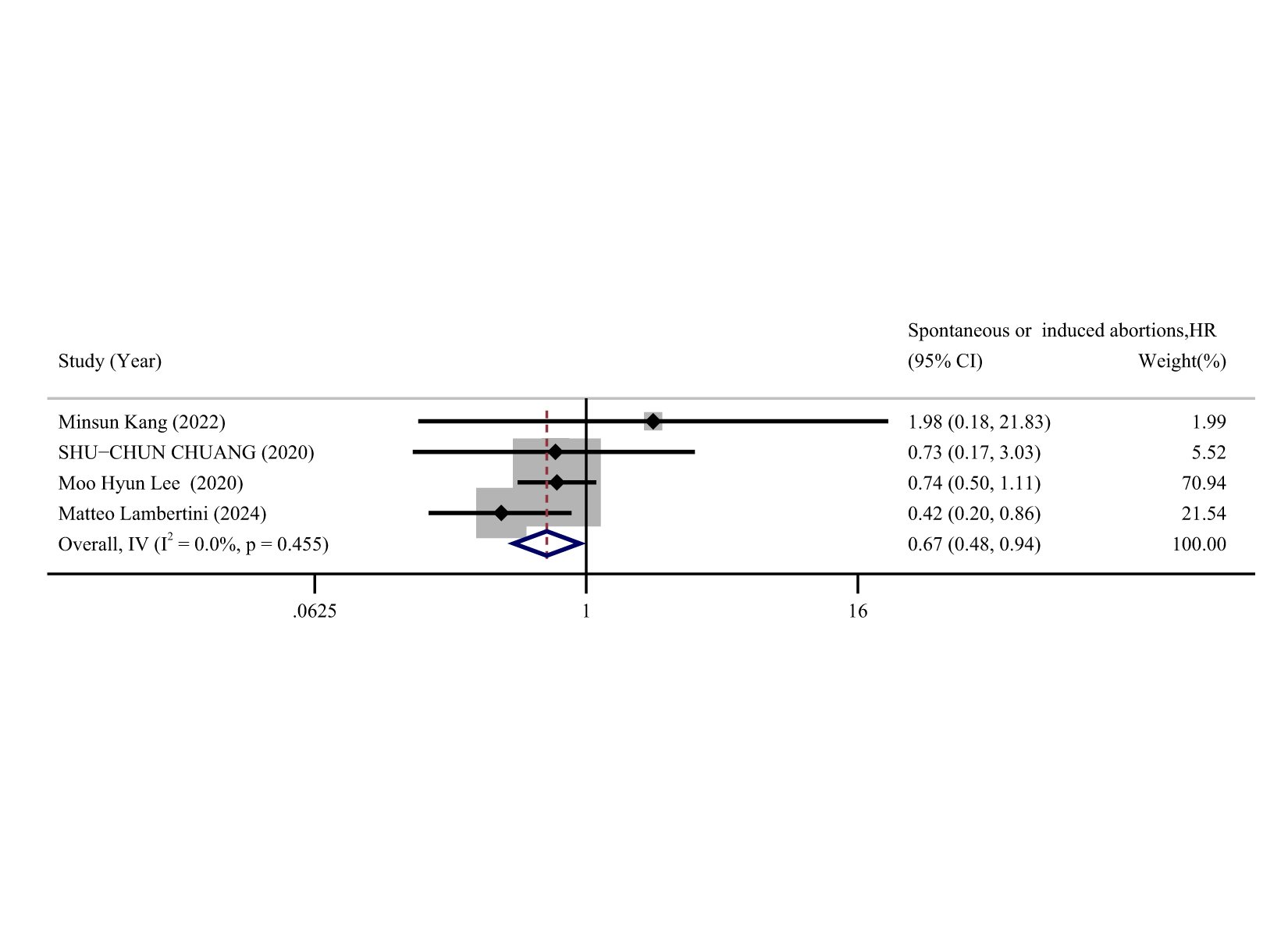


Figure C.30 Forest plot of overall survival in BC patients with spontaneous or induced abortions compared with non-pregnant BC patients by HR (hazard ratio, HR)


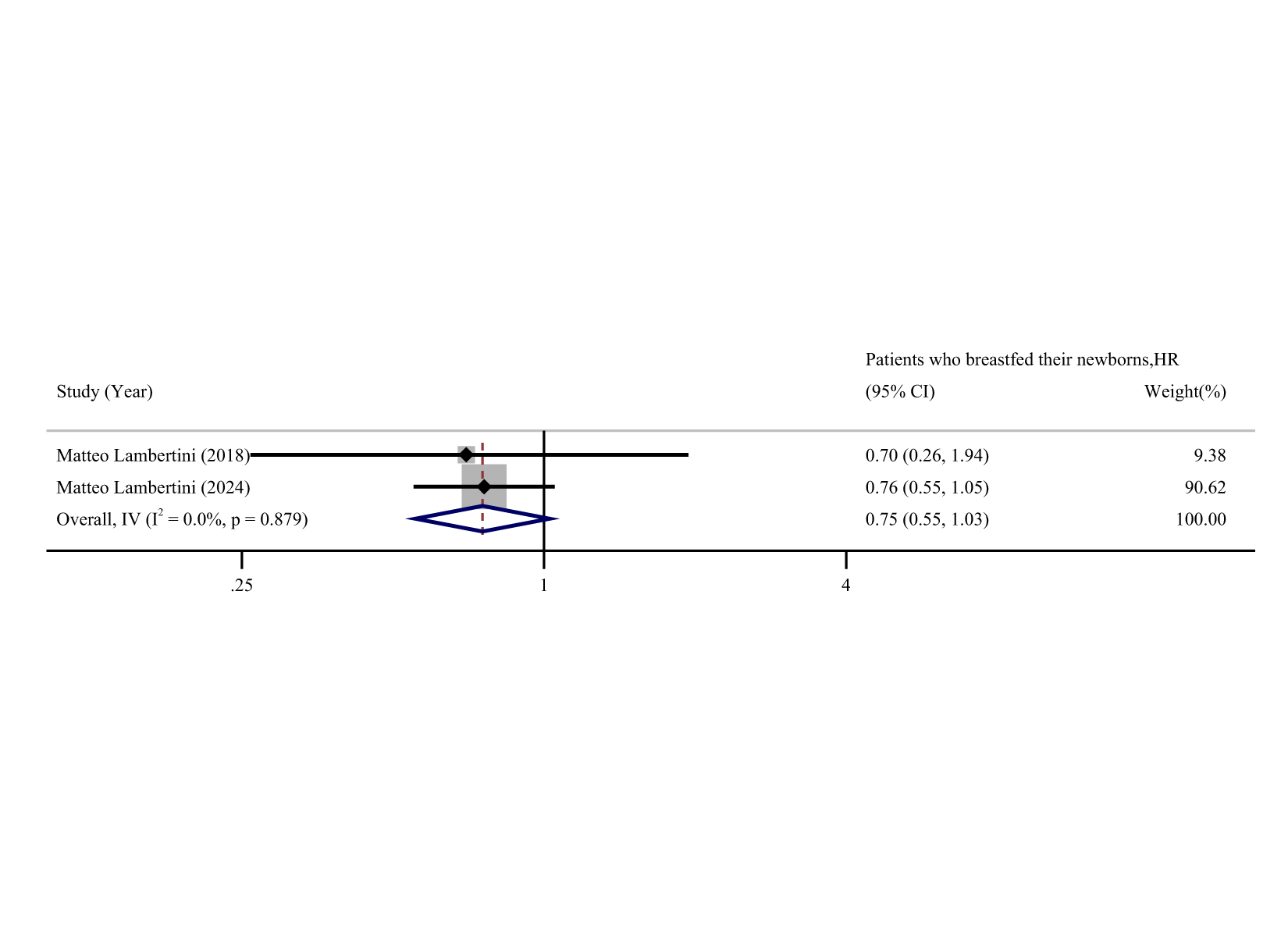


Figure C.31 Forest plot of disease-free survival in pregnant BC patients who had breastfed their newborns compared with non-pregnant BC patients by HR (hazard ratio, HR)


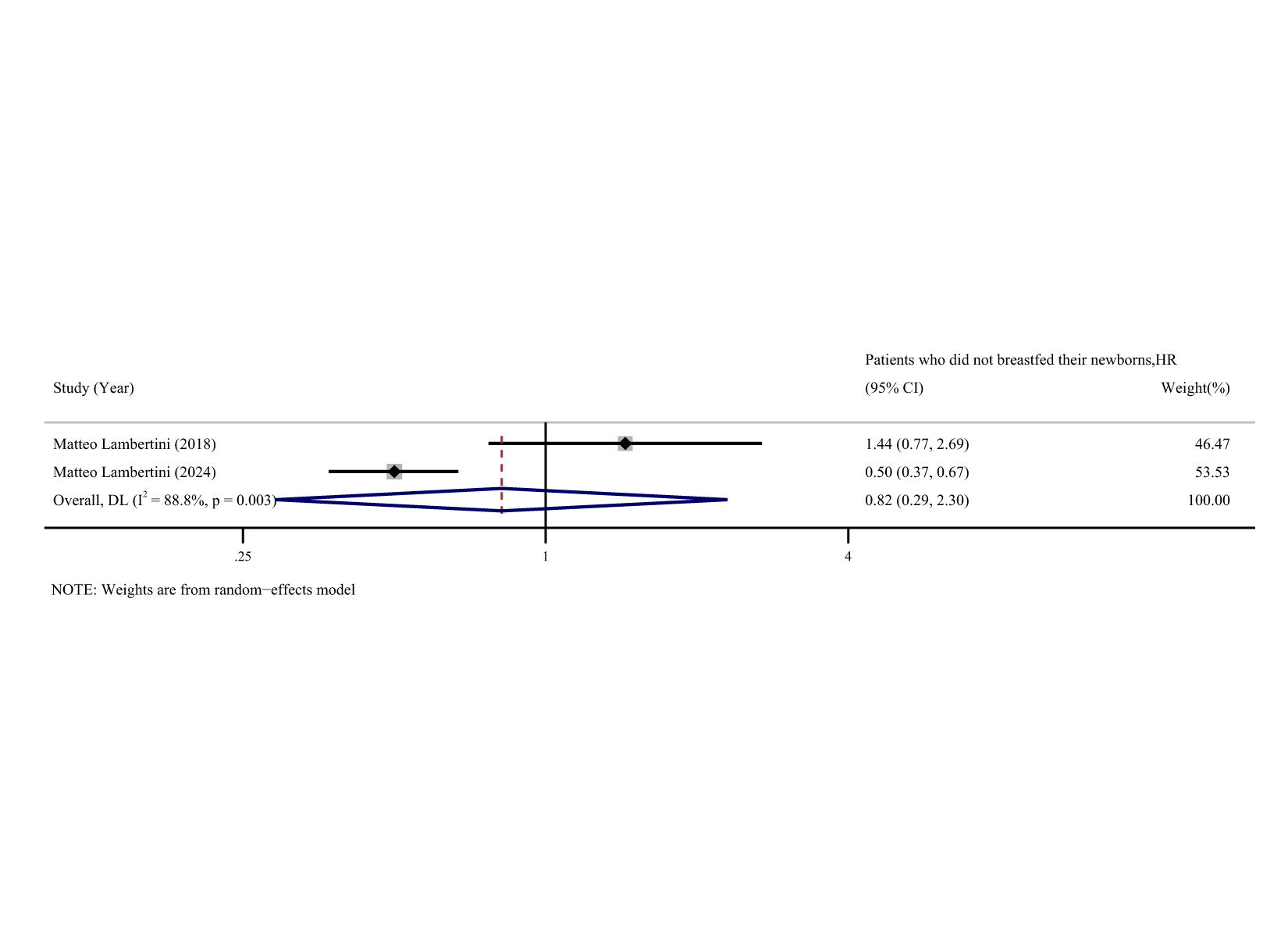


Figure C.32 Forest plot of disease-free survival in pregnant BC patients who had not breastfed their newborns compared with non-pregnant BC patients by HR (hazard ratio, HR)


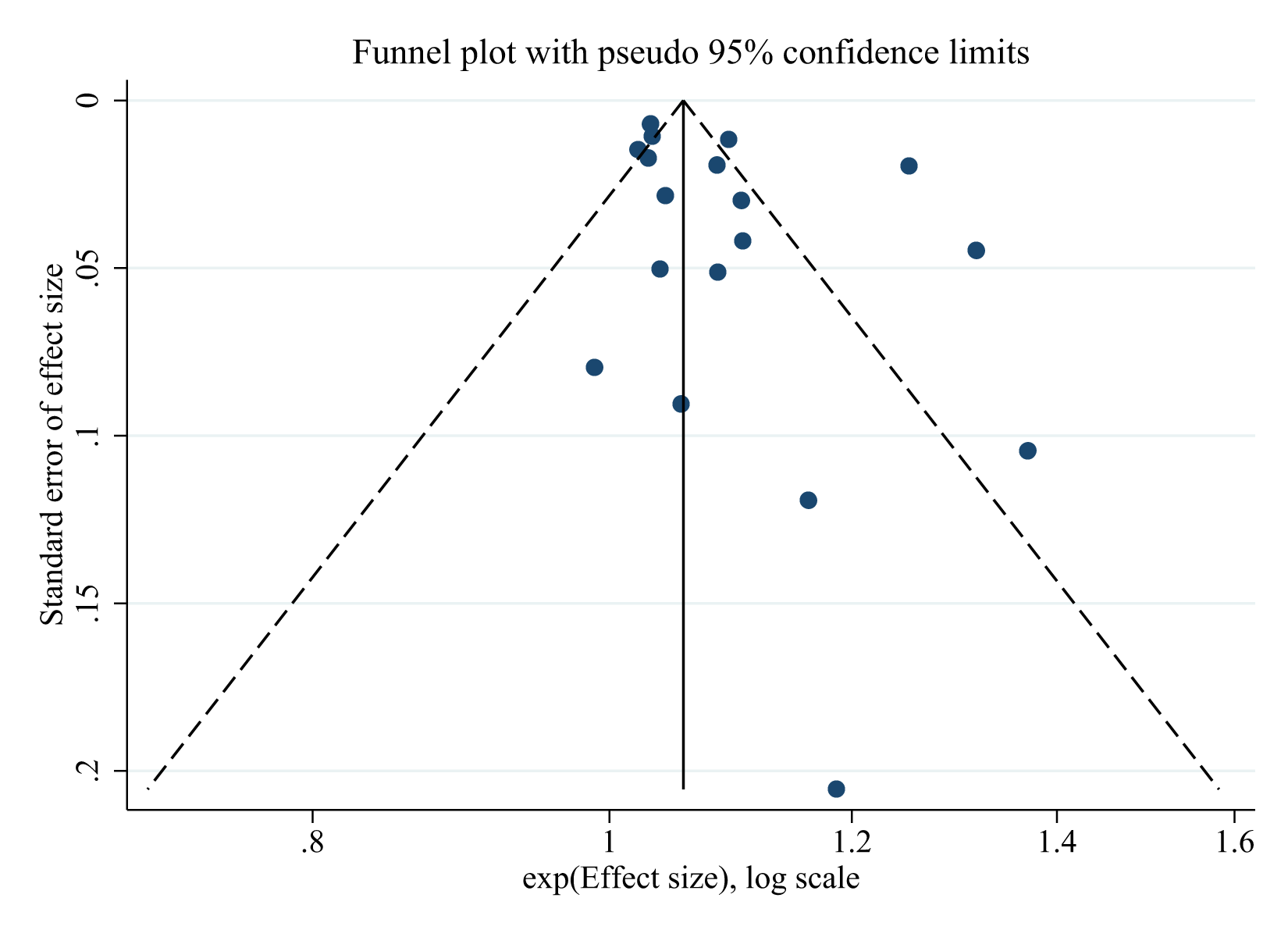


Figure C.33 Funnel plot of overall survival in pregnant BC patients compared with non-pregnant BC patients


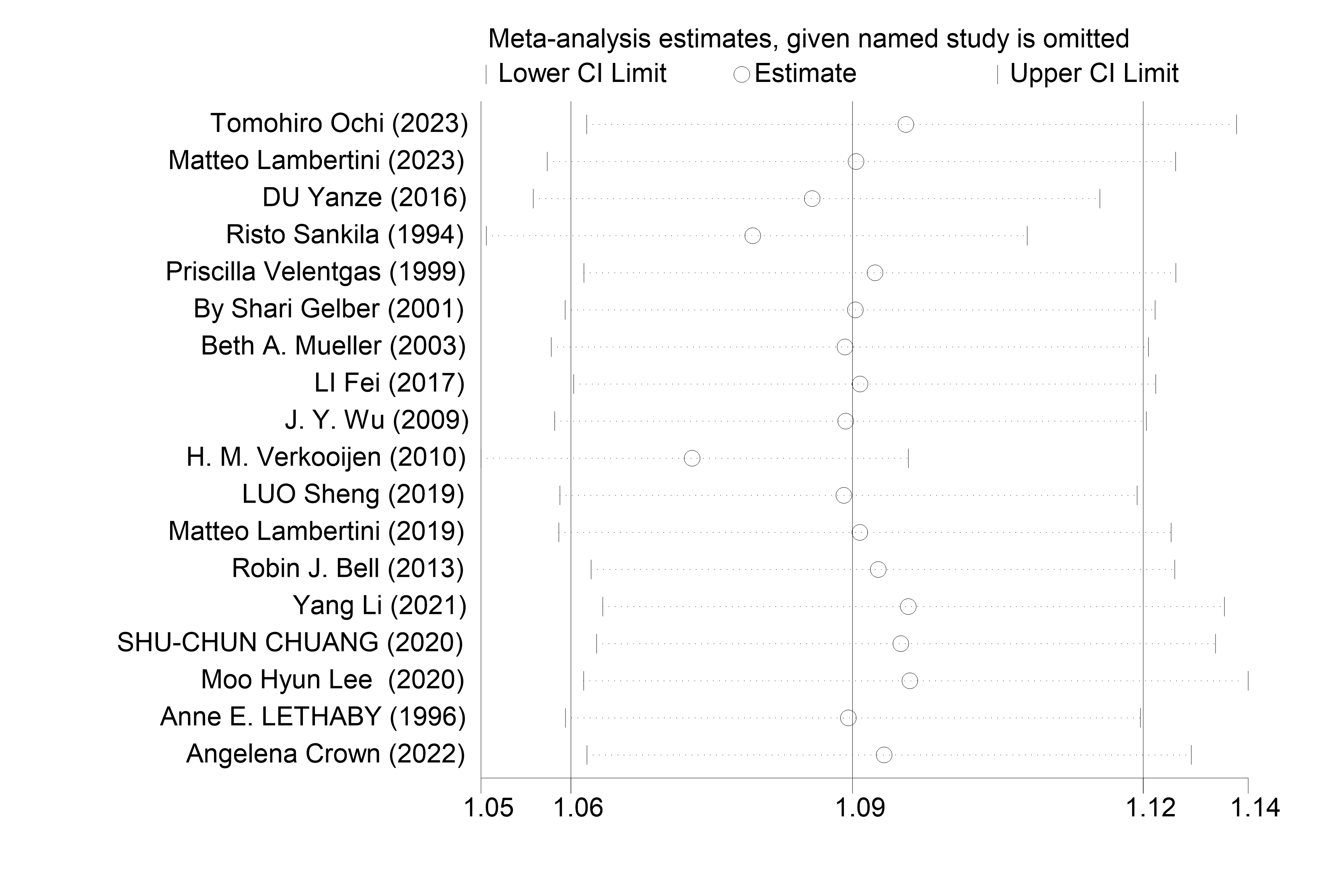
 Figure C.34 Sensitivity analysis of overall survival in pregnant BC patients compared with non-pregnant BC patients


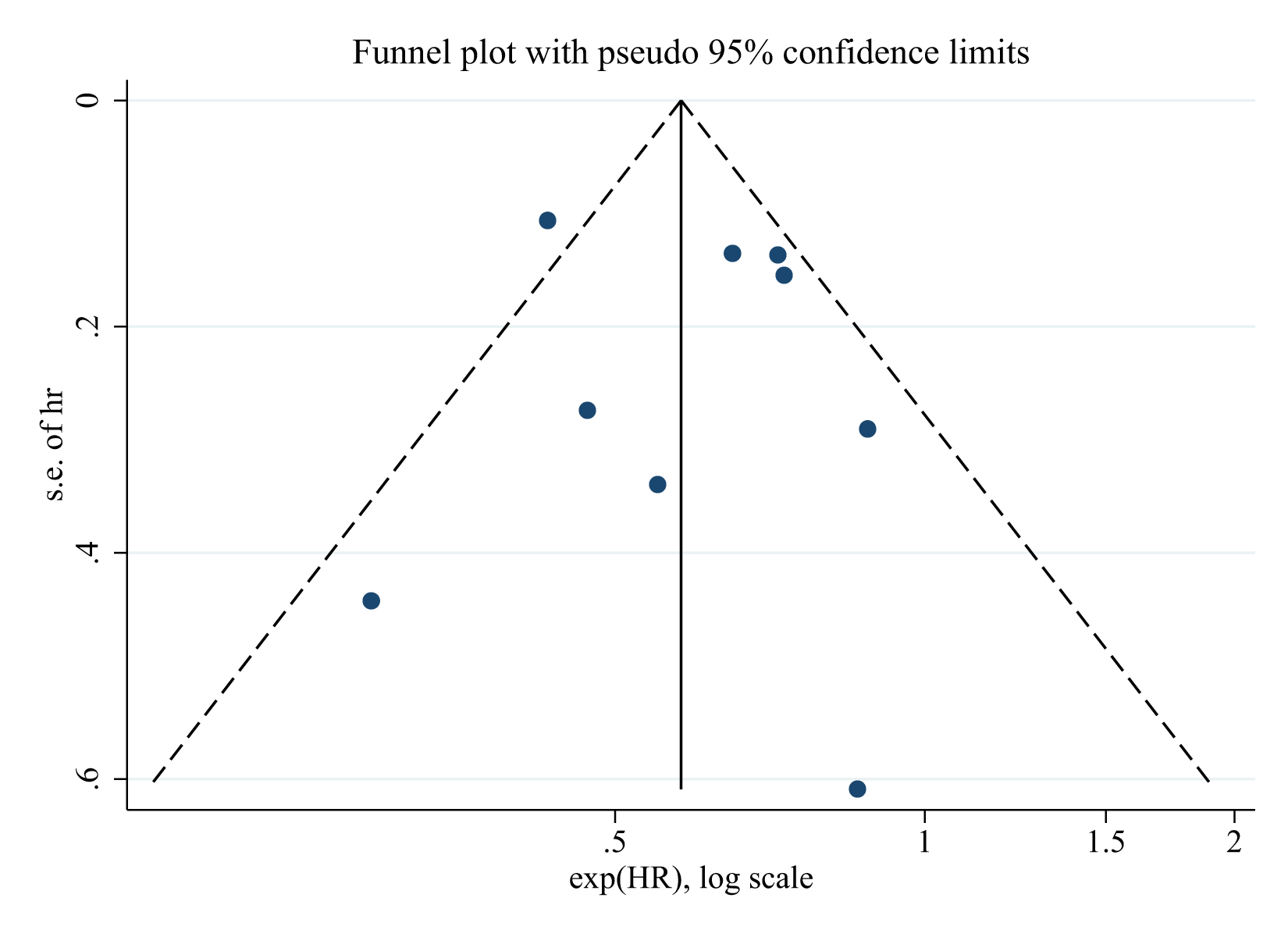


Figure C.35 Funnel plot of overall survival in pregnant BC patients compared with non-pregnant BC patients


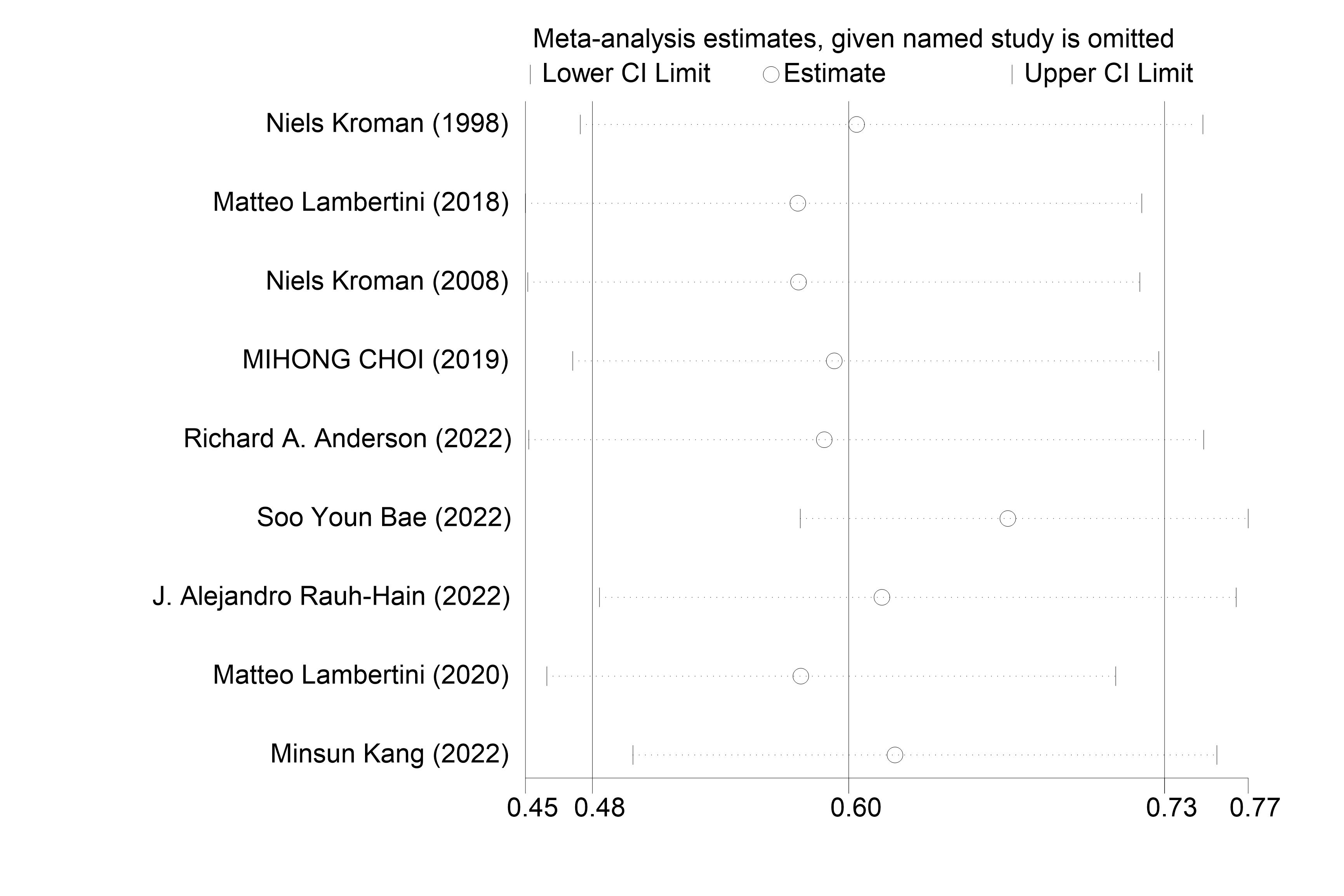


Figure C.36 Sensitivity analysis of overall survival in pregnant BC patients compared with non-pregnant BC patients


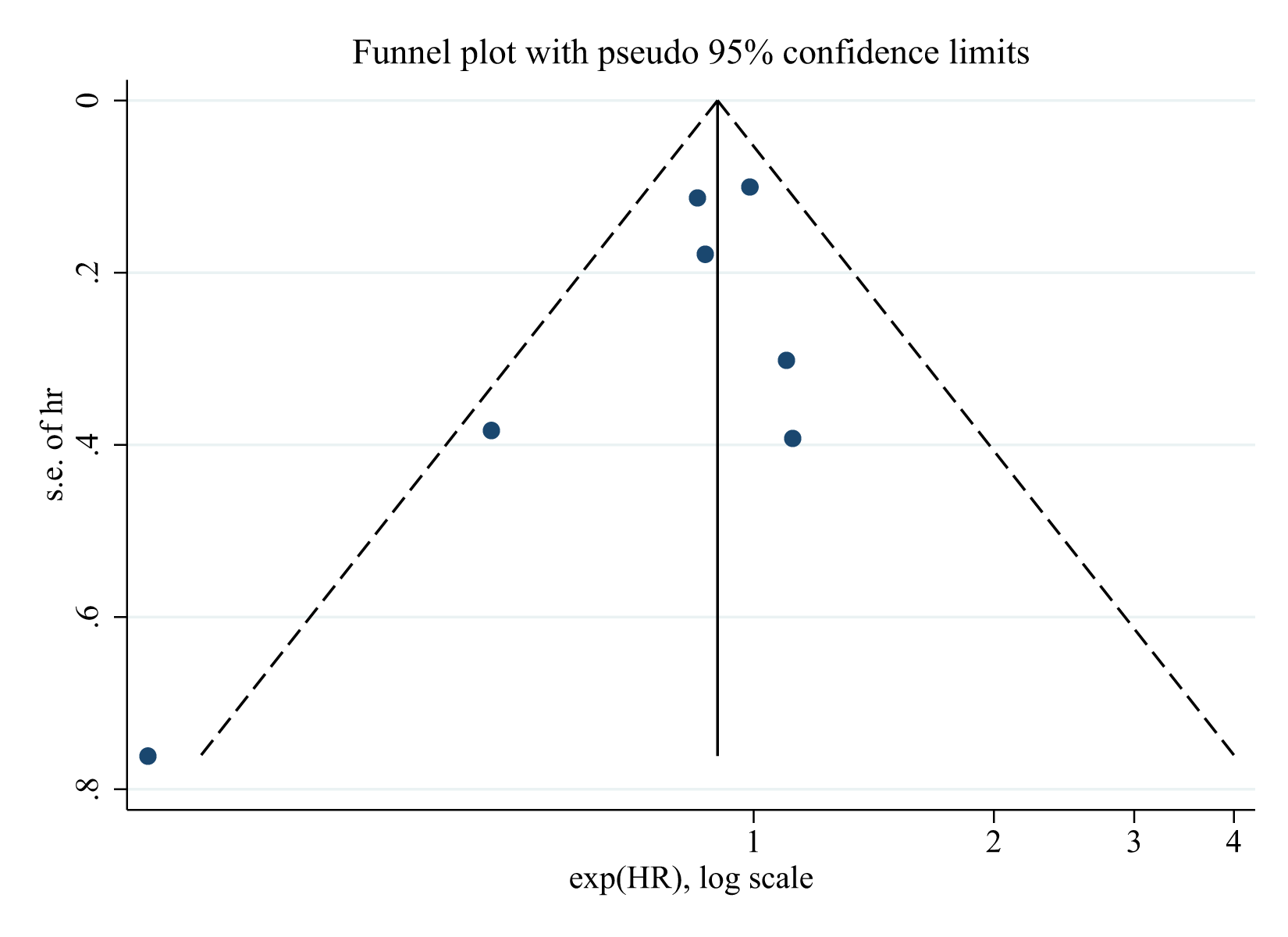


Figure C.37 Funnel plot of disease-free survival in pregnant BC patients compared with non-pregnant BC patients


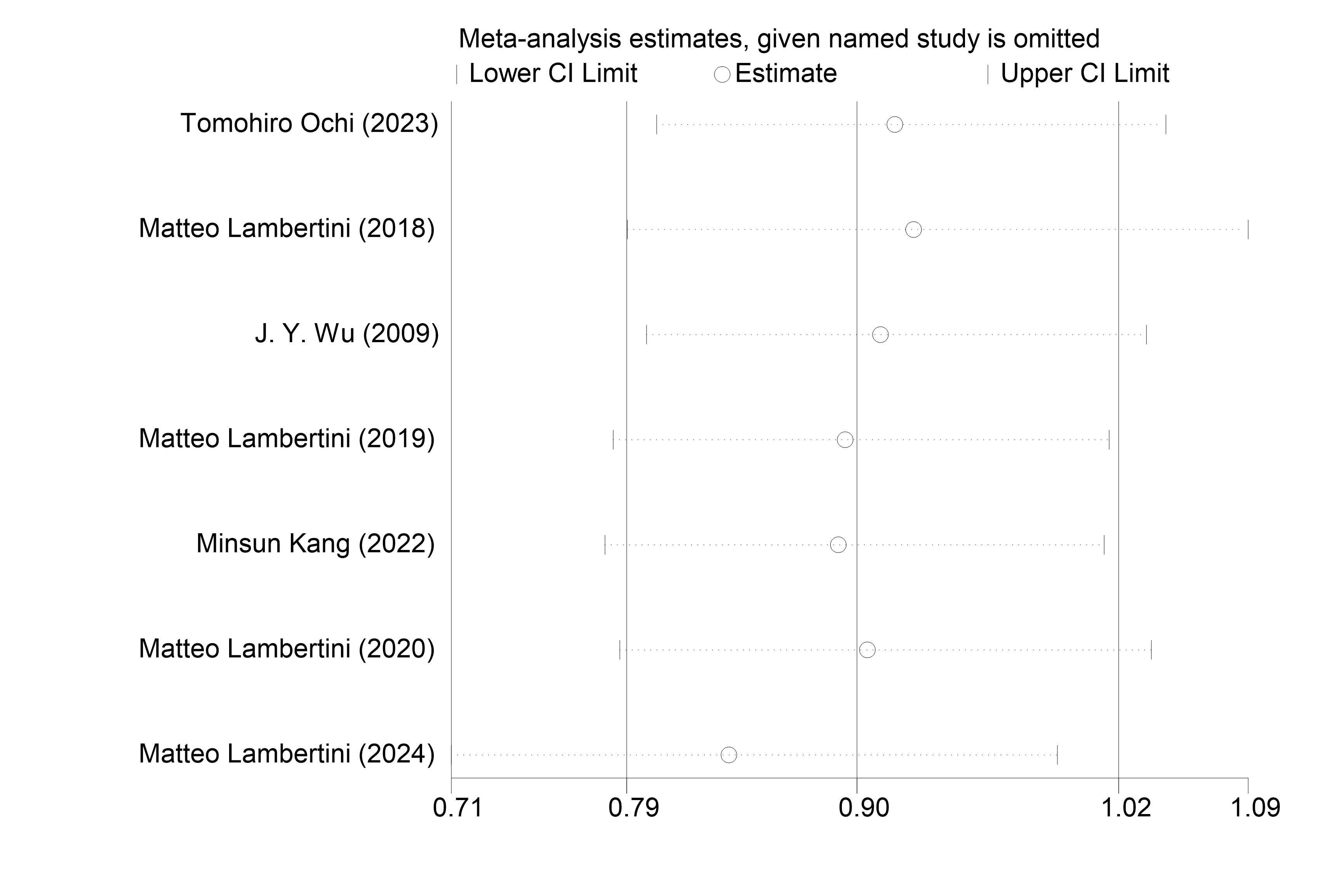


Figure C.38 Sensitivity analysis of disease-free survival in pregnant BC patients compared with non-pregnant BC patients


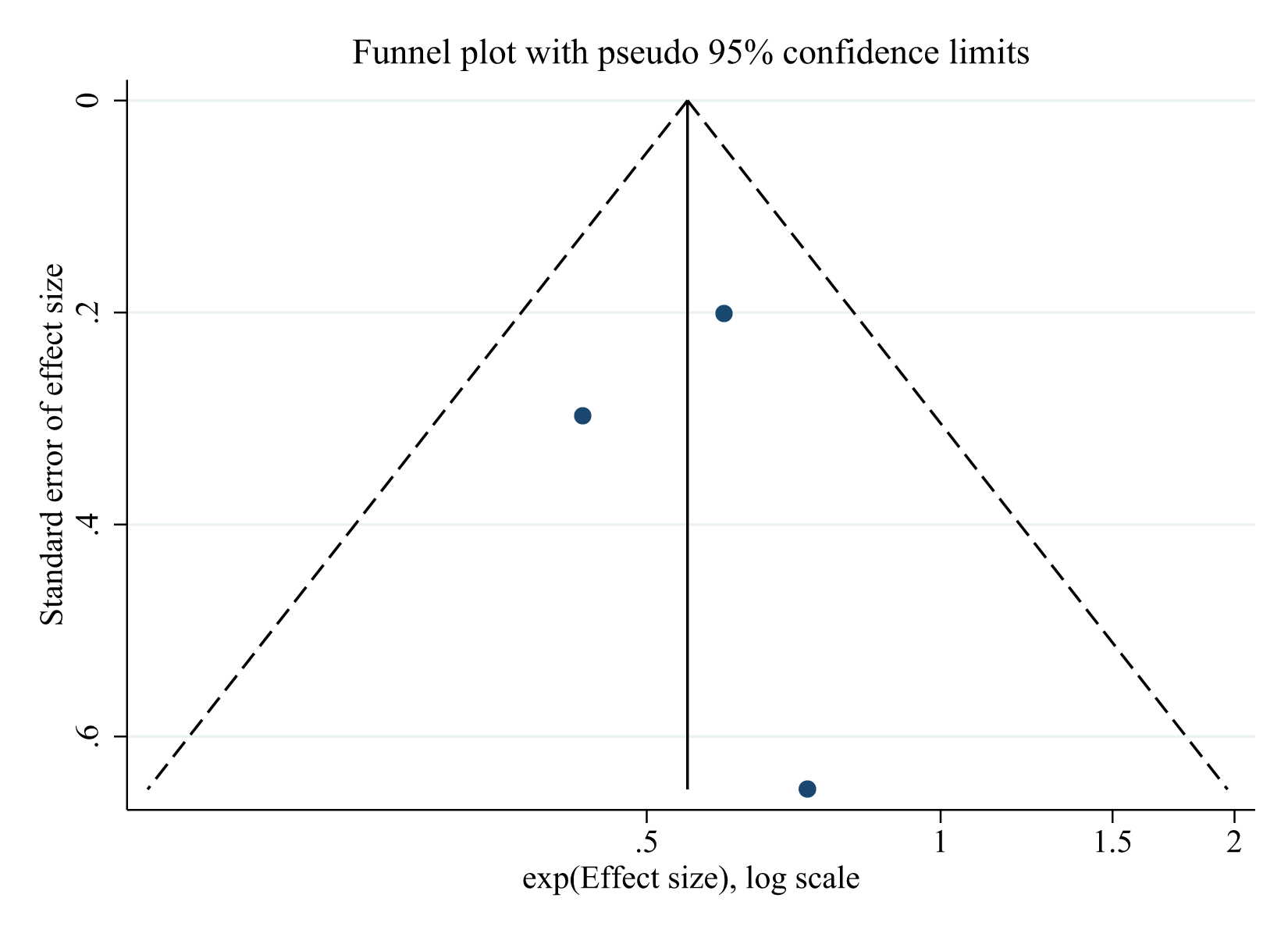


Figure C.39 Funnel plot of breast cancer specific survival in pregnant BC patients compared with non-pregnant BC patients


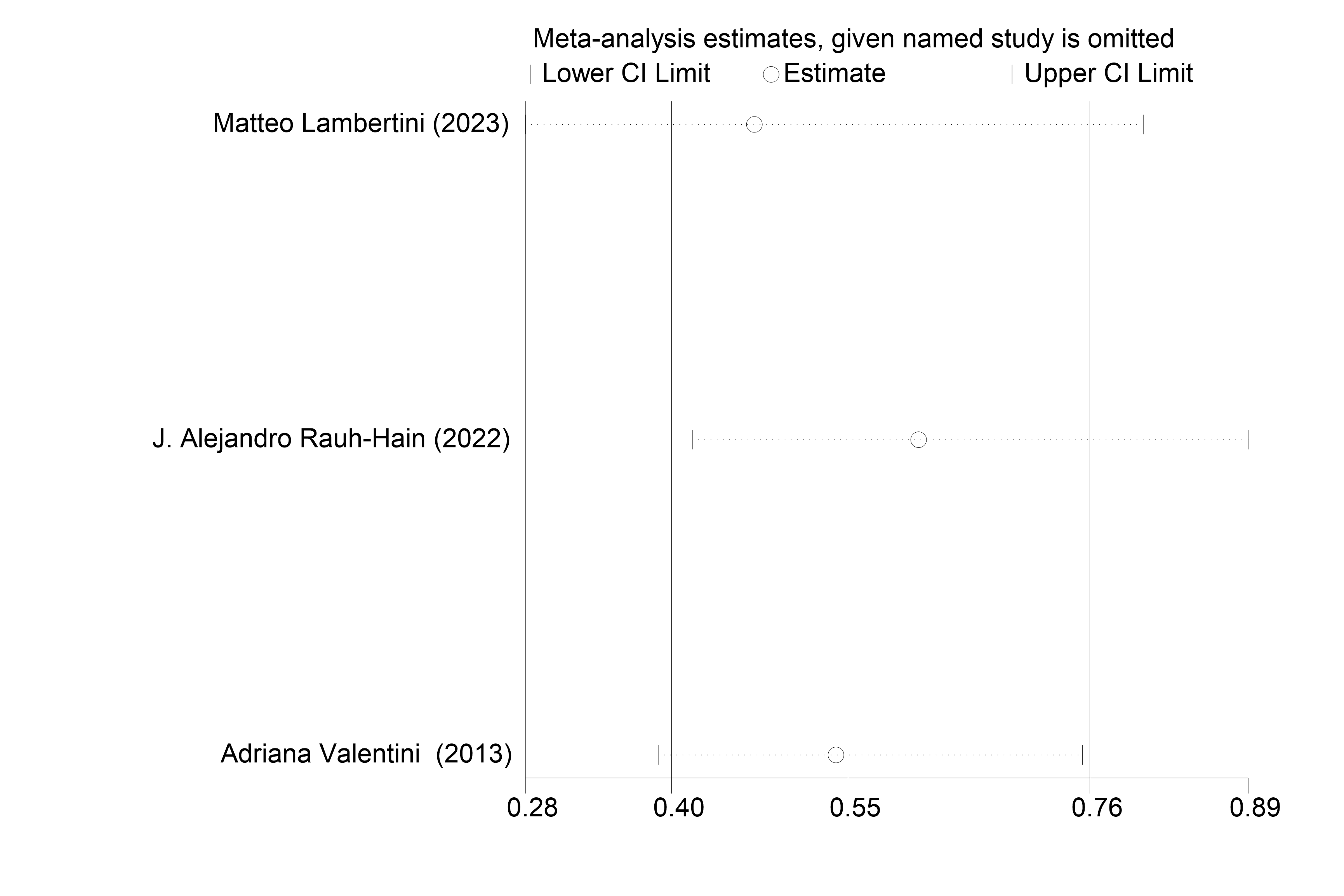


Figure C.40 Sensitivity analysis of breast cancer specific survival in pregnant BC patients compared with non-pregnant BC patients


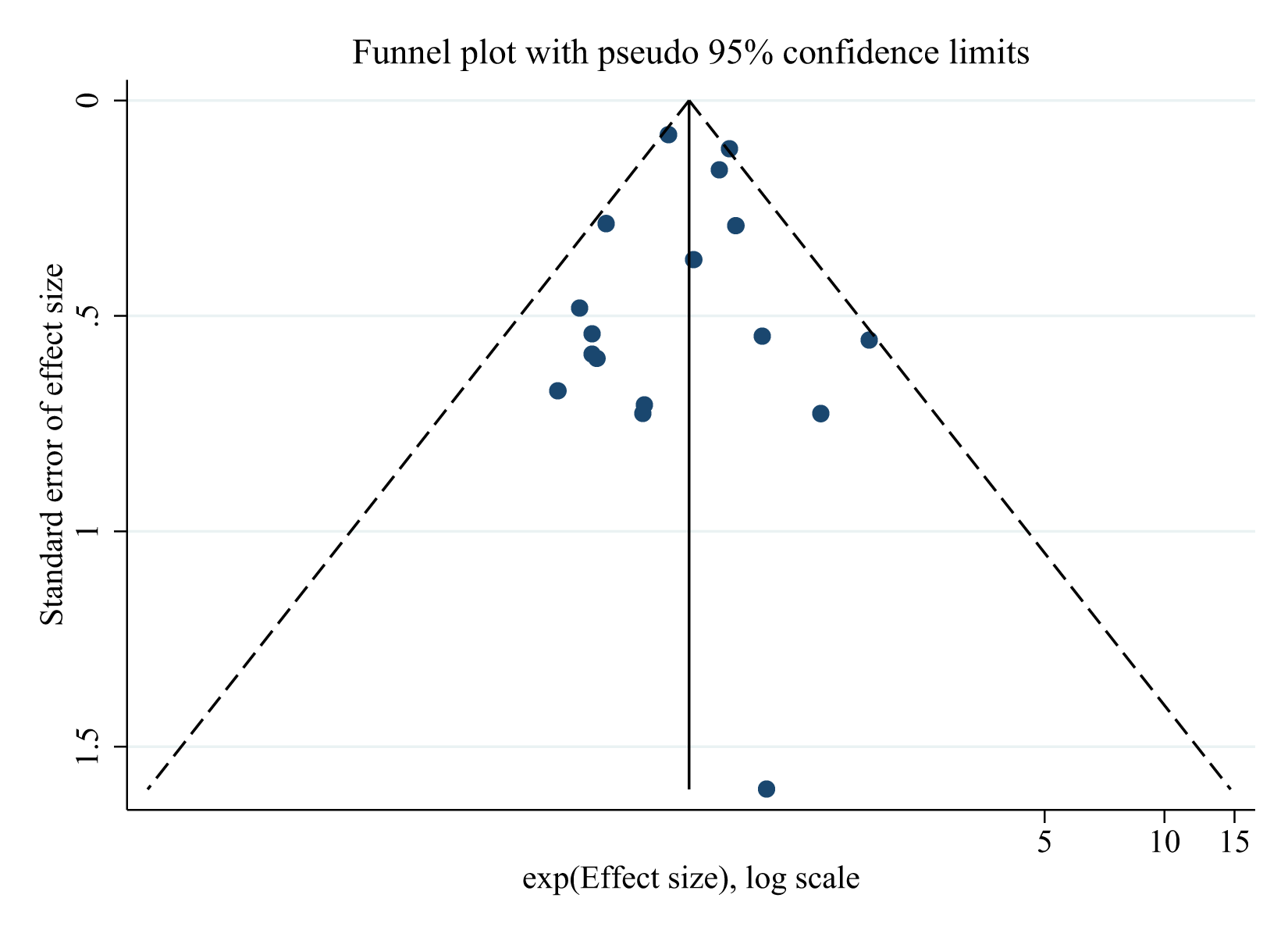


Figure C.41 Funnel plot of recurrence rate in pregnant BC patients compared with non-pregnant BC patients

**
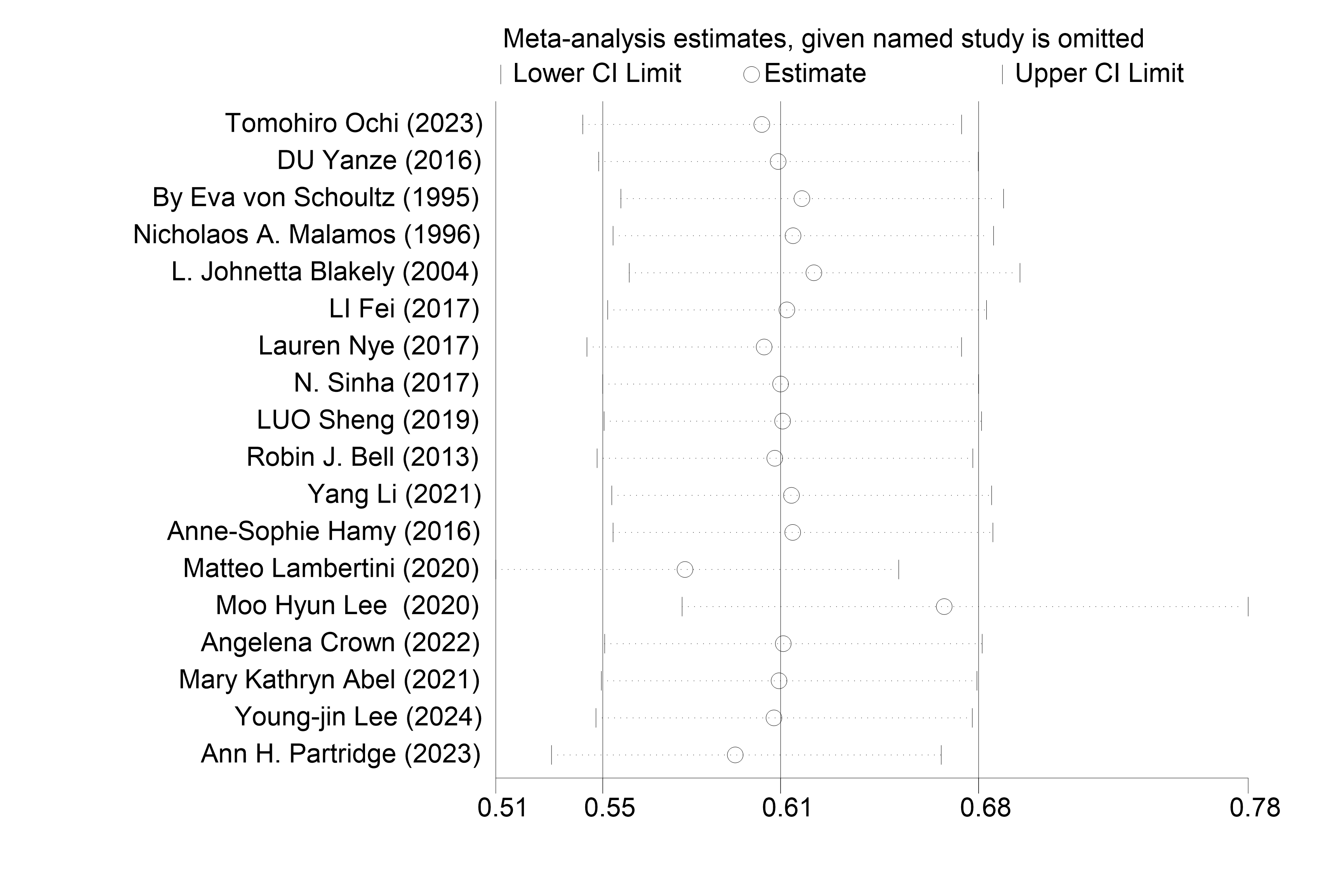
**

Figure C.42 Sensitivity analysis of recurrence rate in pregnant BC patients compared with non-pregnant BC patients


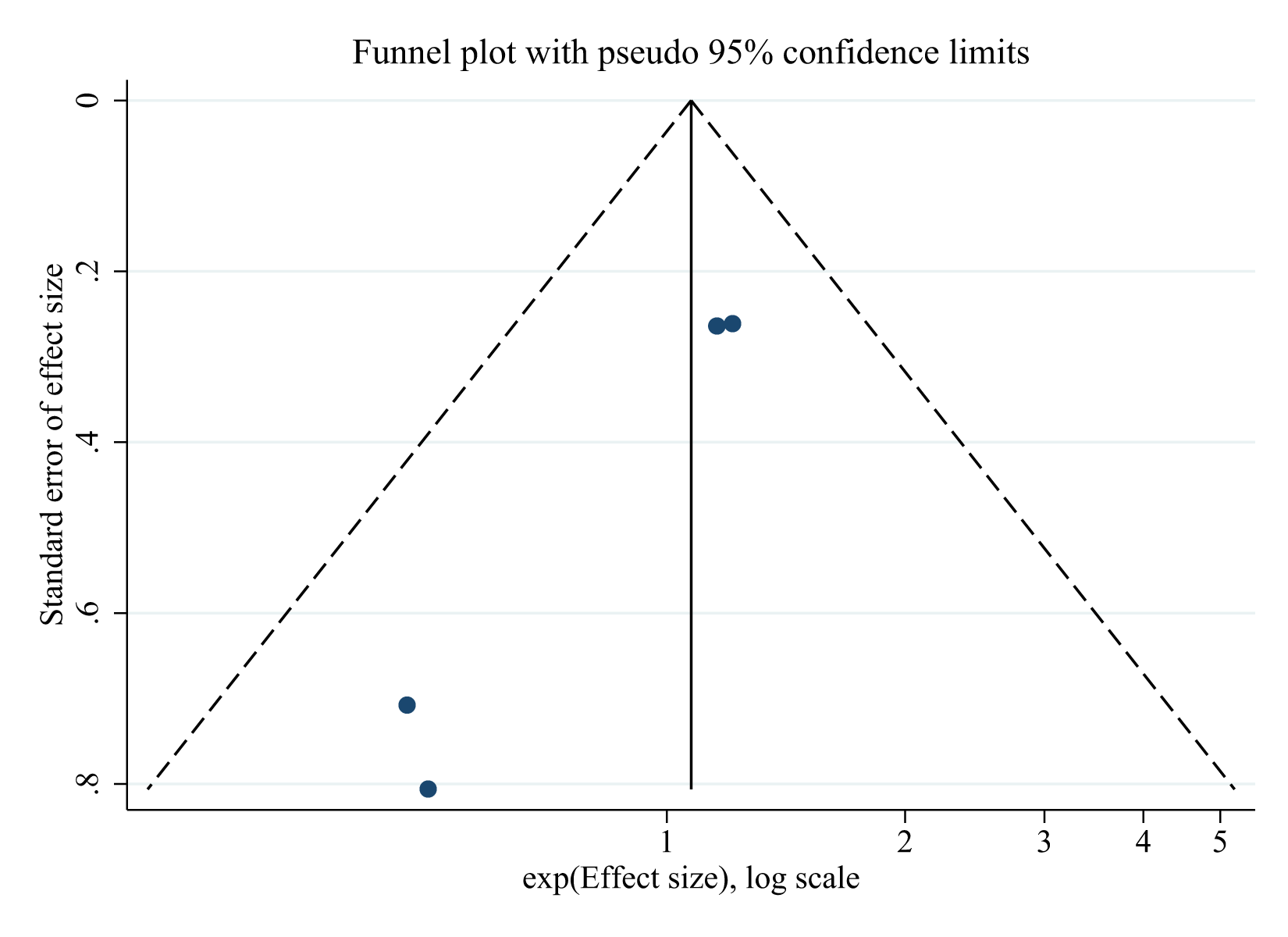


Figure C.43 Funnel plot of loco-regional recurrence rate in pregnant BC patients compared with non-pregnant BC patients


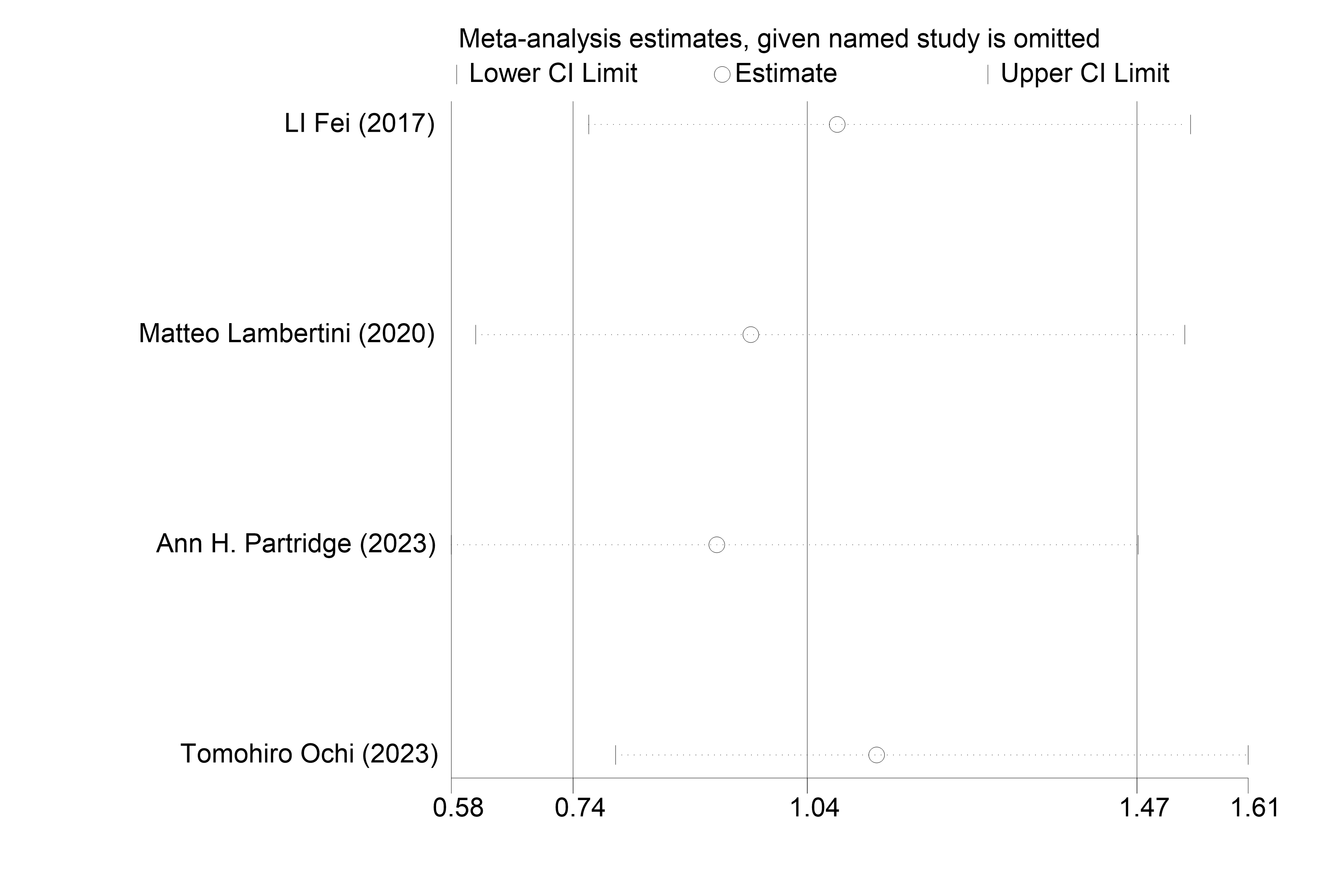


Figure C.44 Sensitivity analysis of loco-regional recurrence rate in pregnant BC patients compared with non-pregnant BC patients


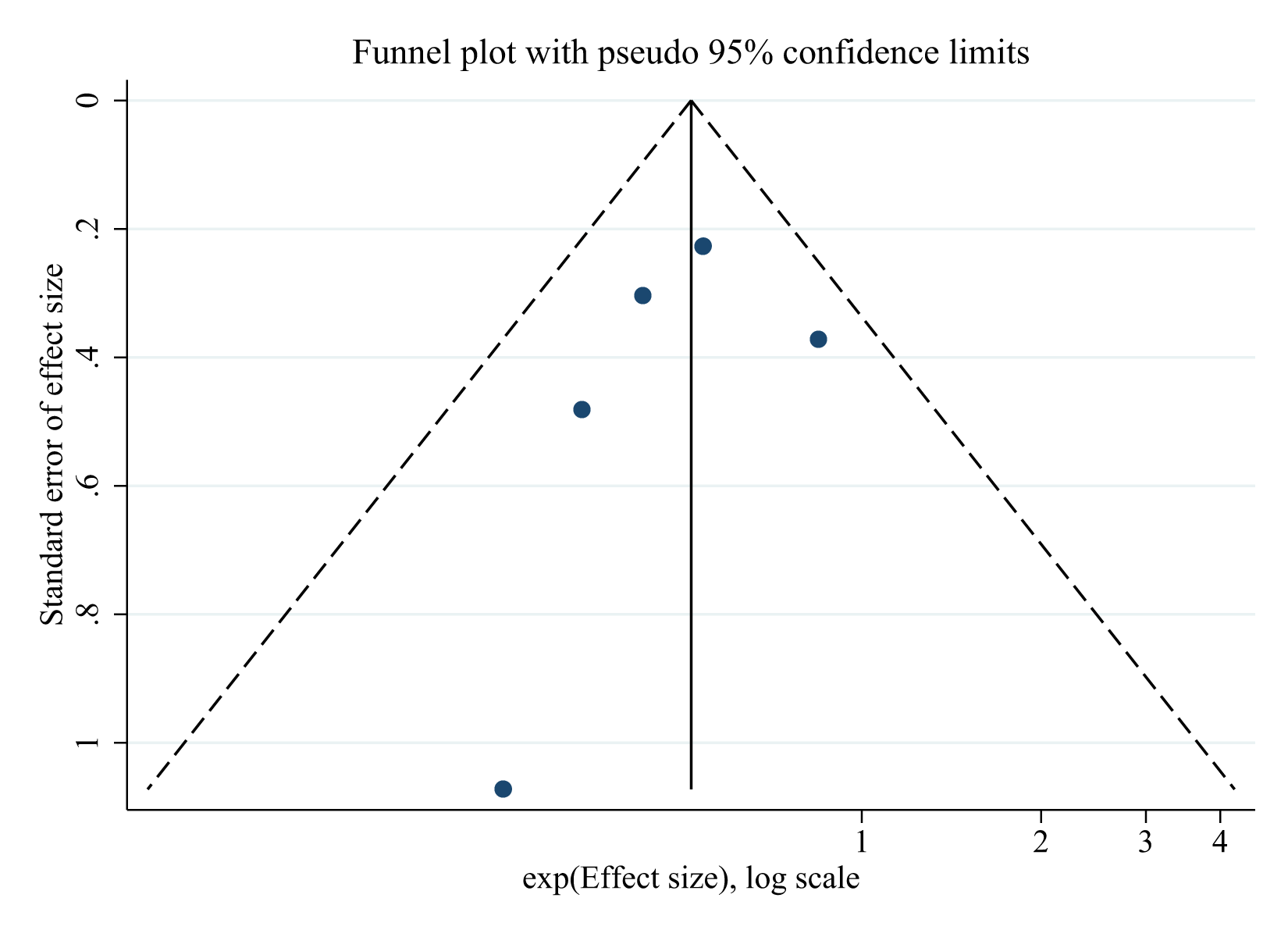


Figure C.45 Funnel plot of distant recurrence rate in pregnant BC patients compared with non-pregnant BC patients


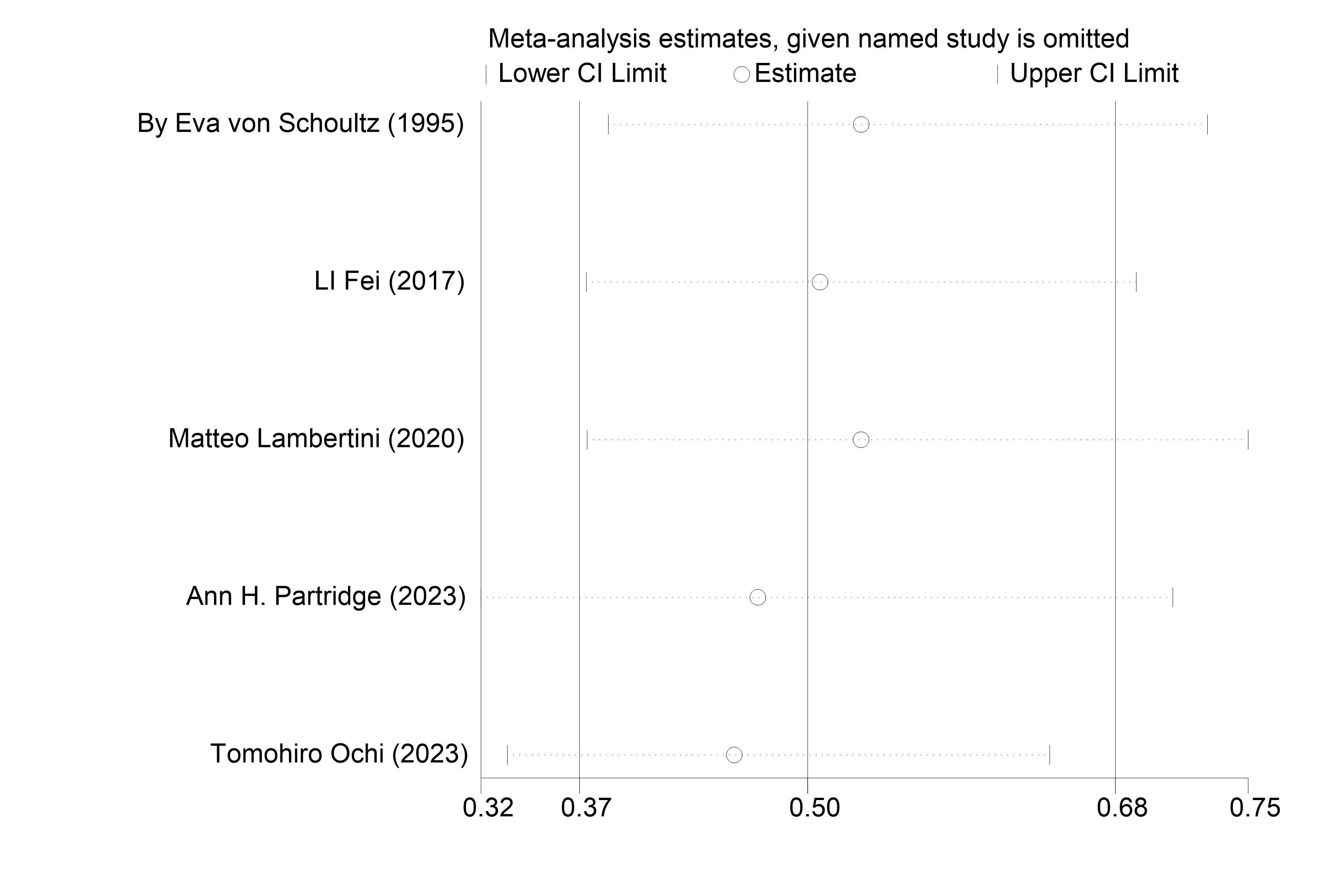


Figure C.46 Sensitivity analysis of distant recurrence rate in pregnant BC patients compared with non-pregnant BC patients


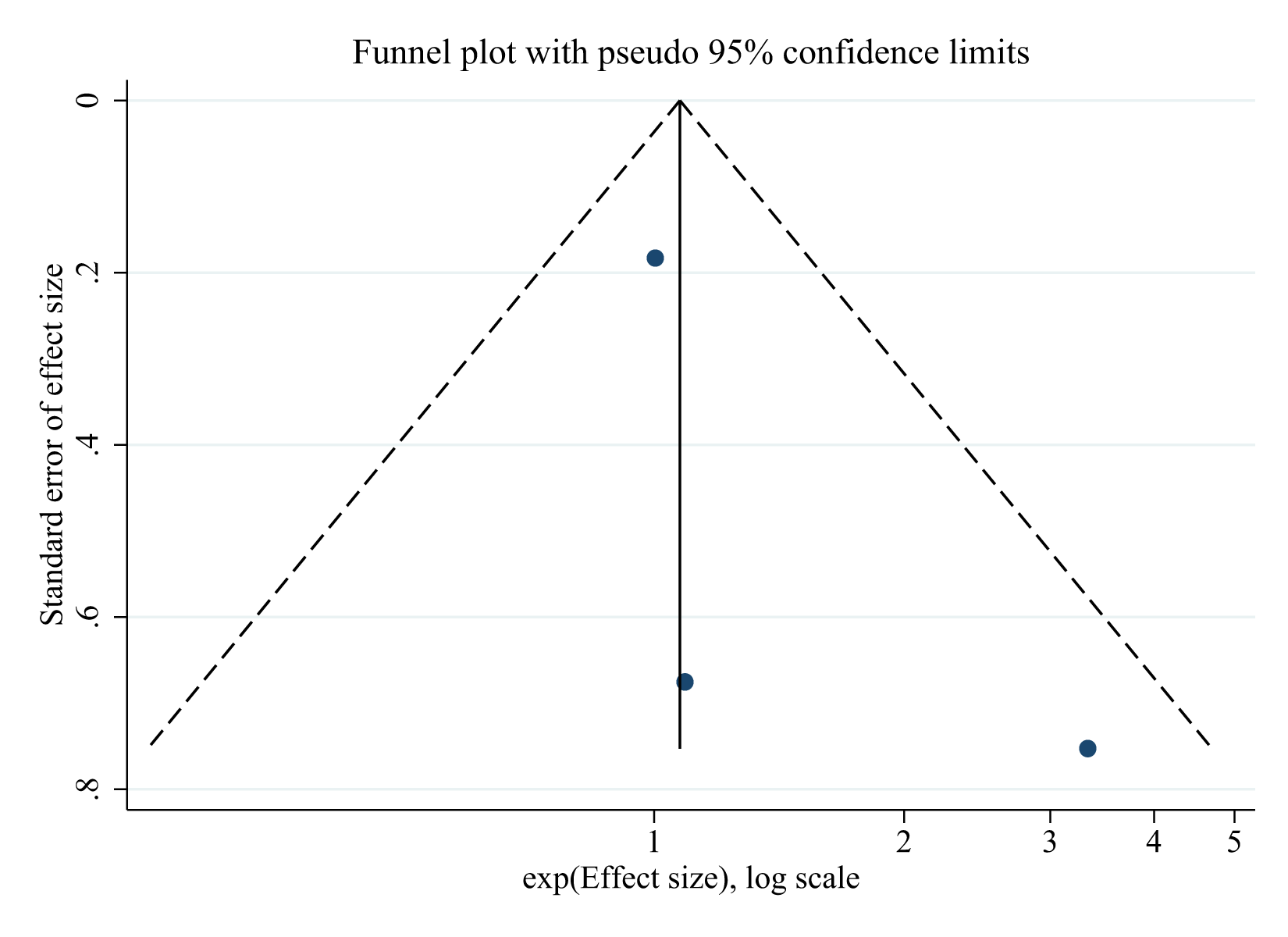


Figure C.47 Funnel plot of contralateral breast cancer rate in pregnant BC patients compared with non-pregnant BC patients


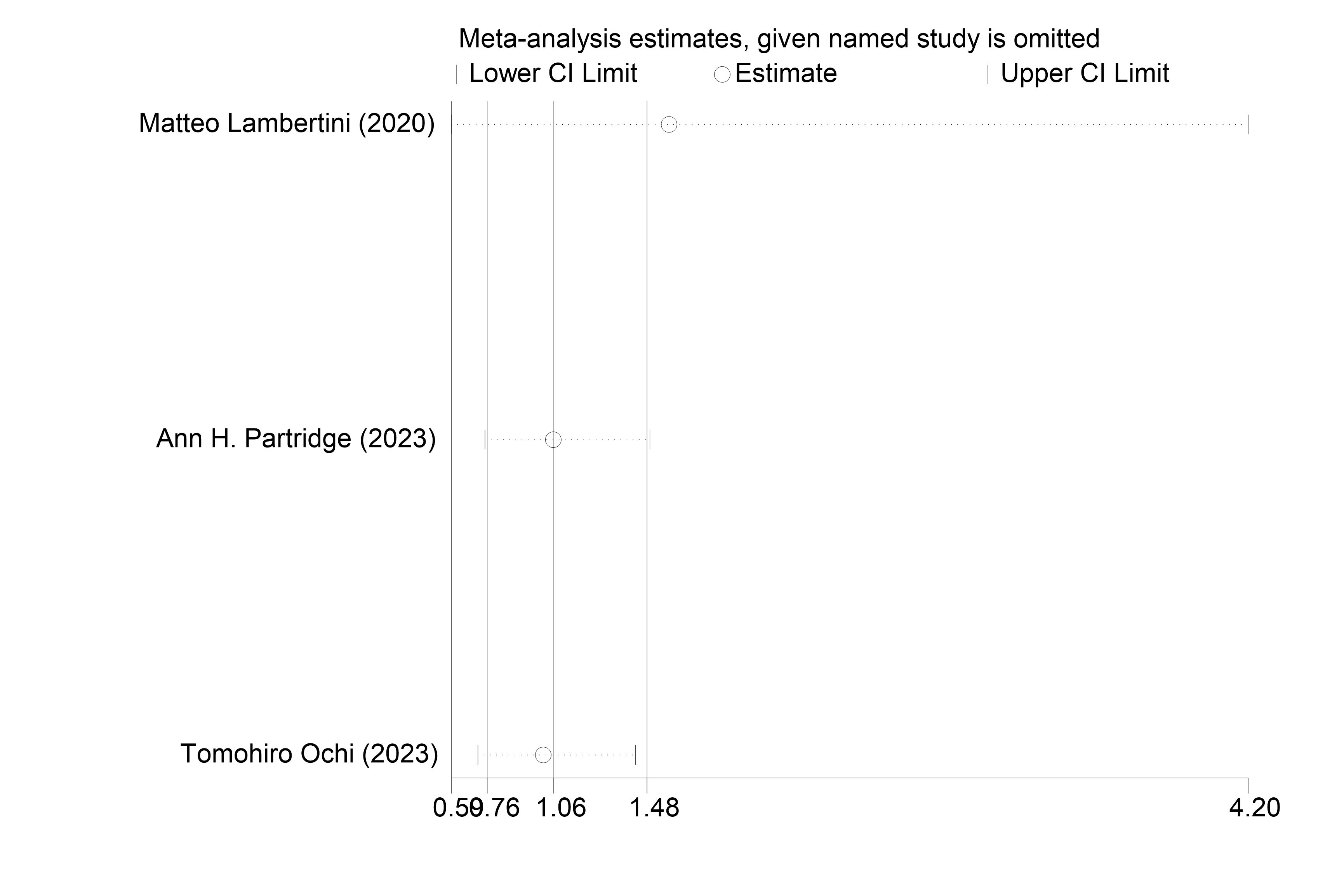


Figure C.48 Sensitivity analysis of contralateral breast cancer rate in pregnant BC patients compared with non-pregnant BC patients


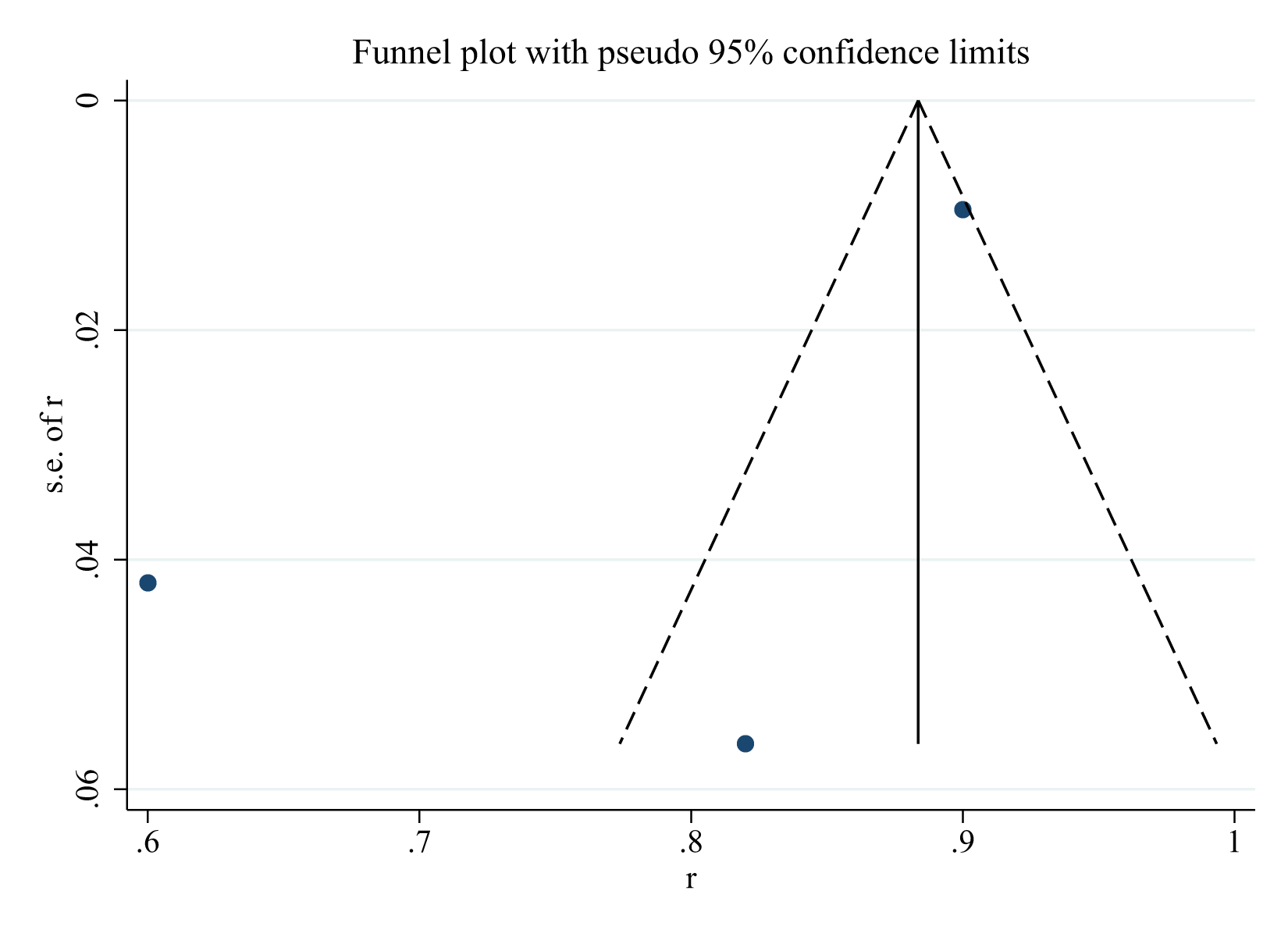


Figure C.49 Funnel plot of 5-year relapse-free survival rate in pregnant BC patients

**
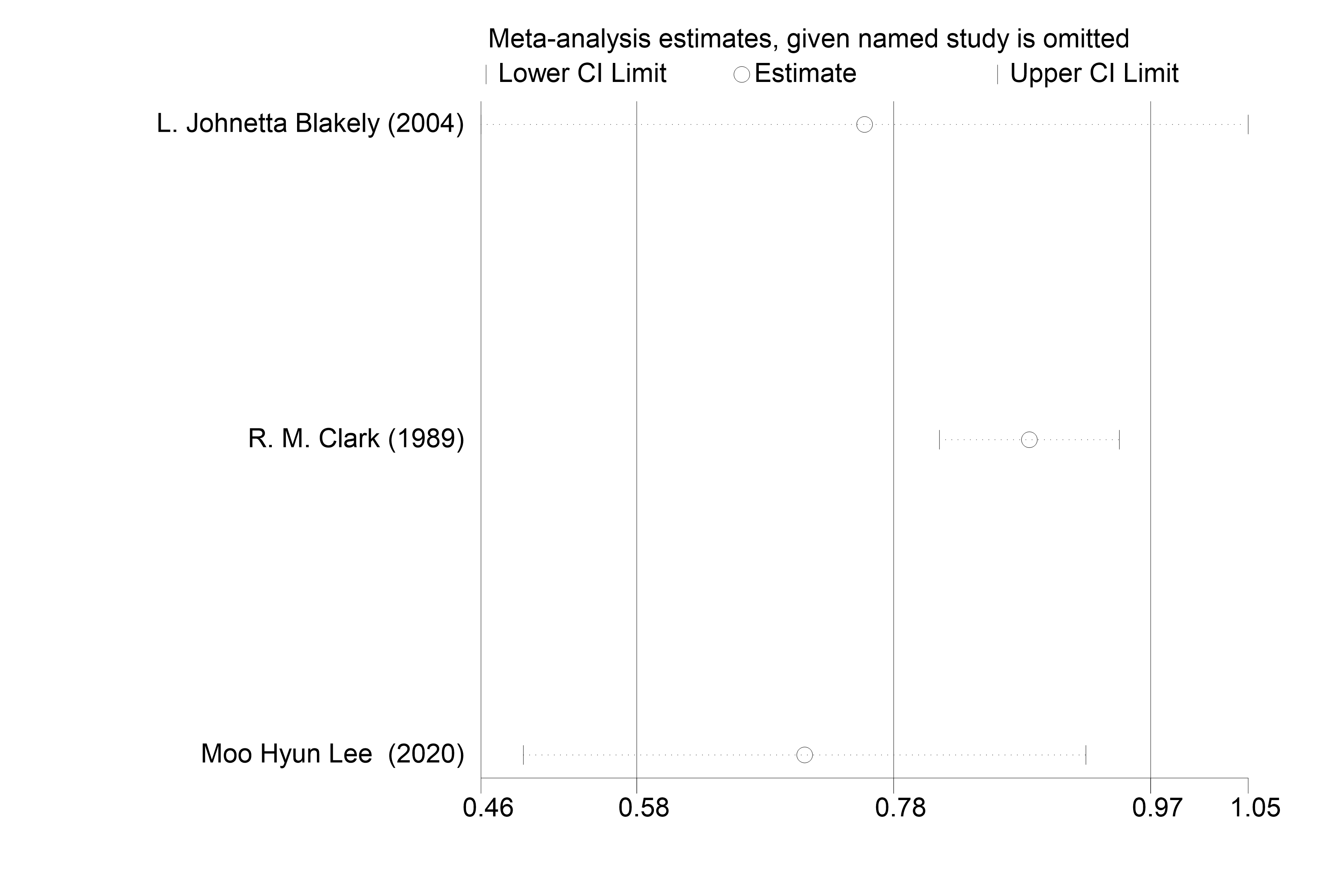
**

Figure C.50 Sensitivity analysis of 5-year relapse-free survival rate in pregnant BC patients


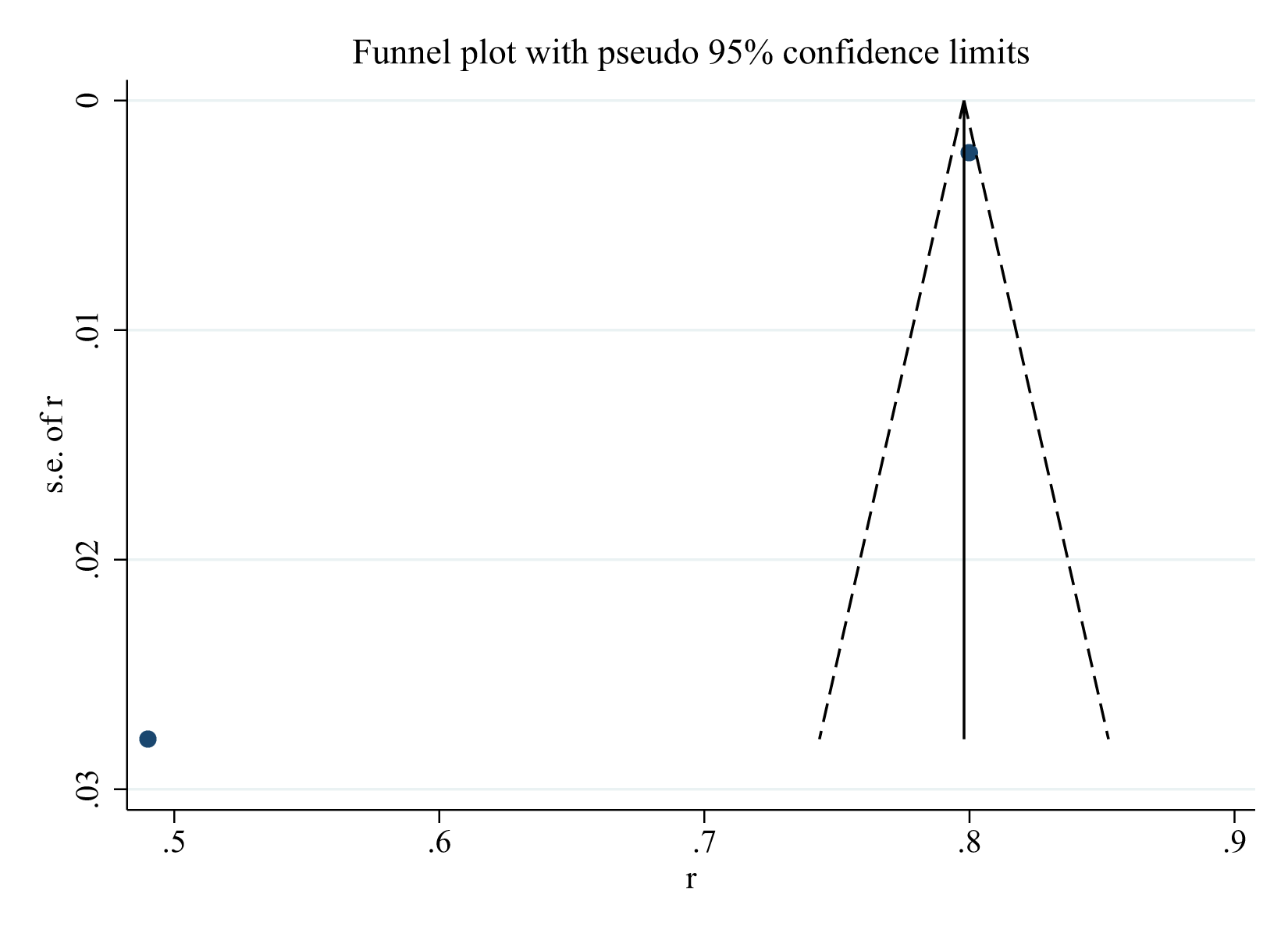


Figure C.51 Funnel plot of 5-year relapse-free survival rate in non-pregnant BC patients


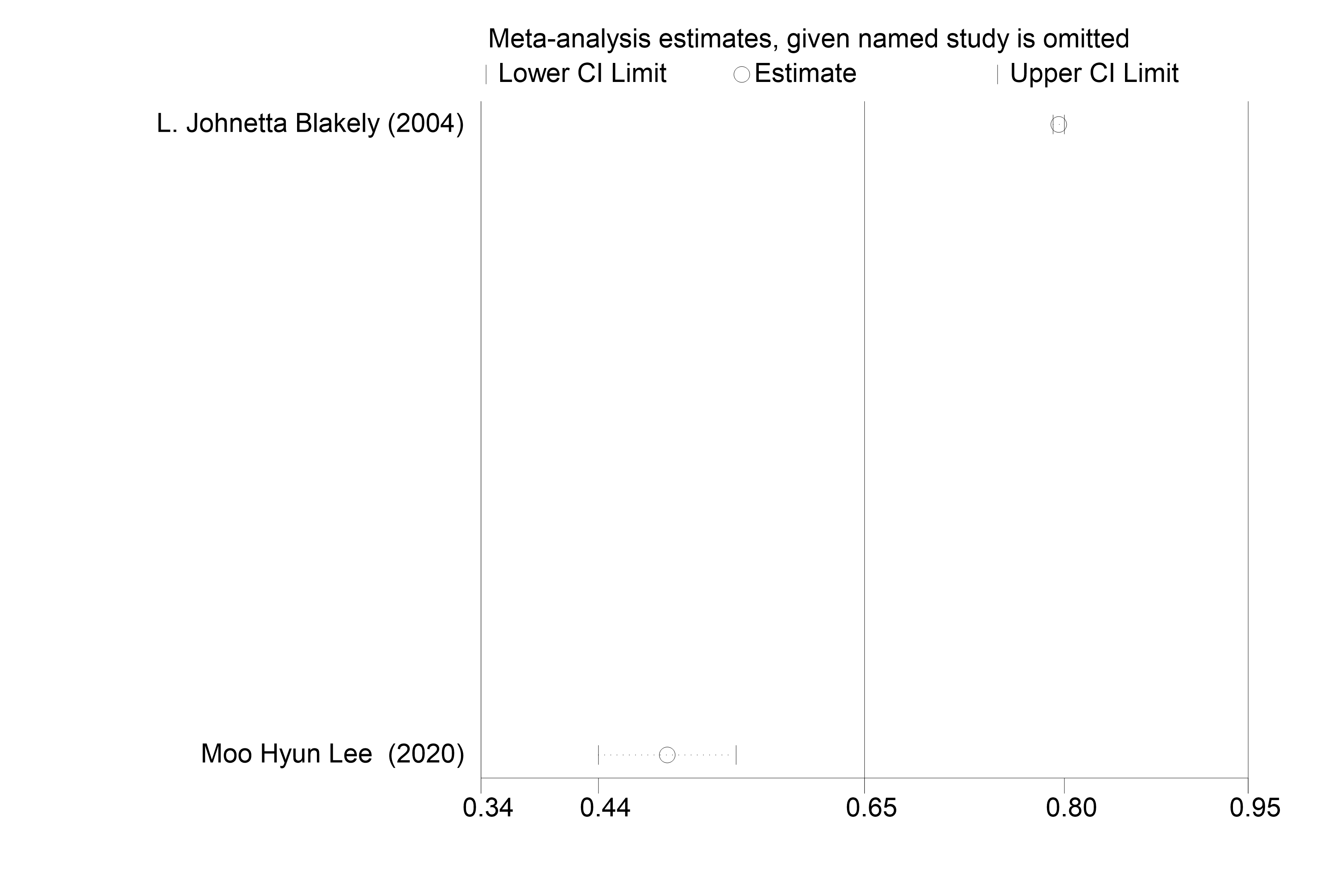


Figure C.52 Sensitivity analysis of 5-year relapse-free survival rate in non-pregnant BC patients


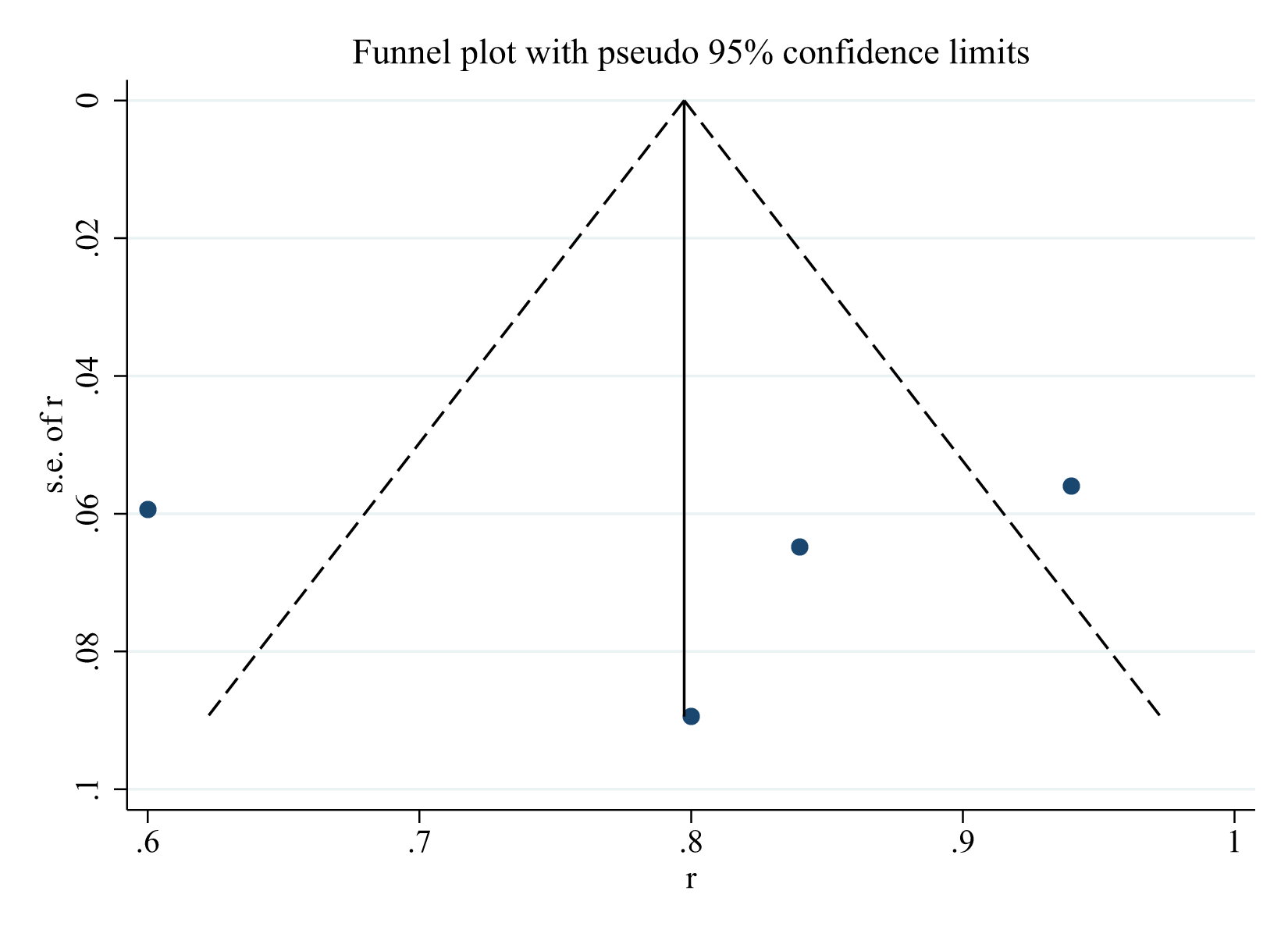


Figure C.53 Funnel plot of 5-year disease-free survival rate in pregnant BC patients


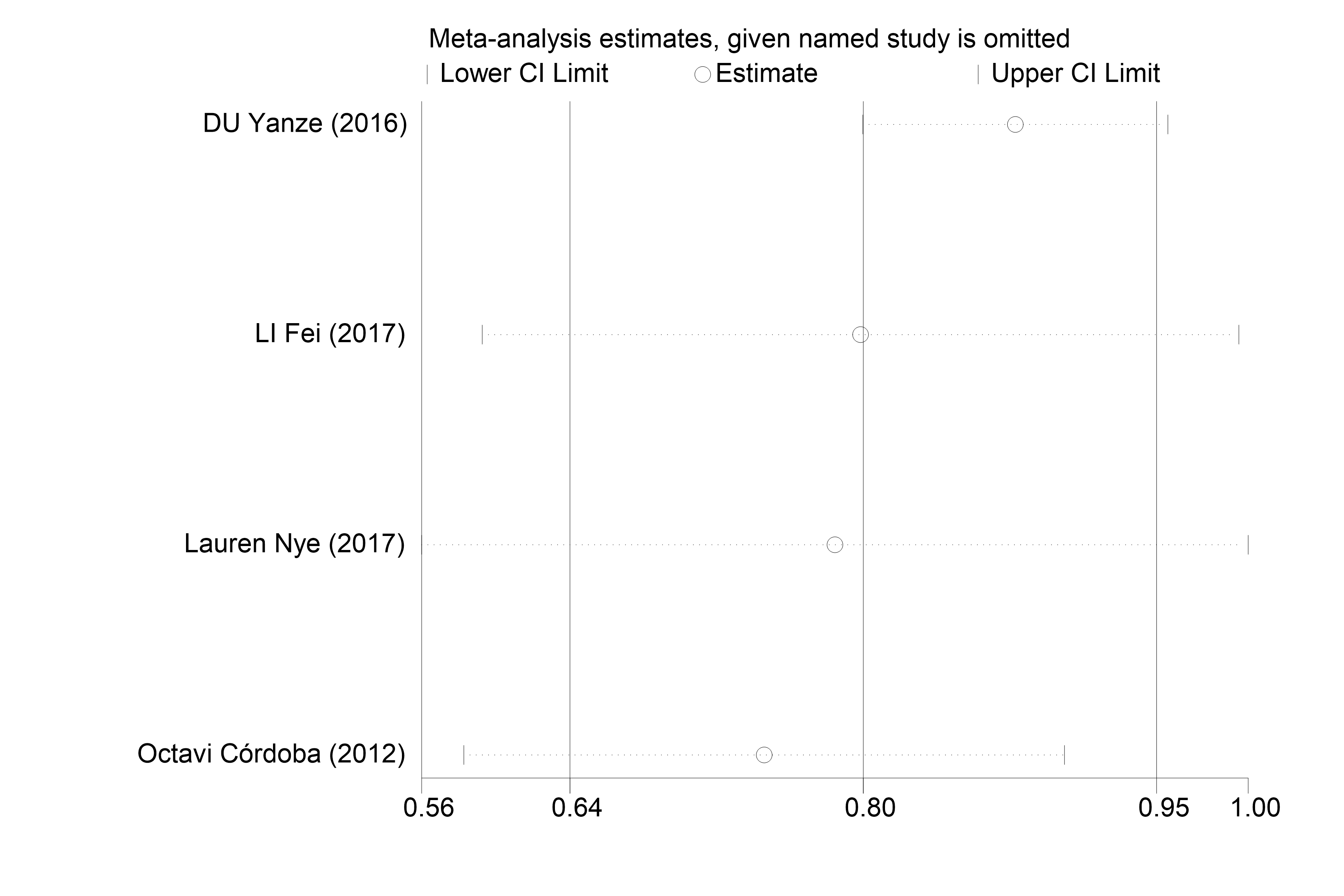


Figure C.54 Sensitivity analysis of 5-year disease-free survival rate in pregnant BC patients


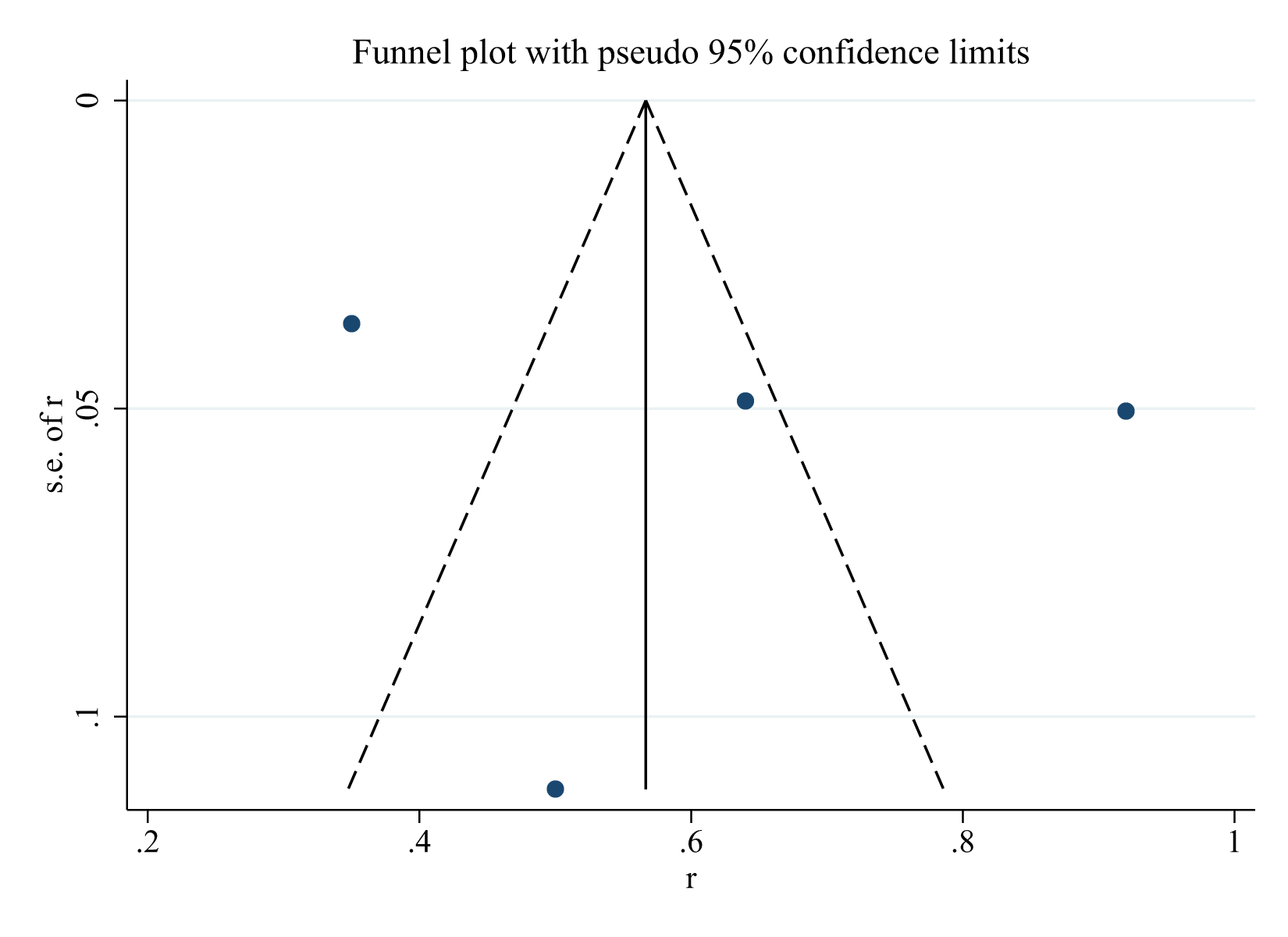


Figure C.55 Funnel plot of 5-year disease-free survival rate in non-pregnant BC patients

**
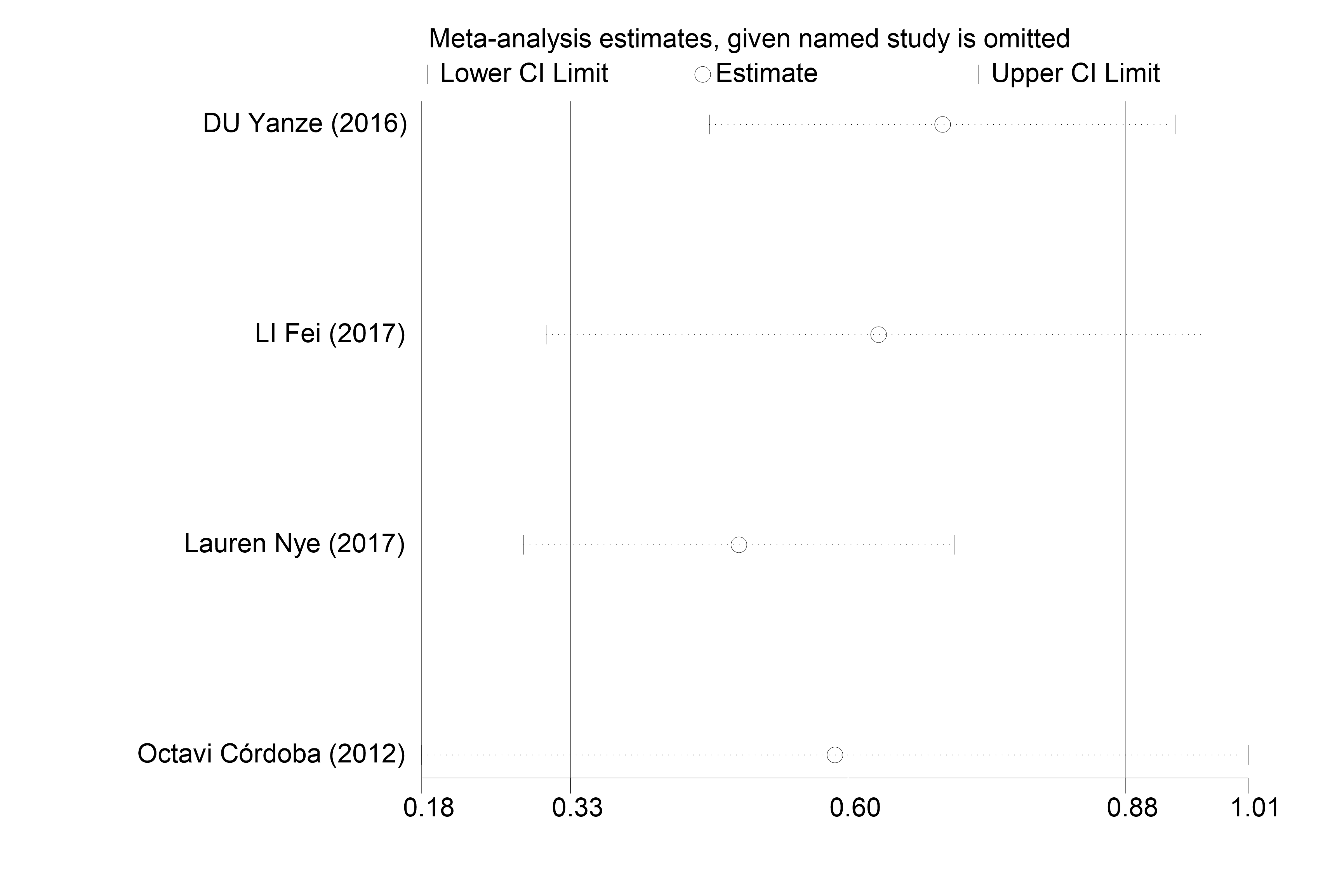
**

Figure C.56 Sensitivity analysis of 5-year disease-free survival rate in non-pregnant BC patients


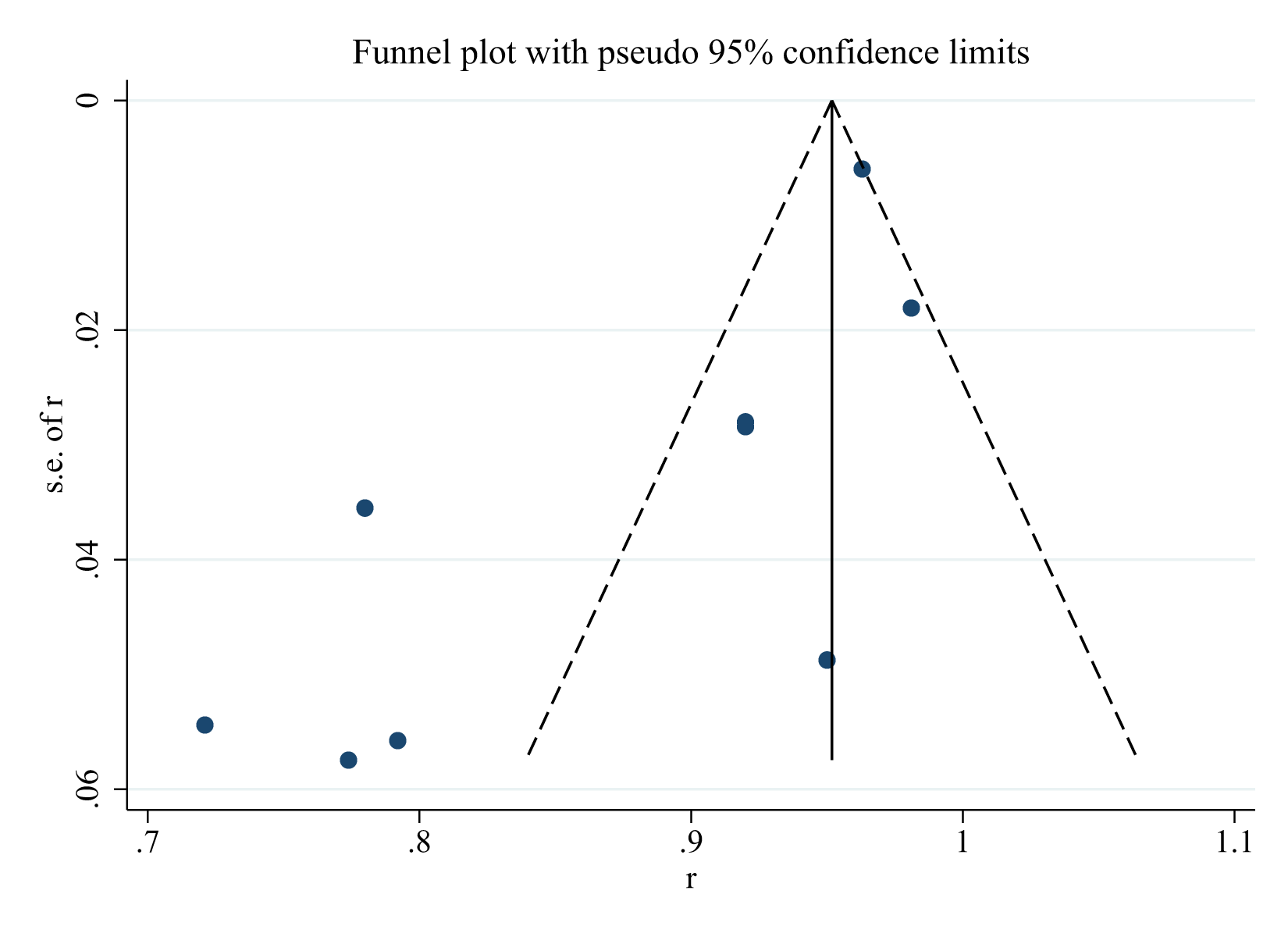


Figure C.57 Funnel plot of 5-year survival rate in pregnant BC patients


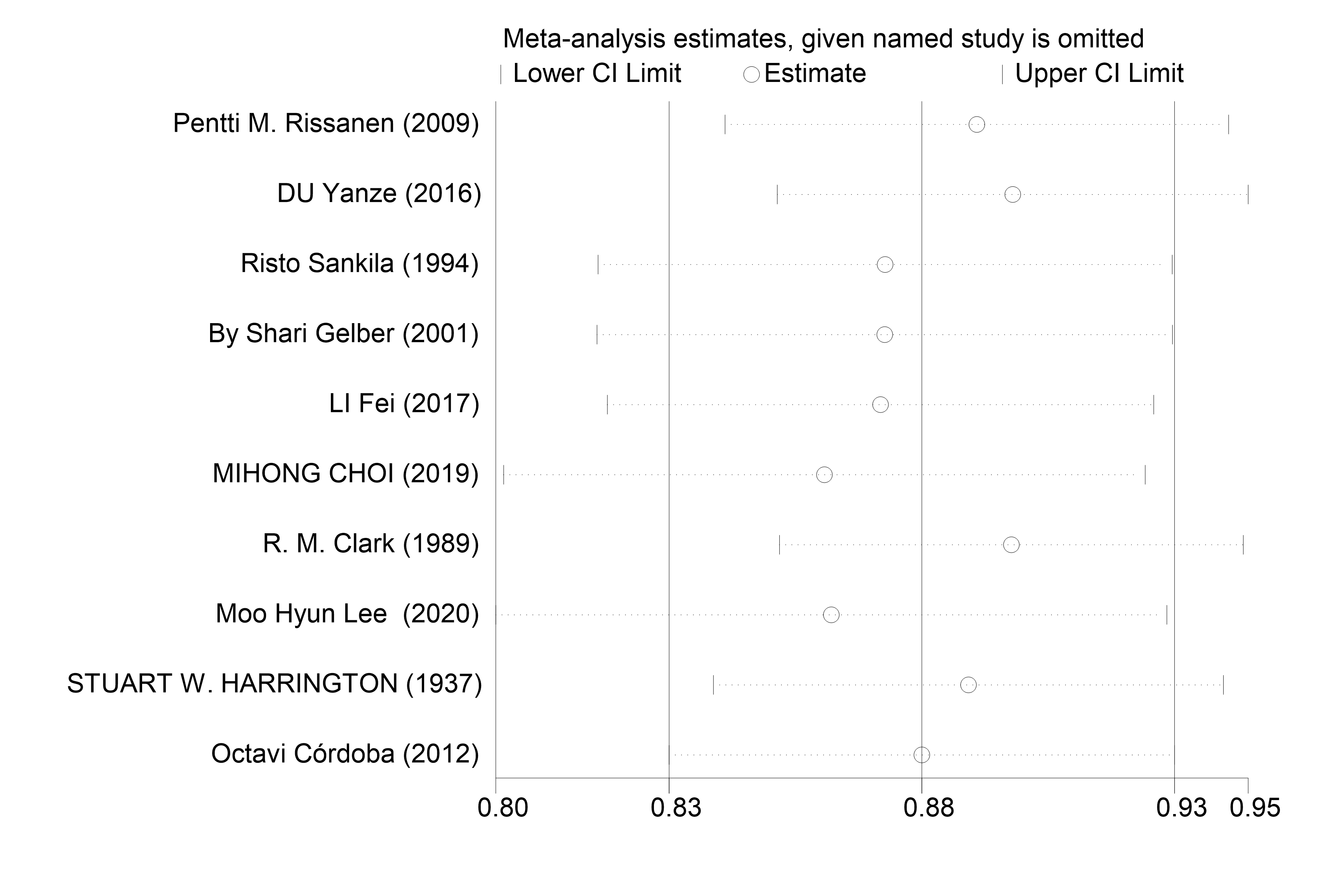


Figure C.58 Sensitivity analysis of 5-year survival rate in pregnant BC patients


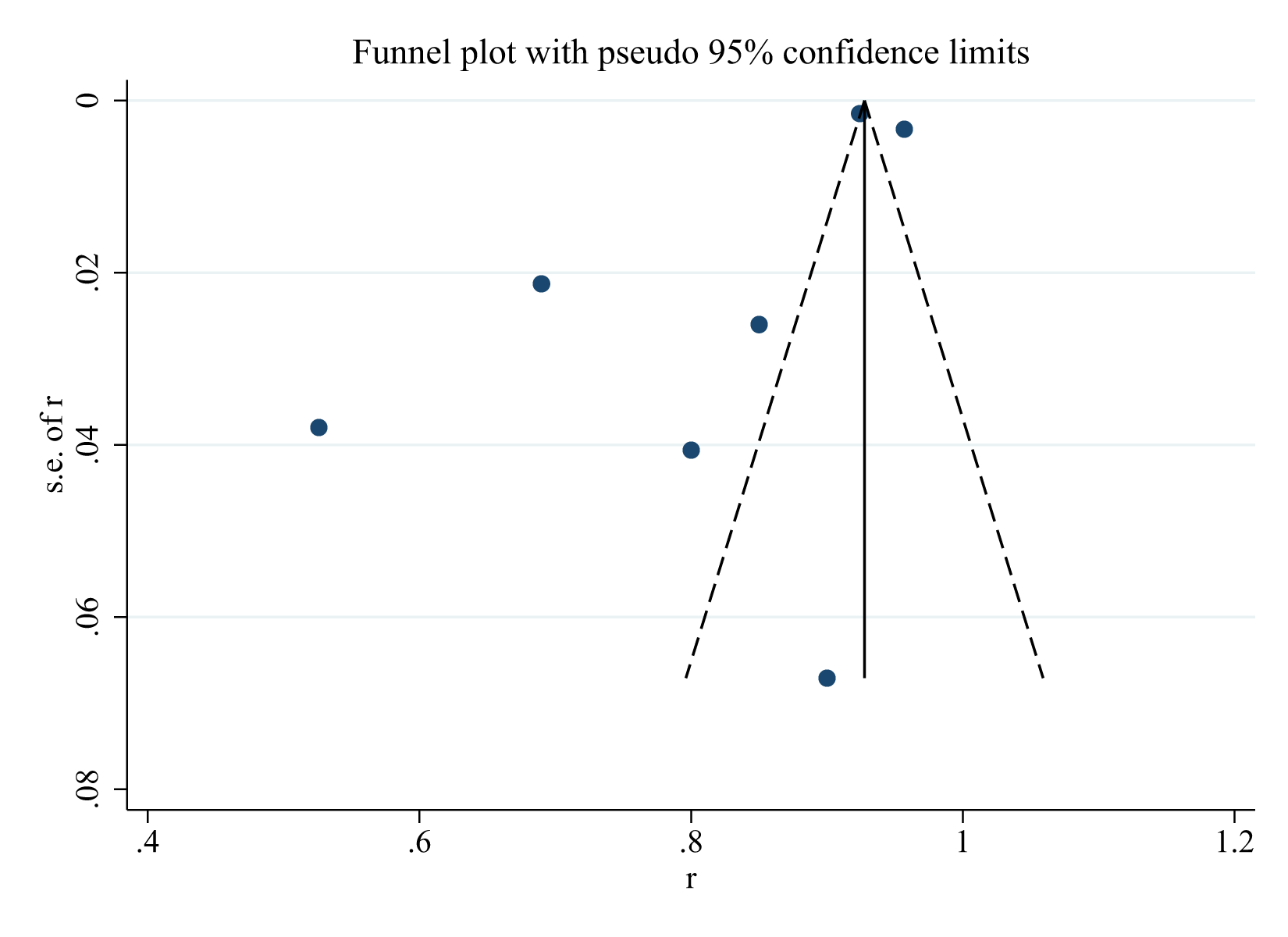


Figure C.59 Funnel plot of 5-year survival rate in non-pregnant BC patients


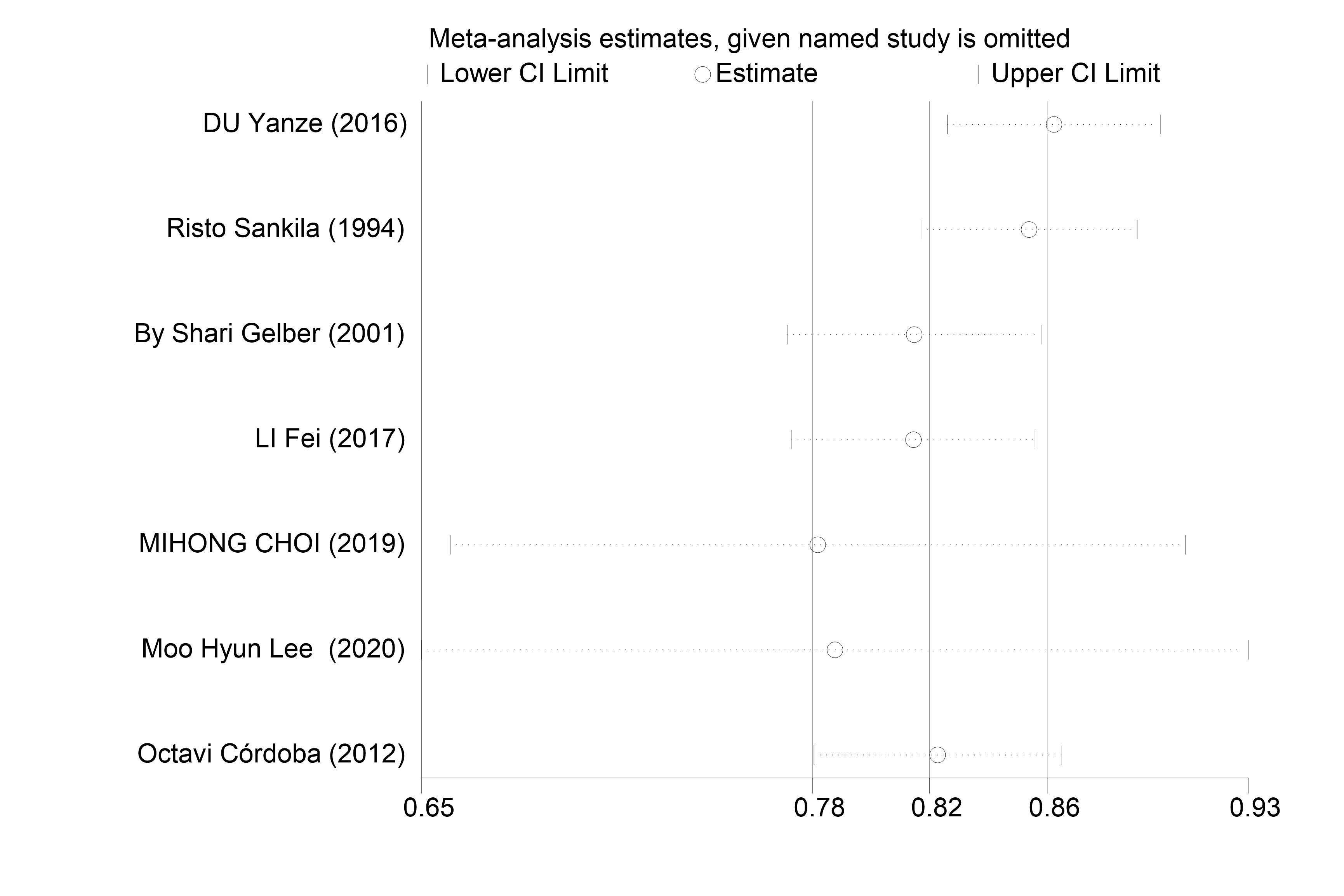


Figure C.60 Sensitivity analysis of 5-year survival rate in non-pregnant BC patients


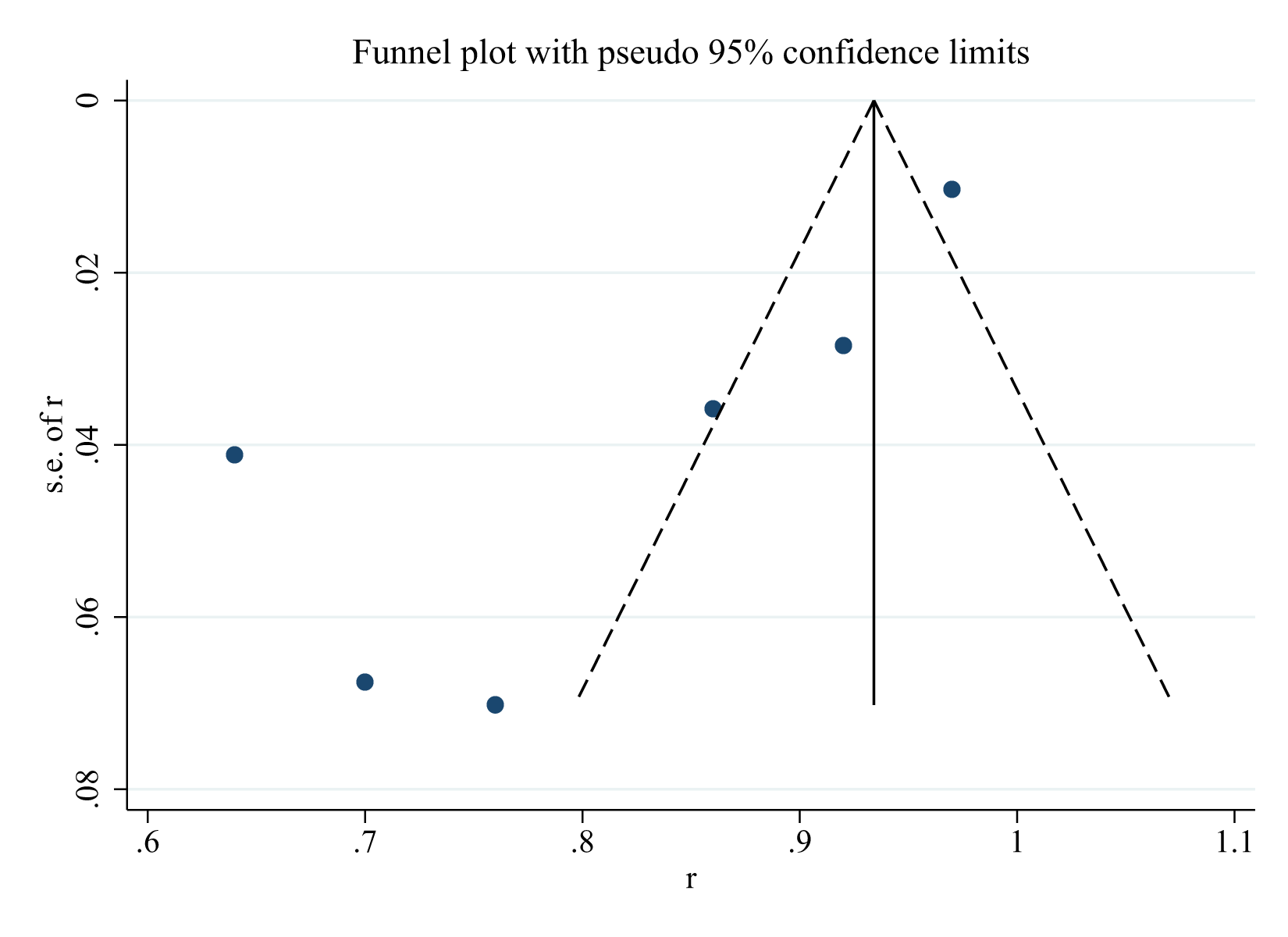


Figure C.61 Funnel plot of 10-year survival rate in pregnant BC patients


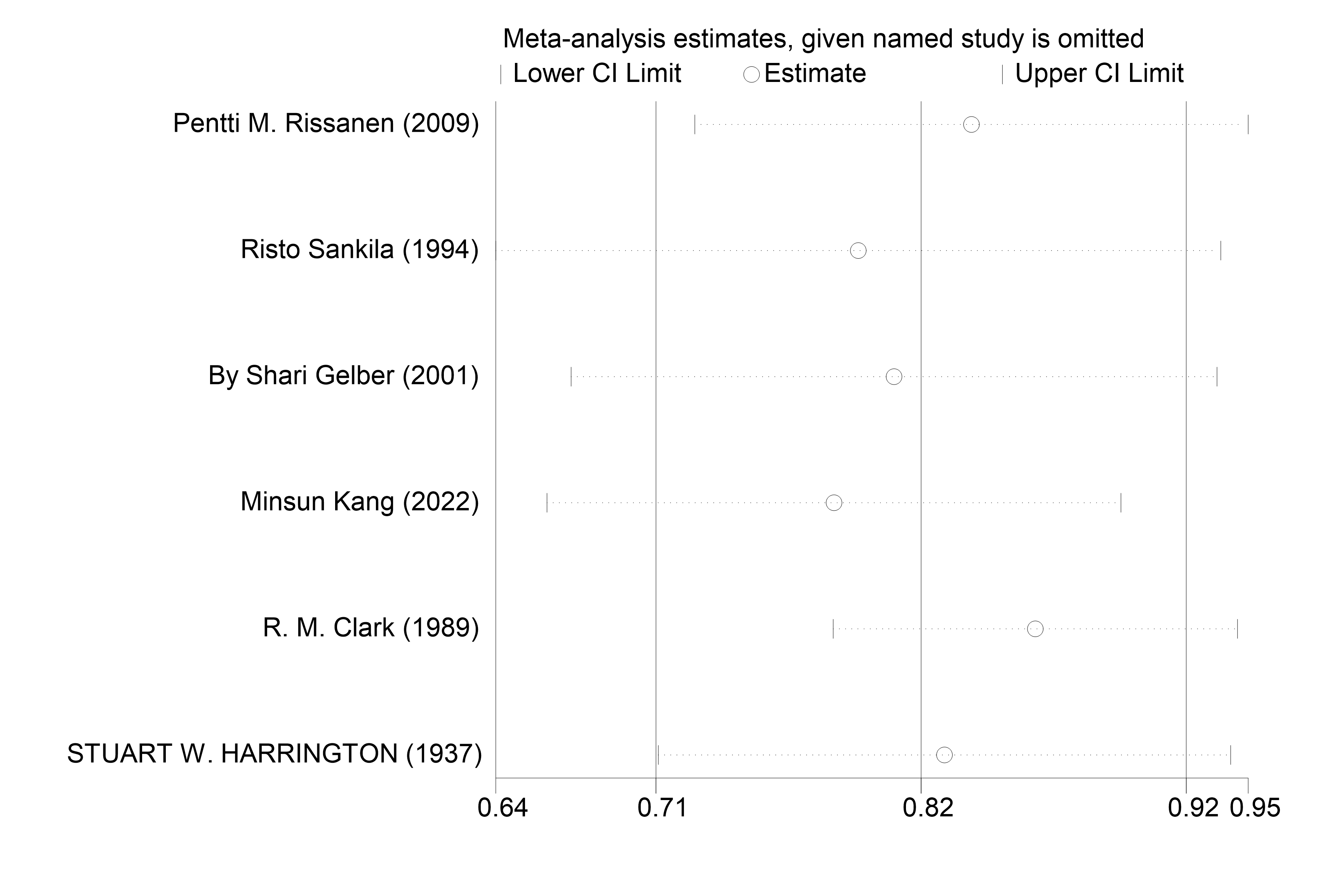


Figure C.62 Sensitivity analysis of 10-year survival rate in pregnant BC patients


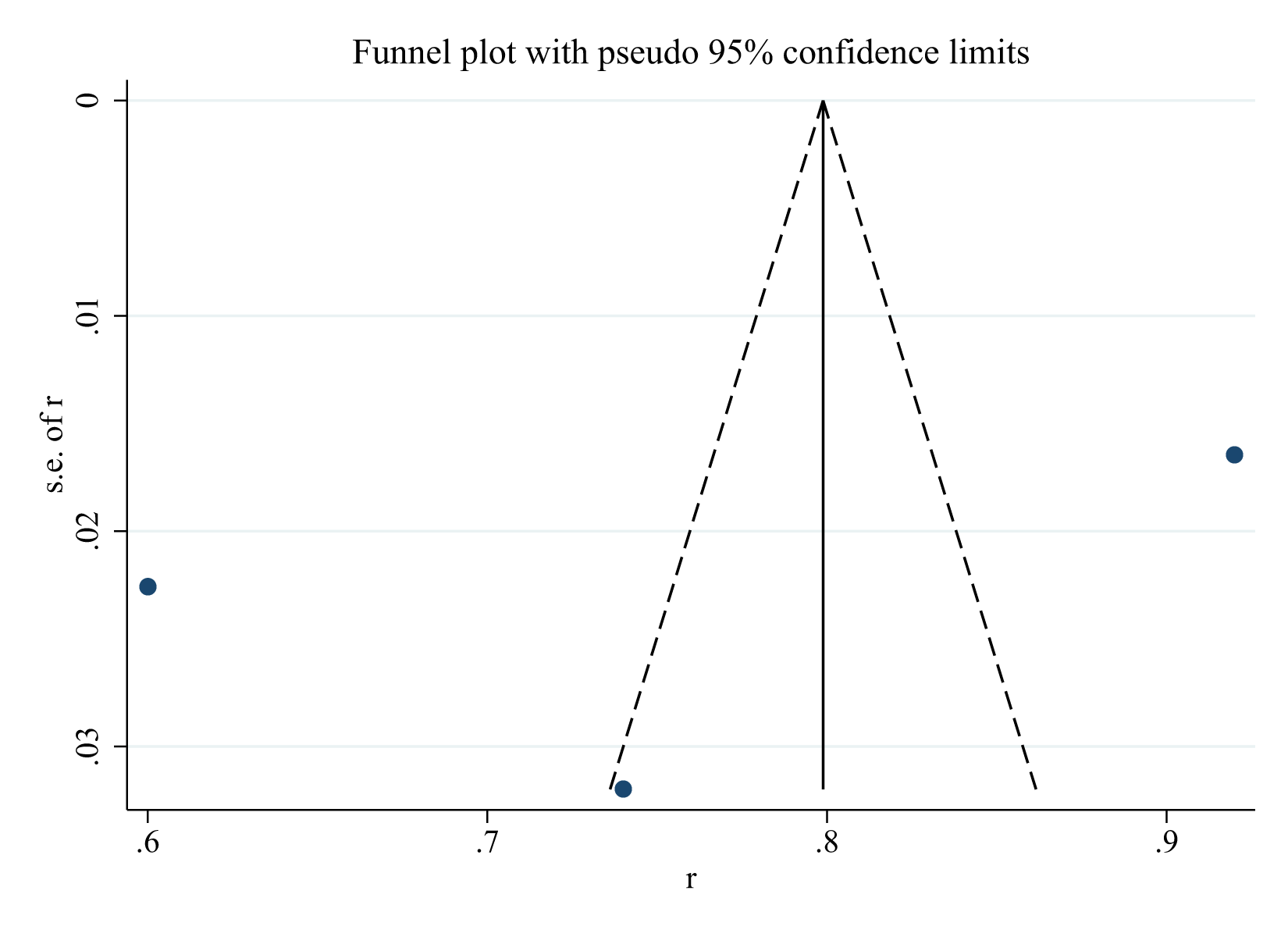


Figure C.63 Funnel plot of 10-year survival rate in non-pregnant BC patients


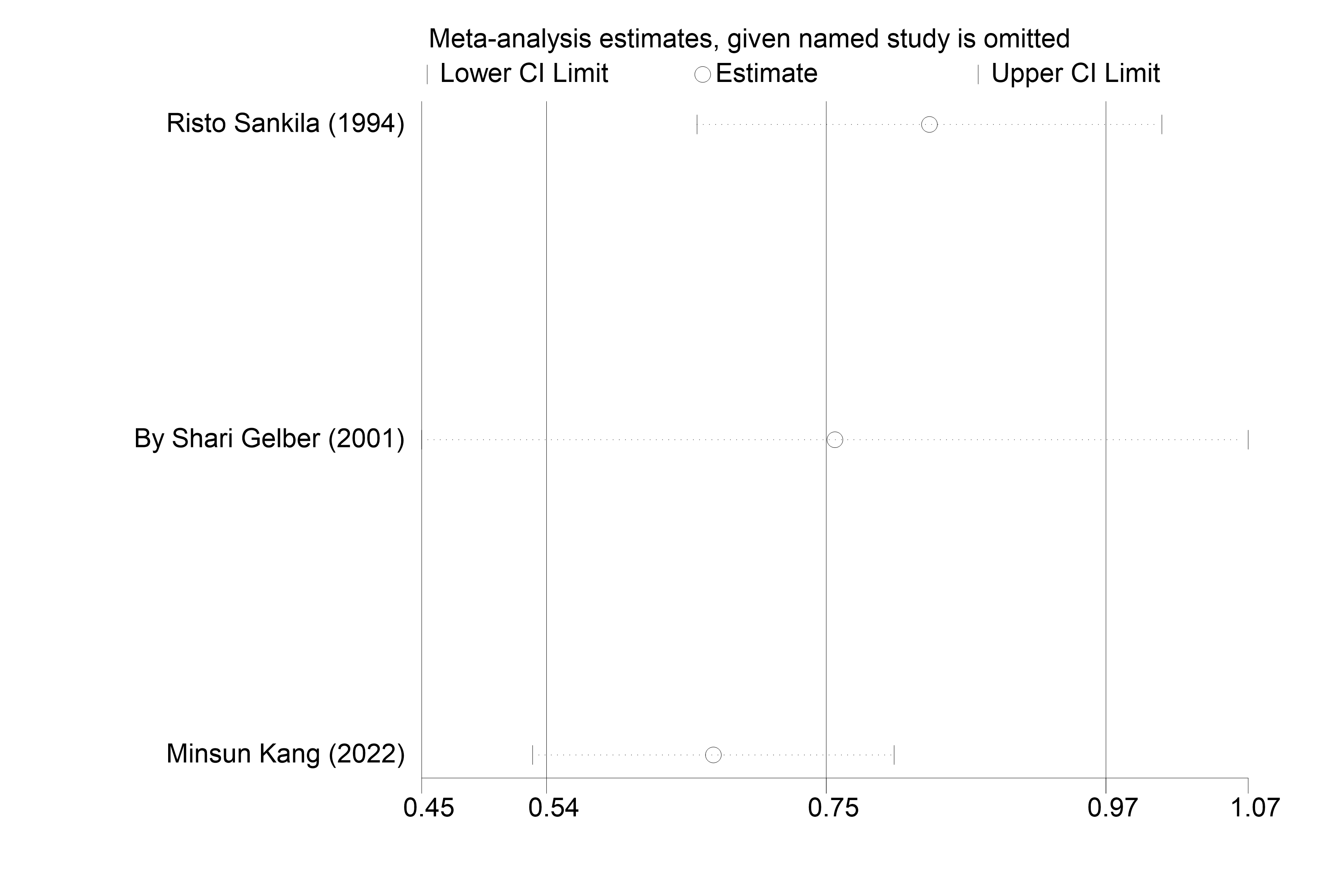


Figure C.64 Sensitivity analysis of 10-year survival rate in non-pregnant BC patients


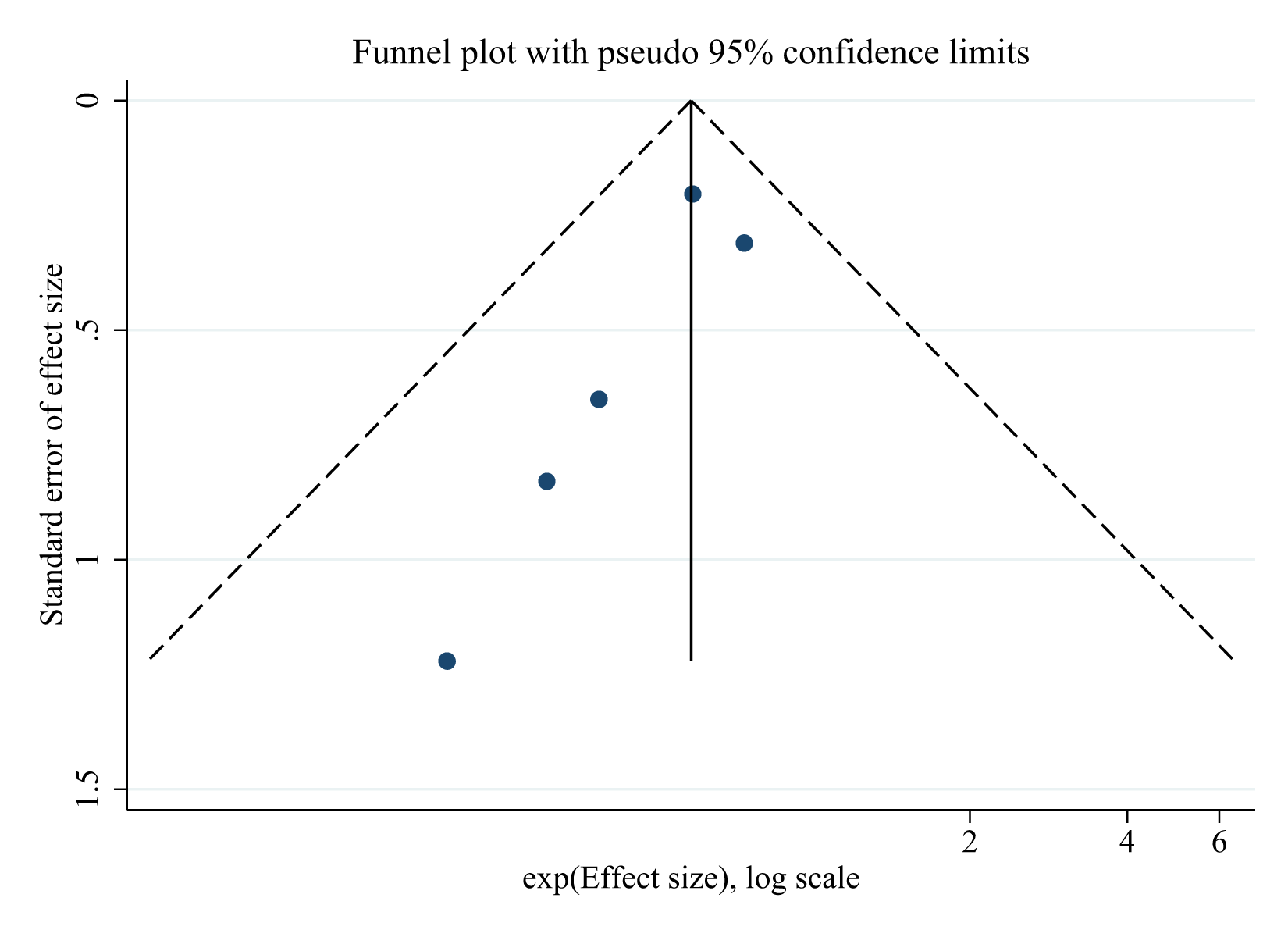


Figure C.65 Funnel plot of overall survival in stage Ⅰ pregnant BC patients compared with non-pregnant BC patients


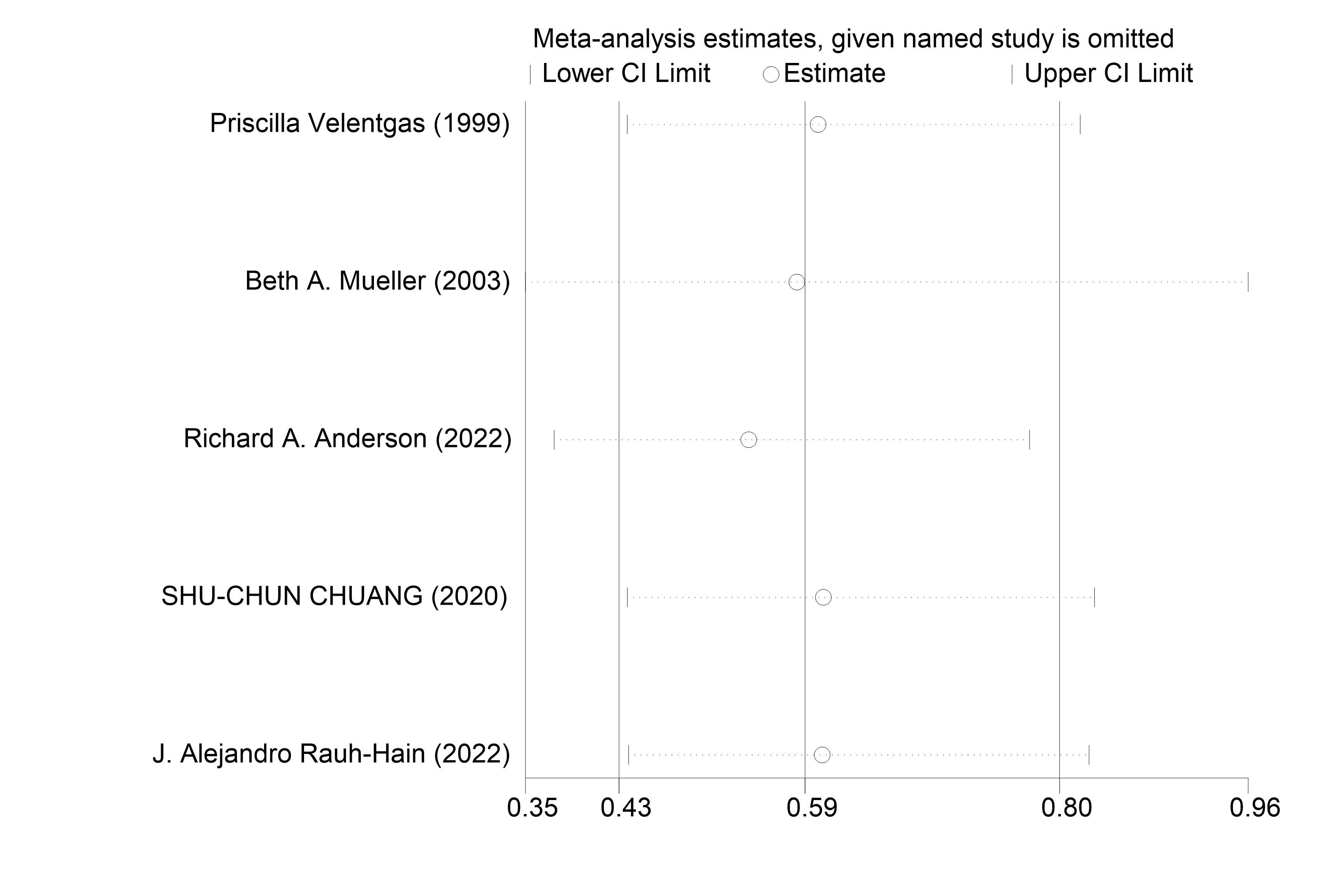


Figure C.66 Sensitivity analysis of overall survival in stage Ⅰ pregnant BC patients compared with non-pregnant BC patients


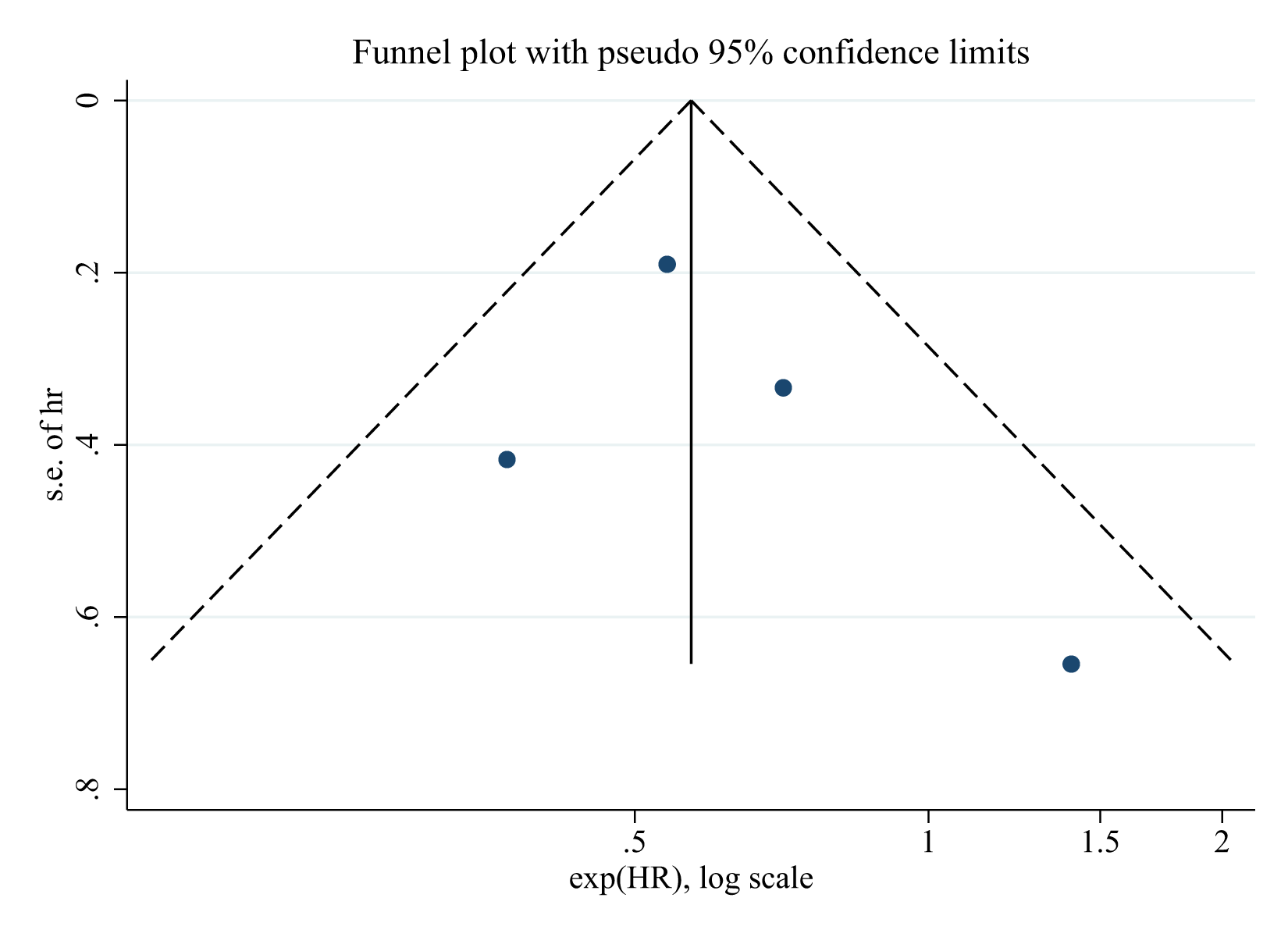


Figure C.67 Funnel plot of overall survival in stage II and III pregnant BC patients compared with non-pregnant BC patients


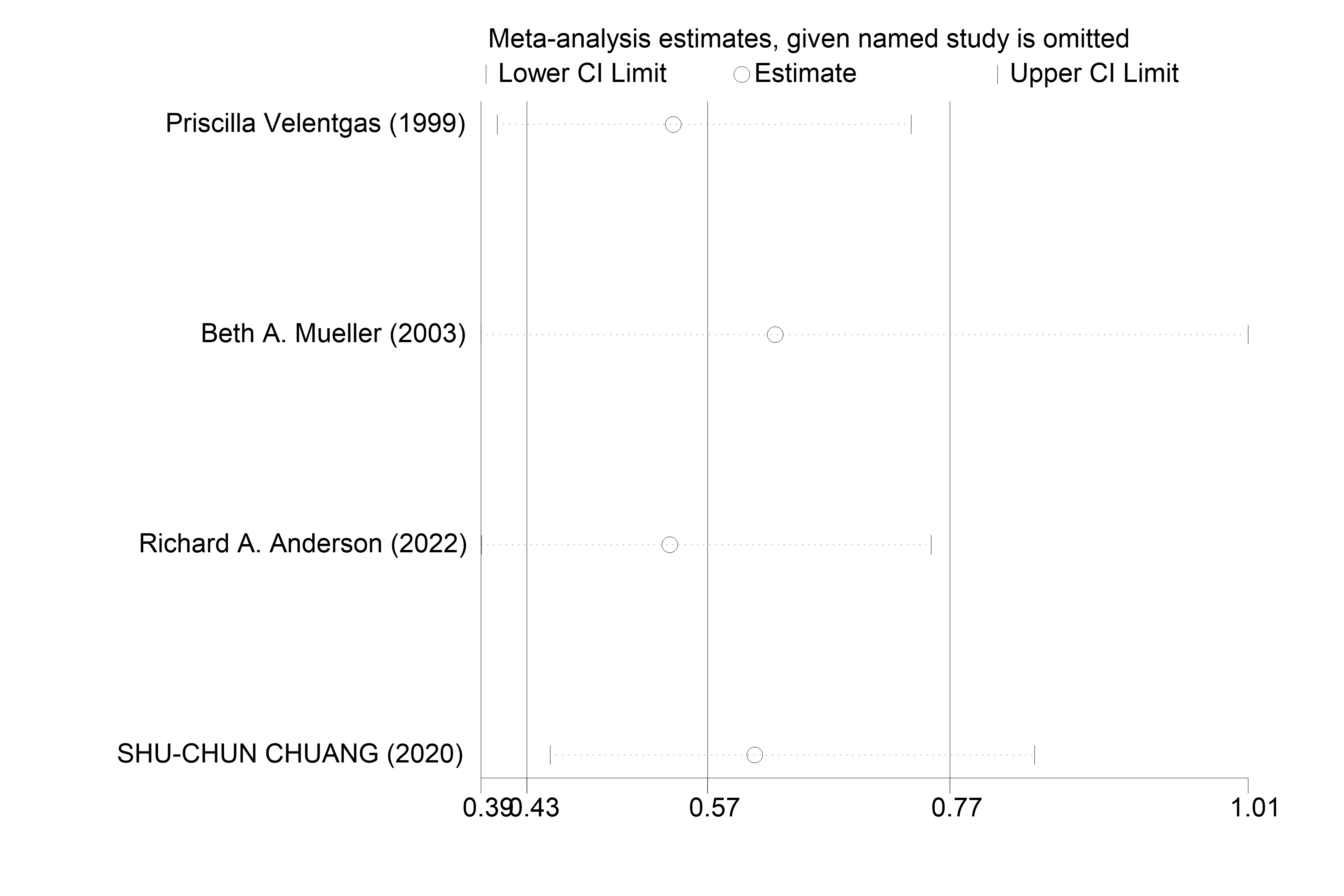


Figure C.68 Sensitivity analysis of overall survival in stage II and III pregnant BC patients compared with non-pregnant BC patients


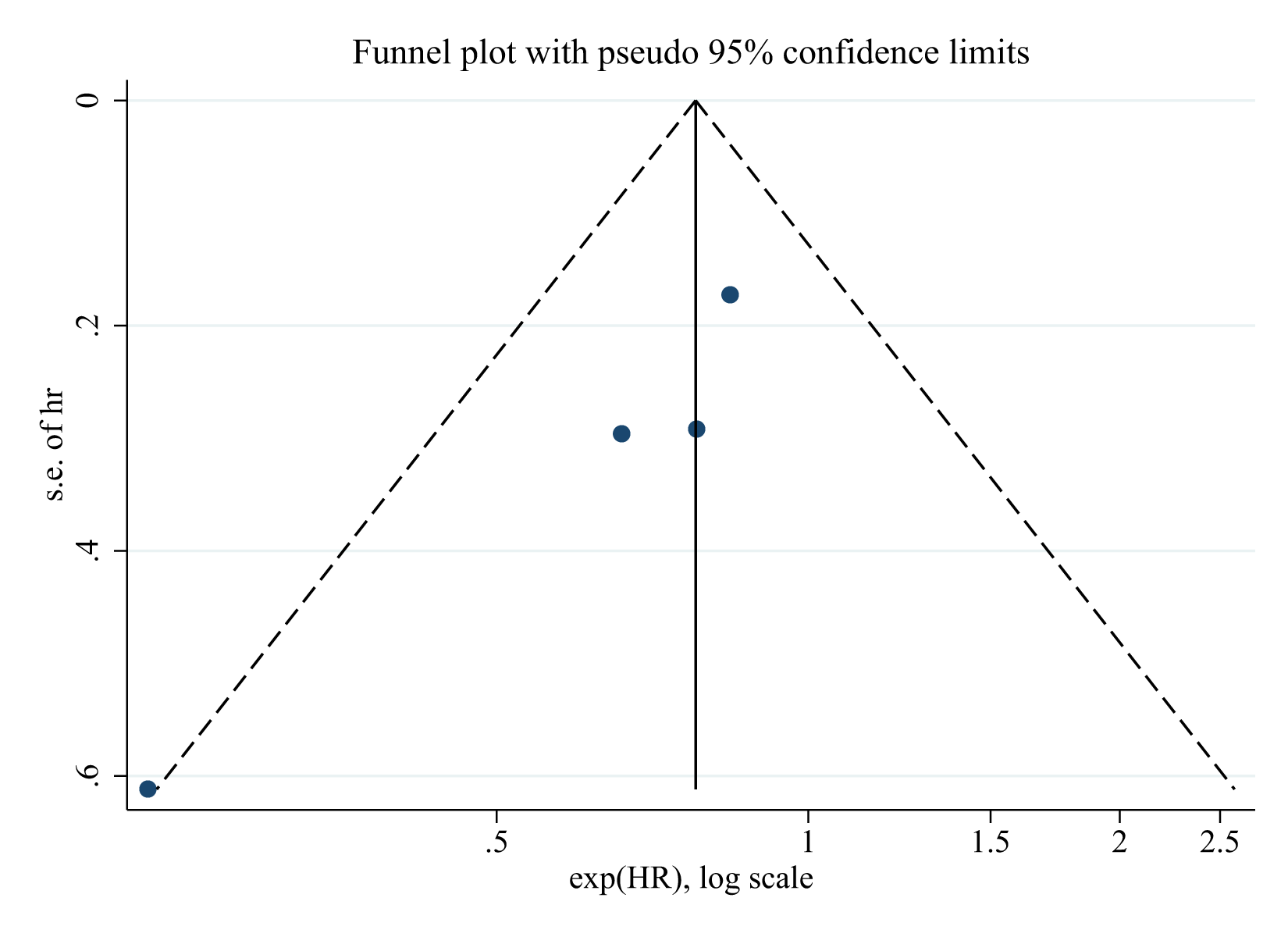


Figure C.69 Funnel plot of overall survival in ER-positive pregnant BC patients compared with non-pregnant BC patients


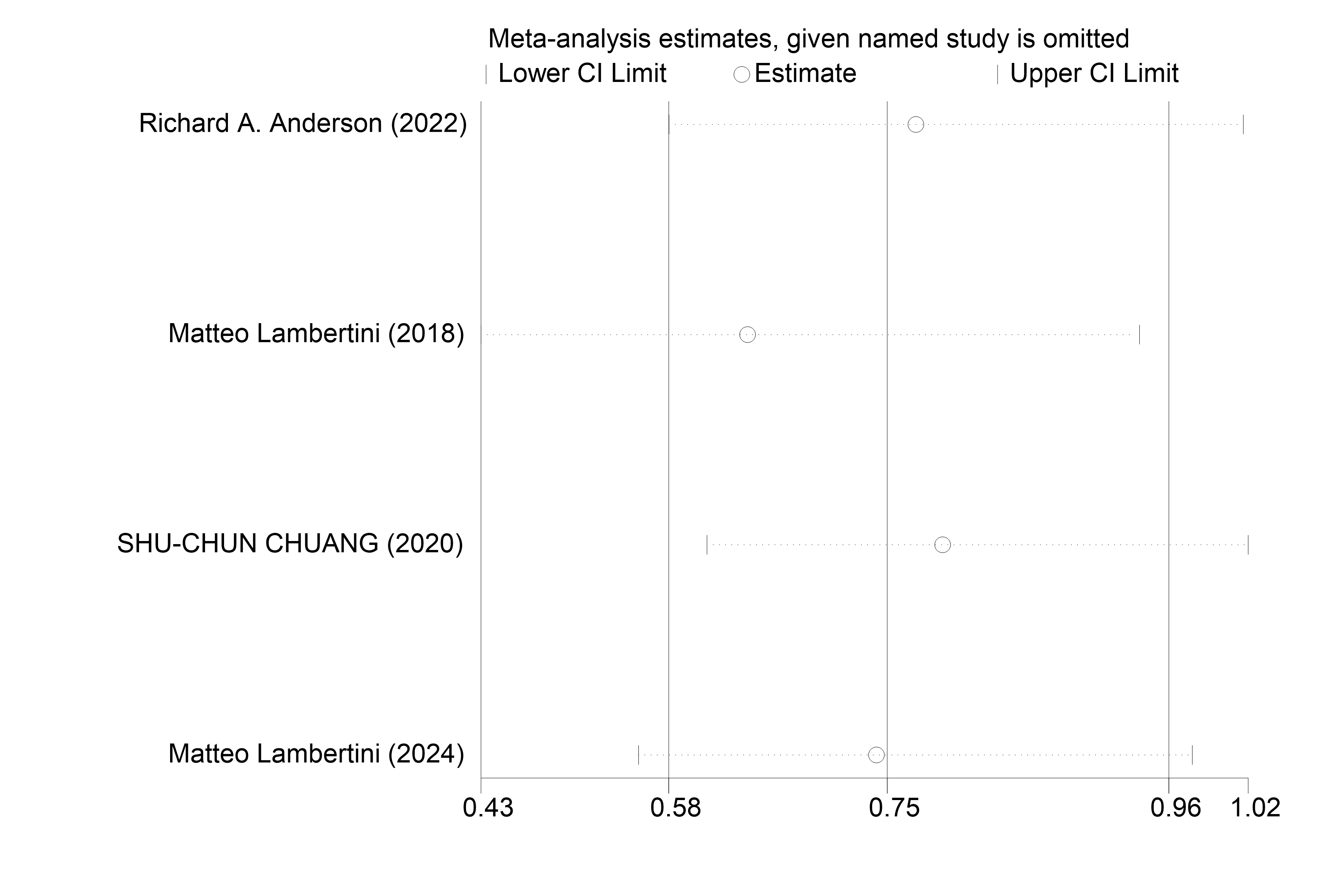


Figure C.70 Sensitivity analysis of overall survival in ER-positive pregnant BC patients compared with non-pregnant BC patients


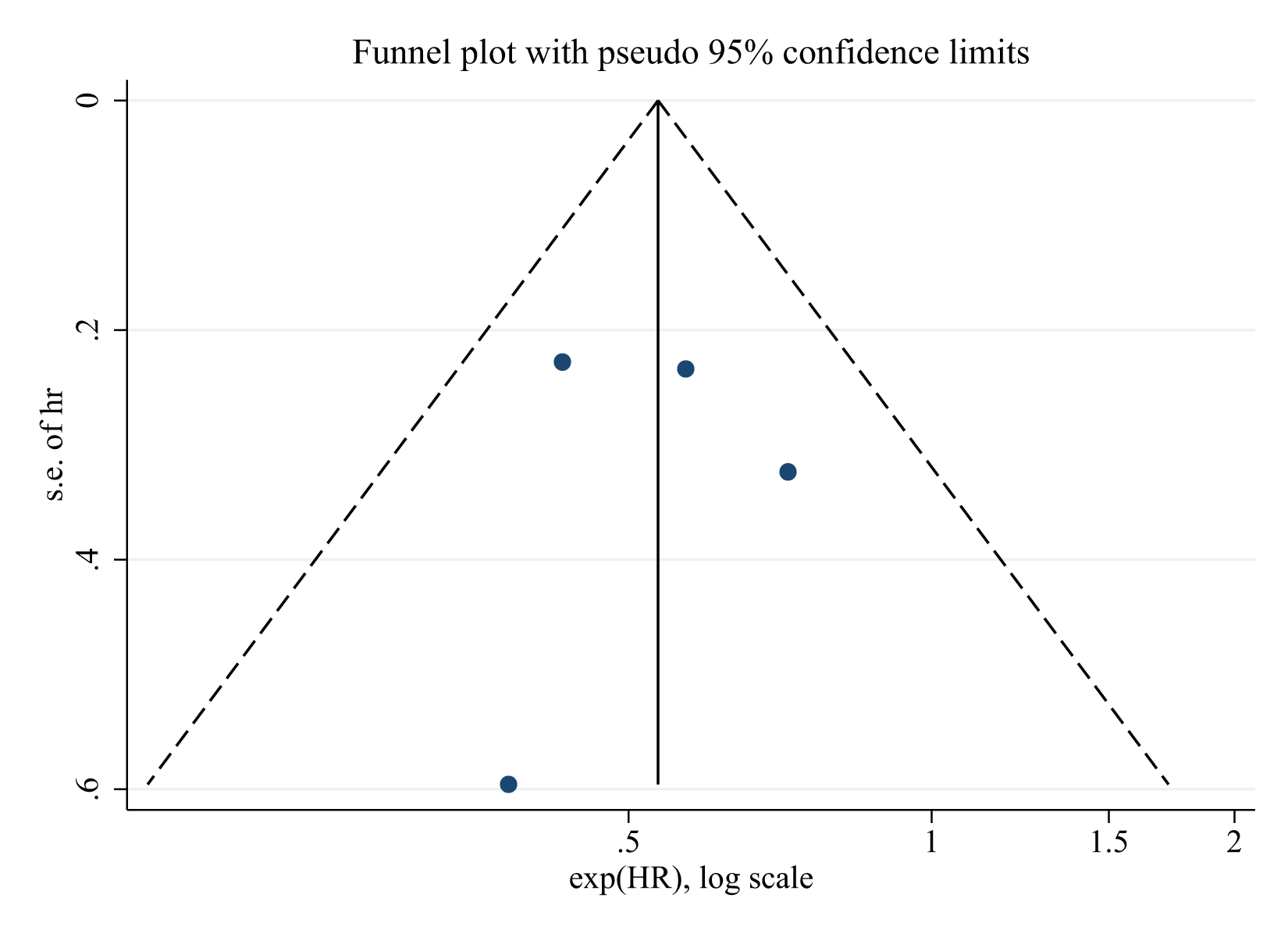


Figure C.71 Funnel plot of overall survival in ER-negative pregnant BC patients compared with non-pregnant BC patients


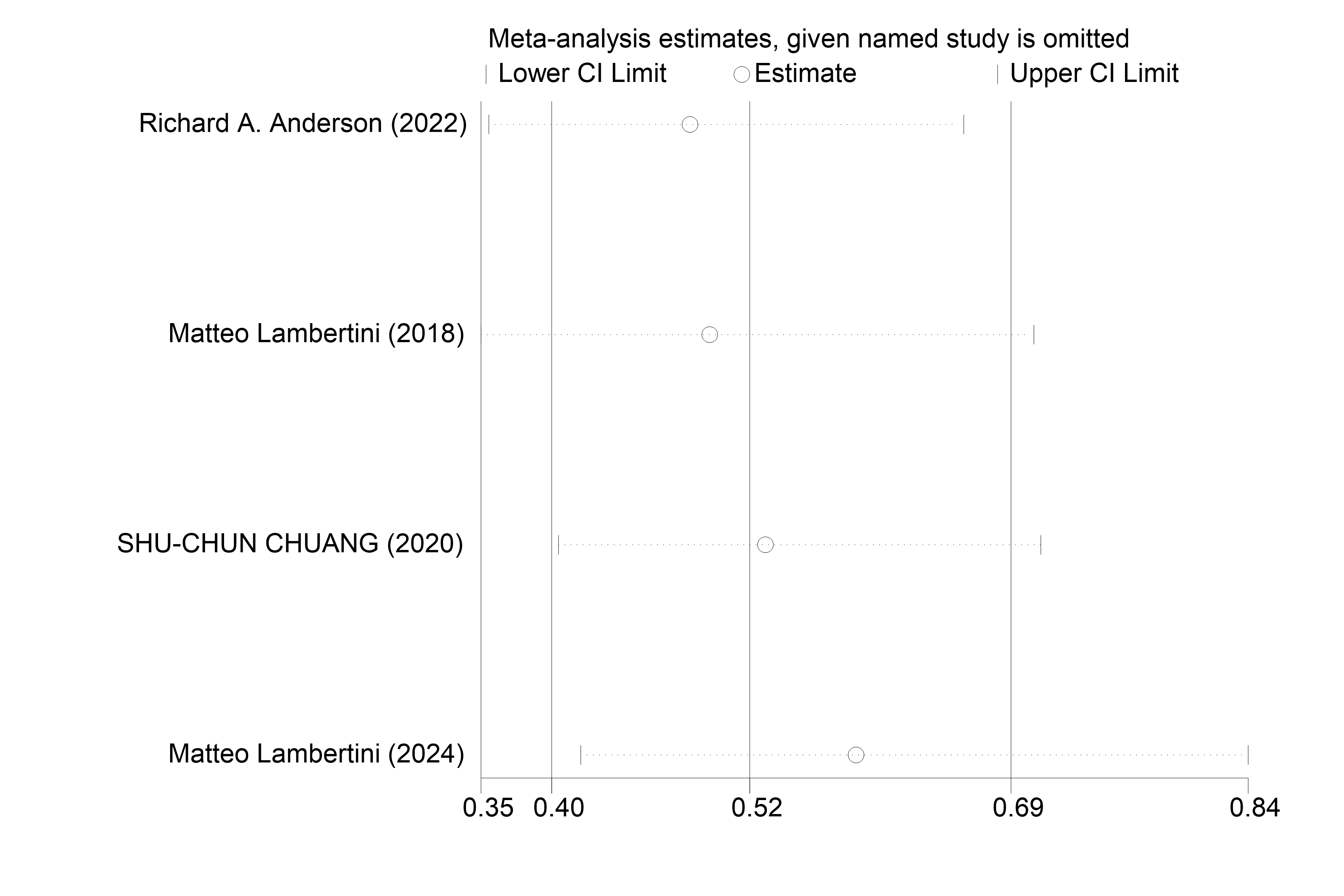


Figure C.72 Sensitivity analysis of overall survival in ER-negative pregnant BC patients compared with non-pregnant BC patients


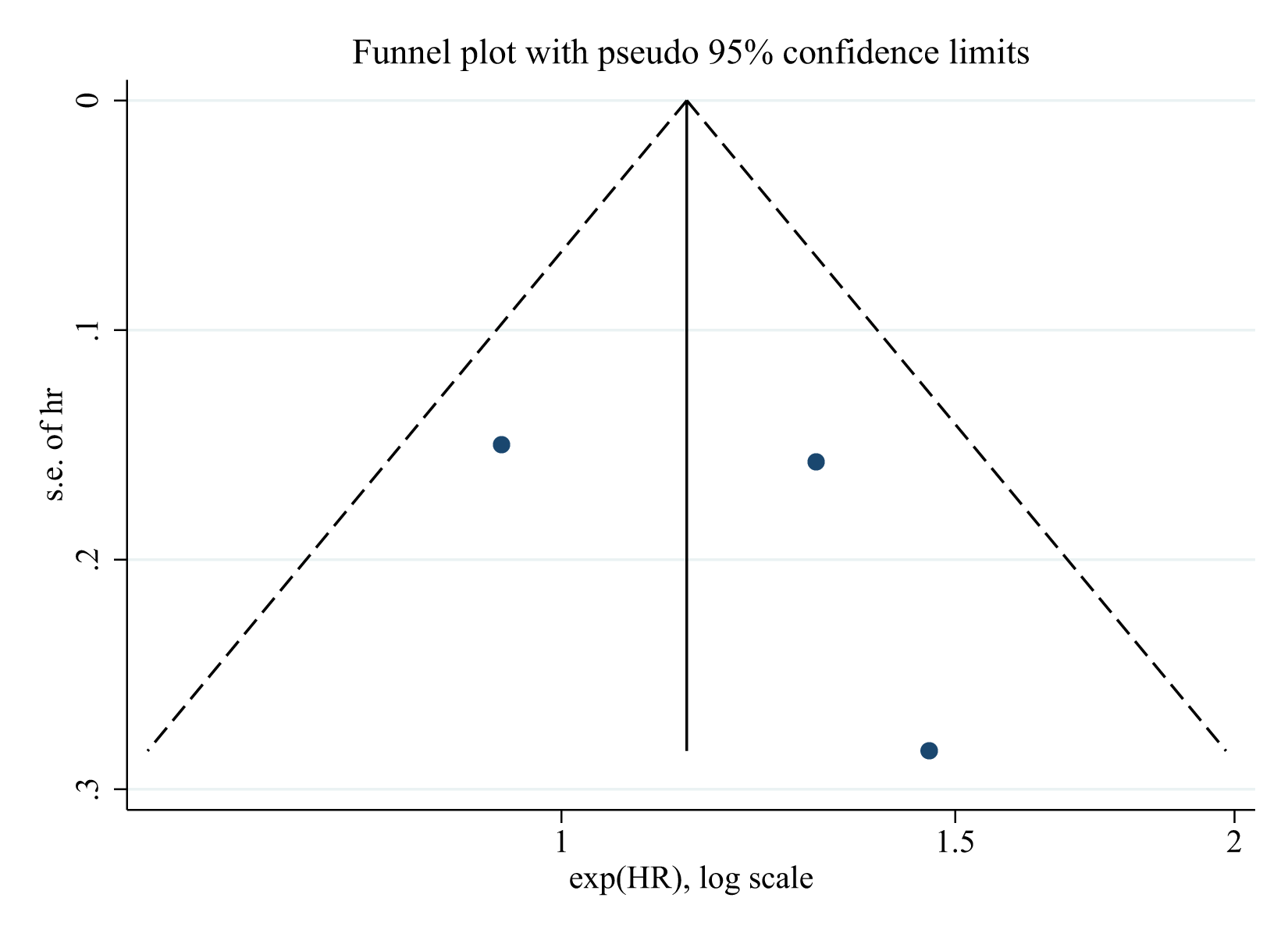


Figure C.73 Funnel plot of disease-free survival in ER-positive pregnant BC patients compared with non-pregnant BC patients


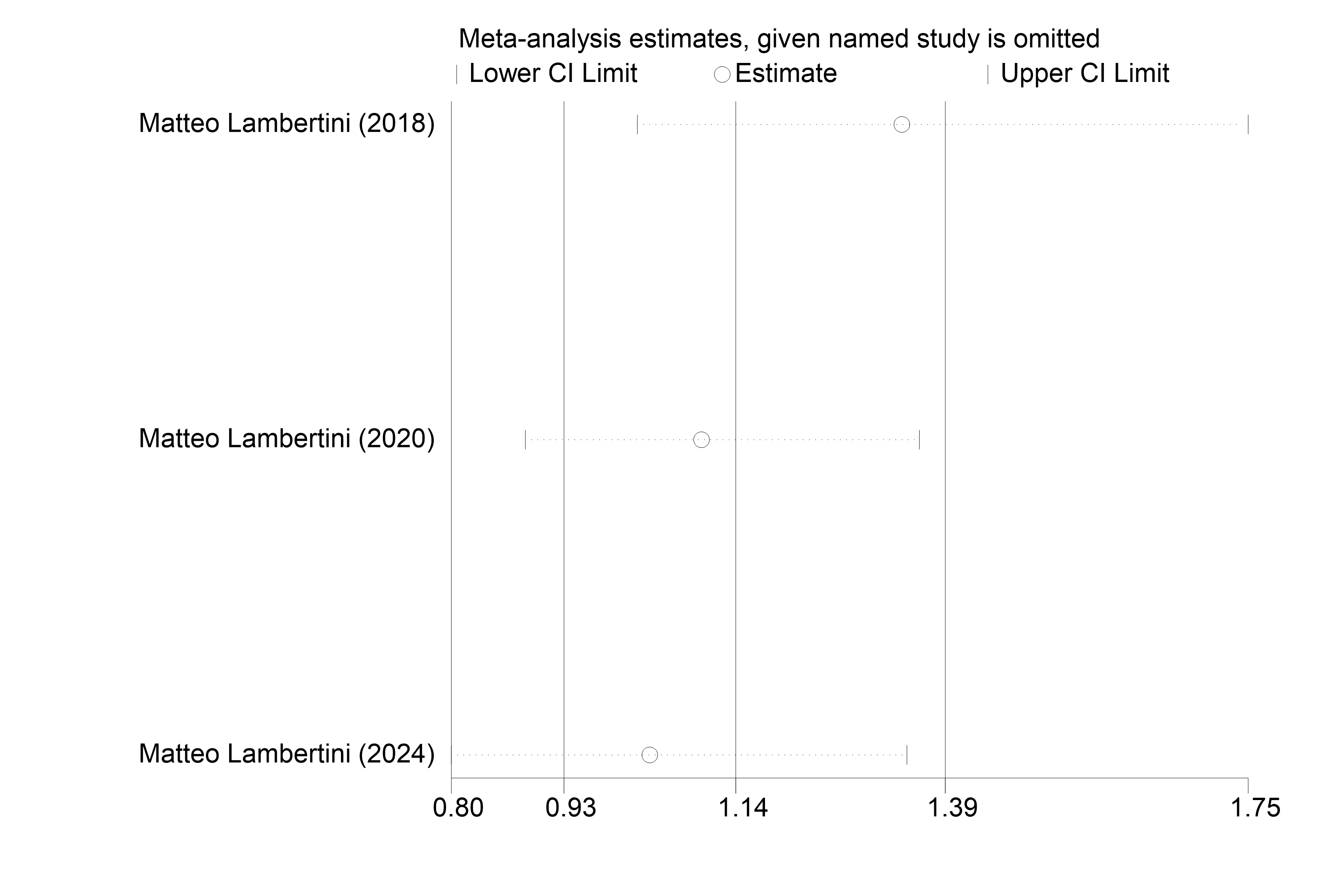


Figure C.74 Sensitivity analysis of disease-free survival in ER-positive pregnant BC patients compared with non-pregnant BC patients


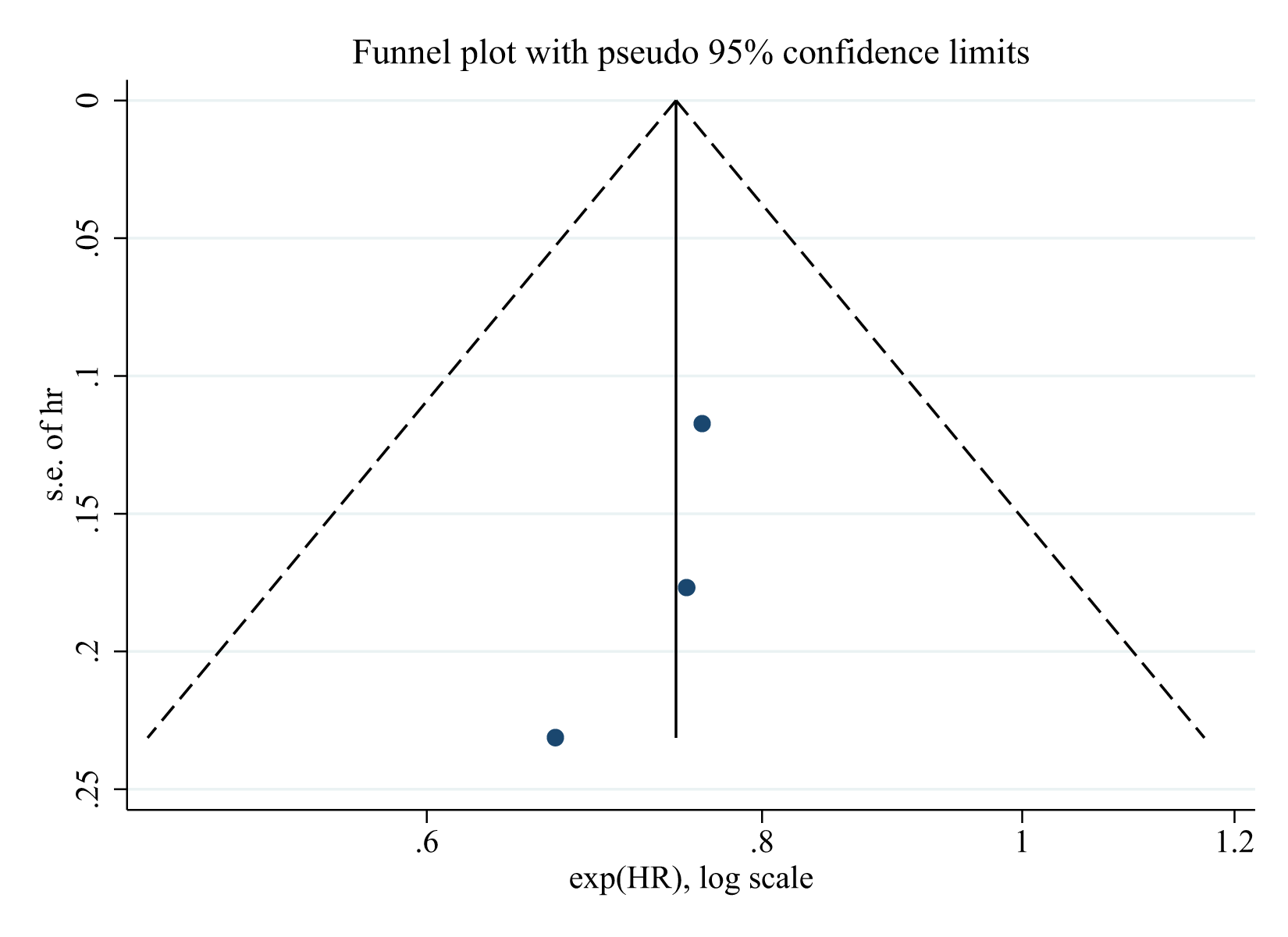


Figure C.75 Funnel plot of disease-free survival in ER-negative pregnant BC patients compared with non-pregnant BC patients


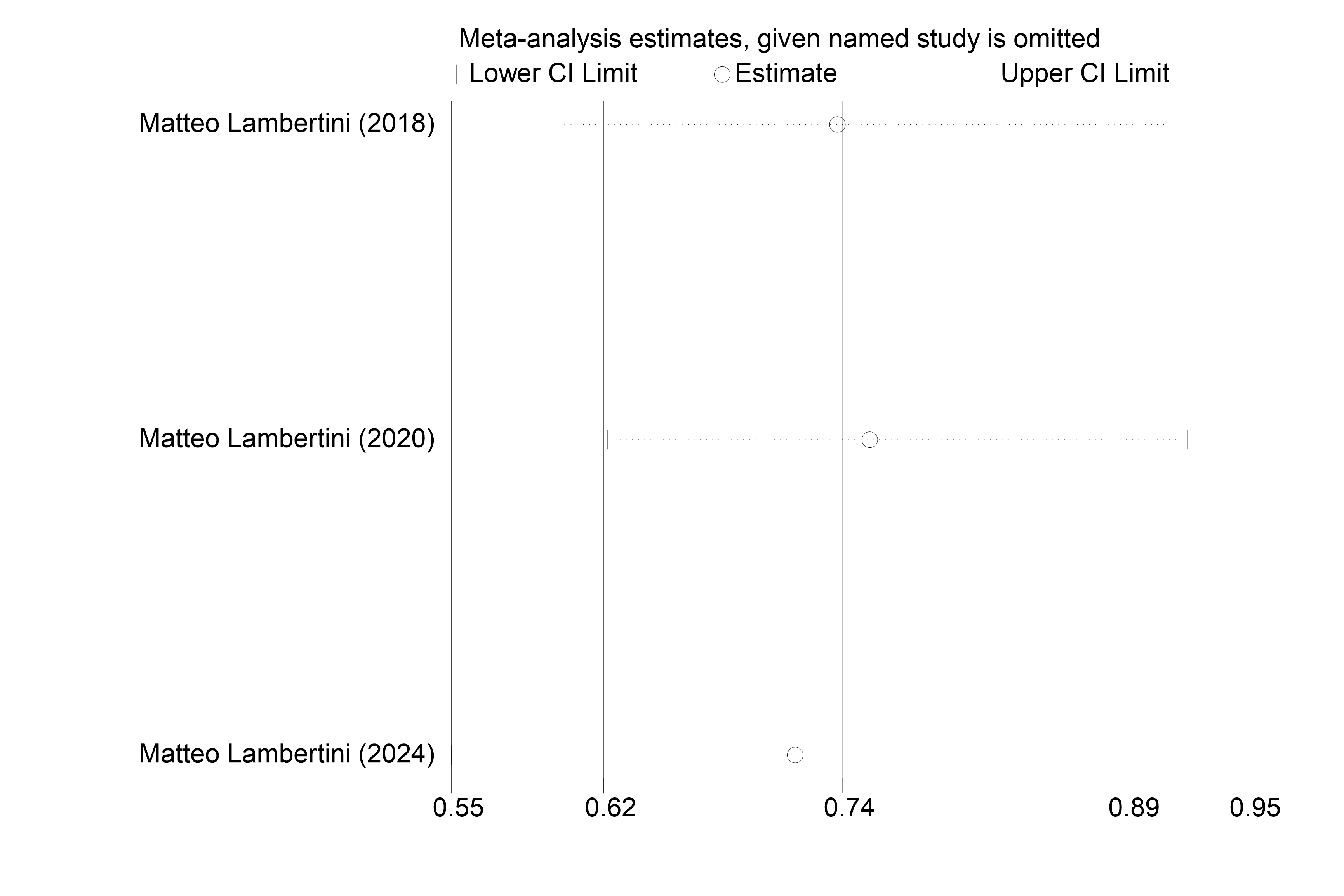


Figure C.76 Sensitivity analysis of disease-free survival in ER-negative pregnant BC patients compared with non-pregnant BC patients


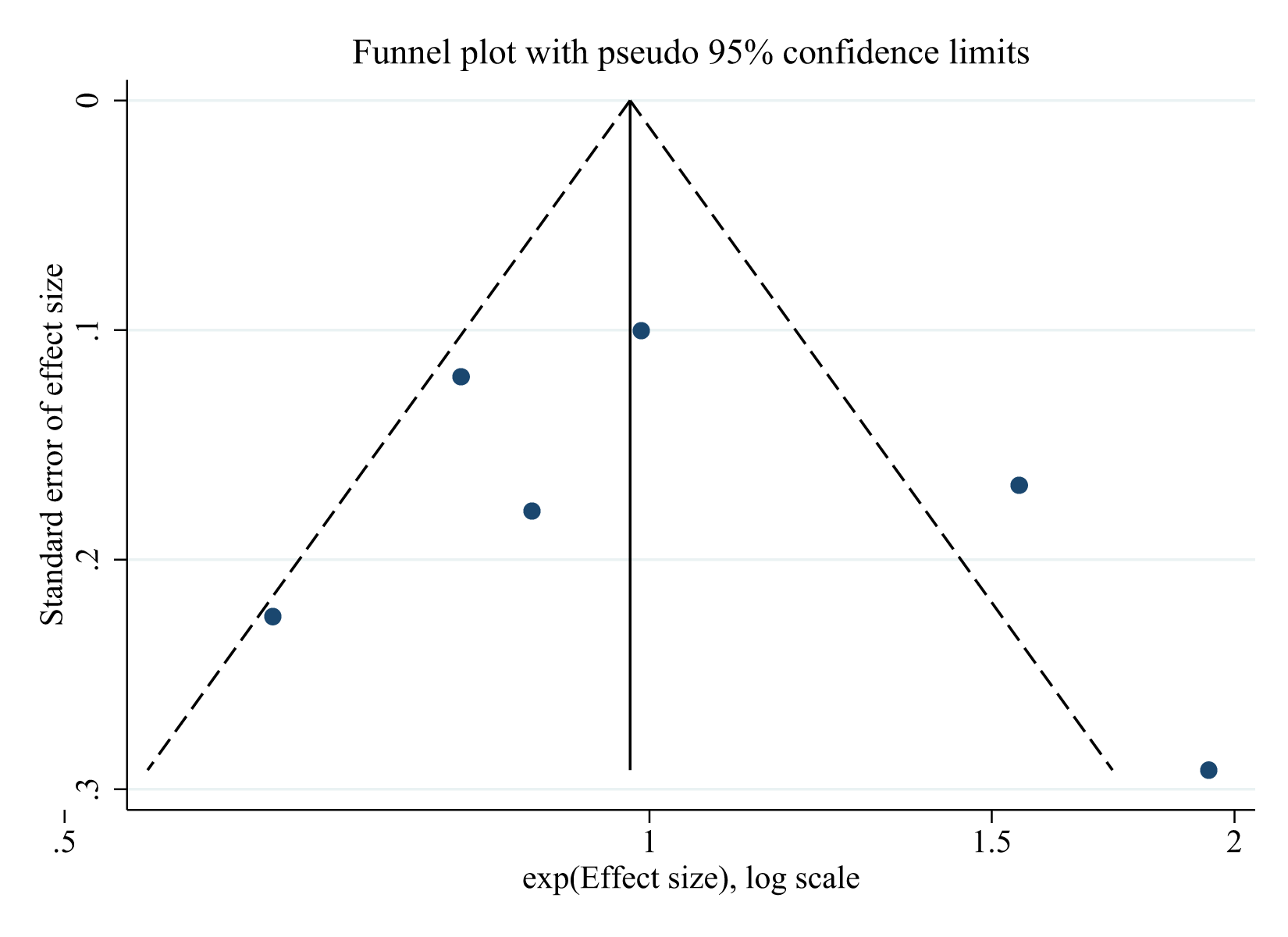


Figure C.77 Funnel plot of disease-free survival in BRCA mutation pregnant BC patients compared with non-pregnant BC patients


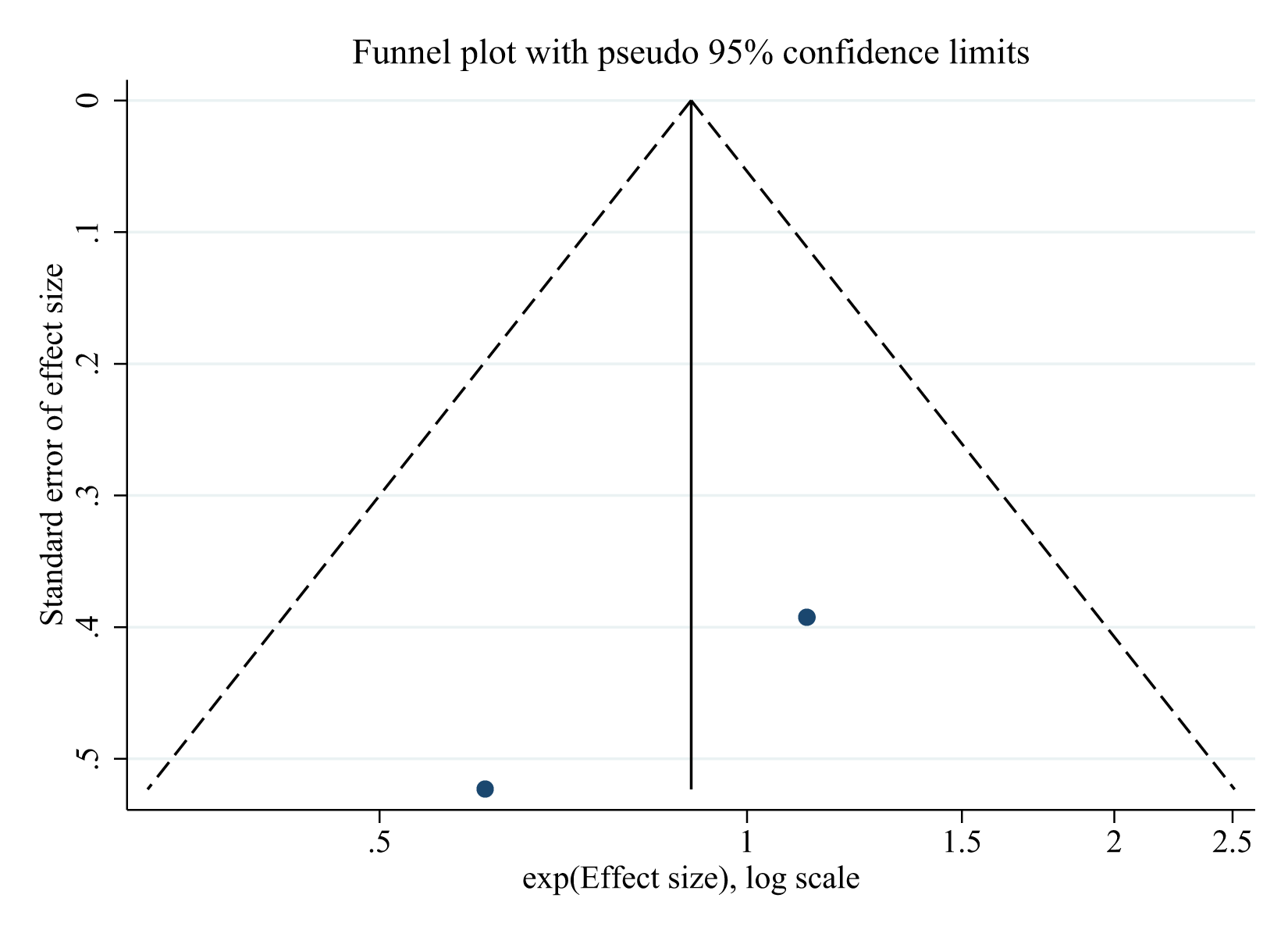


Figure C.78 Funnel plot of disease-free survival in Her-2 positive pregnant BC patients compared with non-pregnant BC patients


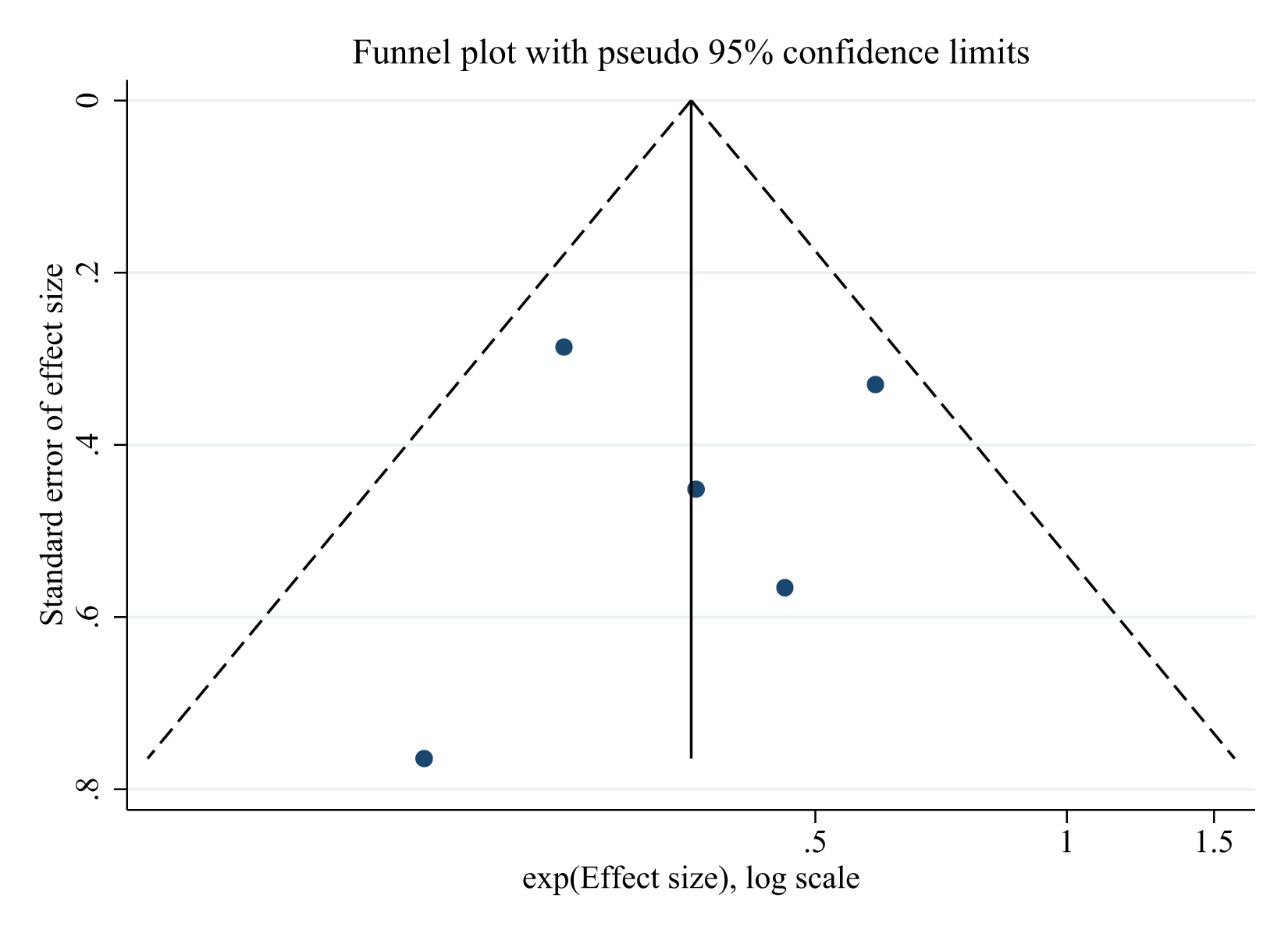


Figure C.79 Funnel plot of overall survival in pregnant BC patients who had received hormone therapy compared with non-pregnant BC patients


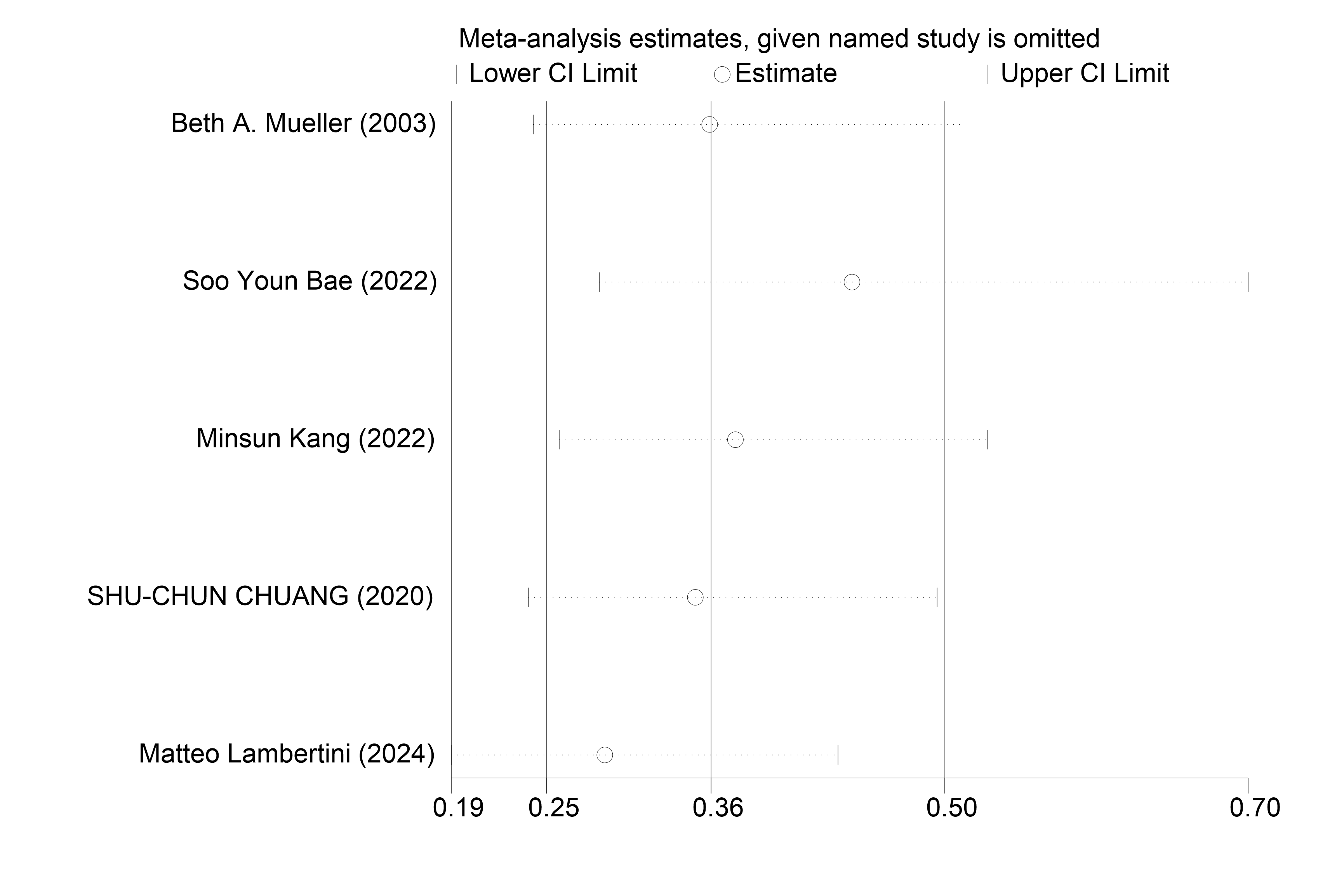


Figure C.80 Sensitivity analysis of overall survival in pregnant BC patients who had received hormone therapy compared with non-pregnant BC patients


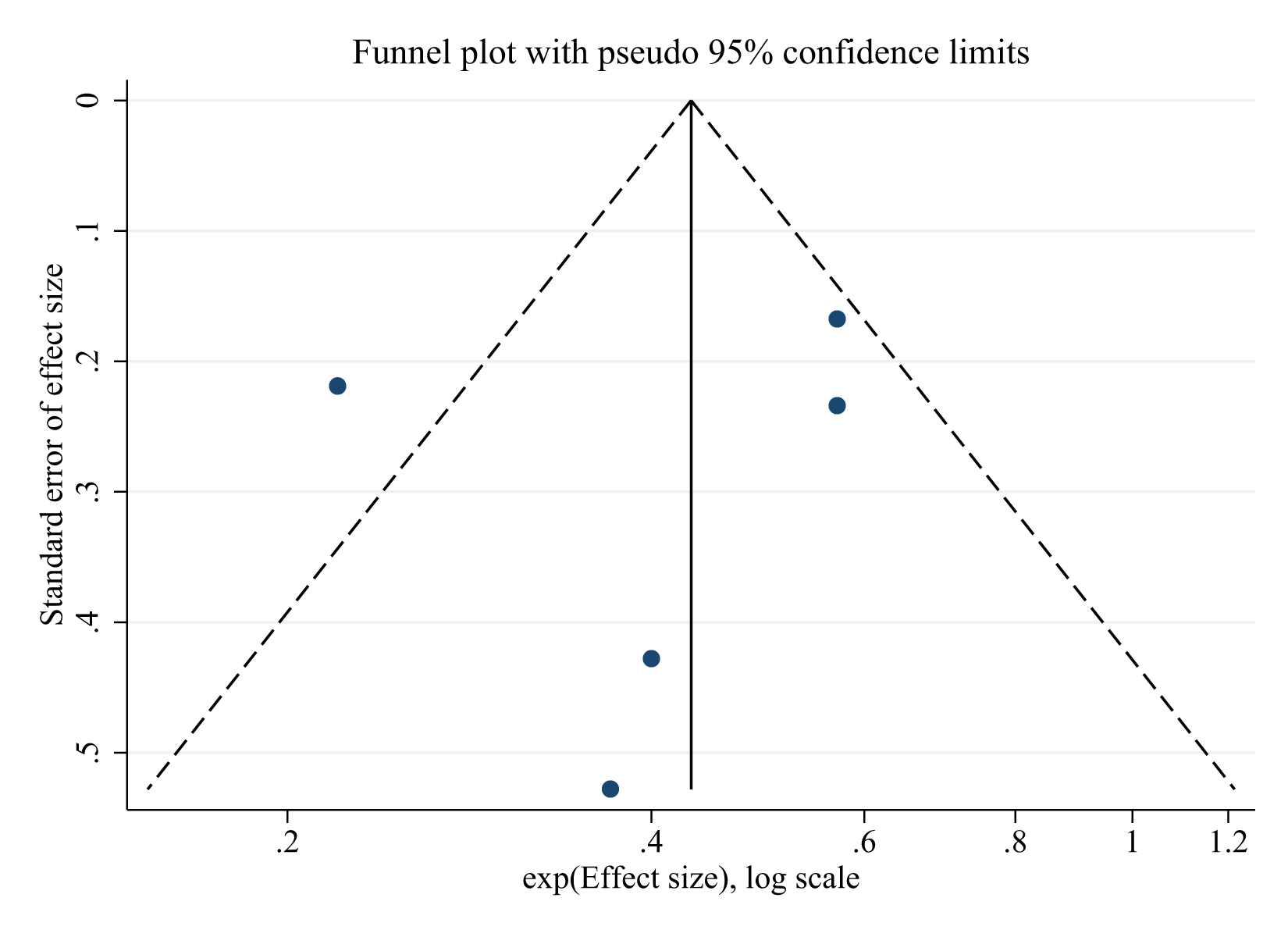


Figure C.81 Funnel plot of overall survival in pregnant BC patients who had not received hormone therapy compared with non-pregnant BC patients


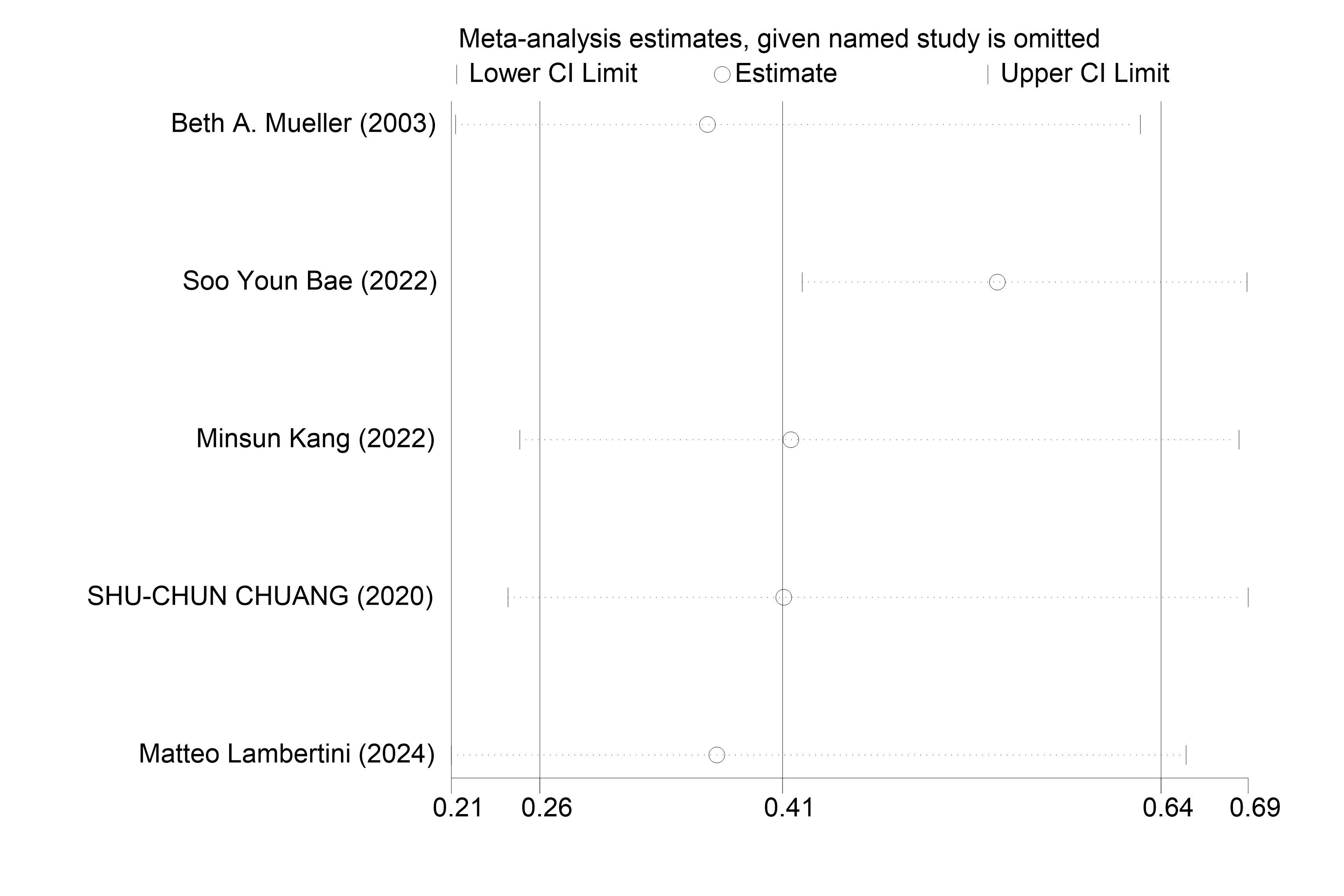


Figure C.82 Sensitivity analysis of overall survival in pregnant BC patients who had not received hormone therapy compared with non-pregnant BC patients


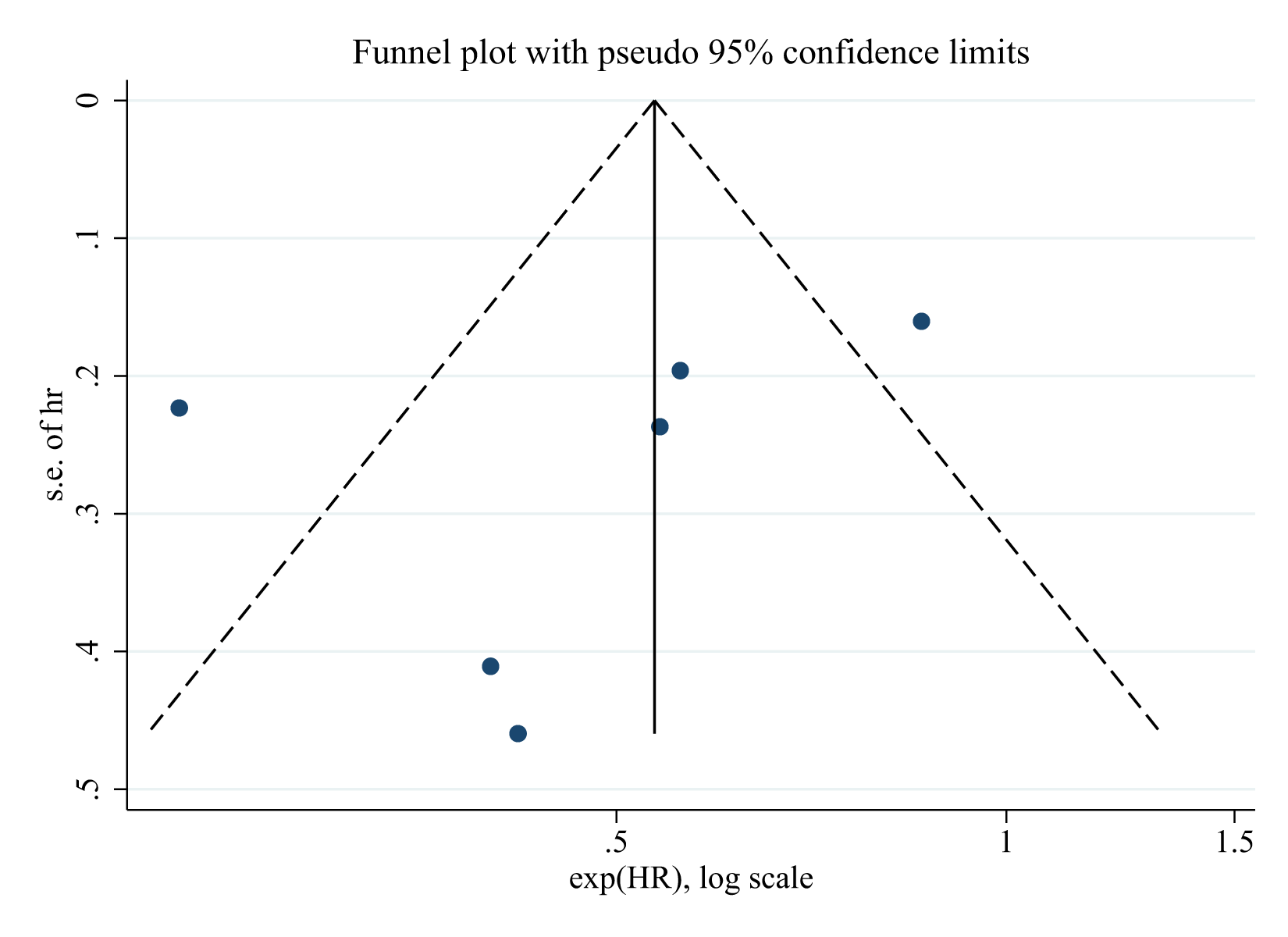


Figure C.83 Funnel plot of overall survival in pregnant BC patients who had received chemotherapy compared with non-pregnant BC patients


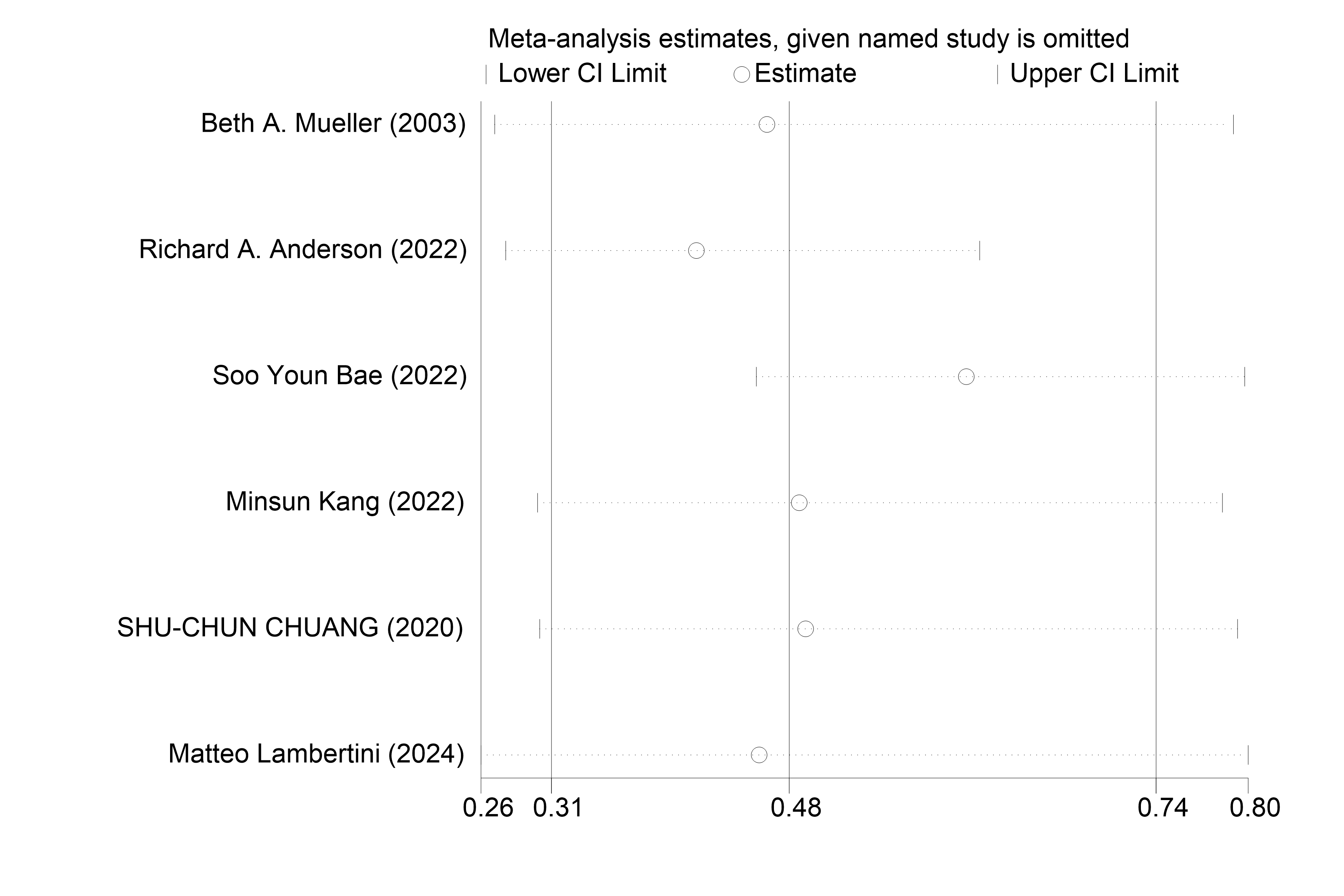


Figure C.84 Sensitivity analysis of overall survival in pregnant BC patients who had received chemotherapy compared with non-pregnant BC patients


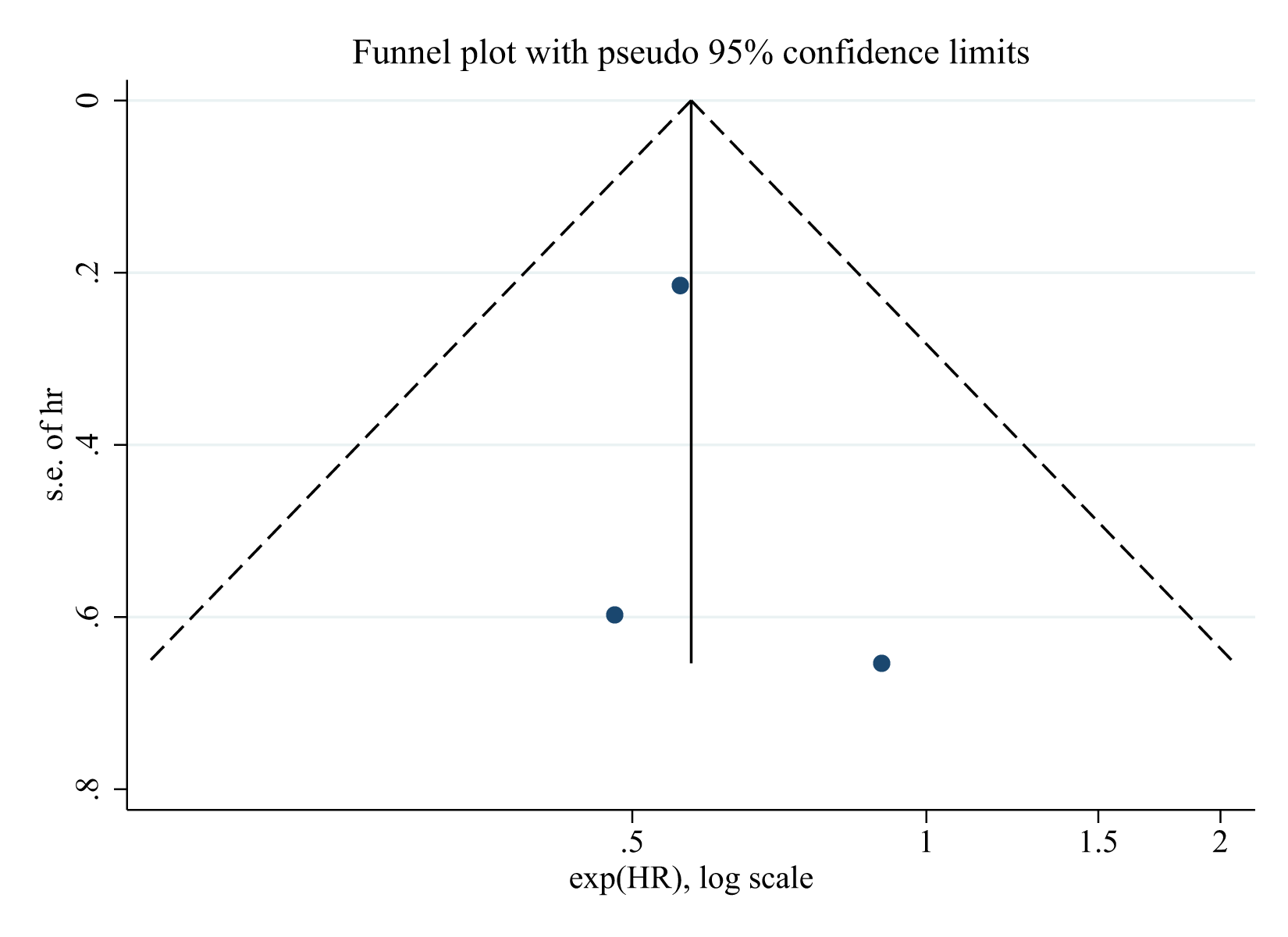


Figure C.85 Funnel plot of overall survival in pregnant BC patients who had not received chemotherapy compared with non-pregnant BC patients


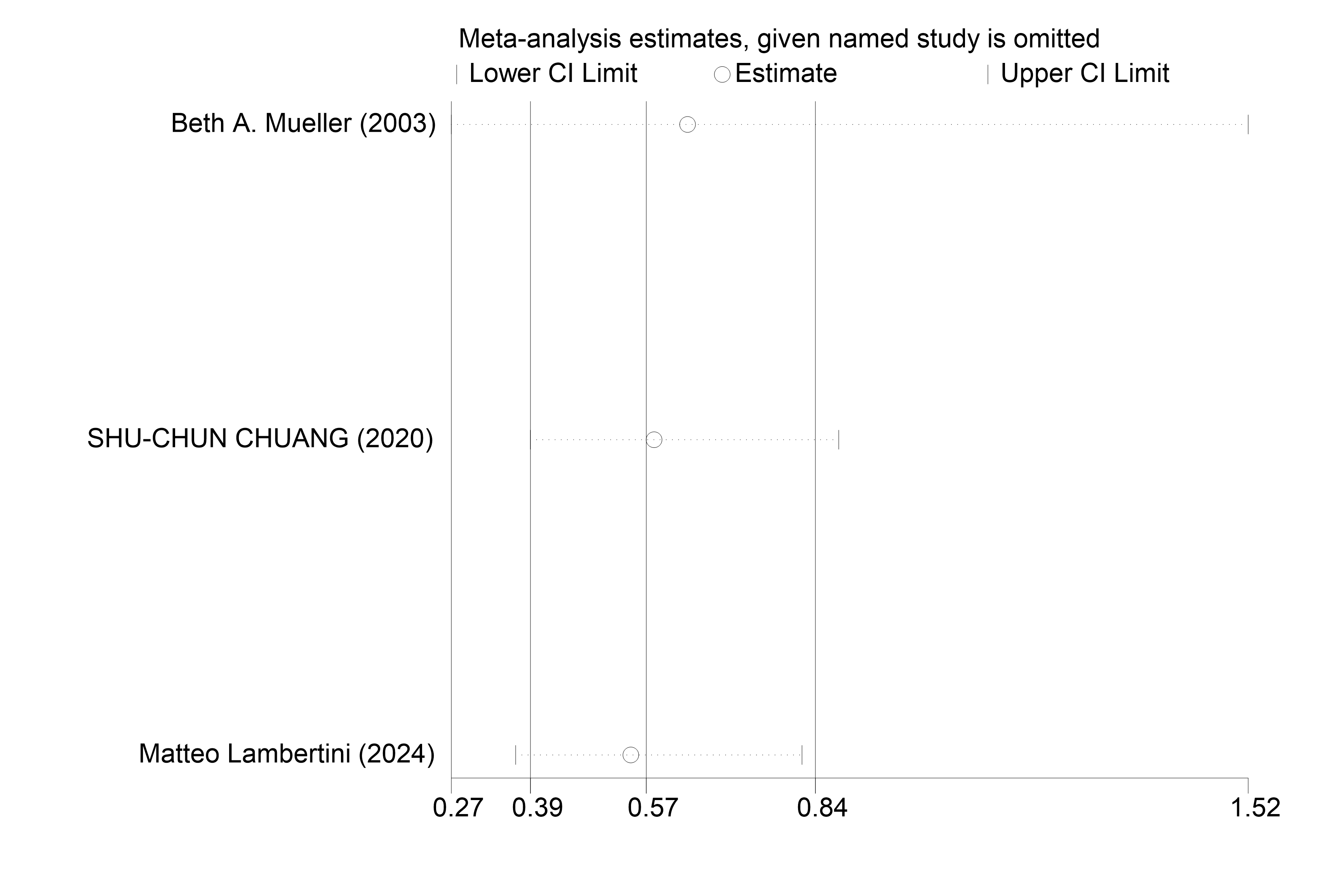


Figure C.86 Sensitivity analysis of overall survival in pregnant BC patients who had not received chemotherapy compared with non-pregnant BC patients


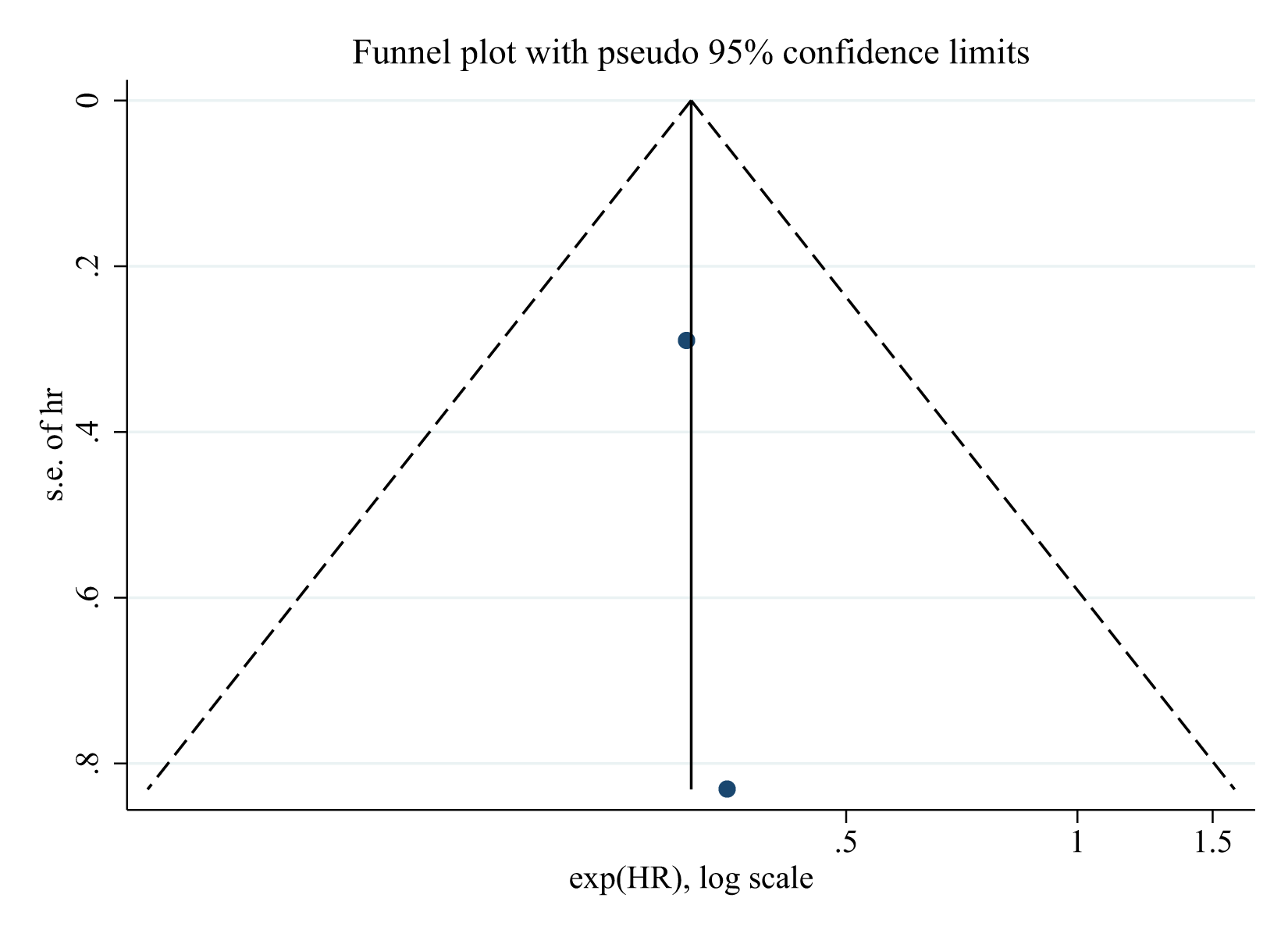


Figure C.87 Funnel plot of overall survival in pregnant BC patients who had received endocrine therapy and chemotherapy compared with non-pregnant BC patients


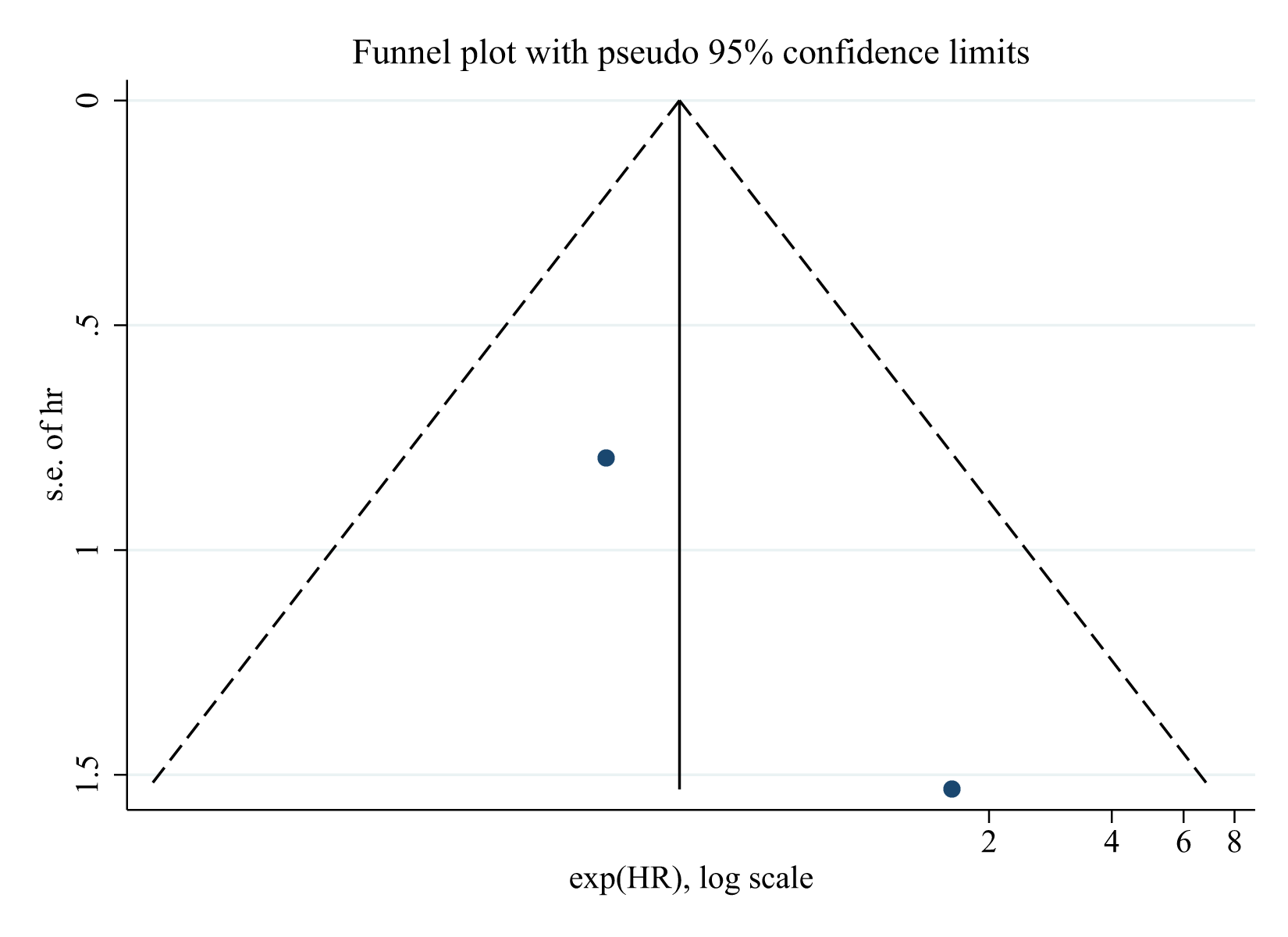


Figure C.88 Funnel plot of overall survival in pregnant BC patients who had received chemotherapy and trastuzumab compared with non-pregnant BC patients


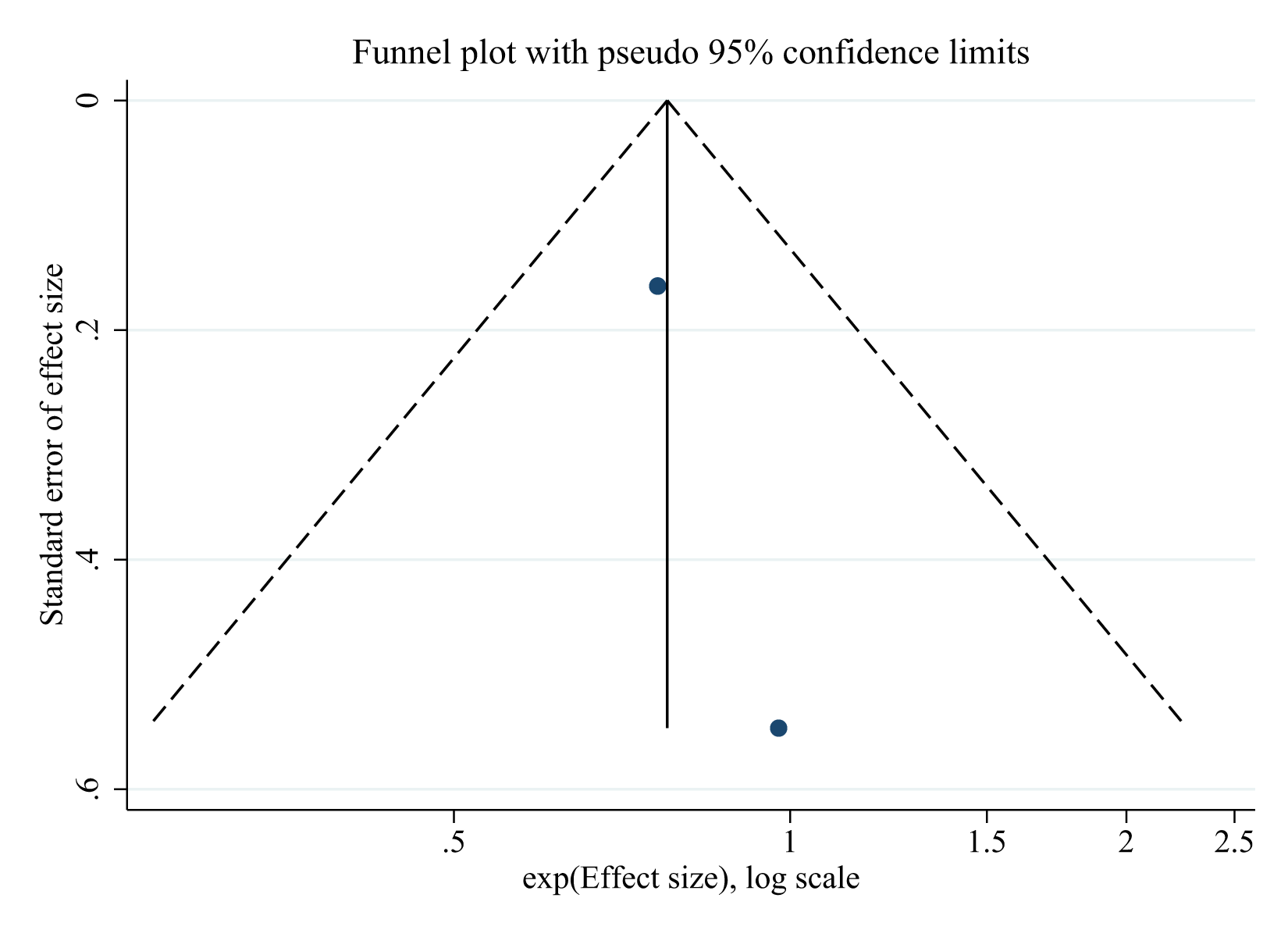


Figure C.89 Funnel plot of recurrence rate in ER-positive pregnant BC patients who had had interruption of adjuvant endocrine therapy compared with non-pregnant BC patients


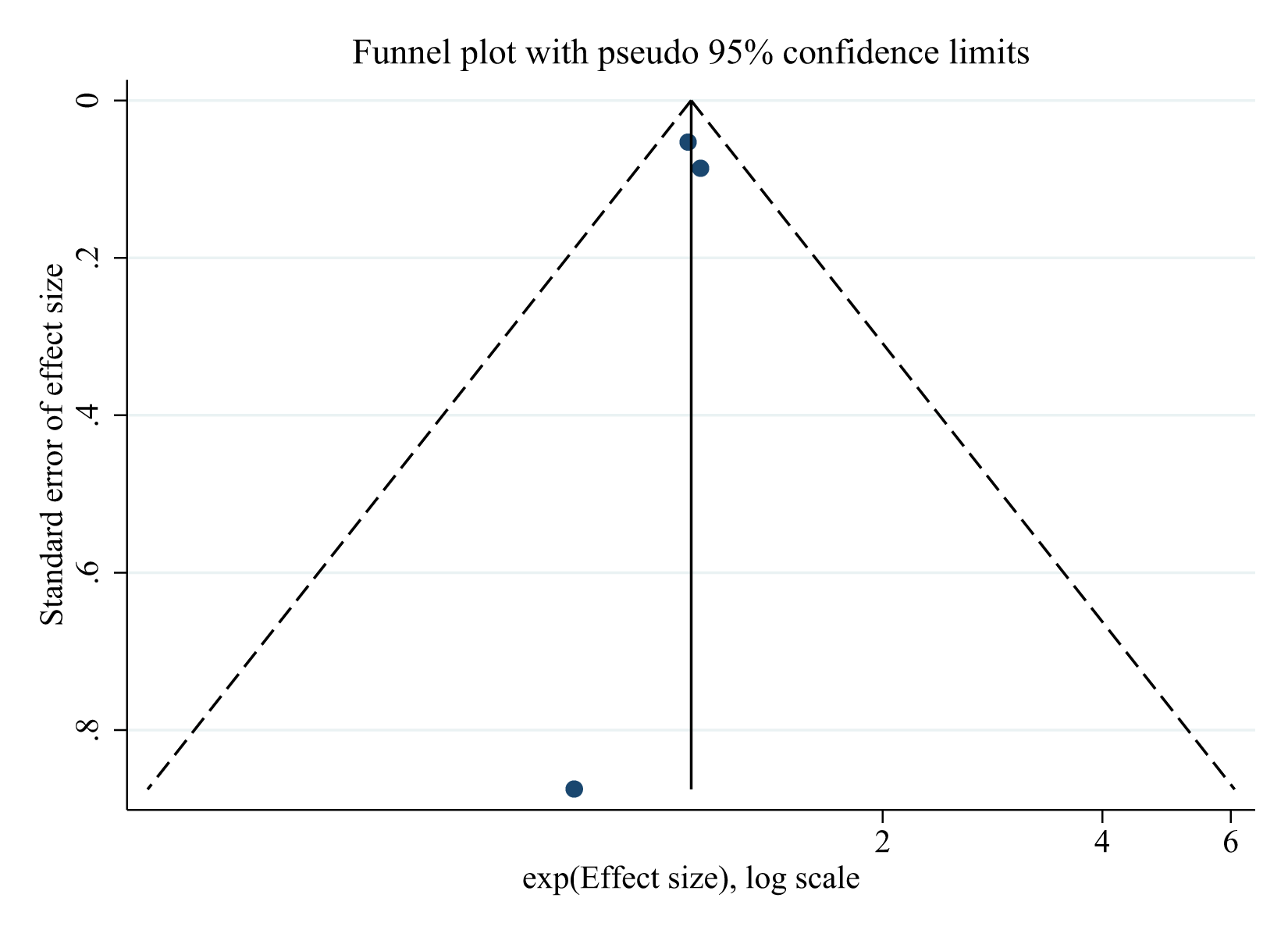


Figure C.90 Funnel plot of overall survival in lymph node positive pregnant BC patients compared with non-pregnant BC patients


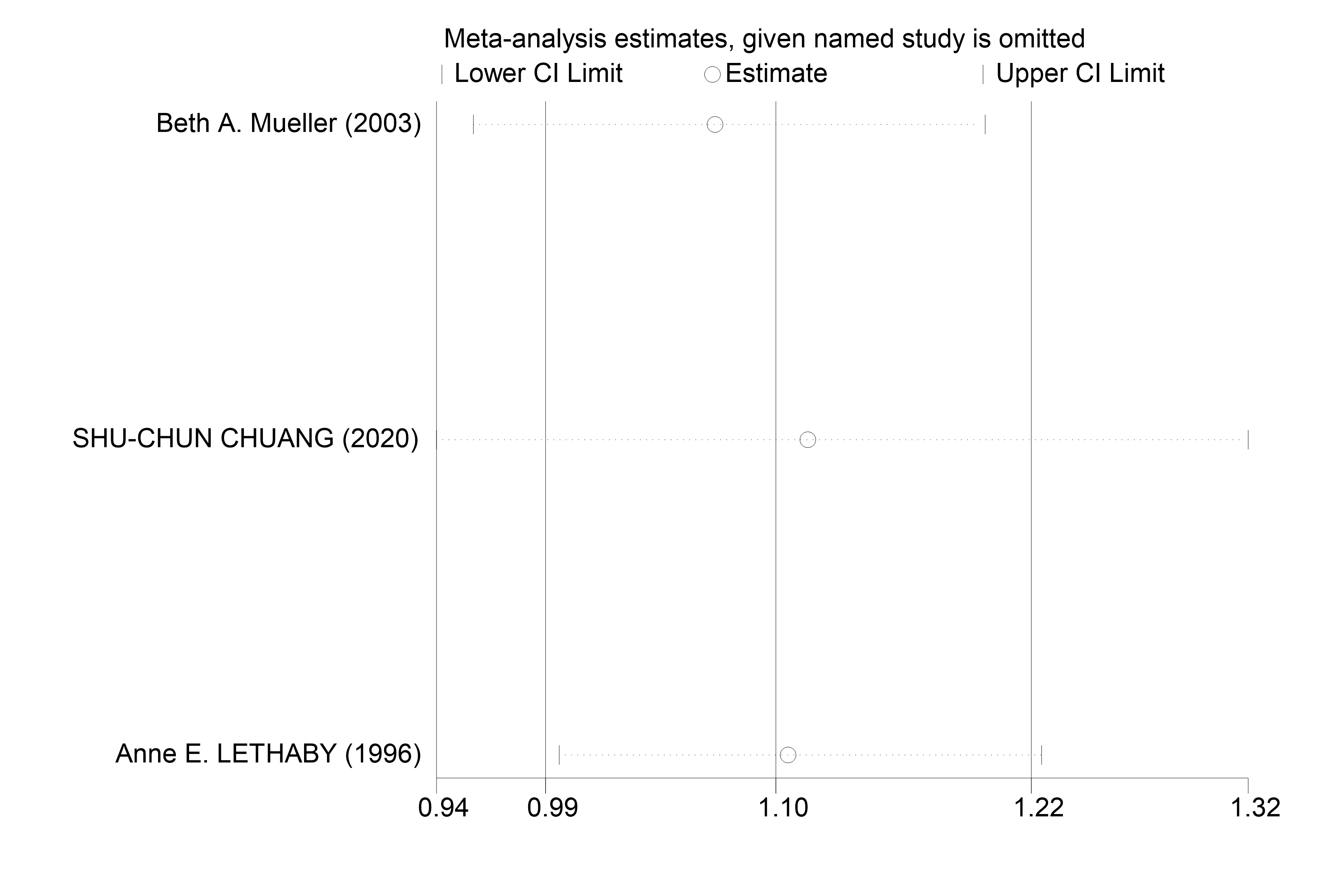


Figure C.91 Sensitivity analysis of overall survival in lymph node positive pregnant BC patients compared with non-pregnant BC patients


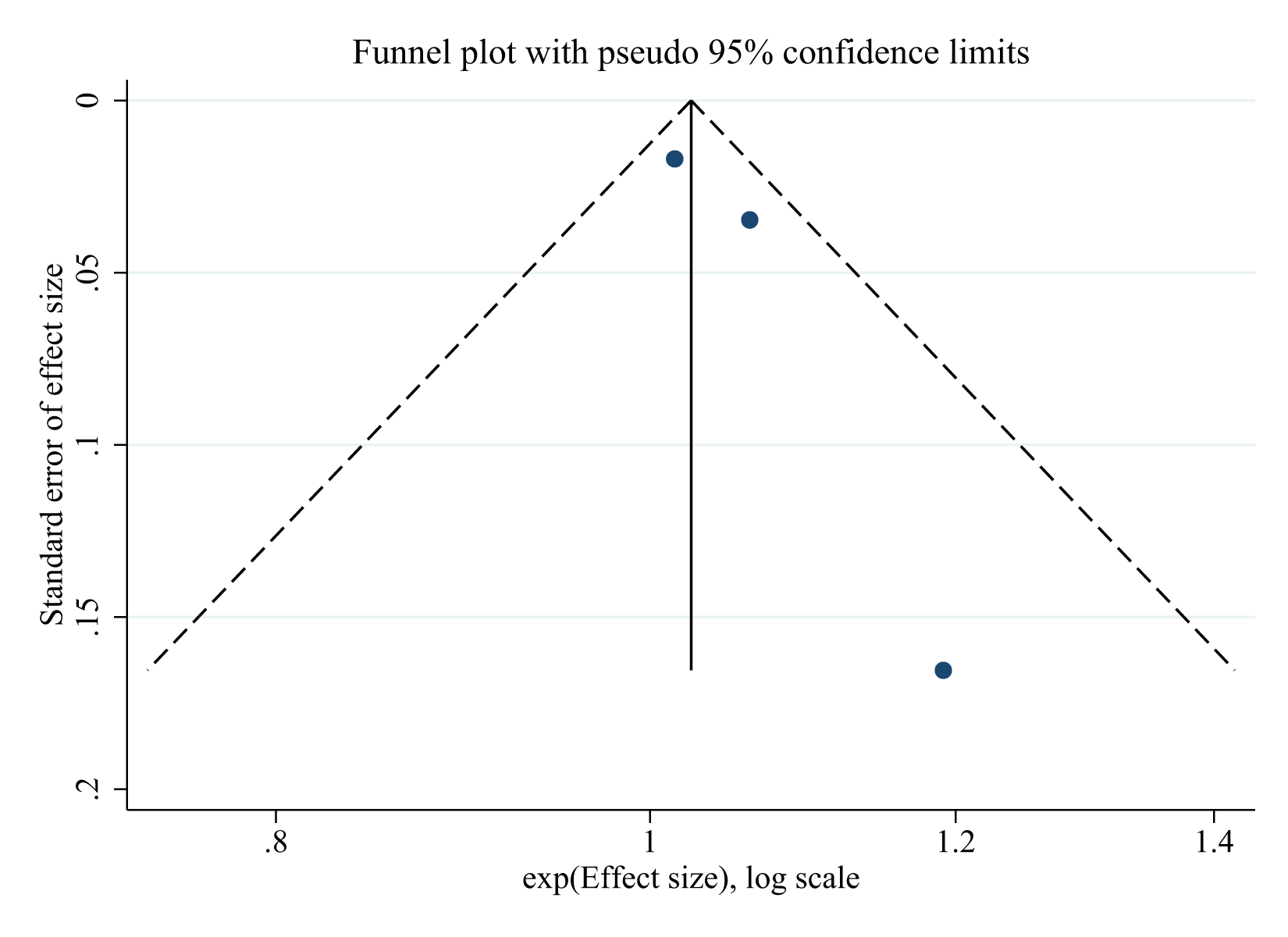


Figure C.92 Funnel plot of overall survival in lymph node negative pregnant BC patients compared with non-pregnant BC patients


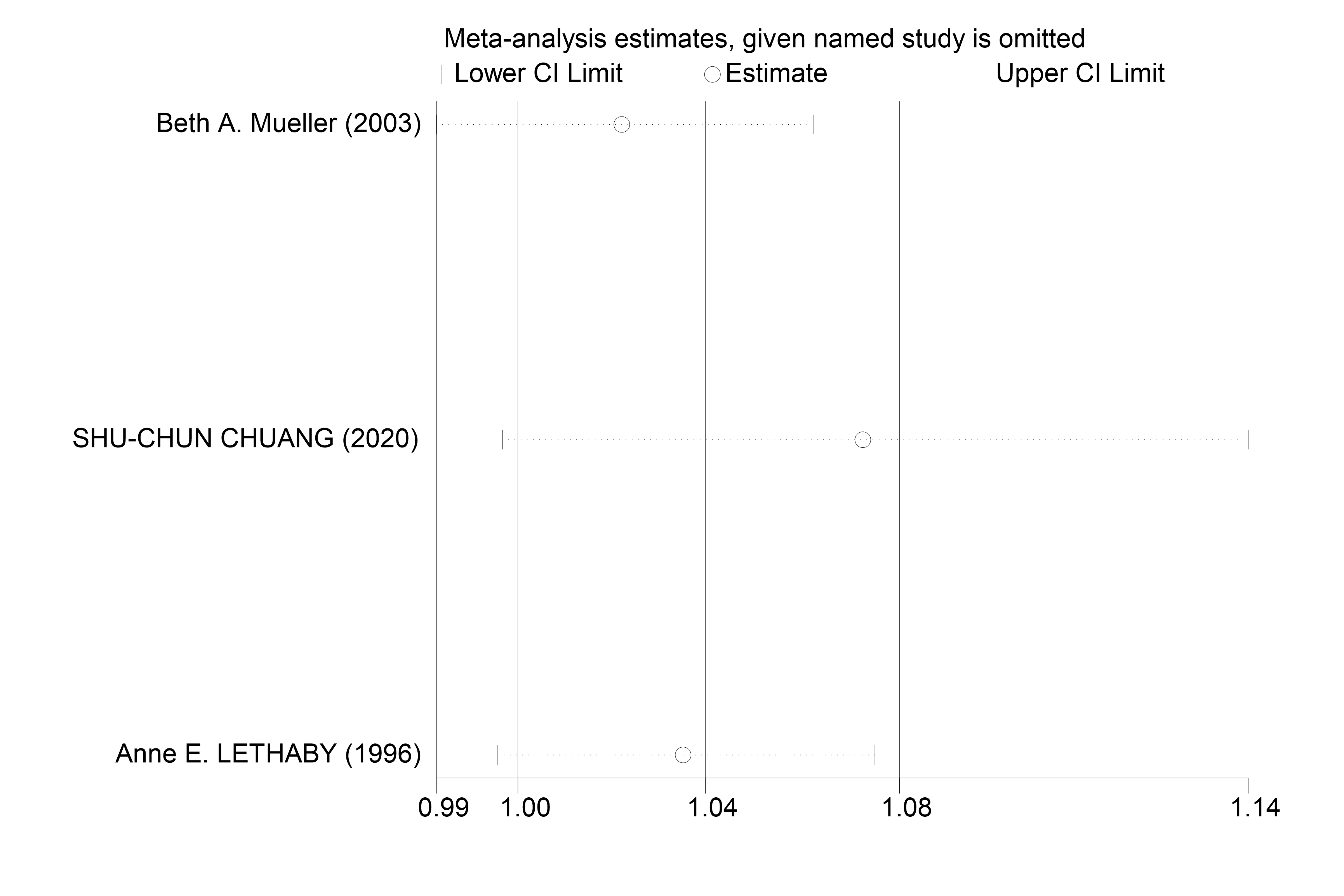


Figure C.93 Sensitivity analysis of overall survival in lymph node negative pregnant BC patients compared with non-pregnant BC patients


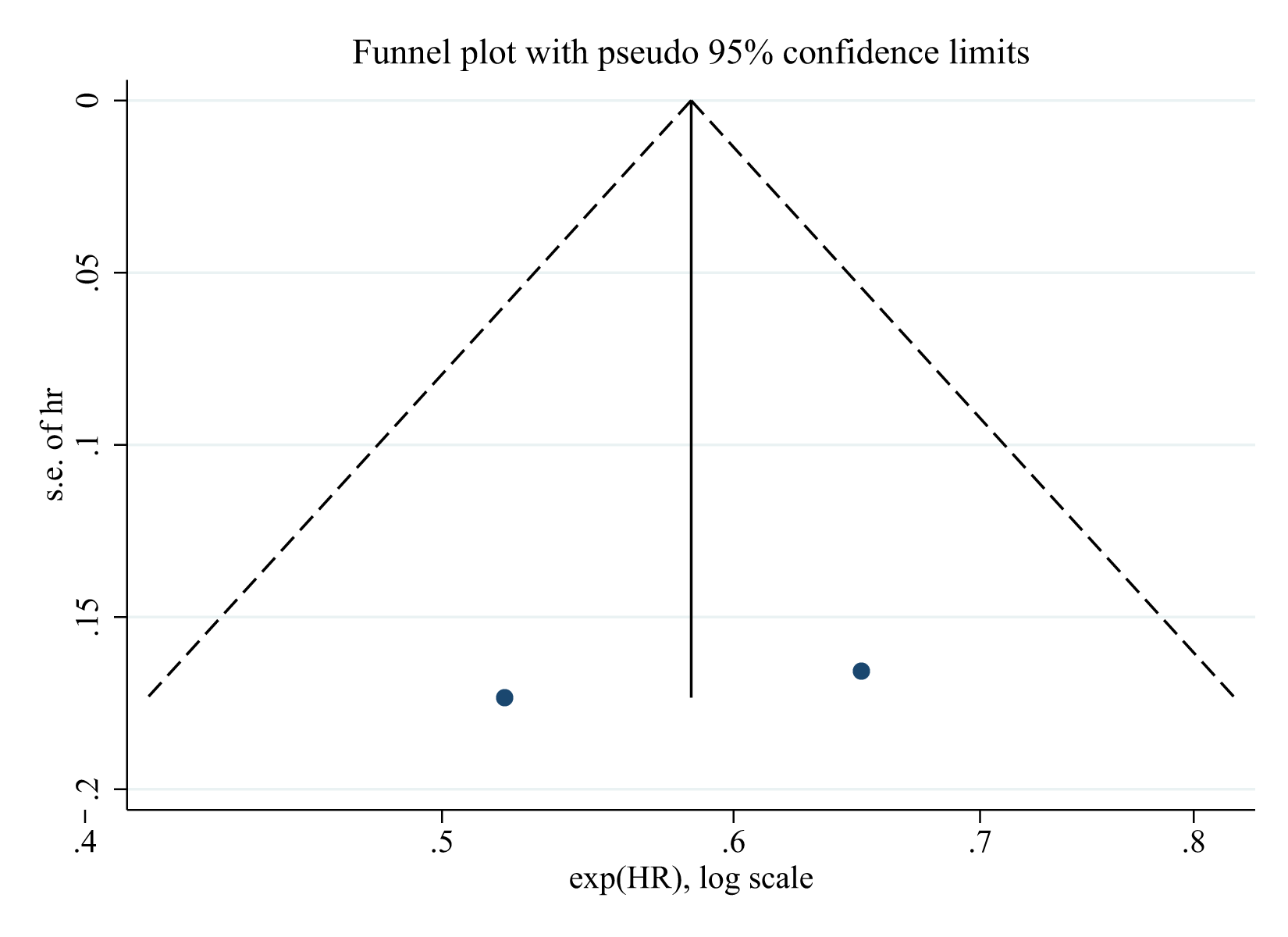


Figure C.94 Funnel plot of disease-free survival in pregnant BC patients who had an interval between diagnosis and pregnancy ≤2 years compared with non-pregnant BC patients


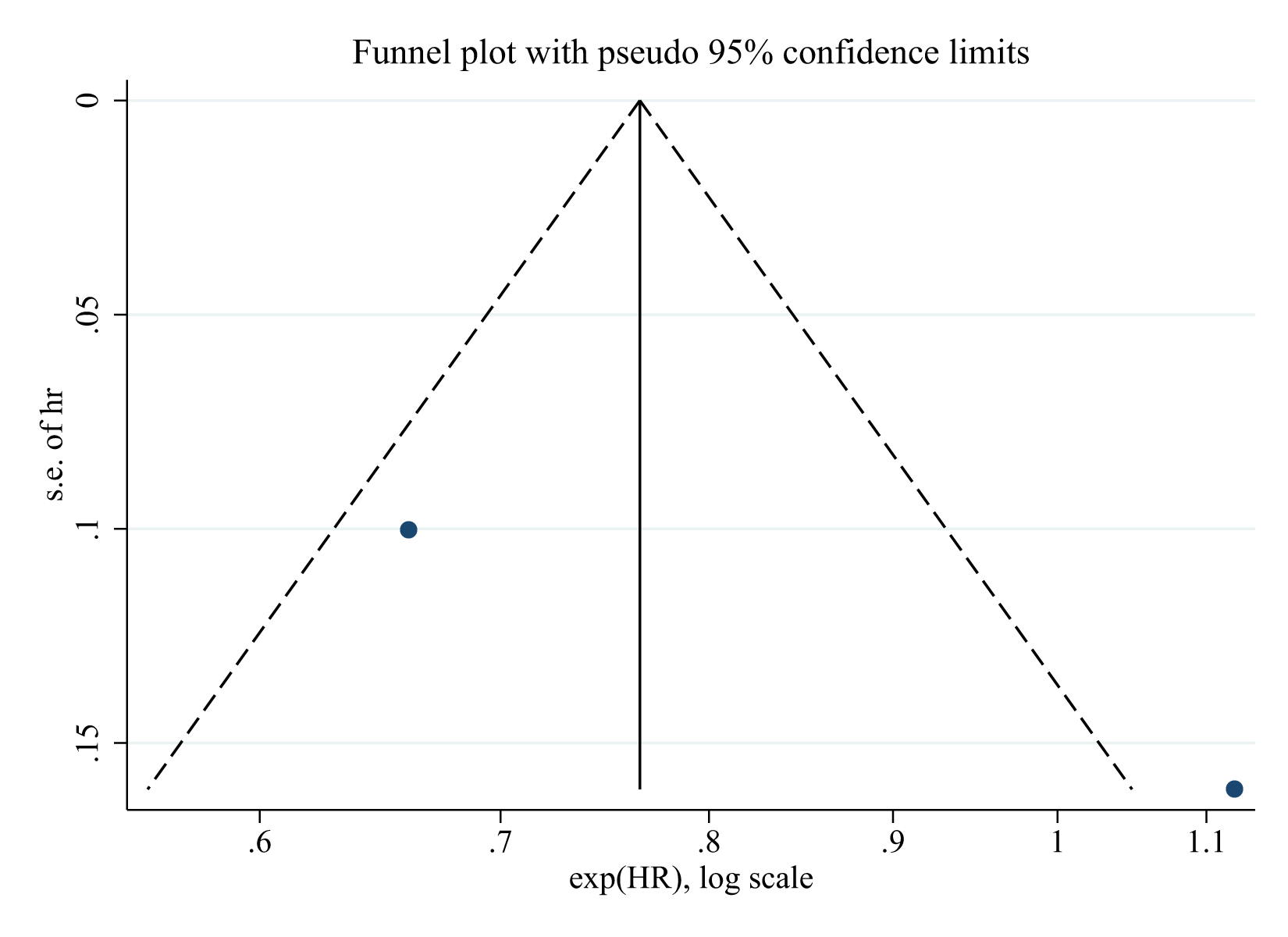


Figure C.95 Funnel plot of disease-free survival in pregnant BC patients who had an interval between diagnosis and pregnancy ＞2 years compared with non-pregnant BC patients


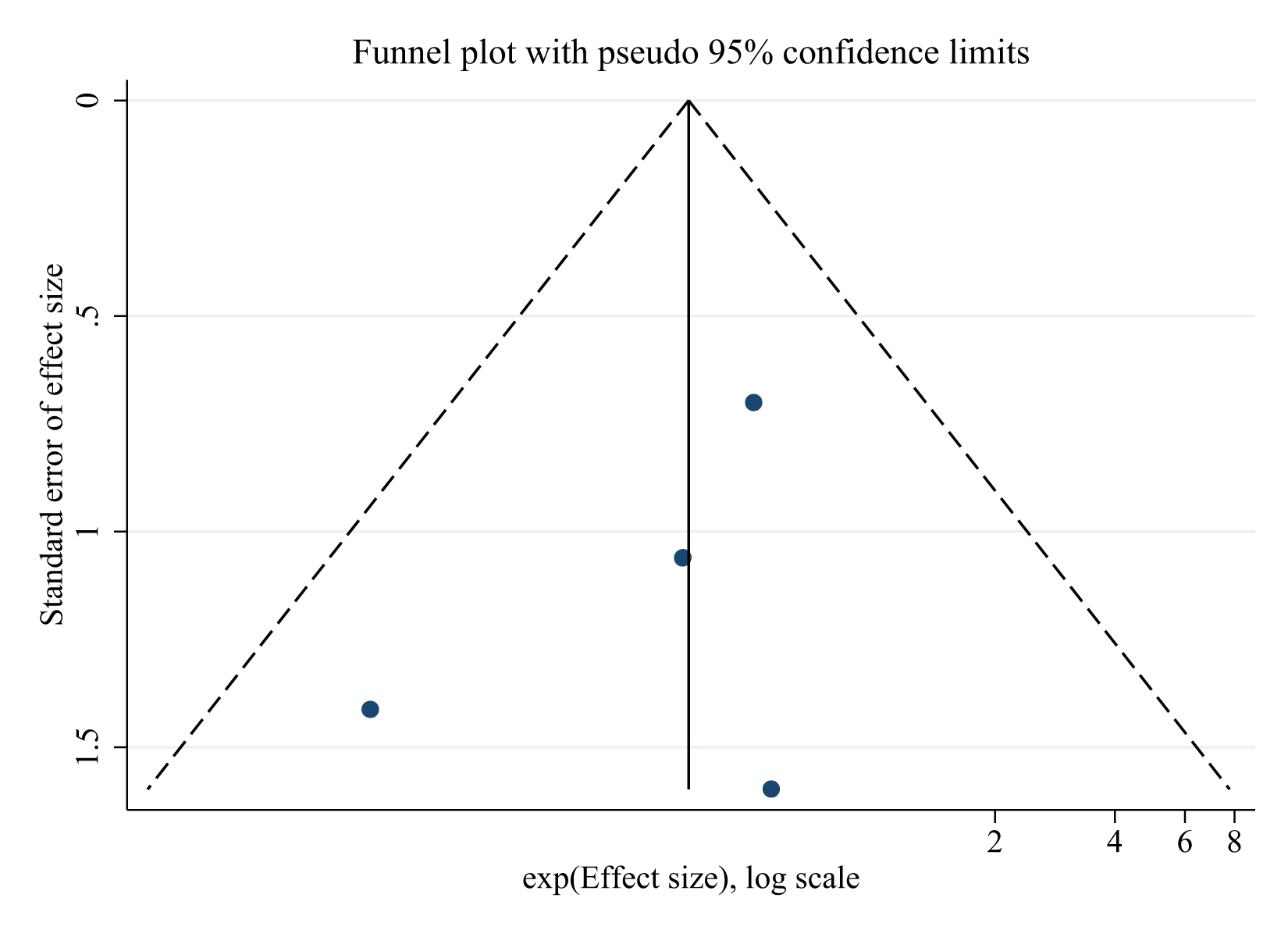


Figure C.96 Funnel plot of recurrence rate in pregnant BC patients who had received assisted reproductive technology compared with pregnant BC patients who had received spontaneous conception


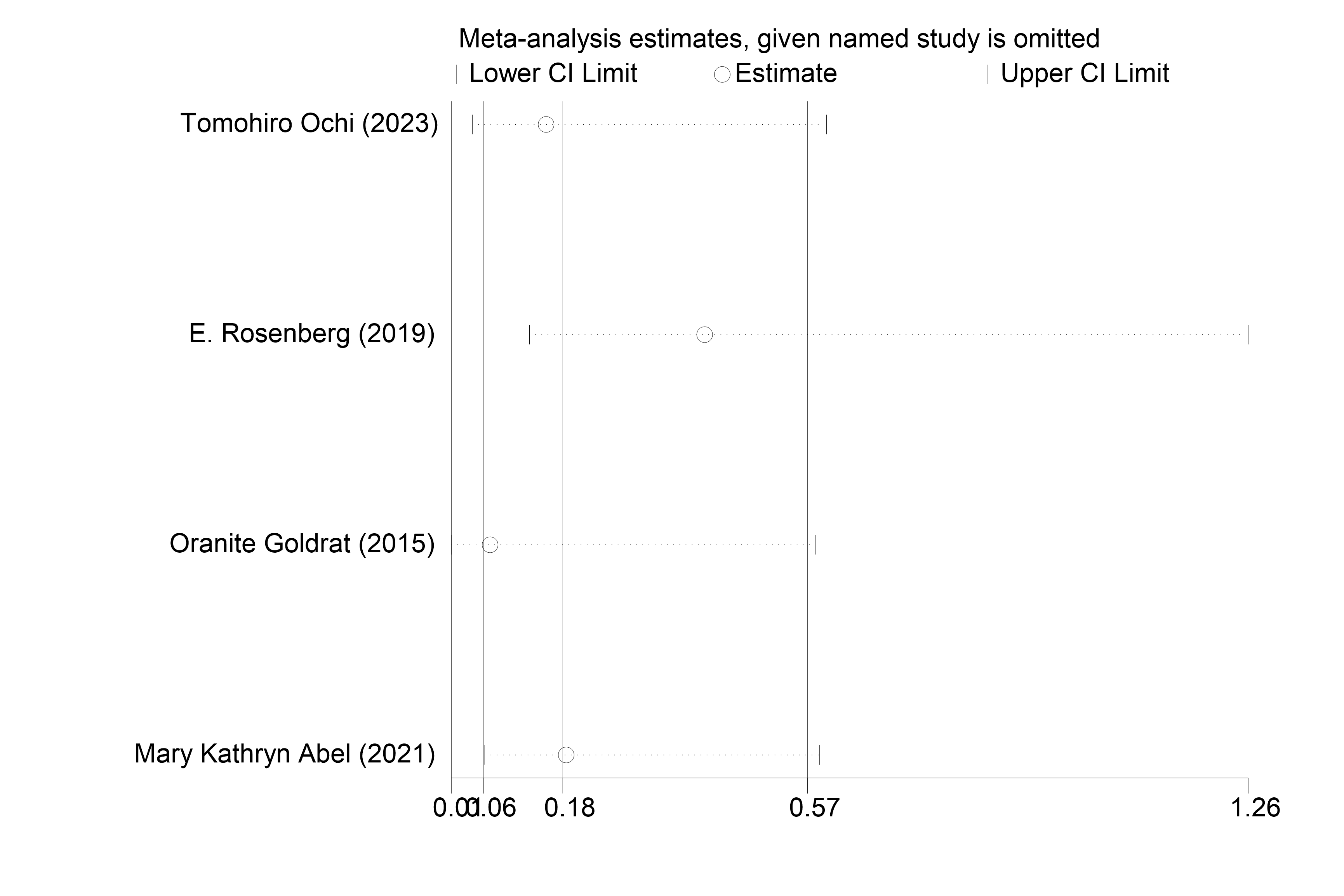


Figure C.97 Sensitivity analysis of recurrence rate in pregnant BC patients who had received assisted reproductive technology compared with pregnant BC patients who had received spontaneous conception


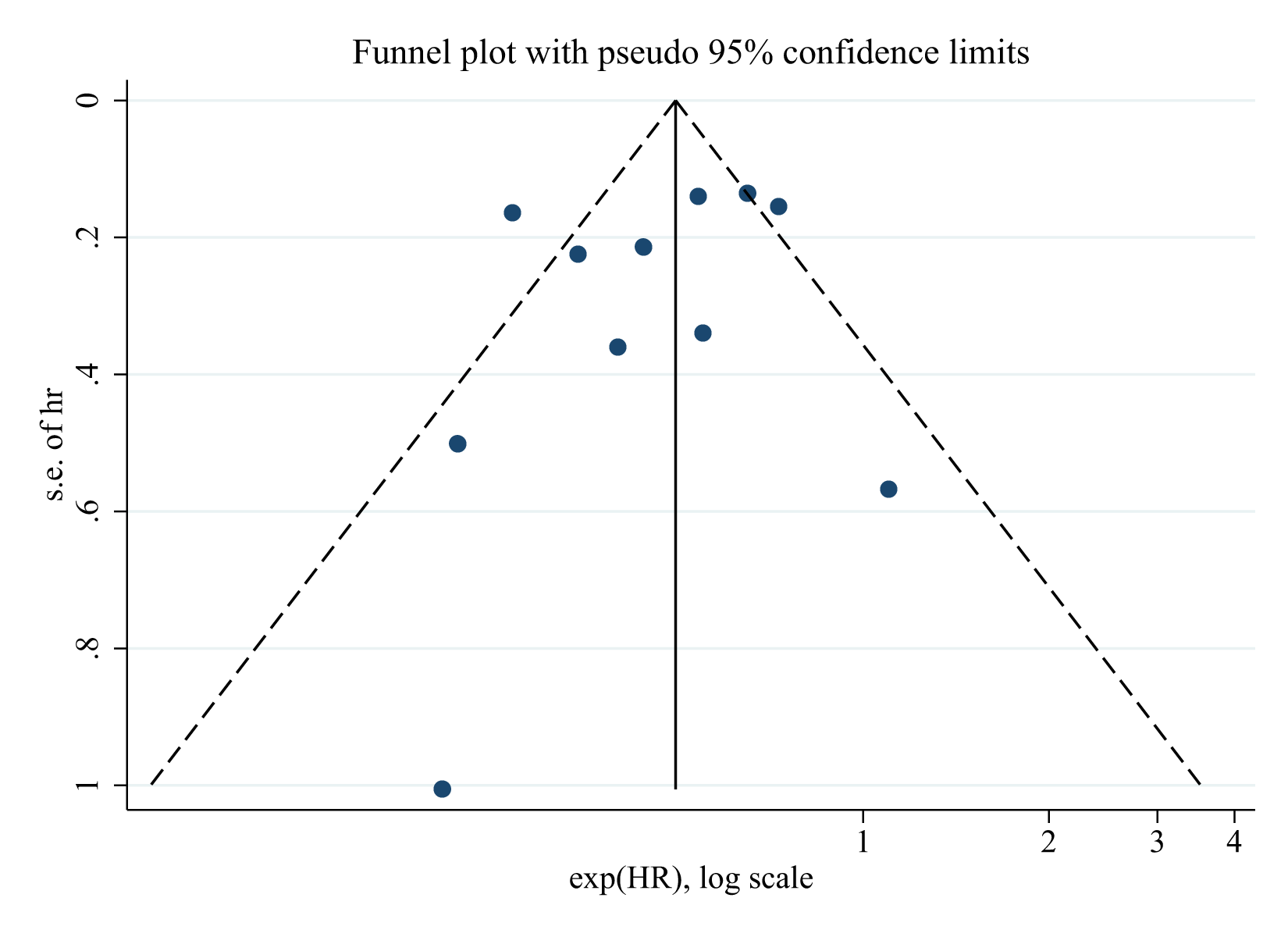


Figure C.98 Funnel plot of overall survival in BC patients with full-term pregnancy compared with non-pregnant BC patients


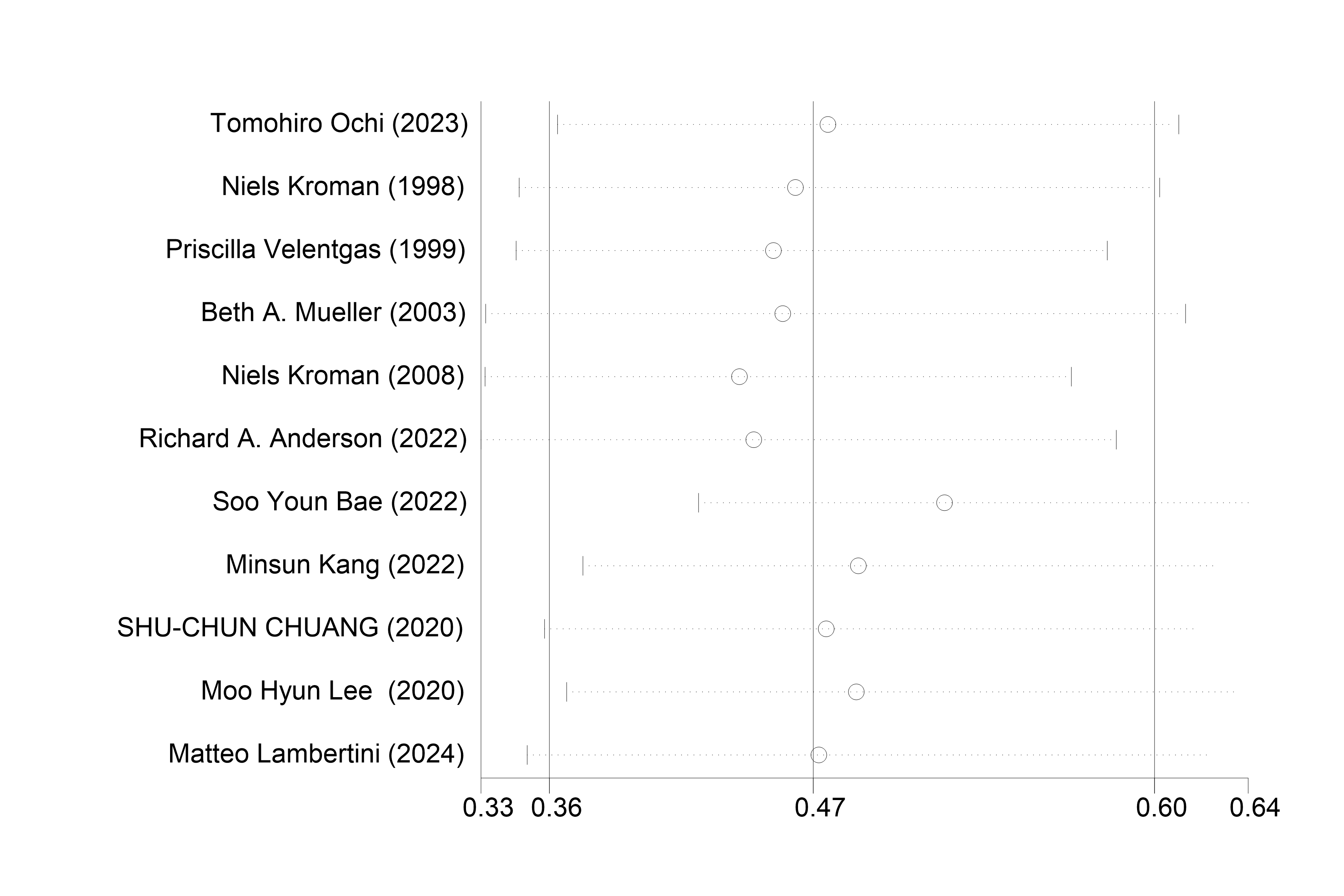


Figure C.99 Sensitivity analysis of overall survival in BC patients with full-term pregnancy compared with non-pregnant BC patients


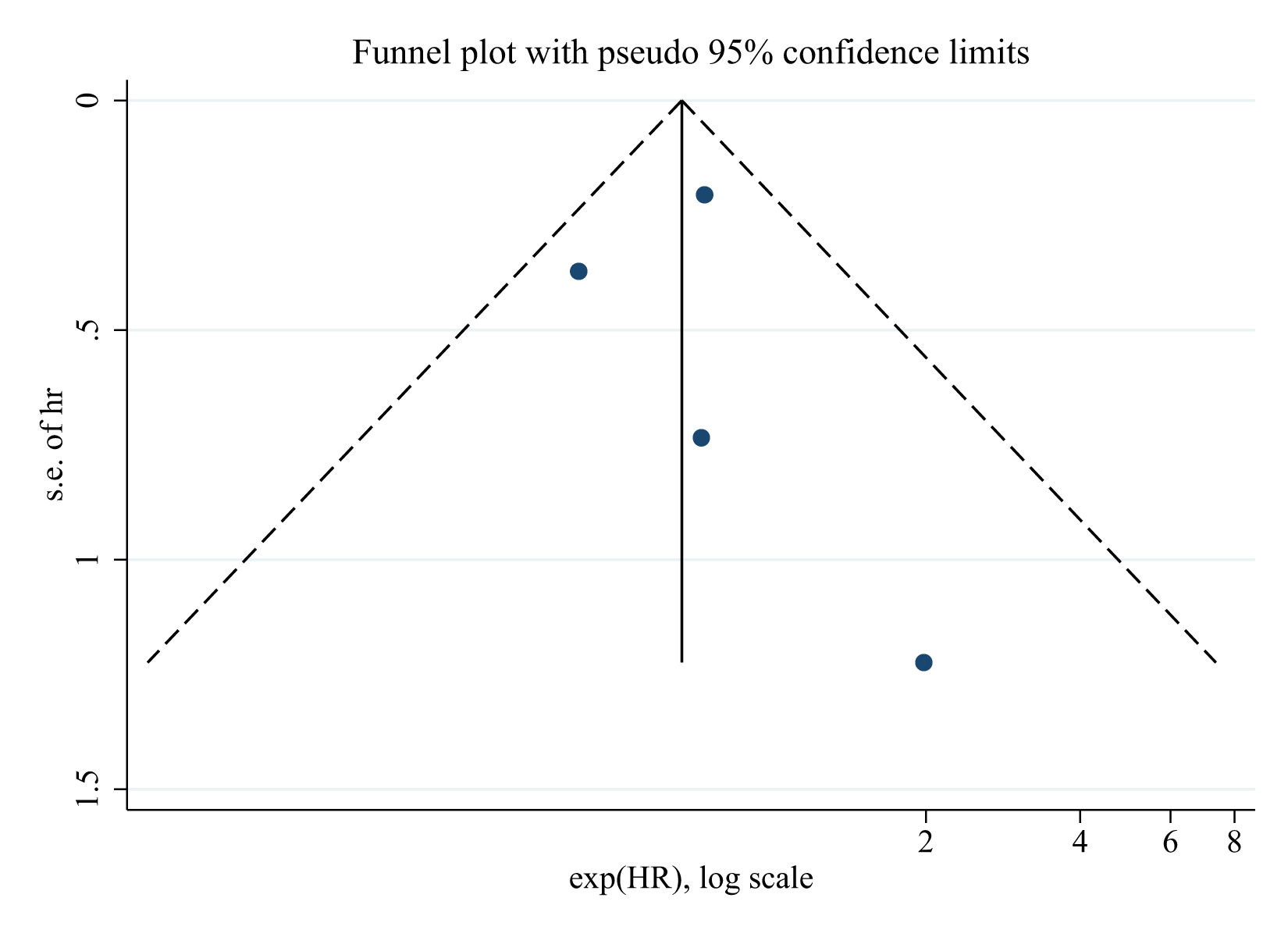


Figure C.100 Funnel plot of overall survival in BC patients with spontaneous or induced abortions compared with non-pregnant BC patients

Figure C.101 Sensitivity analysis of overall survival in BC patients with spontaneous or induced abortions compared with non-pregnant BC patients

Figure C.102 Funnel plot of disease-free survival in pregnant BC patients who had breastfed their newborns compared with non-pregnant BC patients

Figure C.103 Funnel plot of disease-free survival in pregnant BC patients who had not breastfed their newborns compared with non-pregnant BC patients
